# Aminofluorination: transition-metal-free N–F bond insertion into diazocarbonyl compounds†
†Electronic supplementary information (ESI) available: Experimental procedures, and data for new compounds. CCDC 1417241. For ESI and crystallographic data in CIF or other electronic format see DOI: 10.1039/c5sc04237b



**DOI:** 10.1039/c5sc04237b

**Published:** 2015-12-14

**Authors:** Gui Chen, Jinshuai Song, Yinghua Yu, Xuesong Luo, Chunsen Li, Xueliang Huang

**Affiliations:** a Key Laboratory of Coal to Ethylene Glycol and Its Related Technology , Fujian Institute of Research on the Structure of Matter , Chinese Academy of Sciences , Yangqiao west road 155# , Fuzhou , Fujian 350002 , China . Email: huangxl@fjirsm.ac.cn ; http://www.fjirsm.cas.cn/yjxt/yyhxyjzs/hxlktz/; b State Key Laboratory of Structural Chemistry , Fujian Institute of Research on the Structure of Matter , Chinese Academy of Sciences , Yangqiao west road 155# , Fuzhou , Fujian 350002 , China . Email: chunsen.li@fjirsm.ac.cn

## Abstract

Gem-aminofluorination of diazocarbonyl compounds has been achieved for the first time. This reaction proceeds under mild conditions and does not require any transition-metal promoter or catalyst. Treatment of diazoesters with *N*-fluorobenzenesulfonimide (NFSI), which serves as both a fluorine and nitrogen source, results in the facile construction of C–N and C–F bonds, providing aminofluorination products in moderate to excellent yields. Kinetic studies and DFT calculations have provided valuable insight into the potential mechanism for this novel N–F bond insertion.

## Abstract 2

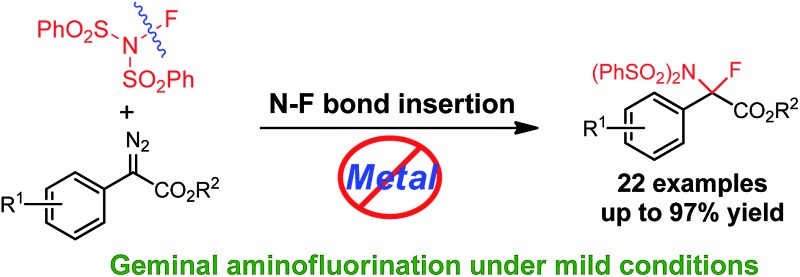
Gem-aminofluorination of diazocarbonyl compounds has been achieved for the first time.

## Introduction

**Scheme 1:**
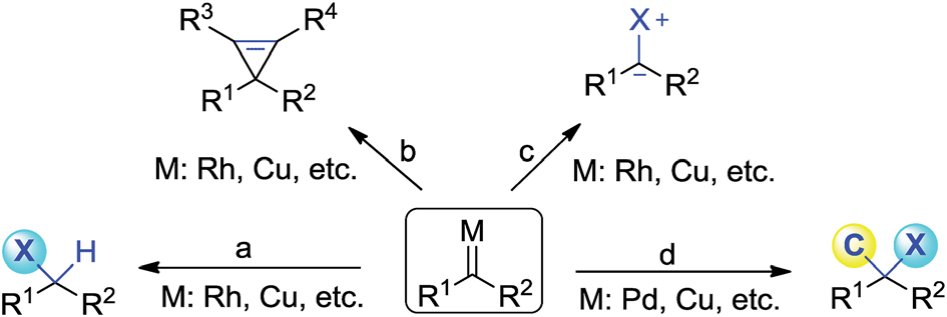
Representative reaction modes of a transition-metal carbenoid.

Diazoacetate derivatives play an important role in the synthetic community. Over the past century, significant advances have been made towards the generation of carbenoid intermediates triggered by transition-metals. The resulting reactive species can undergo valuable transformations, such as three-membered ring (cyclopropane, cyclopropene) formation, X–H bond insertion (X = C, N, O, S, *etc.*) and ylide generation (Scheme 1a–c).^1^ van Vranken, Barluenga, Wang, and others have developed palladium- or copper-catalysed multiple component reactions of diazo compounds, which allow the installation of two separated functional moieties on the carbenic carbon *via* a single operation (Scheme 1d).^2^ Despite these important advances, the direct introduction of two functional groups to the same carbon center, namely gem-difunctionalization of donor/acceptor (D/A) carbenes, is still far from well developed.^3,4^ It is of note that these processes at least involve one C–C bond formation.^5^ Thus the studies on the gem-difunctionalization of D/A carbenes, which involves two distinct carbon heteroatom bond formations, would greatly enhance the synthetic applications of diazo compounds.

**Scheme 2:**
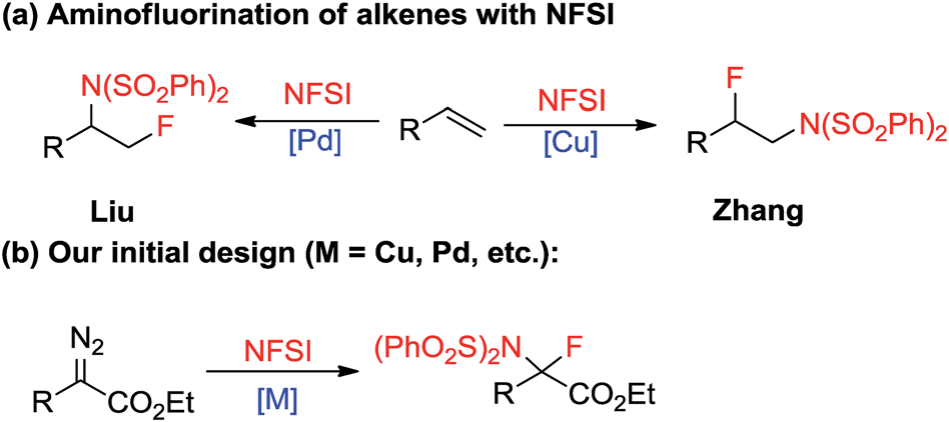
Aminofluorination catalysed by transition-metals.

Single substitution of hydrogen with fluorine may alter the chemical and physical properties of a potential drug candidate by blocking undesired metabolism at a specific site.^6^ In contrast to the relatively large number of reports on the catalytic insertion of N–H bonds to α-diazocarbonyl compounds,^7^ a simple yet appealing concept for the transition-metal catalysed N–F bond insertion has not been realized thus far. *N*-Fluorobenzenesulfonimide (NFSI) is inexpensive and shelf stable, and is often employed as a mild electrophilic fluorinating or aminating reagent.^8,9^ Recently, Liu^10^ and Zhang^11^ demonstrated that NFSI could serve as both an amino and fluorine source for the transition-metal-catalysed aminofluorination of alkenes (Scheme 2a). Inspired by these seminal works, we envisioned that NFSI might be an ideal candidate for the transition-metal catalysed N–F bond insertion into D/A carbenes (Scheme 2b). Although procedures for the amination^7,12^ or fluorination^13,14^ of diazo compounds are known, to our knowledge, direct aminofluorination of diazo compounds remains unexplored. Herein, we present our primary results on gem-aminofluorination of diazocarbonyl compounds under mild conditions. Kinetic studies and DFT calculations shed light on the mechanism of the current N–F bond insertion.

## Results and discussion

### Optimization studies and substrate scope

We initiated our studies using the reaction of ethyl diazophenyl acetate **1a** (ref. 15) with NFSI using CuBr as a precatalyst and bathocuproine (2,9-dimethyl-4,7-diphenyl-1,10-phenanthroline; BC) as a ligand.^11^ Gratifyingly, in the presence of 5 mol% CuBr and 6 mol% BC, ethyl diazophenyl acetate **1a** was completely consumed after stirring in a reaction medium of 1,2-dichloroethane (DCE) at 60 °C for 21 h, and the desired product **2a** was obtained in a 79% NMR yield (Table 1, entry 1). To our surprise, the reaction proceeded equally well in the absence of both CuBr and BC, giving **2a** in 89% yield (Table 1, entry 2). The reaction could complete in a comparably short time when the reaction was carried out at an elevated temperature (Table 1, entries 2–4 *vs.* entry 5). A brief examination of the solvent effects revealed that DCE was still the best choice (Table 1, entries 6–14). Notably, the reaction can also be performed in water, giving **2a** in a moderate yield, which indicates an environmentally benign perspective (Table 1, entry 15). Due to the similar polarity between **2a** and NFSI, a slight excess of **1a** (1.5 eq.) was necessary to make sure that NFSI reached full conversion. In this case, **2a** was isolated in a nearly quantitative yield (Table 1, entry 16).

**Table 1:** Optimization of the reaction conditions

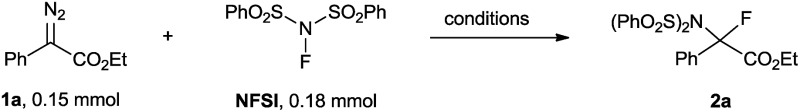				
Entry^*a*^	Solvent	Temp (°C)	Time (h)	Yield^*b*^ (%)
1^*c*^	DCE	60	21	79
2	DCE	60	21	89
3	DCE	80	7	77
4	DCE	100	4	73
5	DCE	RT	24	17
6	CH_2_Cl_2_	60	24	81
7	CHCl_3_	60	24	70
8	Toluene	60	24	58
9	MeCN	60	24	69
10	THF	60	24	47
11	Dioxane	60	24	22
12	DMF	60	24	0
13	MeOH	60	24	0
14	H_2_O	60	12	62
15^*d*^	DCE	60	24	82
16^*e*^	DCE	60	48	97^*f*^

^*a*^All reactions were carried out under an atmosphere of argon in 0.15 mmol scale, [**1a**] = 0.10 M, [NFSI] = 0.12 M.

^*b*^Yields were determined by ^19^F NMR, using 1-bromo-4-fluorobenzene as the internal standard.

^*c*^In the presence of 5 mol% CuCl and 6 mol% BC.

^*d*^Under air.

^*e*^The reaction was carried out in 0.3 mmol scale, [**1a**] = 0.15 M, [NFSI] = 0.10 M.

^*f*^Isolated yield.

With the optimized reaction conditions in hand (Table 1, entry 16), the generality and limitations of this metal-free N–F bond insertion were investigated. The results are summarized in Table 2. Most of the α-diazoacetates reacted with NFSI smoothly to give the corresponding products in moderate to excellent yields. When changing the ester group (R^2^) from methyl to isobutyl, or benzyl, no significant decrease of the product yields was observed (Table 2, **2a–d**). When the diazo compound contained a chiral auxiliary, an ester derived from (+)-menthol, a pair of diastereoisomers **2e** were obtained in a ratio of 1 : 1. Interestingly, diazo compounds derived from cyclic esters and amides were proven to be viable substrates. N–F bond insertion of **1f** and **1g** gave the corresponding products **2f** and **2g** in 97% and 83% yields, respectively. The structure of **2f** was confirmed by X-ray crystallographic analysis.^16^ The effects of substituents on the phenyl ring were also examined. Both electron-withdrawing and electron-donating groups on the aromatic ring of **1** were tolerated under the reaction conditions. The reaction of diazo acetates bearing mild electron-withdrawing or electron-donating groups (fluoro, chloro, bromo and methyl) at the *para* position of the phenyl ring gave the corresponding products in excellent yields (Table 2, **2h**, **2i**, **2m** and **2o**). Incorporation of one chloro group to the *meta* position had no obvious impact on the yields of the products (Table 2, **2i** and **2k**). Interestingly, the yield of **2l** bearing two *meta* chloro substituents was decreased to 52%. While the diazo acetate bearing an *ortho* substituted group was not compatible to the current conditions (Table 2 and **2p**), probably due to the steric hindrance effect. Similarly, strong electron-donating or -withdrawing substituents on the aromatic ring were amenable for the current aminofluorination, albeit giving moderate yields (Table 2, **2n**, **2r–t**). Of note, vinyl and alkyl moieties remained intact (Table 2, **2v** and **2w**), indicating that the involvement of a free carbene intermediate is less likely (*vide infra*). Notably, the reaction could be scaled up to gram scale without sacrificing the yield of **2a** (5 mmol scale, 2.39 g **2a** was obtained with a 97% yield). It is worthwhile to mention that the current conditions are not applicable to alkyl or heteroaryl acetate derived diazo compounds.^17^

**Table 2:** Geminal aminofluorination of various diazocompounds^*a*^

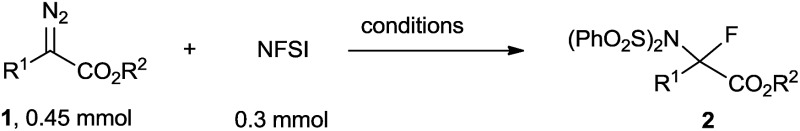
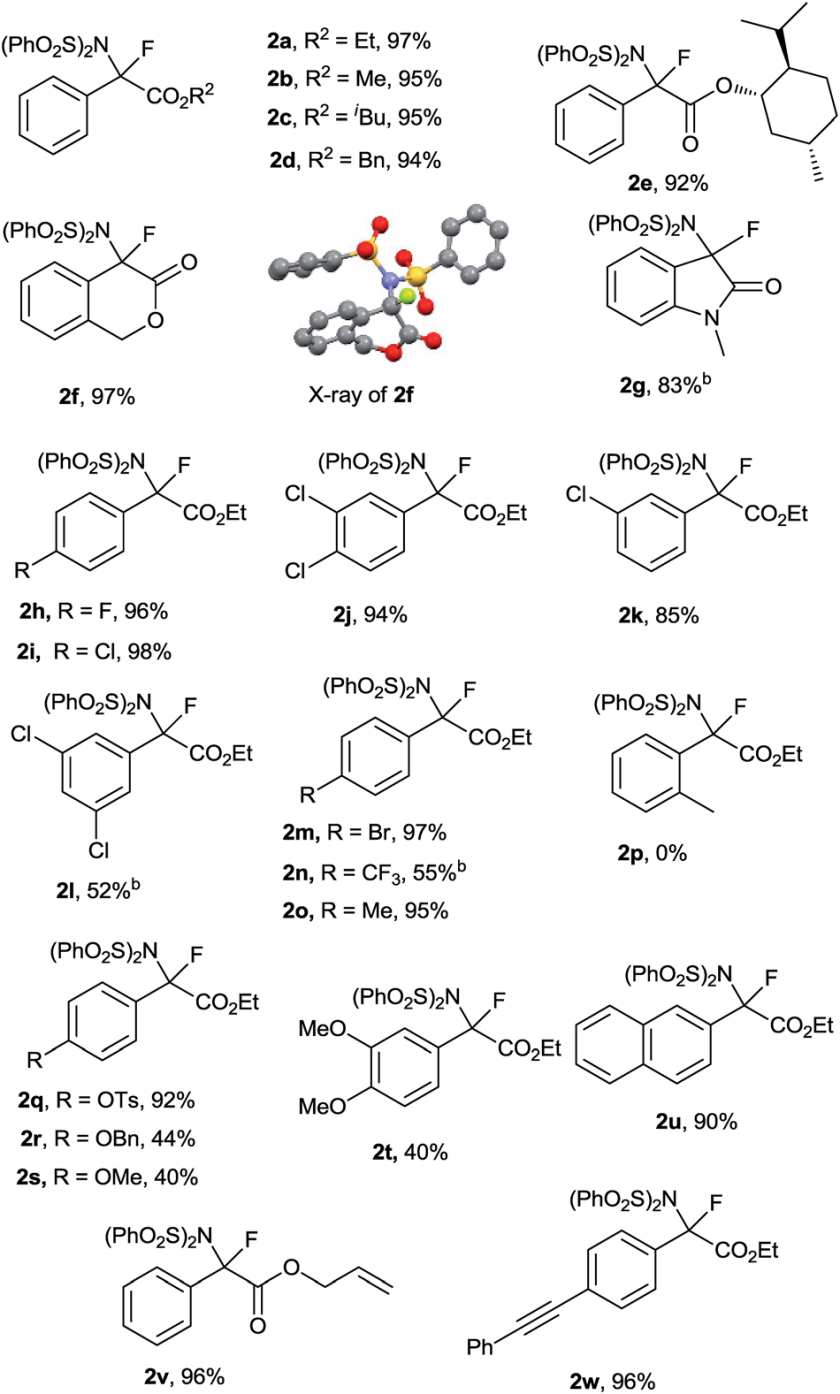

^*a*^All reactions were carried out in 0.30 mmol scale under argon at 60 °C, [**1a**] = 0.15 M, [NFSI] = 0.10 M, isolated yield.

^*b*^Reaction was carried out at 80 °C.

### Mechanistic studies

**Scheme 3:**
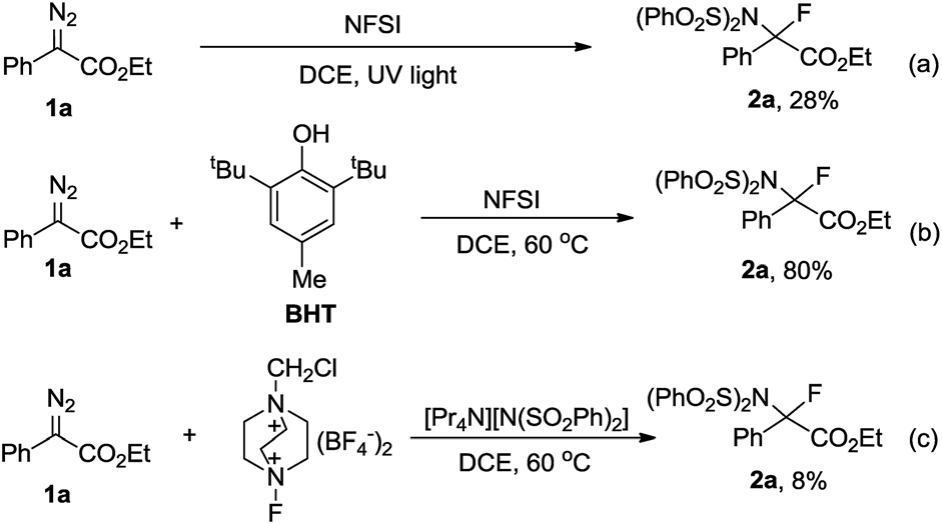
Preliminary mechanistic studies.

Having uncovered an efficient method for N–F bond insertion, we sought to gain more insight into the reaction mechanism. Thus a series of additional experiments were subsequently carried out. Performing the reaction under irradiation by UV light, **2a** was obtained in a much lower yield (Scheme 3a). Furthermore, evolution of N_2_ was significantly slow in the absence of NFSI under otherwise identical conditions (Fig. S1†). These experiments also suggest that a pathway *via* a free carbene intermediate is unfavourable. Addition of the radical scavenger 2,6-di-*tert*-butyl-4-methylphenol (BHT) had no obvious influence on the reaction efficiency (Scheme 3b), which indicates that a mechanism that involves free radical species is also less likely. Additionally, considering the diazo carbon atom is mildly nucleophilic and might be trapped by the electrophilic fluorine atom, a subsequent displacement of dinitrogen by the imide moiety of NFSI would give **2a**. With this consideration in mind, the following control experiment was carried out immediately. The combination of selectfluor, a reactive electrophilic fluorinating reagent, with tetrapropyl ammonium benzenesulfonimide did afford **2a** in 8% yield (Scheme 3c).

**Fig. 1:**
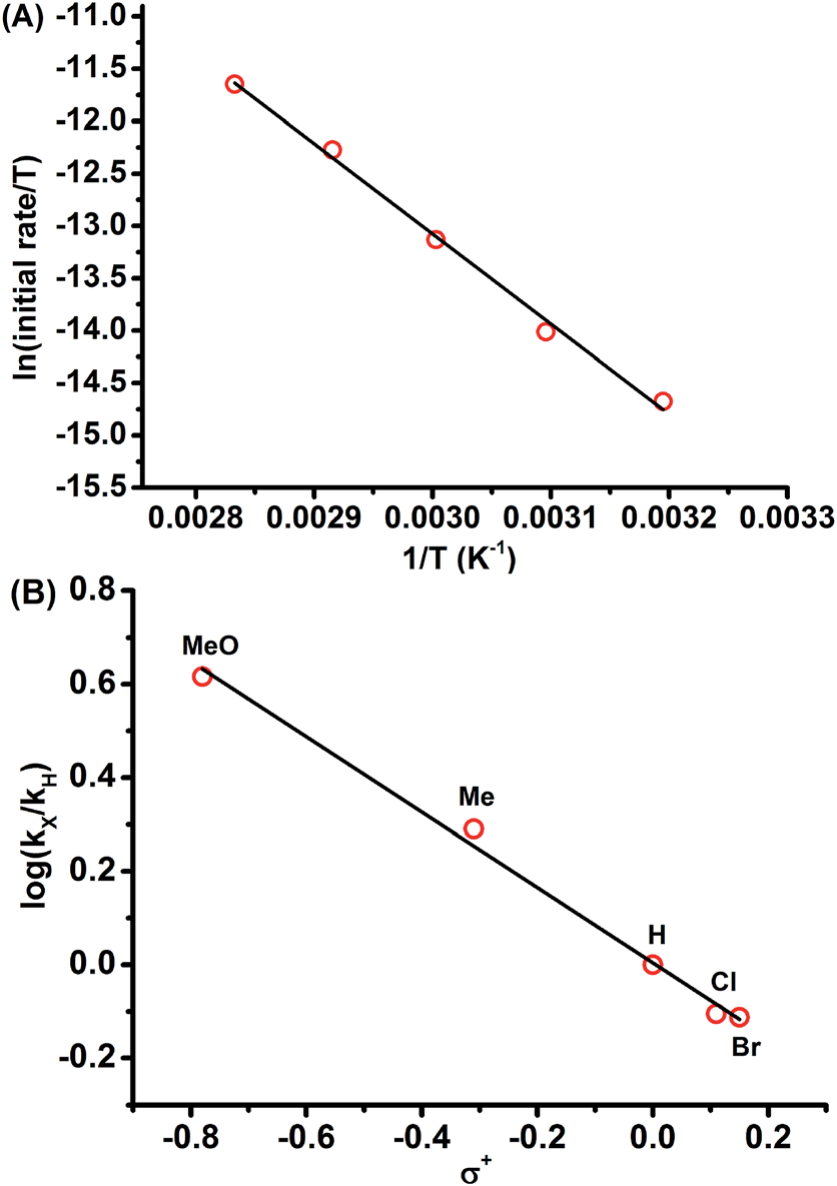
(A) (top) Plot of ln(initial rate/*T*) *vs.* 1/*T* for the reaction between **1a** and NFSI in DCE, [**1a**] = 0.15 M, [NFSI] = 0.1 M, slope = –8.61 × 10^3^, *y*-intercept = 1.27 × 10, *r* ^2^ = 0.996. (B) (bottom) Hammett plot of log(*k*_X_/*k*_H_) *vs. σ* ^+^ for the reaction of NFSI with *para* substituted diazo phenyl acetates **1** in DCE at 50 °C, [**1**] = 0.15 M, [NFSI] = 0.1 M, slope = –0.81, *y*-intercept = 0.4 × 10^–2^, *r* ^2^ = 0.995.

Kinetic studies for the reaction of **1a** with NFSI were further performed to get a deeper understanding of the reaction mechanism. The reaction was found to be first-order in both **1a** and NFSI (Fig. S2–S5†). The activation parameters Δ*H* ^‡^ = 17.1 kcal mol^–1^ and Δ*S* ^‡^ = –13.0 cal mol^–1^ K^–1^ were obtained from Eyring plots by varying the temperature from 313 to 353 K (Fig. 1A, top). The negative Δ*S* ^‡^ value may suggest the generation of a bimolecular transition state involving NFSI and **1a**. Similar kinetic behaviours were observed for the reactions of various *para*-substituted diazo phenyl acetates **1** with NFSI. A fairly linear Hammett correlation between log(*k*_X_/*k*_H_) and *σ* ^+^ was obtained with a reaction constant of *ρ* = –0.81 (Fig. 1B, bottom). The small negative *ρ* value suggests that the transition state is weakly polarized with a positive charge at the reaction center.^18,19^

**Scheme 4:**
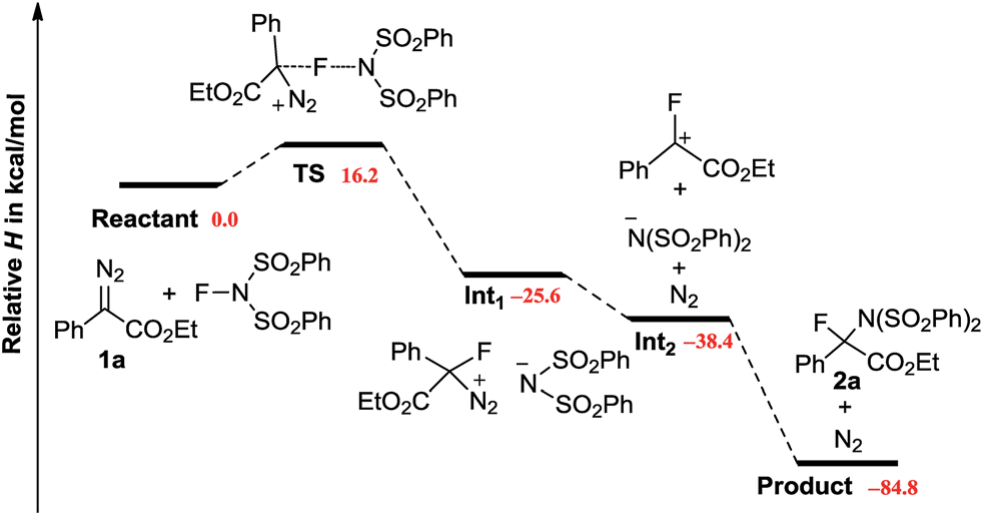
Mechanistic rationale supported by DFT calculations.

DFT calculations were carried out to provide more details on the potential mechanism (Scheme 4).^20^ The reaction of **1a** with NFSI encounters an activation enthalpy of 16.2 kcal mol^–1^ with a slight loss of entropy (Δ*S* ^‡^ = –9.0 cal mol^–1^ K^–1^), which matches well with the experimental values (*vide supra*). According to the downhill energy profile, the following steps are rather facile. The weakly polarized **TS** further collapses to an ion pair **Int1** with an energy change of –41.8 kcal mol^–1^. Releasing N_2_ from **Int1** has been proven to be an exothermic process. Finally, an S_N_1 like reaction takes place to give **2a**, with a total enthalpy change of –84.8 kcal mol^–1^. It is of note, that the formation of a procarbonium ion through the reaction of diazo carbon with Cl^+^, Br^+^ and I^+^ has been reported recently.^21^ However, in our case, the replacement of NFSI by NXS (X = Cl, Br, I) results in no formation of the aminohalogenated product. It is well known that diazo compounds are not stable in the presence of a strong acid. Indeed, addition of strong Brønsted acids (CCl_3_CO_2_H or CF_3_CO_2_H) led to the rapid decomposition of **1a**. However, the addition of acetic acid had no influence on the current N–F bond insertion.^22,23^

## Conclusions

In conclusion, we have developed an unprecedented N–F bond insertion into diazocarbonyl compounds. The current method represents a facile approach to construct C–N and C–F bonds on the same carbon without any transition-metal as a catalyst or promoter. Mechanistic studies, including kinetic experiments and DFT calculations, revealed that a reaction sequence of electrophilic activation of **1** by NFSI, followed by an S_N_1 like displacement of dinitrogen by benzenesulfonimide is preferred. Further study on the asymmetric variant as well as the difunctionalization of the metal carbene is ongoing in our laboratory.
