# Supplementary material for: Aminofluorination: transition-metal-free N–F bond insertion into diazocarbonyl compounds
Source: Chem Sci. 2015 Dec 14;7(3):1786–90. doi: 10.1039/c5sc04237b (PMC5595123; doi:10.1039/c5sc04237b)
Supplement: Supplementary file 1 [file SC-007-C5SC04237B-s001.pdf]

Electronic Supplementary Material (ESI) for Chemical Science.
This journal is © The Royal Society of Chemistry 2015

Supplementary information

Aminofluorination: Transition-Metal-Free N-F Bond Insertion of Diazocarbonyl Compounds

Gui Chen[a], Jinshuai Song[b], Yinghua Yu[a], Xuesong Luo[a], Chunsen Li[*b] and Xueliang Huang[*a]

[a]*Key Laboratory of Coal to Ethylene Glycol and Its Related Technology, Fujian Institute of Research on the Structure of Matter, Chinese Academy of Sciences, Fujian, Fuzhou, 350002, P. R. China*

[b]*State KeyLaboratory of Structural Chemistry, Fujian Institute of Research on the Structure of Matter,Chinese Academy of Sciences, Fujian, Fuzhou, 350002, P. R. China*

chunsen.li@fjirsm.ac.cn, huangxl@fjirsm.ac.cn

Table of content

General information 3
General procedure for preparation of diazoesters 3
General procedure for the germinal aminofluorination of diazoesters 4
Nitrogen evolution 7
Kinetic Studies 7
Computational method 12
Calculated potential energy surface 13
^{1}H and ^{13}C NMR Spectra datafor the prepared substrates 17
^{1}H, ^{13}C and ^{19}F NMR Spectra data for the prepared products 23
Referecnes 34
^{1}H and ^{13}C NMR spectra for the prepared substrates 35
^{1}H, ^{13}C and ^{19}F NMR spectra for the prepared products 57

Table S1. Geminal aminofluorination of various diazocarbonyl compounds………..6

Table S2. B3LYP calculated transition state energies in the first step of four possible pathways………………………………………………………………………….…..13

Figure S1. Plots of nitrogen evolution vs. time for the decomposition of **1a** in the presence of NFSI or without NFSI in DCE at 60 ^{o}C……………………………..…...7

Figure S2. Plots of concentration of **2a** vs time for the reaction between diazoester **1a** and NFSI in DCE at 50 ^{o}C…………………………………………………………..…..8

Figure S3. Plots of concentration of **2a** vs time for the reaction between diazoester **1a** and NFSI in DCE at 50 ^{o}C………………...…………………………………………..…9

Figure S4. Plot of ln(initial rate) vs ln([NFSI])………………………………………9

Figure S5. Plot of ln(initial rate) vs ln([1a])…………………………………...…10

Figure S6.Plot of ln(initial rate/T) vs 1/T for the reaction between **1a** and NFSI in DCE……………………..……………………………………………………………11

Figure S7. Hammett plot of log(k_X/k_H) vs σ^+ for the reaction of NFSI with *para*-substituted diazophenylacetates **1** in DCE at 50 ^{o}C….……..…….….……………12

Figure S8. Geometries of four transition states in Scheme S1……………….……...15

Figure S9. DFT computed enthalpy change (red text, in kcalmol^{-1}) for the reaction between **1a** and NFSI……………………………………………………………..…..16

Scheme S1. The first step of the four possible transformation pathways from reactant complex **1a** to product **2a**………………………………………………………..…13

General information

All reactions were carried out with standard Schlenk techniques under argon. All reagents were used as received from commercial suppliers unless otherwise stated. All solvents were purified by distillation following standard procedures. Reaction progress was monitored by thin layer chromatography (TLC) and components were visualized by observation under UV light at254nm.Flash column chromatography was performed using silica gel 60 (200-300 mesh). All ^{1}H NMR, ^{13}C NMR and ^{19}F NMR spectra were recorded on Bruker AV-III 400 in $CDCl_3$. Chemical shifts were reported in parts per million (ppm, δ). Proton nuclear magnetic resonance (^{1}H NMR) spectra is referenced to the peak of tetramethylsilane (δ = 0.00) and reported as follows: chemical shift (ppm), multiplicity (s = singlet, t = triplet, q = quartet, m = multiplet) and coupling constant (Hz). Carbon-13 nuclear magnetic resonance (^{13}C NMR) spectra is referenced tothe solvent center peak of $CDCl_3$ (δ = 77.0).

CAUTION! Even though we have noted no explosive tendencies of the diazo compounds, it is strongly recommended that they should be handled with great care and proper protection.

General procedure for preparation of diazoesters

Method A:

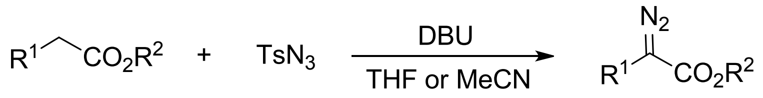

According to a known procedure[1-3], to the solution of ethyl phenylacetate (5mmol) and 4-methylbenzenesulfonyl azide (TsN_3) (1.24 g, 6mmol) in anhydrous CH_3CN or THF (40 mL) was added 1,8-diazabicyclo-[5.4.0]-undec-7-ene (DBU) (1.14 g, 7.5 mmol) slowly at room temperature. Then the reaction mixture was stirred at room temperature for 15 hours. After water (40 mL) was added, the resulting

mixture was extracted with diethyl ether (3 × 40 mL). The combined organic layer was washed with brine (20 mL) and dried over anhydrous Na_2SO_4. After removing the solvent under reduced pressure, the residual was purified by column chromatography on silicagel to give **1a-1k**, **1m**, **1o-1q**, **1s-1v**.

Method B:

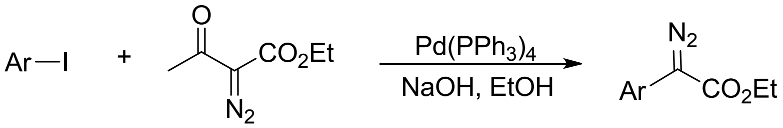

According to a known procedure[4], $Pd(PPh_3)_4$ (144 mg, 5 mol%), NaOH (300.0 mg, 7.5 mmol) were suspended in ethanol (10.0 mL) and stirred for 30 minunder argon. Then aryl iodide (2.5 mmol) and ethyl 2-diazo-3-oxobutanoate (468 mg, 3.0 mmol) was added. The resulting solution was stirred at room temperature for 5 h. The mixture was filtered through a short path of silica gel with ethyl acetate as eluent. After removing the solvent under reduced pressure, the residual was purified by column chromatography on silica gel to give the products **1l**, **1n** and **1w**.

General procedure for the germinalaminofluorination of diazoesters

Method A:

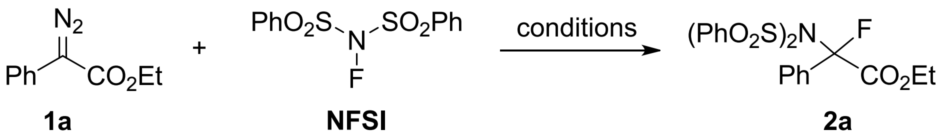

The mixture of diazoesters (0.45mmol) and NFSI (0.3 mmol, 94.6 mg) in 3 mL DCE was stirred for 48 h under argon at 60 °C or 80 °C. After removing the solvent under reduced pressure, the residual was purified by column chromatography on silica gel to give **2**. (**It should be noted that according to the ^{19}F-NMR, the product 2 contains a small amount of impurities, ranging from 1 to 3%, which could not be removed by column chromatography.**)

Method B: irradiating with UV light (Scheme 3a)

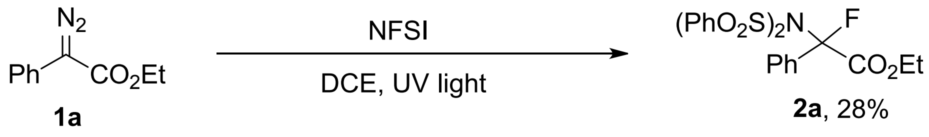

The mixture of diazoester **1a** (0.45mmol, 85.6 mg) and NFSI (0.3 mmol, 94.6 mg) in 3 mL DCE was irradiated under UV light (λ = 254 nm) for 48 h. The yield of **2a** is obtained by ^{19}F NMR Spectroscopy.

Method C: in the presence of radical scavenger (Scheme 3b)

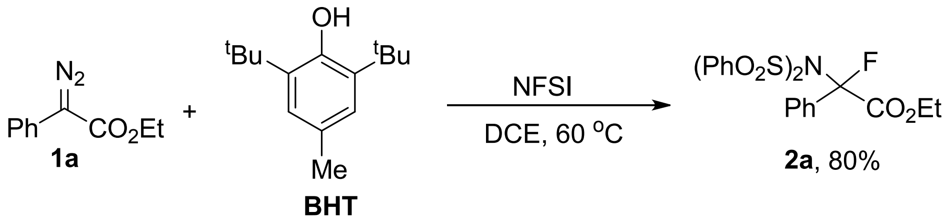

The mixture of diazoester **1a** (0.45mmol, 85.6 mg), NFSI (0.3 mmol, 94.6 mg) and 2,6-di-tert-butyl-4-methylphenol (BHT) (0.3 mmol, 66.1 mg) in 3 mL DCE was stirred for 48 h under argon at 60 °C. After removing the solvent under reduced pressure, the residual was purified by a silica gel column chromatography to give **2a** as white solid.

Method D: using selectfluor as fluorine source (Scheme 3c)

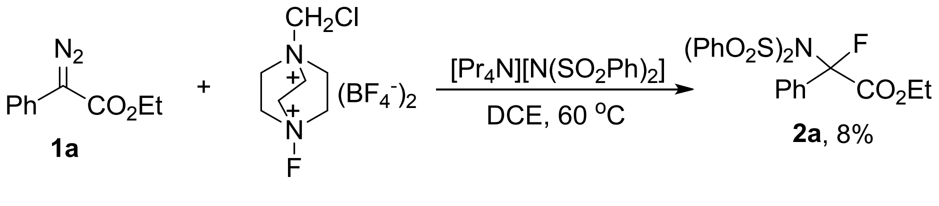

The mixture of diazoester **1a** (0.45mmol, 85.6 mg), selectfluor (0.3 mmol, 106 mg) and tetrapropylammoniumbenzenesulfonimide (0.3 mmol, 131 mg) in 3 mL DCE was stirred for 48 h under argon at 60 °C. The yield of **2a** is obtained by ^{19}F NMR Spectroscopy.

Method E: in the presence of Brønsted acid

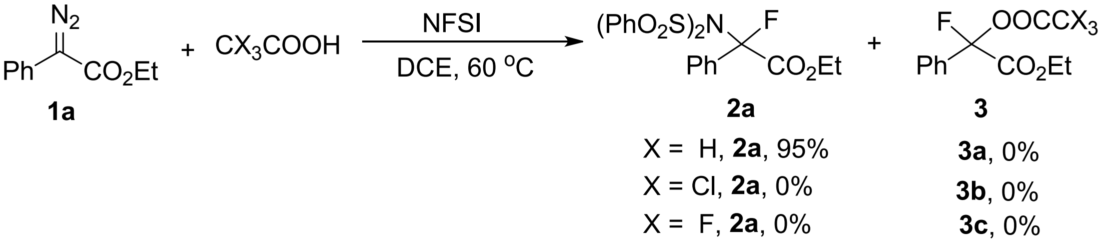

The mixture of diazoester **1a** (0.45mmol, 85.6 mg), NFSI (0.3 mmol, 94.6 mg) and Brønsted acid (2equivalent) in 3 mL DCE was stirred for 48 h under argon at 60 °C. The yield of **2a** is obtained by ^{19}F NMR Spectroscopy.

Table S1. Geminal aminofluorination of various diazocarbonyl compounds.[a]

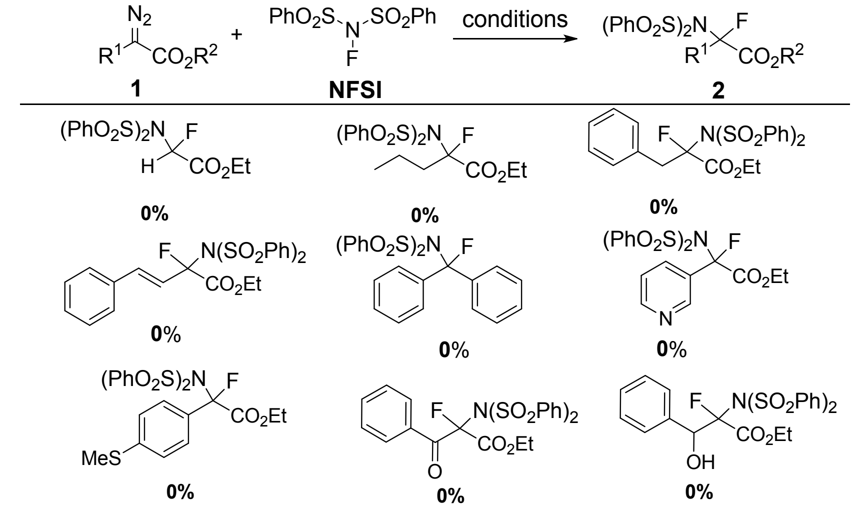

[a]All reaction was carried out in 0.30 mmol scale under argon at 60 °C, [**1a**] = 0.15 M, [NFSI] = 0.10 M, isolated yield.

Nitrogen evolution

The solution of diazoester **1a** (0.45mmol) in 3 mL DCE was placed in a round-bottom flask with NFSI (0.3 mmol, 94.6 mg) or without NFSI. The flask was sealed with rubber septa and deaerated with DCE saturated argon for 15 min. Then, the solution was rigorously stirred at 60 °C. The nitrogen in the headspace was withdrawn by a deaerated Hamilton gas-tight syringe and immediately injected into a HP5890 GC/TCD fitted a Chrompack 5Å molecular sieve column (25 m × 0.32 mm) for analysis and quantified according to a calibration curve prepared by injecting known quantity of pure nitrogen gas into the reaction flask.

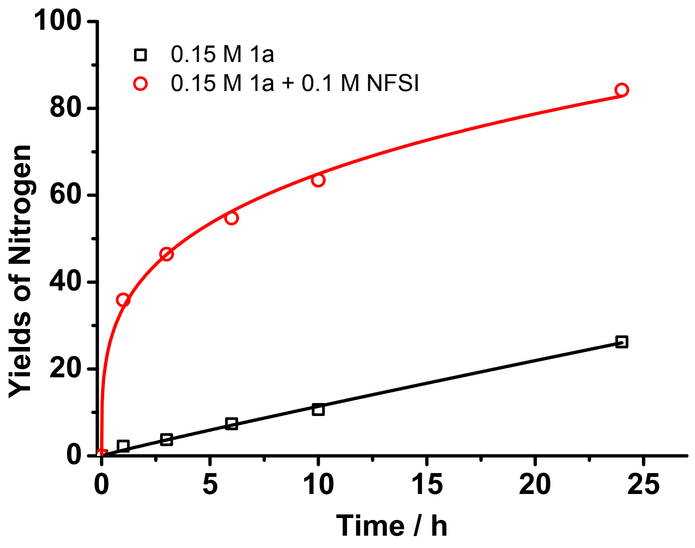

Figure S1. Plots of nitrogen evolution vs. time for the decomposition of **1a** in the presence of NFSI or without NFSI in DCE at 60 °C.

Kinetic Studies

$$aA + bB \longrightarrow cC + dD$$

$$\text{Rate} = -\frac{1}{a}\frac{d[A]}{dt} = -\frac{1}{b}\frac{d[B]}{dt} = \frac{1}{c}\frac{d[C]}{dt} = \frac{1}{d}\frac{d[D]}{dt} = k[A]^a[B]^b$$

$$\begin{aligned}\ln(\text{Rate}) &= \ln(k[A]^a[B]^b) \\ &= a\ln[A] + b\ln[B] + \ln(k)\end{aligned}$$

A mixture of diazoester, NFSI and 1-bromo-4-fluorobenzenein 3 mL DCE was stirred under argon. 100 μL of the mixture was sampled by a syringe and quenched by TFA (2 equivalent) at desired time. The concentration of **2a** is obtained by ^{19}F NMR Spectroscopy based on the internal standard (1-bromo-4-fluorobenzene). All reaction was monitored to 0~20 % yield of **2**. Initial rates were obtained by linear fit of the concentration-time plot (Figure S2 and Figure S3). A plot of ln(initial rate) vs. ln([**1a**]) or In([NFSI]) showed that the rate for the germinal aminofluorination of diazoesters is first-order in both **1a** and NFSI (Figure S4 and Figure S5, equation 1).

$$\text{Rate} = k[1a][NFSI] \qquad (1)$$

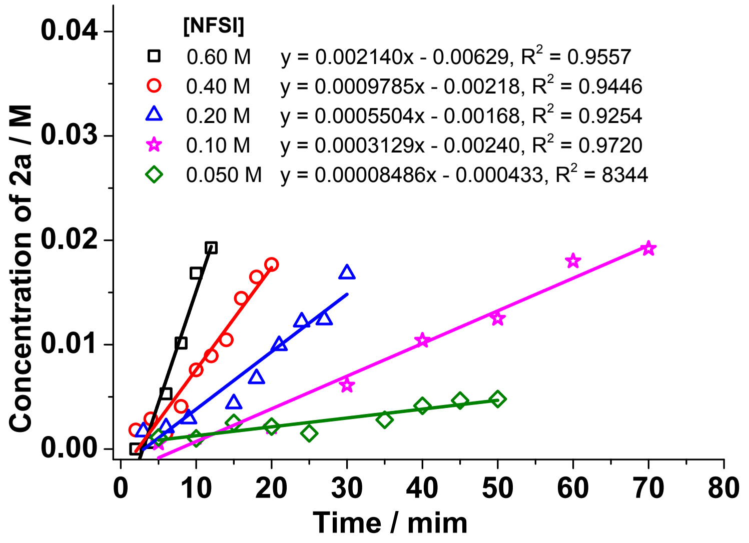

Figure S2. Plots of concentration of **2a** vs time for the reaction between diazoester **1a** and NFSI in DCE at 50 °C. [Diazo] = 0.15 M.

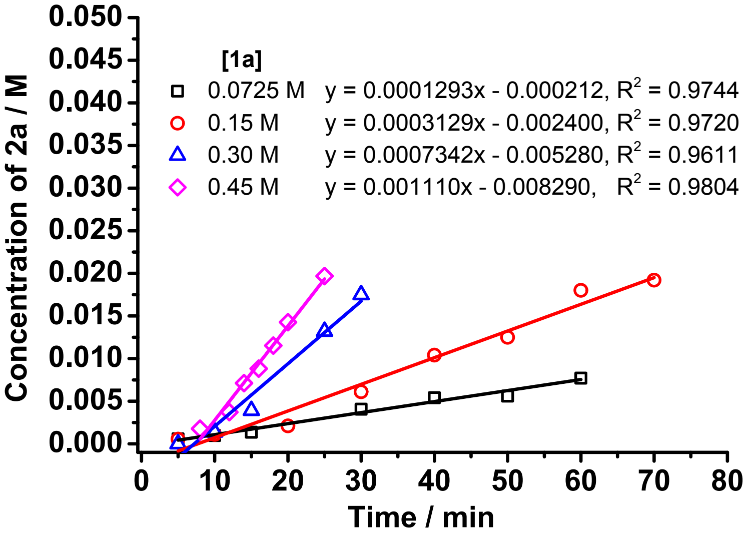

Figure S3. Plots of concentration of **2a** vs time for the reaction between diazoester **1a** and NFSI in DCE at 50 °C. [NFSI] = 0.10 M.

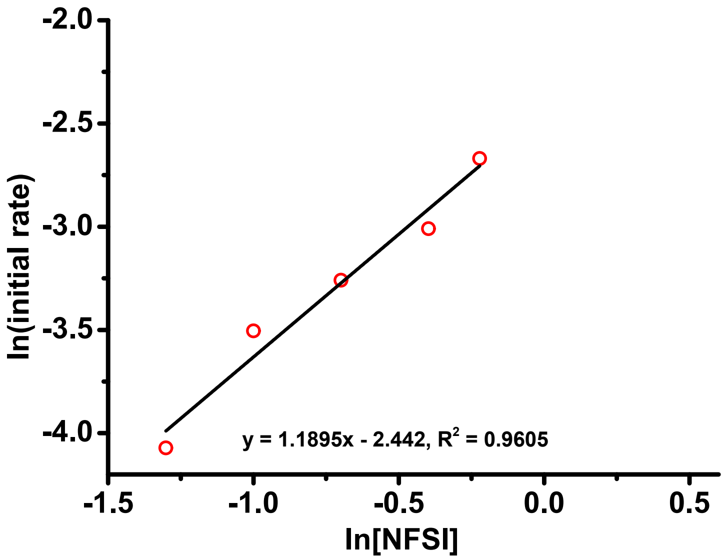

Figure S4. Plot of ln(initial rate) vs ln([NFSI]). y = 1.1895x – 2.442, R^2 = 0.9605. The slope of the line is approximately 1, indicating that the rate for the germinal aminofluorination of diazoester is first-order in NFSI.

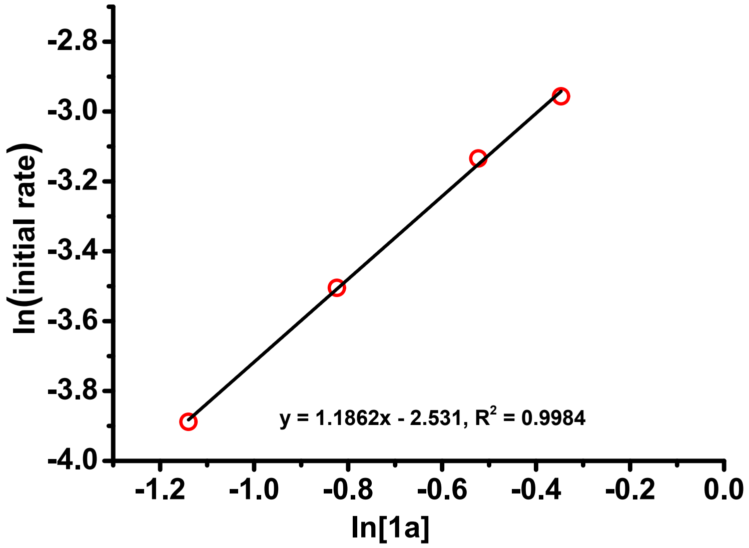

Figure S5. Plot of ln(initial rate) vs ln([**1a**]). y = 1.1862x – 2.531, R^2 = 0.9984. The slope of the line is approximately 1, indicating that the rate for the germinal aminofluorination of diazoester is first-order in **1a**.

Effect of temperature. The effect of temperature on initial rate for the reaction between diazoesters (0.15 M) and NFSI (0.10 M) in DCE were studied from 313 to 353 K (Figure S6). The activation parameters were obtained from the plot of ln(initial rate/T) vs 1/T according to Eyring equation. $\Delta H^{\ddagger}$ and $\Delta S^{\ddagger}$ were found to be 17.1 kcal mol^{-1} and −13.0 cal mol^{-1} K^{-1}, respectively. The negative $\Delta S^{\ddagger}$ value suggests that a bimolecular transition state involving NFSI and **1a** might be generated.

$$\text{Eyring equation: } \ln\frac{k}{T} = \frac{-\Delta H^{\ddagger}}{R}\frac{1}{T} + \ln\frac{k_B}{h} + \frac{\Delta S^{\ddagger}}{R}$$

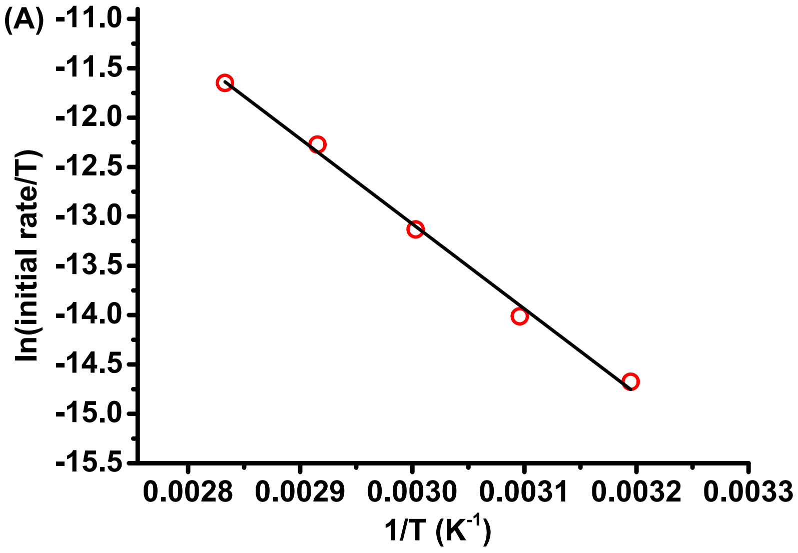

Figure S6.Plot of ln(initial rate/T) vs 1/T for the reaction between **1a** and NFSI in DCE, [**1a**] = 0.15 M, [NFSI] = 0.10 M, slope = -8.61 × 10^3, y-intercept = 1.27 × 10, r^2 = 0.996.

Hammett Correlation. The electronic effects of substituent X in the *para*-position of diazophenylacetates on the rate constants of this reactionhave been correlated by Hammett equation, $\log(k_X/k_H) = \rho\sigma^+$. The plot of $\log(k_X/k_H)$ against σ^+ shows a linear relationship (Figure S7), with the slope $\rho = -0.81$. The small negative ρ value suggests that the transition state is weakly polarized with a positive charge at the reaction center.

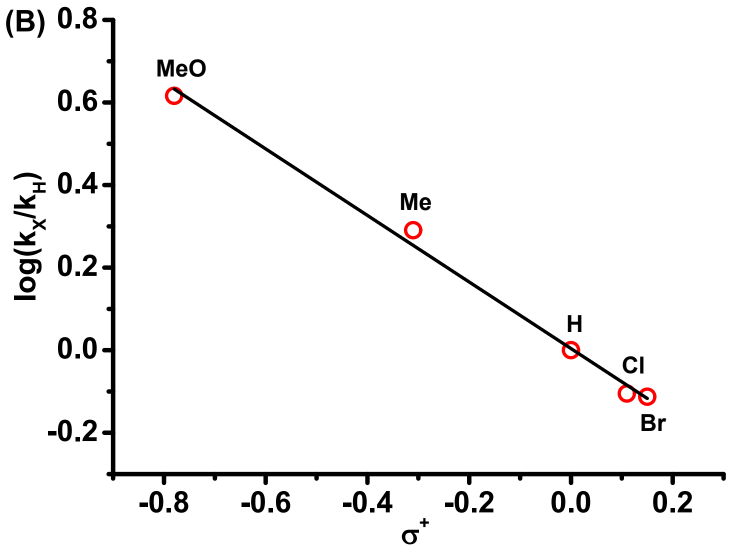

Figure S7. Hammett plot of $\log(k_X/k_H)$ vs σ^+ for the reaction of NFSI with *para*-substituted diazophenylacetate **1** in DCE at 50 °C, [**1**] = 0.15 M, [NFSI] = 0.10 M, slope = -0.81, y-intercept = 0.4×10^{-2}, $r^2 = 0.995$.

Computational method

All the calculations were carried out by using ORCA program package.[5] Full geometry optimization and frequency calculation were performed by using B3LYP functional[6,7] coupled with def2-SVP[8] basis set for all atoms. A larger basis set of def2-TZVPP4 was employed for single point energy corrections. To improve computational speed, the RIJCOSX approximation[9-11] in combination with def2-SVP/J and def2-TZVPP/J[12] auxiliary basis sets was applied. Dispersion effects were

computed by using the well-established dispersion corrections D3 with Becke-Johnson damping scheme.[13,14] Solvation effects were taken into account by the universal solvation model based on solute electron density (SMD)[15] with the conductor-like screening model (COSMO).[16]

Calculated potential energy surface

As shown in Scheme S1, four possible transformation pathways from reactant **1a** to product **2a** were calculated to explore the reaction mechanism. The calculated barriers of the first step in each pathway are collected in Table S1.

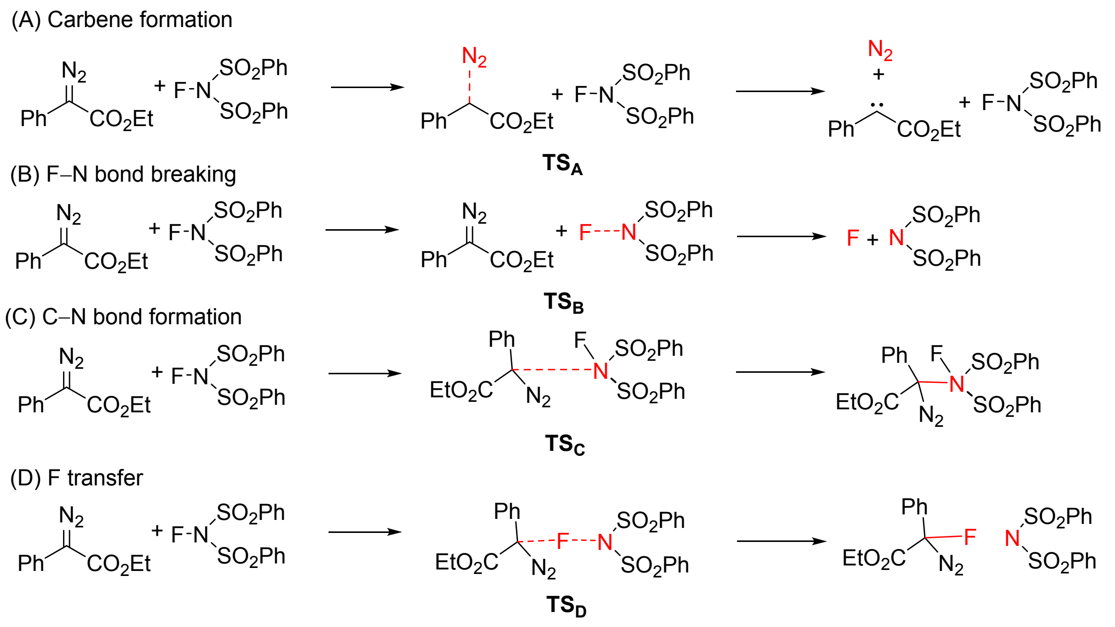

Scheme S1. The first step of the four possible transformation pathways from reactant **1a** to product **2a**. The major geometric differences were marked in red.

As can be seen from Table S1, pathway D is the lowest energy pathway where a fluorine transfer transition state (TS_D) is located with an enthalpy barrier of 16.2 kcal mol^{-1}, which is in good agreement with experimental value of 17.1 kcal mol^{-1}. The C−F and N−F bond lengths in TS_D are 1.772 Å and 1.920 Å, respectively (Figure S8). The following intermediate **Int**$_1$ after C−F bond formation and C−N bond breaking is

exothermic by 25.6 kcal mol^{-1}(Figure S9). Though the C-N bond has not been formed yet, these two atoms are quite close with a distance of 2.858 Å (Figure S8e), indicating the intermediate **Int**$_1$ is an ionic pair. All attempts to find other possible mechanisms excluding those shown in Scheme S1 failed as the obtained energy barriers were higher than 32 kcal mol^{-1} which were not possible in real experiment. The second step in pathway D was found to proceed in no barrier fashion with N_2 leaving. Thus, the corresponding intermediate **Int**$_2$ is exothermic by 12.8 kcal mol^{-1}. Like **Int**$_1$, this intermediate is also an ionic pair because the distance of C and N is 3.239 Å (Figure S8f). Finally, using the N−C distance as the scanning coordinate resulted in a downhill energy profile that fell down to the product. As a result, the total enthalpy change of the reaction is −84.8 kcal mol^{-1}.

Table S2. B3LYP calculated transition state energies in the first step of four possible pathways. The SCF energy, enthalpy and free energy changes are in kcal mol^{-1} while entropy changes are in cal mol^{-1} K^{-1}.

	$\Delta^{\ddagger}E$	$\Delta^{\ddagger}H$	$\Delta^{\ddagger}G$	$\Delta^{\ddagger}S$
A	33.6	32.0	29.0	10.1
B	52.7	51.6	50.9	2.5
C	58.2	54.5	57.9	−11.4
D	18.3	16.2	18.9	−9.0

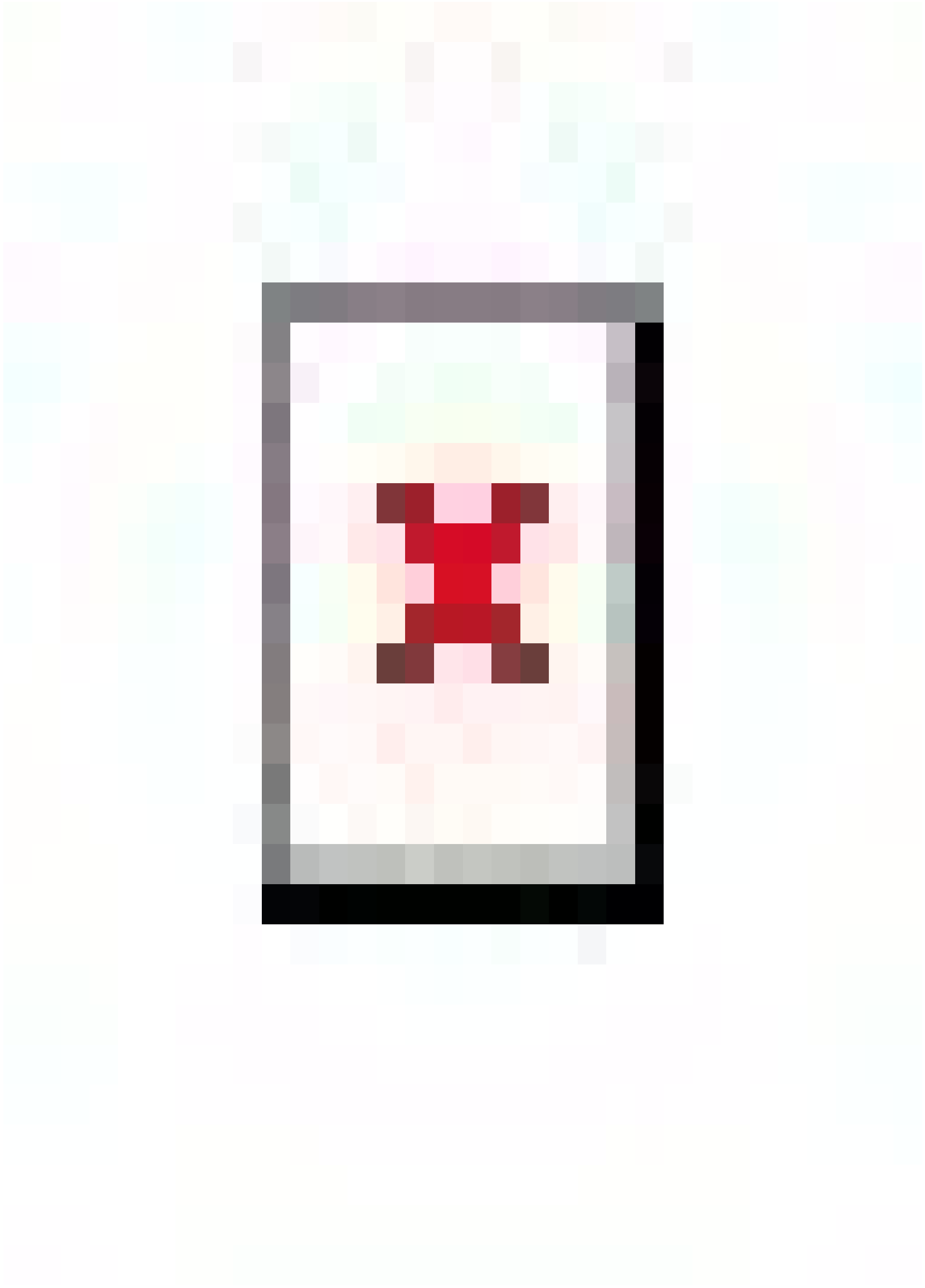

Figure S8. Geometries of four transition states in Scheme S1.

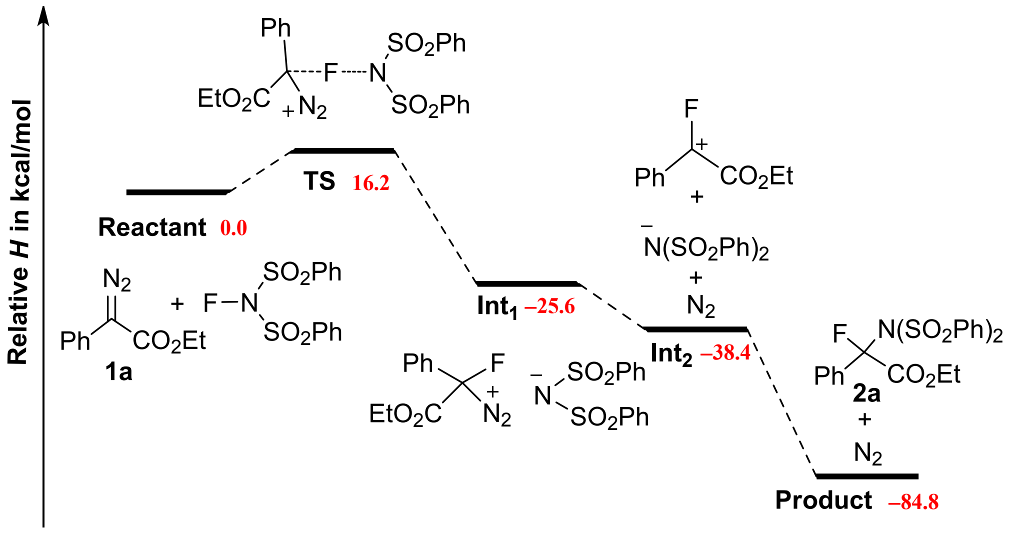

Figure S9. DFT computed enthalpy change (red text, in kcalmol^{-1}) for the reaction between **1a** and NFSI.

^{1}H and ^{13}C NMR Spectra datafor the prepared substrates

Ethyl 2-diazo-2-phenylacetate (1a)[1]

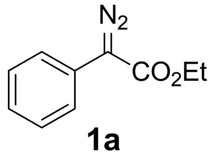

1a

^{1}H NMR (400 MHz, $CDCl_3$) δ 7.48 (d, *J* = 7.6 Hz, 2H), 7.38 (t, *J* = 7.6 Hz, 2H), 7.17 (t, *J* = 7.6 Hz, 1H), 4.34 (q, *J* = 7.1 Hz, 2H), 1.34 (t, *J* = 7.1 Hz, 3H); ^{13}C NMR (100 MHz, $CDCl_3$) δ 165.2, 128.9, 125.7, 125.6, 123.9, 60.9, 14.4.

Methyl 2-diazo-2-phenylacetate (1b)[1]

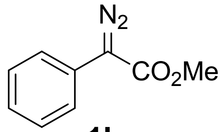

1b

^{1}H NMR (400 MHz, $CDCl_3$) δ 7.48 (d, *J* = 7.5 Hz, 2H), 7.38 (t, *J* = 7.5 Hz, 2H), 7.18 (t, *J* = 7.5 Hz, 1H), 3.86 (s, 3H); ^{13}C NMR (100 MHz, $CDCl_3$) δ 165.6, 128.9, 125.8, 125.4, 123.9, 51.9.

Isobutyl 2-diazo-2-phenylacetate (1c)[1]

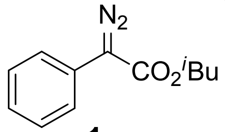

1c

^{1}H NMR (400 MHz, $CDCl_3$) δ 7.48 (d, *J* = 7.5 Hz, 2H), 7.38 (t, *J* = 7.5 Hz, 2H), 7.17 (t, *J* = 7.5 Hz, 1H), 4.06 (d, *J* = 6.6 Hz, 2H), 2.07–1.94 (m, 1H), 0.97 (d, *J* = 6.6 Hz, 6H); ^{13}C NMR (100 MHz, $CDCl_3$) δ 165.2, 128.9, 125.7, 125.6, 123.9, 70.9, 27.87, 19.0.

Benzyl 2-diazo-2-phenylacetate (1d)[1]

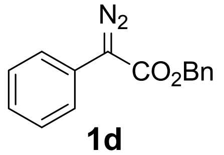

1d

^{1}H NMR (400 MHz, $CDCl_3$) δ 7.49 (d, *J* = 7.4 Hz, 2H), 7.32 – 7.41 (m, 7H), 7.18 (t, *J* = 7.4 Hz, 1H), 5.32 (s, 2H); ^{13}C NMR (100 MHz, $CDCl_3$) δ 165.0, 135.9, 128.9, 128.6, 128.3, 128.2, 125.9, 125.4, 124.0, 66.5.

(+)-Menthyl 2-diazo-2-phenylacetate (1e)[2]

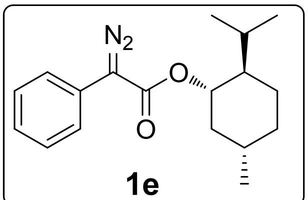

1e

^{1}H NMR (400 MHz, $CDCl_3$) δ 7.49 (d, *J* = 7.6 Hz, 2H), 7.36 (t, *J* = 7.6 Hz, 2H), 7.15 (t, *J* = 7.6 Hz, 1H), 4.91–4.85 (m,1H), 2.14–2.10 (m, 1H), 1.96–1.88 (m, 1H), 1.73–1.67 (m, 2H), 1.54–1.48 (m, 1H), 1.44 –1.41 (m, 1H), 1.15–1.02 (m, 2H), 0.93–0.90 (m, 6H), 0.88– 0.84 (m, 1H), 0.81 (d, *J* = 7.0 Hz, 3H); ^{13}C NMR (100 MHz, $CDCl_3$) δ 164.6, 128.8, 125.7, 125.5, 123.8, 74.9, 47.1, 41.3, 34.2, 31.4, 26.5, 23.6, 21.9, 20.6, 16.5.

4-Diazo-isochroman-3-one (1f)[1]

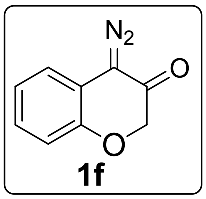

1f

^{1}H NMR (400 MHz, $CDCl_3$) δ 7.40–7.36 (m, 1H), 7.18–7.17 (m, 2H), 6.96 (d, *J* = 7.8 Hz, 1H),5.35 (s, 2H); ^{13}C NMR (100 MHz, $CDCl_3$) δ 166.8, 134.5, 125.4, 124.0, 118.2, 116.7, 108.6, 26.7.

3-Diazo-1-methyl-2-indolinone (1g)[3]

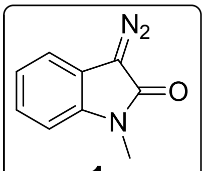

1g

^{1}H NMR (400 MHz, $CDCl_3$) δ 7.20(t, *J* = 7.6 Hz, 2H), 7.09 (t, *J* = 7.6 Hz, 1H), 6.92 (t, *J* = 7.6 Hz, 1H), 3.32 (s, 3H); ^{13}C NMR (100 MHz, $CDCl_3$) δ 166.8, 134.5, 125.4, 122.0, 118.2, 116.7, 108.6, 26.8.

Ethyl 2-diazo-2-(4-fluorophenyl)acetate (1h)[1]

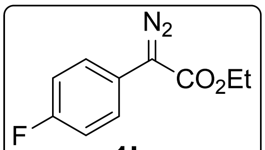

1h

^{1}H NMR (400 MHz, $CDCl_3$) δ 7.45–7.41 (m, 2H), 7.09–7.05 (m, 2H), 4.31 (q, *J* = 7.1 Hz, 2H), 1.33 (t, *J* = 7.1 Hz, 3H); ^{13}C NMR (100 MHz, $CDCl_3$) δ165.1, 160.8 (d, $J_{C\text{-}F}$ = 245 Hz), 125.7 (d,$J_{C\text{-}F}$ = 7.9 Hz), 121.3 (d, $J_{C\text{-}F}$= 3.2 Hz), 115.8 (d, $J_{C\text{-}F}$ = 21.8 Hz), 60.9, 14.3.

Ethyl 2-(4-chlorophenyl)-2-diazoacetate (1i)[1]

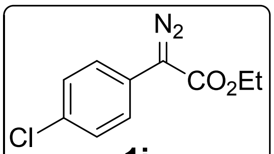

1i

^{1}H NMR (400 MHz, $CDCl_3$) δ 7.41 (d, *J* = 8.7 Hz, 2H),7.32 (d, *J* = 8.7 Hz, 2H), 4.32(q, *J* = 7.1 Hz, 2H), 1.33 (t, *J* = 7.1 Hz, 3H); ^{13}C NMR (100 MHz, $CDCl_3$) δ 164.8, 131.3, 129.0, 125.0, 124.2, 61.1, 14.4.

Ethyl 2-diazo-2-(3,4-dichlorophenyl)acetate (1j)[1]

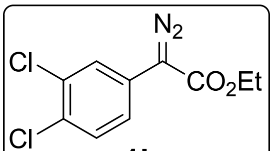

1j

^{1}H NMR (400 MHz, $CDCl_3$) δ 7.65 (d, *J* = 2.2 Hz, 1H), 7.42 (d, *J* = 8.5 Hz, 1H), 7.28 (dd, *J* = 8.5 Hz, *J* = 2.2 Hz, 1H), 4.34 (q, *J* = 7.1 Hz, 2H), 1.34 (t, *J* = 7.1 Hz, 3H); ^{13}C NMR (100 MHz, $CDCl_3$) δ 164.3, 133.1, 130.6, 129.2, 126.1, 125.1, 122.5, 61.3, 14.4.

Ethyl 2-(3-chlorophenyl)-2-diazoacetate (1k)[1]

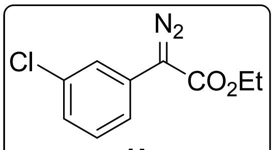

1k

^{1}H NMR (400 MHz, $CDCl_3$) δ 7.55 (s, 1H), 7.34–7.27 (m,2H), 7.14 (d, *J* = 7.4 Hz, 1H), 4.34 (q, *J* = 7.1 Hz, 2H), 1.34 (t, *J* = 7.1 Hz, 3H); ^{13}C NMR (100 MHz, $CDCl_3$) δ 164.6, 135.0, 130.0, 127.8, 125.6, 123.6, 121.5, 61.2, 14.4.

Ethyl 2-diazo-2-(3,5-dichlorophenyl)acetate (1l)[1]

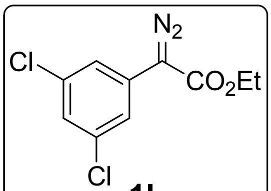

1l

^{1}H NMR (400 MHz, $CDCl_3$) δ 7.39 (d, *J* = 1.8 Hz, 2H), 7.13(t, *J* = 1.8 Hz, 1H), 4.34 (q, *J* = 7.1 Hz, 2H), 1.34 (t, *J* = 7.1 Hz, 3H); ^{13}C NMR (100 MHz, $CDCl_3$) δ 164.0, 135.5, 129.5, 125.4, 121.5, 61.4, 14.4.

Ethyl 2-(4-bromophenyl)-2-diazoacetate (1m)[1]

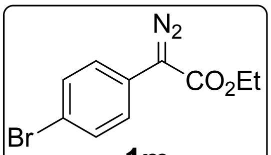

1m

^{1}H NMR (400 MHz, $CDCl_3$) δ 7.49 (d, *J* = 8.7 Hz, 2H), 7.36 (d, *J* = 8.7 Hz, 2H), 4.33 (q, *J* = 7.1 Hz, 2H), 1.34 (t, *J* = 7.1 Hz, 3H); ^{13}C NMR (100 MHz, $CDCl_3$) δ 164.8, 131.9, 125.3, 124.8, 119.2, 61.1, 14.4.

Ethyl 2-diazo-2-(4-(trifluoromethyl)phenyl)acetate (1n)[4]

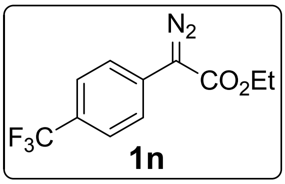

^{1}H NMR (400 MHz, $CDCl_3$) δ 7.61 (s, 4H), 4.35 (q, J = 7.1 Hz, 2H), 1.35 (t, J = 7.1 Hz, 3H); ^{13}C NMR (100 MHz, $CDCl_3$) δ 164.4, 130.2, 127.3 (q, $J_{C\text{-}F}$ =32.6 Hz), 125.7 (q, $J_{C\text{-}F}$ = 3.8 Hz), 124.0 (q, $J_{C\text{-}F}$ = 270 Hz), 123.3, 61.3, 14.4.

Ethyl 2-diazo-2-(3,4-dimethylphenyl)acetate (1o)[1]

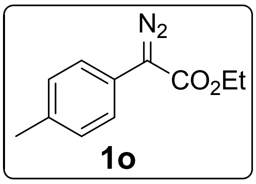

^{1}H NMR (400 MHz, $CDCl_3$) δ 7.36 (d, J = 8.2 Hz, 2H), 7.18 (d, J = 8.2 Hz, 2H), 4.31(q, J = 7.1 Hz, 2H), 2.33 (s, 3H), 1.33 (t, J = 7.1 Hz, 3H); ^{13}C NMR (100 MHz, $CDCl_3$) δ 165.4, 135.5, 129.6, 124.0, 122.2, 60.8, 20.9, 14.4.

Ethyl 2-diazo-2-(4-(tosyloxy)phenyl)acetate (1q)[1]

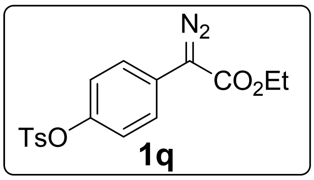

^{1}H NMR (400 MHz, $CDCl_3$) δ 7.70 (d, J = 7.6 Hz, 2H), 7.40 (d, J = 8.0 Hz, 2H), δ 7.31 (d, J = 7.6 Hz, 2H), 6.98 (d, J = 8.0 Hz, 2H), 4.32(q, J = 6.8 Hz, 2H), 2.45 (s, 3H), 1.33 (t, J = 6.8 Hz, 3H); ^{13}C NMR (100 MHz, $CDCl_3$) δ 164.8, 147.2, 145.4, 132.2, 129.8, 128.5, 124.9, 124.7, 122.9, 61.1, 21.7, 14.4.

Ethyl 2-(4-(benzyloxy)phenyl)-2-diazoacetate (1r)[1]

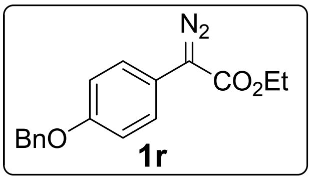

^{1}H NMR (400 MHz, $CDCl_3$) δ 7.42 (d, J = 7.0 Hz, 2H), 7.40–7.36 (m, 4H), 7.34–7.30 (m, 1H), 5.06 (s, 2H), 4.31(q, J = 7.1 Hz, 2H), 1.33 (t, J = 7.1 Hz, 3H); ^{13}C NMR (100 MHz, $CDCl_3$) δ 165.7, 157.1, 136.8, 128.6, 128.0, 127.4, 125.9, 117.4, 115.6, 70.1, 60.9, 14.5.

Ethyl 2-diazo-2-(4-methoxyphenyl)acetate (1s)[1]

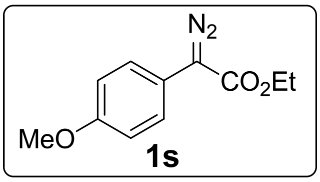

^{1}H NMR (400 MHz, $CDCl_3$) δ 7.37 (d, *J* = 8.9 Hz, 2H), 6.93 (d, *J* = 8.9 Hz, 2H), 4.30(q, *J* = 7.1 Hz, 2H), 3.78 (s, 3H),1.32 (t, *J* = 7.1 Hz, 3H); ^{13}C NMR (100 MHz, $CDCl_3$) δ 165.6, 157.9, 125.7, 116.9, 114.4, 60.8, 55.2, 14.4.

Ethyl 2-diazo-2-(4-methoxyphenyl)acetate (1t)[1]

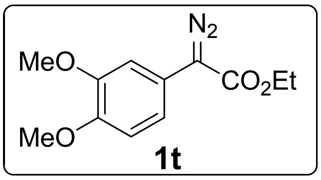

^{1}H NMR (400 MHz, $CDCl_3$) δ 7.20 (d, *J* = 1.5 Hz, 1H), 6.91–6.85 (m, 2H), 4.32(q, *J* = 7.1 Hz, 2H), 3.90 (s, 3H),3.88 (s, 3H),1.34 (t, *J* = 7.1 Hz, 3H); ^{13}C NMR (100 MHz, $CDCl_3$) δ 165.7, 149.4, 147.2, 117.5, 116.3, 111.6, 108.2, 60.9, 55.9, 55.8, 14.5.

Ethyl 2-diazo-2-(naphthalen-2-yl)acetate (1u)[1]

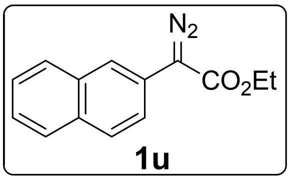

^{1}H NMR (400 MHz, $CDCl_3$) δ 8.01 (s,1H), 7.85 (d, *J* = 8.5 Hz, 1H), 7.80 (d, *J* = 7.3 Hz, 2H), 7.54 (d, *J* = 8.3 Hz, 1H), 7.50–7.42 (m, 2H), 4.38 (q, *J* = 6.8 Hz, 2H),1.38 (t, *J* = 6.8 Hz, 3H); ^{13}C NMR (100 MHz, $CDCl_3$) δ 165.3, 133.6, 131.4, 128.6, 127.6, 127.5, 126.6, 125.7, 122.8, 122.5, 121.9, 61.0, 14.5.

Vinyl 2-diazo-2-phenylacetate (1v)[1]

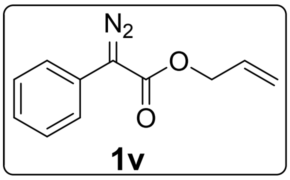

^{1}H NMR (400 MHz, $CDCl_3$)δ7.48 (d, *J* = 8.0 Hz, 2H), 7.38 (t, *J* = 8.0 Hz, 2H), 7.18 (t, *J* = 8.0 Hz, 1H), 6.02–5.93 (m, 1H), 5.40 (dd, *J* = 17.2Hz, *J* = 1.2Hz, 1H), 5.27 (d, *J* = 10.4Hz, 1H), 4.76 (t, J = 5.5 Hz, 2H); ^{13}C NMR (100 MHz, $CDCl_3$) δ 164.7, 132.0, 128.8, 125.7, 125.3, 123.9, 118.2, 65.3.

Vinyl 2-diazo-2-phenylacetate(1w)[4]

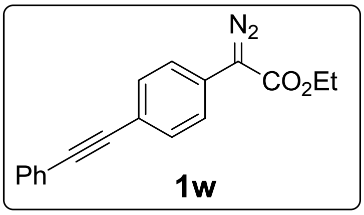

^{1}H NMR (400 MHz, $CDCl_3$) δ 7.54–7.52 (m, 4H), 7.47 -7.45 (m, 2H), 7.33-7.32 (m, 3H), 4.33 (q, *J* = 7.1 Hz, 2H), 1.34 (t, *J* = 7.1 Hz,3H); ^{13}C NMR (100 MHz, $CDCl_3$) δ 164.8, 132.1, 131.5, 128.3, 128.2, 125.7, 123.4, 123.2, 120.3, 89.6, 89.1, 61.1, 14.4.

^{1}H, ^{13}C and ^{19}F NMR Spectra data for the prepared products

Ethyl 2-fluoro-2-phenyl-2-(N-(phenylsulfonyl)phenylsulfonamido)acetate (2a)

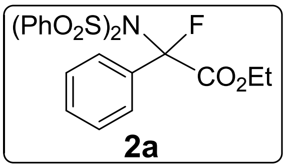

Compound **2a** was obtained as a white solid in 97% yield; R_f = 0.61 (petroleum ether : ethyl acetate = 3 : 1); ^{1}H NMR (400 MHz, $CDCl_3$) δ 7.85 (d, *J* = 7.5 Hz, 4H), 7.61 (t, *J* = 7.4 Hz, 2H), 7.46 (t, *J* = 7.5， 4H), 7.28–7.25 (m, 3H), 7.06 (t, *J* = 7.8, 2H), 4.38 – 4.22 (m, 2H), 1.30 (t, *J* = 7.2 Hz,3H); ^{13}C NMR (100 MHz, $CDCl_3$) δ 165.8 (d, $J_{C\text{-}F}$ = 29.8 Hz), 140.6, 133.9, 130.0, 129.9 (d, $J_{C\text{-}F}$ = 24.9 Hz), 128.7, 128.4 (d, $J_{C\text{-}F}$ = 9.8 Hz), 128.3, 127.4 (d, $J_{C\text{-}F}$ = 1.7 Hz), 100.3 (d, $J_{C\text{-}F}$ = 232.5 Hz), 63.4, 13.7; ^{19}F NMR (376 MHz, $CDCl_3$) δ -118.7; IR (KBr) 1773, 1762, 1450, 1378, 1358, 1261, 1190, 1172, 1067, 1045, 1005, 836, 790, 760, 747, 723, 716, 694, 685, 605, 593, 553 cm^{-1}; HRMS-(DART) (m/z): $(M + NH_4)^+$ calcd for $C_{22}H_{24}FN_2O_6S_2$, 495.1060; found 495.1043.

Methyl 2-fluoro-2-phenyl-2-(N-(phenylsulfonyl)phenylsulfonamido)acetate (2b)

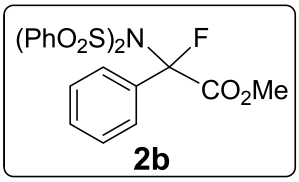

Compound **2a** was obtained as a white solid in 95% yield; R_f = 0.55 (petroleum ether : ethyl acetate = 3 : 1); ^{1}H NMR (400 MHz, $CDCl_3$) δ 7.84 (d, *J* = 7.8 Hz, 4H), 7.62 (t, *J* = 7.4 Hz, 2H), 7.47 (t, *J* = 7.8， 4H), 7.29– 7.25 (m, 3H), 7.06 (t, *J* = 7.9, 2H), 3.84(s,3H); ^{13}C NMR (100 MHz, $CDCl_3$) δ 166.6 (d, $J_{C\text{-}F}$ = 30.0 Hz), 140.5, 133.9, 130.0, 129.8 (d, $J_{C\text{-}F}$ = 25.0 Hz), 128.8, 128.4 (d, $J_{C\text{-}F}$ = 10.1 Hz), 128.2, 127.5 (d, $J_{C\text{-}F}$ = 1.7 Hz), 100.4 (d, $J_{C\text{-}F}$ = 233.1 Hz), 53.9; ^{19}F NMR (376 MHz, $CDCl_3$)δ -118.9; IR (KBr) 1772, 1753, 1450, 1387, 1360, 1279, 1208, 1194, 1194, 1087, 1065, 1045, 1034, 1007, 829, 822, 780, 752, 722, 682, 636, 606, 584, 561, 533 cm^{-1}; HRMS-(DART) (m/z): $(M + NH_4)^+$ calcd for $C_{21}H_{22}FN_2O_6S_2$, 481.0903; found 481.0893.

Isobutyl2-fluoro-2-phenyl-2-(N-(phenylsulfonyl)phenylsulfonamido)acetate (2c)

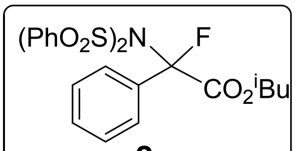

2c

Compound **2c** was obtained as a white solid in 95% yield; R_f = 0.39 (petroleum ether : ethyl acetate = 5 : 1); ^1H NMR (400 MHz, $CDCl_3$) δ 7.85 (d, *J* = 8.0 Hz, 4H), 7.61 (t, *J* = 7.4 Hz, 2H), 7.46 (t, *J* = 7.9, 4H), 7.27– 7.24 (m, 3H), 7.05 (t, *J* = 7.8, 2H), 4.00 (d, *J* = 6.6 Hz, 2H), 2.04–1.93 (m, 1H), 0.882 (d, *J* = 6.6 Hz, 3H), 0.876 (d, *J* = 6.8 Hz, 3H); ^{13}C NMR (100 MHz, $CDCl_3$) δ 165.9 (d, $J_{C\text{-}F}$ = 30.0 Hz), 140.6, 133.9, 130.1 (d, $J_{C\text{-}F}$ = 25.1 Hz), 129.9, 128.7, 128.33, 128.36 (d, $J_{C\text{-}F}$ = 9.6 Hz), 127.4 (d, $J_{C\text{-}F}$ = 1.6 Hz), 100.4 (d, $J_{C\text{-}F}$ = 232.5 Hz), 73.2, 27.4, 18.9; ^{19}F NMR (376 MHz, $CDCl_3$) δ -118.8; IR (KBr) 1768, 1452, 1381, 1357, 1262, 1246, 1208, 1190, 1174, 1162, 1083, 1066, 1042, 1007, 818, 777, 758, 748, 717, 684, 606, 591, 546; HRMS-(DART) (m/z): (M + $NH_4)^+$ calcd for $C_{24}H_{28}FN_2O_6S_2$, 523.1373; found 523.1360.

Benzyl2-fluoro-2-phenyl-2-(N-(phenylsulfonyl)phenylsulfonamido)acetate (2d)

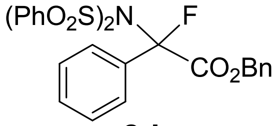

2d

Compound **2d** was obtained as a white solid in 94% yield; R_f = 0.63 (petroleum ether : ethyl acetate = 3 : 1); ^1H NMR (400 MHz, $CDCl_3$)δ 7.81 (d, *J* = 7.9 Hz, 4H), 7.58 (t, *J* = 7.4 Hz, 2H), 7.39 (t, *J* = 7.8, 4H), 7.33 – 7.31 (m, 5H), 7.25– 7.19 (m, 3H), 7.01 (t, *J* = 7.7, 2H), 5.28 (d, *J* = 12.1 Hz, 1H), 5.23 (d, *J* = 12.1 Hz, 1H); ^{13}C NMR (100 MHz, $CDCl_3$) δ 165.5 (d, $J_{C\text{-}F}$ = 30.7 Hz), 140.4, 134.3, 133.9, 130.0, 129.7 (d, $J_{C\text{-}F}$ = 24.9 Hz), 128.7, 128.48, 128.47 (d, $J_{C\text{-}F}$ = 8.0 Hz), 128.46, 128.3, 127.4 (d, $J_{C\text{-}F}$ = 1.5 Hz), 100.3 (d, $J_{C\text{-}F}$ = 231.8 Hz), 68.9; ^{19}F NMR (376 MHz, $CDCl_3$) δ -118.4; IR (KBr) 1771, 1751, 1450, 1381, 1363, 1267, 1190, 1174, 1080, 1068, 1044, 1004, 816, 772, 753, 723, 695, 680, 595, 555 cm^{-1}; HRMS-(DART) (m/z): (M + $NH_4)^+$ calcd for $C_{27}H_{26}FN_2O_6S_2$, 557.1216; found 557.1204.

(+)-Menthyl 2-fluoro-2-phenyl-2-(N-(phenylsulfonyl)phenylsulfonamido)acetate (2e)

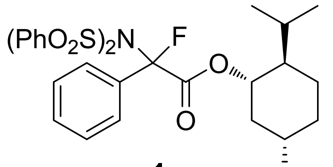

1e

Compound **2e** was obtained as a white solid in 92% yield; R_f = 0.57 (petroleum ether : ethyl acetate = 5 : 1); ^{1}H NMR (400 MHz, $CDCl_3$)δ 7.88 – 7.84 (m, 8H), 7.61– 7.58 (m, 4H), 7.47 – 7.43 (m，8H), 7.27 – 7.23 (m, 6H), 7.08 – 7.02 (m, 4H), 4.79– 4.66 (m, 2H), 2.18(d, *J* = 12.2 Hz,1H), 2.03 – 1.99 (m, 1H), 1.80 (d, *J* = 11.9 Hz, 1H), 1.64 – 1.54 (m, 4H), 1.46 – 1.36 (m, 3H), 1.34 – 1.26 (m, 2H), 1.15 – 1.04 (m, 3H), 1.01 – 0.91 (m, 1), 0.89 – 0.83 (m, 8H), 0.79 (d, *J* = 6.6 Hz, 3H), 0.74 (d, *J* = 6.9 Hz, 3H), 0.53 (d, *J* = 7.0 Hz, 3H), 0.29 (d, *J* = 6.9 Hz, 3H); ^{13}C NMR (100 MHz, $CDCl_3$) δ 165.8 (d, J_{C-F} = 27.9 Hz), 165.6 (d, J_{C-F} = 28.8 Hz), 140.6, 140.5, 133.83, 133.77, 130.1 (d, J_{C-F} = 11.9 Hz), 129.9, 129.85 (d, J_{C-F} = 11.7 Hz), 129.83, 128.65, 128.62, 128.5, 128.4, 128.36, 127.2 (d, J_{C-F} = 1.1 Hz), 127.1 (d, J_{C-F} = 1.4 Hz), 100.7 (d, J_{C-F} = 233.5 Hz), 100.5 (d, J_{C-F} = 235.4 Hz), 78.1, 77.7, 46.8, 46.7, 39.6, 39.2, 34.0, 33.9, 31.4, 31.2, 25.51, 25.55, 23.1, 23.0, 21.85, 21.81, 20.7, 20.1, 15.74, 15.70; ^{19}F NMR (376 MHz, $CDCl_3$) δ -118.4, -119.4; IR (KBr) 1742, 1450, 1380, 1289, 1269, 1174, 1080, 1045, 1006, 830, 786, 756, 719, 684, 596, 551 cm^{-1}; HRMS-(DART) (m/z): $(M + NH_4)^+$ calcd for $C_{30}H_{38}FN_2O_6S_2$, 605.2155; found 605.2143.

N-(4-fluoro-3-oxoisochroman-4-yl)-N-(phenylsulfonyl)benzenesulfonamide (2f)

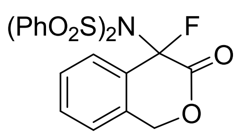

2f

Compound **2f**was obtained as a white solid in 97% yield; R_f = 0.29 (petroleum ether : ethyl acetate = 3 : 1); ^{1}H NMR (400 MHz, $CDCl_3$) δ 7.95 (d, *J* = 7.8 Hz, 1H), 7.79 (d, *J* = 6.2 Hz, 4H), 7.61 (t, *J* = 7.4, 2H), 7.52 (t, *J* = 7.7 Hz, 1H), 7.44 (t, *J* = 7.7 Hz, 4H), 7.38 (t, *J* = 7.5 Hz, 1H), 6.82 (d, *J* = 7.5 Hz, 1H), 5.10 (d, *J* = 13.9 Hz, 1H), 4.89 (d, *J* = 13.9 Hz, 1H); ^{13}C NMR (100 MHz, $CDCl_3$) δ 163.1 (d, J_{C-F} = 26.4 Hz), 139.5, 134.2, 133.9 (d, J_{C-F} = 5.5 Hz), 130.7, 129.9 (d, J_{C-F} = 4.8 Hz), 129.2 (d, J_{C-F} = 25.5 Hz), 128.8, 128.6, 128.2, 124.6, 95.9 (d, J_{C-F} = 232.9 Hz), 68.6; ^{19}F NMR (376 MHz, $CDCl_3$) δ -120.5;

IR (KBr) 1771, 1455, 1449, 1387, 1363, 1240, 1191, 1175, 1130, 1086, 1072, 1004, 989, 792, 758, 722, 702, 681, 670, 612, 592, 581, 547 cm^{-1}; HRMS-(DART) (m/z): $(M + NH_4)^+$ calcd for $C_{21}H_{20}FN_2O_6S_2$, 479.0747; found 479.0731.

N-(3-fluoro-1-methyl-2-oxoindolin-3-yl)-N-(phenylsulfonyl)benzenesulfonamide (2g)

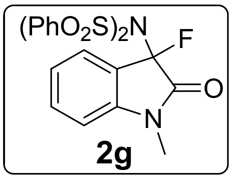

Compound **2g** was obtained as a white solid in 83% yield; R_f = 0.36 (petroleum ether : ethyl acetate = 3 : 1); ^{1}H NMR (400 MHz, $CDCl_3$) δ 7.85 – 7.80 (m, 5H), 7.64 (t, J = 7.4 Hz, 2H), 7.50 – 7.46 (m, 5H), 7.16 (t, J = 7.6, 1H), 6.89 (t, J = 7.8 Hz, 1H), 3.20 (s, 3H); ^{13}C NMR (100 MHz, $CDCl_3$) δ 167.9 (d, $J_{C\text{-}F}$ = 21.9 Hz), 144.6 (d, $J_{C\text{-}F}$ = 5.8 Hz), 139.9, 134.1, 133.2 (d, $J_{C\text{-}F}$ = 3.5 Hz), 128.7, 126.4, 123.6 (d, $J_{C\text{-}F}$ = 3.1 Hz), 123.0,122.8, 109.3 (d, $J_{C\text{-}F}$ = 1.2 Hz), 98.2 (d, $J_{C\text{-}F}$ = 208.6 Hz), 26.6; ^{19}F NMR (376 MHz, $CDCl_3$) δ -129.4; IR (KBr) 1751, 1617, 1473, 1450, 1382, 1362, 1180, 1169, 1114, 1084, 1064, 1028, 885, 762, 753, 719, 687, 610, 581 cm^{-1}; HRMS-(DART) (m/z): $(M + NH_4)^+$ calcd for $C_{21}H_{21}FN_3O_5S_2$, 478.0907; found 478.0897.

Ethyl 2-fluoro-2-(4-fluorophenyl)-2-(N-(phenylsulfonyl)phenylsulfonamido)acetate (2h)

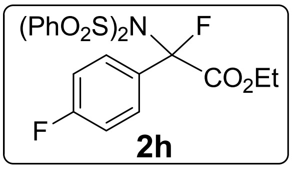

Compound **2h** was obtained as a white solid in 96% yield; R_f = 0.44 (petroleum ether : ethyl acetate = 5 : 1); ^{1}H NMR (400 MHz, $CDCl_3$) δ 7.89 (d, J = 7.6 Hz, 4H), 7.63 (t, J = 7.4 Hz, 2H), 7.49 (t, J = 7.6 Hz, 4H), 7.27–7.23 (m, 2H), 6.74 (t, J = 8.6 Hz, 2H), 4.39–4.23 (m, 2H), 1.30 (t, J = 7.1 Hz, 3H); ^{13}C NMR (100 MHz, $CDCl_3$) δ 165.8 (d, $J_{C\text{-}F}$ = 29.4 Hz), 163.6 (d, $J_{C\text{-}F}$ = 250 Hz), 140.4, 134.0, 130.8 (t, $J_{C\text{-}F}$ = 9.4 Hz), 128.8, 128.3, 125.7 (dd, $J_{C\text{-}F}$ = 25.4 Hz, $J_{C\text{-}F}$ = 3.2 Hz), 114.5 (dd, $J_{C\text{-}F}$ = 21.7 Hz, $J_{C\text{-}F}$ = 1.2 Hz), 100.0 (d, $J_{C\text{-}F}$ = 233.0 Hz), 63.6, 13.7; ^{19}F NMR (376 MHz, $CDCl_3$) δ -110.0, 117.9; IR (KBr) 1746, 1603, 1509, 1449, 1381, 1357, 1303, 1270, 1239, 1184, 1176, 1168, 1074, 1045, 998, 903, 852, 812, 753, 722, 683, 619, 600, 575, 550 cm^{-1}; HRMS-(DART) (m/z): $(M + NH_4)^+$ calcd for $C_{22}H_{23}F_2N_2O_6S_2$, 513.0966; found 513.0954.

Ethyl 2-(4-chlorophenyl)-2-fluoro-2-(N-(phenylsulfonyl)phenylsulfonamido)acetate (2i)

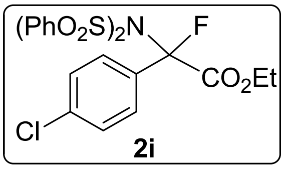

Compound **2i** was obtained as a white solid in 98% yield; R_f = 0.43 (petroleum ether : ethyl acetate = 5 : 1); ^{1}H NMR (400 MHz, $CDCl_3$) δ 7.86 (d, *J* = 7.4 Hz, 4H), 7.64 (t, *J* = 7.4 Hz, 2H), 7.49 (t, *J* = 7.5 Hz，4H), 7.20 (d, *J* = 8.8, 2H), 7.02 (d, *J* = 8.6 Hz, 2H), 4.39–4.22 (m, 2H), 1.30 (t, *J* = 7.2 Hz,3H); ^{13}C NMR (100 MHz, $CDCl_3$) δ 165.6 (d, $J_{C\text{-}F}$ = 29.6 Hz), 140.4, 136.5, 134.1, 129.9 (d, $J_{C\text{-}F}$ = 9.8 Hz), 128.8, 128.5 (d, $J_{C\text{-}F}$ = 25.7 Hz), 128.3, 127.6 (d, $J_{C\text{-}F}$ = 1.2 Hz), 99.9 (d, $J_{C\text{-}F}$ = 233.8 Hz), 63.6, 13.7; ^{19}F NMR (376 MHz, $CDCl_3$) δ -118.9; IR (KBr) 1748, 1491, 1451, 1381, 1385, 1300, 1266, 1182, 1175, 1162, 1096, 1075, 1048, 997, 907, 849, 813, 798, 754, 721, 685, 616, 608, 597, 560, 548 cm^{-1}; HRMS-(DART) (m/z): $(M + NH_4)^+$ calcd for $C_{22}H_{23}ClFN_2O_6S_2$, 529.0670; found 529.0658.

Ethyl2-(3,4-dichlorophenyl)-2-fluoro-2-(N-(phenylsulfonyl)phenylsulfonamido)acetate(2j)

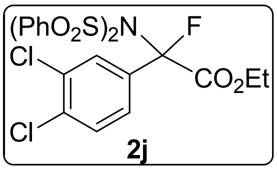

Compound **2j** was obtained as a white solid in 94% yield; R_f = 0.43 (petroleum ether : ethyl acetate = 5 : 1); ^{1}H NMR (400 MHz, $CDCl_3$) δ 7.87 (d, *J* = 7.7 Hz, 4H), 7.66 (t, *J* = 7.4 Hz, 2H), 7.52 (t, *J* = 7.6 Hz，4H), 7.30–7.23 (m, 2H), 7.16 (d, *J* = 1.8 Hz, 1H), 4.41–4.22 (m, 2H), 1.31(t, *J* = 7.2 Hz,3H); ^{13}C NMR (100 MHz, $CDCl_3$) δ 165.2 (d, $J_{C\text{-}F}$ = 29.6 Hz), 140.1, 134.9, 134.3, 131.8 (d, $J_{C\text{-}F}$ = 1.9 Hz), 130.21 (d, $J_{C\text{-}F}$ = 10.0 Hz), 130.20 (d, $J_{C\text{-}F}$ = 26.0 Hz), 129.4, 128.9, 128.2, 128.1 ($J_{C\text{-}F}$ = 10.2 Hz), 99.2 (d, $J_{C\text{-}F}$ = 234.8 Hz), 63.8, 13.6; ^{19}F NMR (376 MHz, $CDCl_3$) δ -118.5; IR (KBr) 1740, 1466, 1449, 1388, 1369, 1360, 1296, 1263, 1212, 1190, 1176, 1083, 1056, 1033, 1015, 909, 851, 827, 789, 760, 751, 724, 682, 616, 609, 579 cm^{-1}; HRMS-(DART) (m/z): $(M + NH_4)^+$ calcd for $C_{22}H_{22}Cl2FN_2O_6S_2$, 563.0280; found 563.0269.

Ethyl 2-(4-chlorophenyl)-2-fluoro-2-(N-

(phenylsulfonyl)phenylsulfonamido)acetate (**2k**)

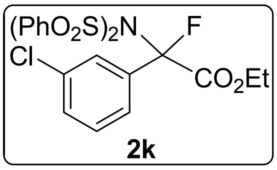

Compound **2k** was obtained as a white solid in 85% yield; R_f = 0.38 (petroleum ether : ethyl acetate = 5 : 1); ^{1}H NMR (400 MHz, $CDCl_3$) δ 7.90 (d, *J* = 7.5 Hz, 4H), 7.64 (t, *J* = 7.4 Hz, 2H), 7.50 (t, *J* = 7.6 Hz，4H), 7.33 (d, *J* = 7.9, 1H), 7.26 (d, *J* = 5.9 Hz, 1H), 7.10 (t, *J* = 8.0 Hz, 1H), 7.05 (s, 1H), 4.40–4.22 (m, 2H),1.30 (t, *J* = 7.1 Hz,3H); ^{13}C NMR (100 MHz, $CDCl_3$) δ 165.4 (d, $J_{C\text{-}F}$ = 29.9 Hz), 140.2, 134.2, 133.4 (d, $J_{C\text{-}F}$ = 2.2 Hz), 132.0 (d, $J_{C\text{-}F}$ = 25.6 Hz), 130.3, 128.8, 128.7 (d, $J_{C\text{-}F}$ = 6.1 Hz), 128.4 (d, $J_{C\text{-}F}$ = 10.1 Hz), 128.2, 126.9 (d, $J_{C\text{-}F}$ = 10.2 Hz), 99.5 (d, $J_{C\text{-}F}$ = 233.9 Hz), 63.6, 13.6; ^{19}F NMR (376 MHz, $CDCl_3$) δ -118.4; IR (KBr) 1773, 1749, 1577, 1477, 1452, 1417, 1389, 1362, 1281, 1267, 1206, 1193, 1168, 1108, 1087, 1066, 1048, 1023, 998, 904, 835, 776, 753, 721, 683, 639, 606 cm^{-1}; HRMS-(DART) (m/z): $(M + NH_4)^+$ calcd for $C_{22}H_{23}ClFN_2O_6S_2$, 529.0670; found 529.0664.

Ethyl2-(3,5-dichlorophenyl)-2-fluoro-2-(N-(phenylsulfonyl)phenylsulfonamido)acetate (**2l**)

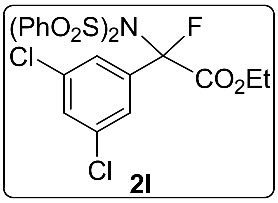

Compound **2l** was obtained as a white solid in 52% yield; R_f = 0.52 (petroleum ether : ethyl acetate = 5 : 1); ^{1}H NMR (400 MHz, $CDCl_3$) δ 7.91 (d, *J* = 7.8 Hz, 4H), 7.66 (t, *J* = 7.4 Hz, 2H), 7.53 (t, *J* = 7.8 Hz，4H), 7.30 (s, 1H), 7.13 (s, 2H), 4.41 – 4.22 (m, 2H),1.30(t, *J* = 7.2 Hz,3H); ^{13}C NMR (100 MHz, $CDCl_3$) δ 165.1 (d, $J_{C\text{-}F}$ = 30.0 Hz), 140.2, 134.4, 134.3 (d, $J_{C\text{-}F}$ = 2.0 Hz), 133.6 (d, $J_{C\text{-}F}$ = 26.2 Hz), 130.4, 128.9, 128.3, 127.0 ($J_{C\text{-}F}$ = 10.4 Hz), 99.0 (d, $J_{C\text{-}F}$ = 235.1 Hz), 63.9, 13.6; ^{19}F NMR (376 MHz, $CDCl_3$) δ -118.0.IR (KBr) 1770, 1471, 1450, 1382, 1358, 1298, 1261, 1245, 1208, 1190, 1175, 1163, 1083, 1065, 1041, 1006, 929, 898, 817, 777, 762, 758, 746, 716, 683, 633, 614, 606, 590, 560, 541 cm^{-1}; HRMS-(DART) (m/z): $(M + NH_4)^+$ calcd for $C_{22}H_{22}Cl_2FN_2O_6S_2$, 563.0280; found 563.0267.

Ethyl 2-(4-bromophenyl)-2-fluoro-2-(N-(phenylsulfonyl)phenylsulfonamido)acetate (**2m**)

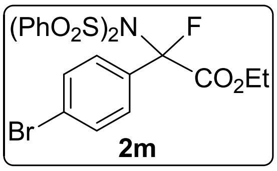

2m

Compound **2m** was obtained as a white solid in 97% yield; R_f = 0.44 (petroleum ether : ethyl acetate = 5 : 1); ^{1}H NMR (400 MHz, $CDCl_3$) δ 7.85 (d, *J* = 7.6 Hz, 4H), 7.64 (t, *J* = 7.4 Hz, 2H), 7.49 (t, *J* = 7.7 Hz，4H), 7.18 (d, *J* = 8.7, 2H), 7.13 (d, *J* = 8.8 Hz, 2H), 4.39–4.22 (m, 2H), 1.30 (t, *J* = 7.1 Hz,3H); ^{13}C NMR (100 MHz, $CDCl_3$) δ 165.5 (d, $J_{C\text{-}F}$ = 29.3 Hz), 140.4, 134.1, 130.6 (d, $J_{C\text{-}F}$ = 1.3 Hz), 130.0 (d, $J_{C\text{-}F}$ = 9.8 Hz), 129.1 (d, $J_{C\text{-}F}$ = 25.6 Hz), 128.8, 128.3, 124.9, 99.9 (d, $J_{C\text{-}F}$ = 234.0 Hz), 63.7, 13.7; ^{19}F NMR (376 MHz, $CDCl_3$) δ -119.1; IR (KBr) 1748, 1589, 1488, 1450, 1403, 1378, 1355, 1296, 1266, 1184, 1173, 1162, 1078, 1048, 996, 905, 847, 810, 754, 742, 720, 701, 683, 614, 605, 597, 559, 543 cm^{-1}; HRMS-(DART) (m/z): $(M + NH_4)^+$ calcd for $C_{22}H_{23}BrFN_2O_6S_2$, 573.0165; found 573.0160.

Ethyl 2-fluoro-2-(N-(phenylsulfonyl)phenylsulfonamido)-2-(4-(trifluoromethyl)phenyl)acetate (**2n**)

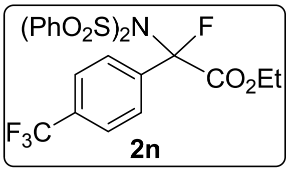

2n

Compound **2n** was obtained as a white solid in 55% yield; R_f = 0.45 (petroleum ether : ethyl acetate = 5 : 1); ^{1}H NMR (400 MHz, $CDCl_3$) δ 7.84 (d, *J* = 7.6 Hz, 4H), 7.64 (t, *J* = 7.5 Hz, 2H), 7.48 (t, *J* = 7.8 Hz，4H), 7.42 (d, *J* = 8.2, 2H), 7.30 (d, *J* = 8.4 Hz, 2H), 4.40 – 4.22 (m, 2H), 1.30 (t, *J* = 7.1 Hz,3H); ^{13}C NMR (100 MHz, $CDCl_3$) δ 165.3 (d, $J_{C\text{-}F}$ = 29.7 Hz), 140.4, 134.2, 134.1 (d, $J_{C\text{-}F}$ = 25.5 Hz), 131.9 (q, $J_{C\text{-}F}$ = 32.6 Hz), 128.87 (d, $J_{C\text{-}F}$ = 9.5 Hz), 128.86, 128.3, 123.5 (q, $J_{C\text{-}F}$ = 270.8 Hz), 124.2 (m), 99.7 (d, $J_{C\text{-}F}$ = 234.6 Hz), 63.8, 13.6; ^{19}F NMR (376 MHz, $CDCl_3$) δ -63.1, -119.3;IR (KBr) 1747, 1453, 1388, 1365, 1328, 1300, 1282, 1176, 1133, 1084, 1073, 1056, 1005, 998, 916, 856, 812, 763, 717, 687, 616, 605, 593, 559, 547 cm^{-1}; HRMS-(DART) (m/z): $(M + NH_4)^+$ calcd for $C_{23}H_{23}F_4N_2O_6S_2$, 563.0934; found 563.0924.

Ethyl 2-fluoro-2-(N-(phenylsulfonyl)phenylsulfonamido)-2-(p-tolyl)acetate (**2o**)

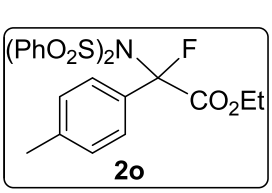

2o

Compound **2o** was obtained as a white solid in 95% yield; R_f =

0.36 (petroleum ether : ethyl acetate = 5 : 1); ^{1}H NMR (400 MHz, $CDCl_3$) δ 7.84 (d, *J* = 7.6 Hz, 4H), 7.61 (t, *J* = 7.4 Hz, 2H), 7.46 (t, *J* = 7.7 Hz， 4H), 7.13 (d, *J* = 8.2, 2H), 6.85 (d, *J* = 8.1 Hz, 2H), 4.38– 4.21 (m, 2H),2.29 (s, 3H), 1.30 (t, *J* = 7.1 Hz,3H); ^{13}C NMR (100 MHz, $CDCl_3$) δ165.9 (d, J_{C-F} = 29.5 Hz), 140.5, 140.2, 133.8, 128.6, 128.4 (d, J_{C-F} = 9.8 Hz), 128.3,128.1 (d, J_{C-F} = 1.6 Hz), 126.7 (d, J_{C-F} = 25.0 Hz), 100.4 (d, J_{C-F} = 232.3 Hz), 63.3, 21.1, 13.7; ^{19}F NMR (376 MHz, $CDCl_3$)δ -118.6; IR (KBr) 1747, 1450, 1377, 1356, 1315, 1298, 1271, 1183, 1174, 1162, 1107, 1050, 1028, 1014, 996, 906, 855, 845, 810, 792, 754, 722, 704, 685, 620, 612, 600, 576, 553, 532 cm^{-1}; HRMS-(DART) (m/z): $(M + NH_4)^+$ calcd for $C_{23}H_{26}FN_2O_6S_2$, 509.1216; found 509.1210.

Ethyl 2-fluoro-2-(N-(phenylsulfonyl)phenylsulfonamido)-2-(4-(tosyloxy)phenyl)acetate (**2q**)

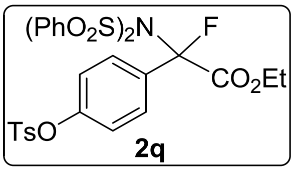

Compound **2q** was obtained as a white solid in 92% yield; R_f = 0.40 (petroleum ether : ethyl acetate = 3 : 1); ^{1}H NMR (400 MHz, $CDCl_3$) δ 7.83 (d, *J* = 7.8 Hz, 4H), 7.74 (d, *J* = 8.2 Hz, 2H), 7.64 (t, *J* = 7.4 Hz, 2H), 7.50 (t, *J* = 7.7 Hz， 4H), 7.35 (d, *J* = 8.1, 2H), 7.19 (d, *J* = 8.6 Hz, 2H), 6.72 (d, *J* = 8.8 Hz, 2H), 4.37–4.23 (m, 2H),2.45 (s, 3H), 1.29(t, *J* = 7.2Hz,3H); ^{13}C NMR (100 MHz, $CDCl_3$) δ 165.5 (d, J_{C-F} = 29.9 Hz), 150.7, 145.8, 140.3, 134.2, 132.2, 130.1 (d, J_{C-F} = 9.9 Hz), 129.9, 128.9, 128.5 (d, J_{C-F} = 21.2 Hz), 128.39, 128.35, 121.3(d, J_{C-F} = 1.1 Hz), 99.8 (d, J_{C-F} = 232.9 Hz), 63.6, 21.7, 13.7; ^{19}F NMR (376 MHz, $CDCl_3$) δ -118.0; IR (KBr) 1771, 1750, 1597, 1502, 1451, 1380, 1363, 1295, 1268, 1203, 1179, 1157, 1078, 1048, 1004, 997, 867, 818, 770, 754, 722, 684, 661, 621, 600, 572, 557, 542 cm^{-1}; HRMS-(DART) (m/z): $(M + NH_4)^+$ calcd for $C_{29}H_{30}FN_2O_9S_3$, 665.1097; found 665.1076.

Ethyl 2-(4-(benzyloxy)phenyl)-2-fluoro-2-(N-(phenylsulfonyl)phenylsulfonamido)acetate (**2r**)

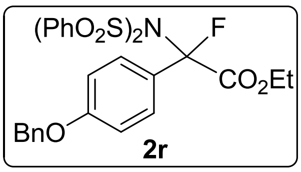

Compound **2r** was obtained as a white solid in 44% yield; R_f = 0.63 (petroleum ether : ethyl acetate = 3 : 1); ^{1}H NMR (400 MHz, $CDCl_3$) δ 7.82 (d, *J* = 7.7 Hz, 4H), 7.55 (t, *J* =

7.4 Hz, 2H), 7.41 – 7.34 (m，9H), 7.13 (d, J = 8.7, 2H), 6.61 (d, J = 8.6 Hz, 2H), 5.05 (d, J = 12.0 Hz, 1H), 5.00 (d, J = 12.0 Hz, 1H), 4.37– 4.21 (m, 2H), 1.29 (t, J = 7.1 Hz,3H); ^{13}C NMR (100 MHz, $CDCl_3$) δ 165.8(d, J_{C-F} = 29.4 Hz), 159.8, 140.5, 136.4, 133.8, 130.1 (d, J_{C-F} = 9.7 Hz), 128.63, 128.62, 128.5, 128.2, 127.4, 121.4 (d, J_{C-F} = 25.1 Hz), 113.7 (d, J_{C-F} = 1.0 Hz), 100.3 (d, J_{C-F} = 231.6 Hz), 69.8, 63.3, 13.7; ^{19}F NMR (376 MHz, $CDCl_3$) δ -117.5; IR (KBr) 1769, 1748, 1607, 1583, 1510, 1450, 1378, 1362, 1257, 1180, 1075, 1045, 993, 846, 820, 800, 754, 731, 722, 683, 606, 550 cm^{-1}; HRMS-(DART) (m/z): (M + NH_4)$^+$ calcd for $C_{29}H_{30}FN_2O_7S_2$, 601.1478; found 601.1467.

Ethyl 2-fluoro-2-(4-methoxyphenyl)-2-(N-(phenylsulfonyl)phenylsulfonamido)acetate (**2s**)

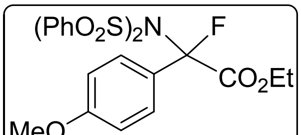

2s

Compound **2s** was obtained as a white solid in 43% yield; R_f = 0.20 (petroleum ether : ethyl acetate = 3 : 1); ^{1}H NMR (400 MHz, $CDCl_3$) δ 7.85 (d, J = 7.6 Hz, 4H), 7.61 (t, J = 7.5 Hz, 2H), 7.47 (t, J = 7.7 Hz, 4H), 7.15 (d, J = 8.8, 2H), 6.54 (d, J = 8.8 Hz, 2H), 4.39– 4.23 (m, 2H), 3.77 (s, 3H), 1.31 (t, J = 7.2Hz,3H); ^{13}C NMR (100 MHz, $CDCl_3$) δ 165.9 (d, J_{C-F} = 29.3 Hz), 160.8, 140.5, 133.8, 130.1 (d, J_{C-F} = 9.8 Hz), 128.7, 128.2, 121.2 (d, J_{C-F} = 25.0 Hz), 112.8 (d, J_{C-F} = 1.2 Hz), 100.4 (d, J_{C-F} = 231.7 Hz), 63.3, 55.3, 13.7; ^{19}F NMR (376 MHz, $CDCl_3$) δ -117.7; IR (KBr) 1768, 1746, 1609, 1585, 1513, 1449, 1374, 1262, 1170, 1086, 1045, 1018, 992, 849, 819, 753, 722, 684, 581, 547 cm^{-1}; HRMS-(DART) (m/z): (M + NH_4)$^+$ calcd for $C_{23}H_{26}FN_2O_7S_2$, 525.1165; found 525.1165.

Ethyl 2-(3,4-dimethoxyphenyl)-2-fluoro-2-(N-(phenylsulfonyl)phenylsulfonamido)acetate (**2t**)

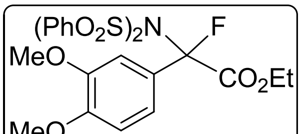

2t

Compound **2t** was obtained as a white solid in 40% yield; R_f = 0.21 (petroleum ether : ethyl acetate = 3 : 1); ^{1}H NMR (400 MHz, $CDCl_3$) δ 7.85 (d, J = 7.8 Hz, 4H), 7.61 (t, J = 7.4 Hz, 2H), 7.48 (t, J = 7.7 Hz, 4H), 6.86 (d, J = 7.5, 1H), 6.72 (s, 1H), 6.54 (d, J =

8.7, 1H), 4.40– 4.24 (m, 2H), 3.85 (s, 3H), 3.58 (s, 3H), 1.32 (t, J = 7.2 Hz,3H); ^{13}C NMR (100 MHz, $CDCl_3$) δ 165.8 (d, $J_{C\text{-}F}$ = 29.4 Hz), 150.3, 147.5, 140.6, 133.8, 128.7, 128.2, 122.1 (d, $J_{C\text{-}F}$ = 10.2 Hz), 121.3 (d, $J_{C\text{-}F}$ = 25.2 Hz), 111.4 (d, $J_{C\text{-}F}$ = 10.2 Hz), 109.6, 100.3 (d, $J_{C\text{-}F}$ = 232.0 Hz), 63.4, 55.9, 55.4, 13.7; ^{19}F NMR (376 MHz, $CDCl_3$) δ -117.1;IR (KBr) 1770, 1750, 1518, 1465, 1448, 1376, 1361, 1266, 1248, 1189, 1171, 1150, 1082, 1056, 1039, 1024, 882, 856, 827, 800, 758, 722, 684, 625, 592, 550 cm^{-1}; HRMS-(DART) (m/z): (M + NH_4)$^+$ calcd for $C_{24}H_{28}FN_2O_8S_2$, 555.1271; found 555.1258.

Ethyl 2-fluoro-2-(naphthalen-2-yl)-2-(N-(phenylsulfonyl)phenylsulfonamido)acetate (**2u**)

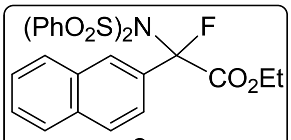

2u

Compound **2u** was obtained as a white solid in 90% yield; R_f = 0.39 (petroleum ether : ethyl acetate = 5 : 1); ^{1}H NMR (400 MHz, $CDCl_3$) δ 7.80 (d, J = 7.8 Hz, 4H), 7.77 (d, J = 10.8 Hz, 1H), 7.62–7.49 (m, 6H), 7.45–7.42 (m, 2H), 7.37 (d, J = 8.1 Hz, 4H), 4.41–4.21 (m, 2H), 1.28 (t, J = 7.2 Hz,3H); ^{13}C NMR (100 MHz, $CDCl_3$) δ 165.8 (d, $J_{C\text{-}F}$ = 29.7 Hz), 140.5, 133.9, 133.5, 131.6 (d, $J_{C\text{-}F}$ = 1.4 Hz), 128.870 (d, $J_{C\text{-}F}$ = 9.9 Hz), 128.872, 128.6, 128.3, 127.7, 127.3, 127.0, 126.9 (d, $J_{C\text{-}F}$ = 1.6 Hz), 126.3, 124.9 (d, $J_{C\text{-}F}$ = 9.6 Hz), 100.5 (d, $J_{C\text{-}F}$ = 233.0 Hz), 63.4, 13.7; ^{19}F NMR (376 MHz, $CDCl_3$) δ -118.2; IR (KBr) 1770, 1750, 1506, 1475, 1449, 1380, 1361, 1293, 1270, 1174, 1129, 1082, 1051, 1026, 943, 914, 860, 829, 810, 751, 722, 684, 630, 616, 611, 599, 580, 558, 543 cm^{-1}; HRMS-(DART) (m/z): (M + NH_4)$^+$ calcd for $C_{26}H_{26}FN_2O_6S_2$, 545.1216; found 545.1203.

Allyl 2-fluoro-2-phenyl-2-(N-(phenylsulfonyl)phenylsulfonamido)acetate (**2v**)

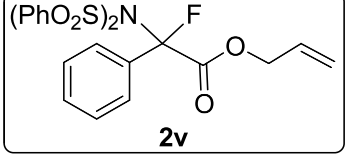

2v

Compound **2v** was obtained as a white solid in 96% yield; R_f = 0.38 (petroleum ether : ethyl acetate = 5 : 1); ^{1}H NMR (400 MHz, $CDCl_3$) δ 7.74 (d, J = 7.6 Hz, 4H), 7.50 (d, J = 7.5 Hz, 2H),7.35 (t, J = 7.8 Hz, 4H), 7.19– 7.15 (m, 3H), 6.95 (d, J = 8.0 Hz, 2H), 5.84–5.76 (m, 1H), 5.20– 5.10 (m, 2H), 4.68– 4.58 (m, 2H); ^{13}C NMR (100

MHz, $CDCl_3$) δ 165.5 (d, J_{C-F} = 30.1 Hz), 140.4, 133.9, 130.7, 130.0, 129.7 (d, J_{C-F} = 24.9 Hz), 128.7, 128.4 (d, J_{C-F} = 9.8 Hz), 128.2, 127.4 (d, J_{C-F} = 1.7 Hz), 119.3, 100.3 (d, J_{C-F} = 232.7 Hz), 67.6; ^{19}F NMR (376 MHz, $CDCl_3$) δ -118.8; IR (KBr) 1770, 1751, 1450, 1381, 1362, 1295, 1273, 1253, 1208, 1172, 1082, 1068, 1046, 1007, 931, 827, 777, 754, 721, 694, 683, 601, 553, 542 cm^{-1}; HRMS-(DART) (m/z): (M + NH_4)$^+$ calcd for $C_{23}H_{24}FN_2O_6S_2$, 507.1060; found 507.1049.

Ethyl 2-fluoro-2-(4-(phenylethynyl)phenyl)-2-(N-(phenylsulfonyl)phenylsulfonamido)acetate (**2w**)

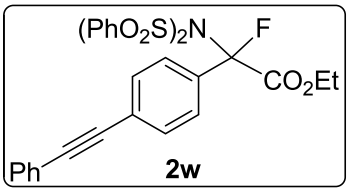

Compound **2w** was obtained as a white solid in 96% yield; R_f = 0.45 (petroleum ether : ethyl acetate = 5 : 1); ^{1}H NMR (400 MHz, $CDCl_3$) δ 7.79 (d, J = 7.5 Hz, 4H), 7.53 (t, J = 7.4 Hz, 2H), 7.45– 7.43 (m, 2H), 7.39 (t, J = 7.6 Hz, 4H), 7.27 – 7.26 (m, 3H), 7.17– 7.10 (m, 4H), 4.29– 4.14 (m, 2H), 1.20(t, J = 7.2 Hz,3H); ^{13}C NMR (100 MHz, $CDCl_3$) δ 165.5 (d, J_{C-F} = 29.7 Hz), 140.4, 134.0, 131.6, 130.3, 129.6 (d, J_{C-F} = 25.3 Hz), 128.76, 128.68, 128.41, 128.38, 128.3, 125.2, 122.6, 100.1 (d, J_{C-F} = 233.3 Hz), 91.3, 88.2, 63.5, 13.6; ^{19}F NMR (376 MHz, $CDCl_3$) δ -118.9; IR (KBr) 1771, 1750, 1606, 1508, 1477, 1449, 1382, 1363, 1288, 1265, 1211, 1190, 1176, 1078, 1048, 1026, 997, 854, 812, 755, 722, 684, 617, 609, 600, 576, 551 cm^{-1}; HRMS-(DART) (m/z): (M + NH_4)$^+$ calcd for $C_{30}H_{28}FN_2O_6S_2$, 595.1373; found595.1362.

Referecnes:

1 M. Hu, J. Rong, W. Miao, C. Ni, Y. Han and J. Hu, *Org. Lett.* 2014, **16**, 2030.

2 E. J. Park, S. Lee and S. Chang, *J. Org. Chem.* 2010, **75**, 2760.

3 G. K. Murphy, F. Z. Abbas and A. V. Poulton, *Adv. Synth. Catal.* 2014, **356**, 2919.

4 F. Ye, C. Wang, Y. Zhang and J. Wang, *Angew. Chem. Int. Ed.* **2014**, *53*, 11625.

5 F. Neese, *Wiley Interdiscip. Rev. Comput. Mol. Sci.* 2012, **2**, 73.

6 E.van Lenthe, E. J.Baerends and J. G. Snijders, *J. Chem. Phys.* 1993, **99**, 4597.

7 C. Lee, W. Yang and R. G. Parr, *Phys. Rev. B* 1988, **37**, 785.

8 F. Weigend and R. Ahlrichs, *Phys. Chem. Chem. Phys.* 2005, **7**, 3297.

9 R. Izsák, F. Neese and W. Klopper, *J. Chem. Phys.* 2013, **139**, 094111.

10 R. Izsák and F. Neese, *J. Chem. Phys.* 2011, **135**, 144105.

11 F. Neese, F. Wennmohs, A. Hansen and U. Becker, *Chem. Phys.* 2009, **356**, 98.

12 F. Weigend, *Phys. Chem. Chem. Phys.* 2006, **8**, 1057.

13 S. Grimme, S. Ehrlich and L. Goerigk, *J. Chem. Comput.* 2011, **32**, 1456.

14 S. Grimme, J. Antony, S. Ehrlich and H. Krieg, *J. Chem. Phys.* 2010, **132**, 154104.

15 A. V. Marenich, C. J. Cramer and D. G. Truhlar, *J. Phys. Chem. B* 2009, **113**, 6378.

16 A. Klamt and G. Schüürmann, *J. Chem. Soc. Perk. T. 2* 1993, 799.

^{1}H and ^{13}C NMR spectra for the prepared substrates

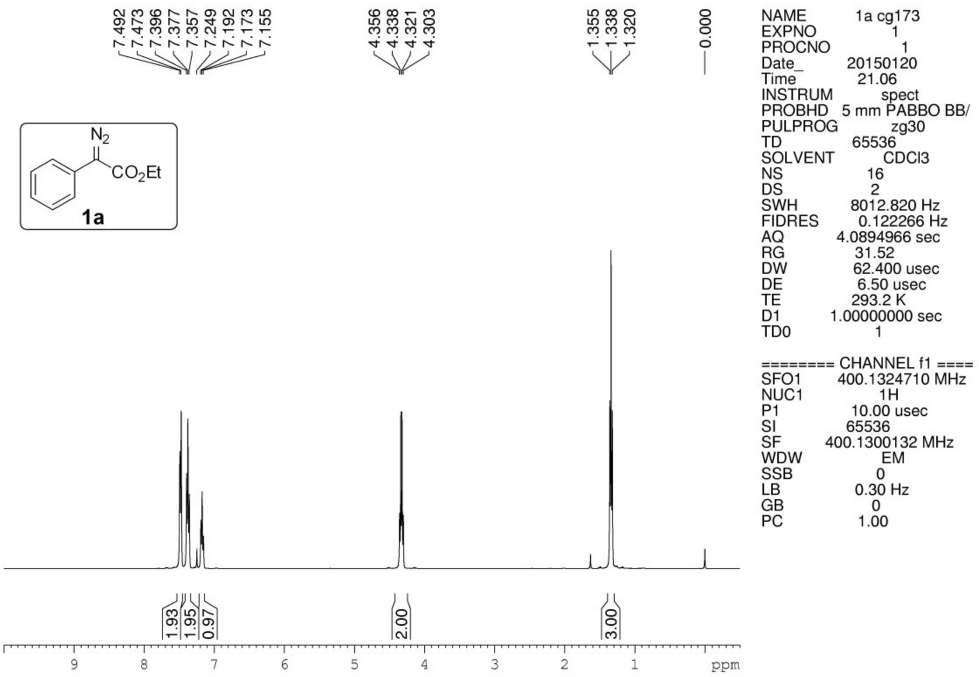

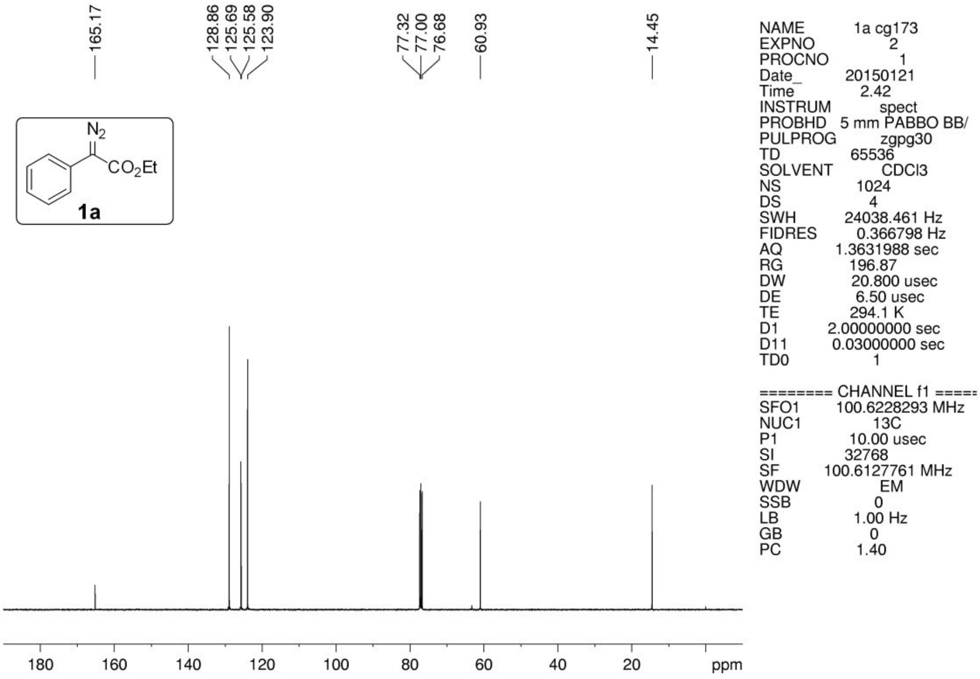

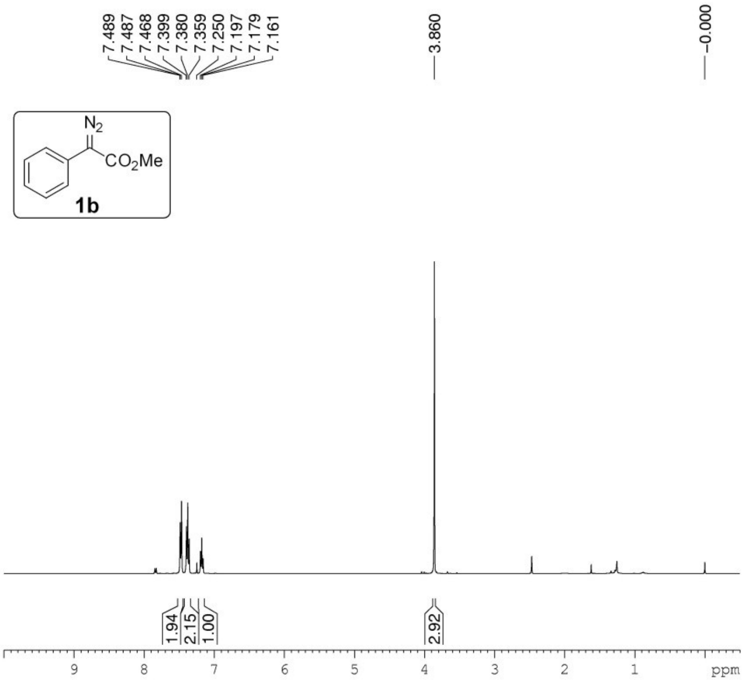

NAME 1b mzf156
EXPNO 1
PROCNO 1
Date_ 20141213
Time 23.16
INSTRUM spect
PROBHD 5 mm PABBO BB/
PULPROG zg30
TD 65536
SOLVENT CDCl3
NS 16
DS 2
SWH 8012.820 Hz
FIDRES 0.122266 Hz
AQ 4.0894966 sec
RG 48.95
DW 62.400 usec
DE 6.50 usec
TE 294.9 K
D1 1.00000000 sec
TD0 1

======== CHANNEL f1 ====
SFO1 400.1324710 MHz
NUC1 1H
P1 10.00 usec
SI 65536
SF 400.1300132 MHz
WDW EM
SSB 0
LB 0.30 Hz
GB 0
PC 1.00

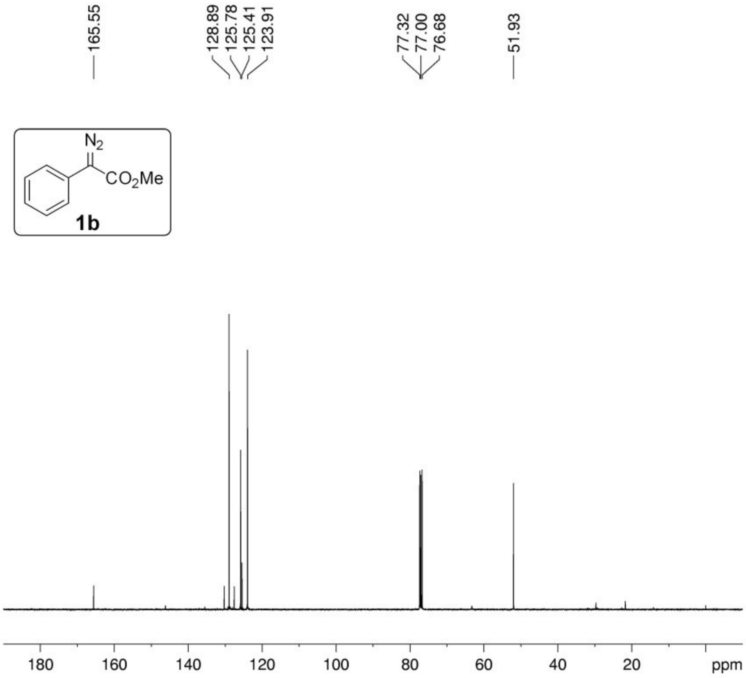

NAME 1b mzf156
EXPNO 2
PROCNO 1
Date_ 20141215
Time 3.23
INSTRUM spect
PROBHD 5 mm PABBO BB/
PULPROG zgpg30
TD 65536
SOLVENT CDCl3
NS 1024
DS 4
SWH 24038.461 Hz
FIDRES 0.366798 Hz
AQ 1.3631988 sec
RG 196.87
DW 20.800 usec
DE 6.50 usec
TE 296.0 K
D1 2.00000000 sec
D11 0.03000000 sec
TD0 1

======== CHANNEL f1 =====
SFO1 100.6228293 MHz
NUC1 13C
P1 10.00 usec
SI 32768
SF 100.6127757 MHz
WDW EM
SSB 0
LB 1.00 Hz
GB 0
PC 1.40

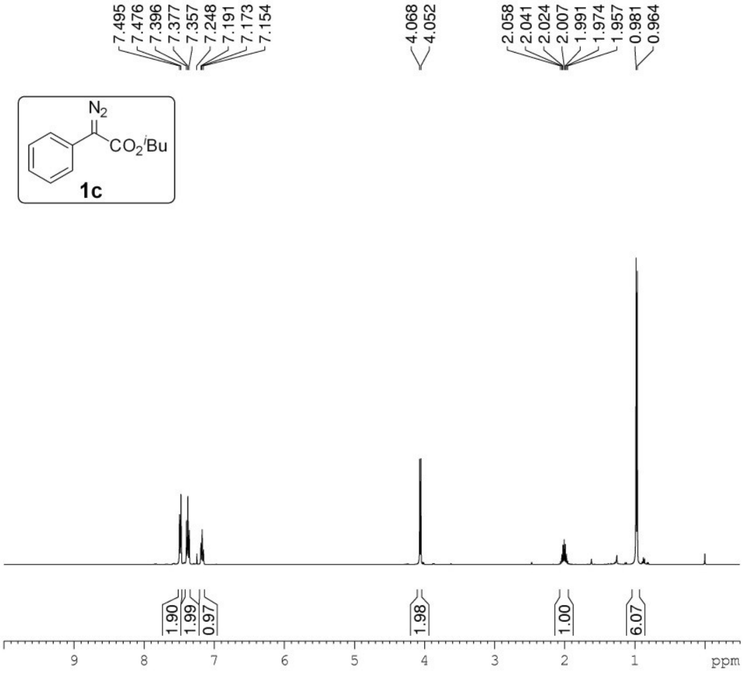

NAME 1c mzf159
EXPNO 1
PROCNO 1
Date_ 20141213
Time 23.10
INSTRUM spect
PROBHD 5 mm PABBO BB/
PULPROG zg30
TD 65536
SOLVENT CDCl3
NS 16
DS 2
SWH 8012.820 Hz
FIDRES 0.122266 Hz
AQ 4.0894966 sec
RG 31.52
DW 62.400 usec
DE 6.50 usec
TE 294.9 K
D1 1.00000000 sec
TD0 1

======== CHANNEL f1 ====
SFO1 400.1324710 MHz
NUC1 1H
P1 10.00 usec
SI 65536
SF 400.1300136 MHz
WDW EM
SSB 0
LB 0.30 Hz
GB 0
PC 1.00

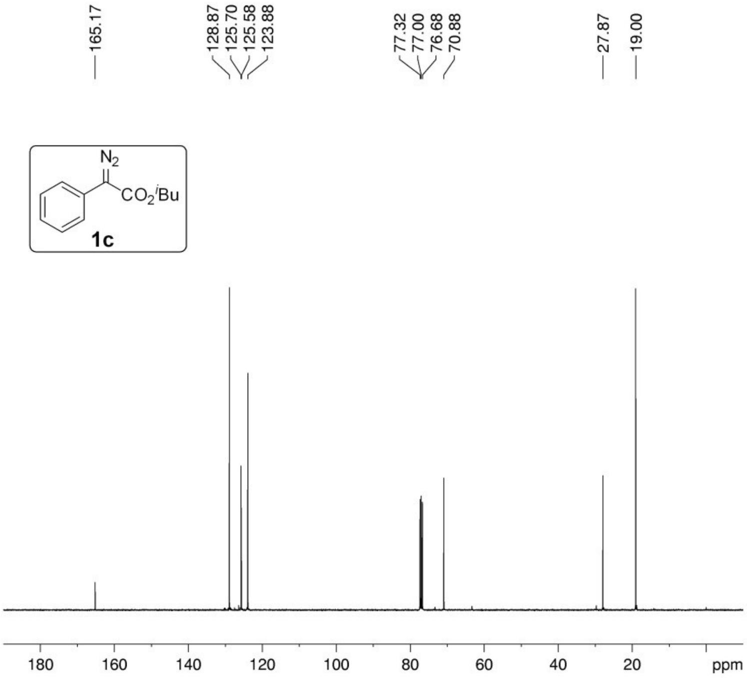

NAME 1c mzf159
EXPNO 2
PROCNO 1
Date_ 20141215
Time 5.30
INSTRUM spect
PROBHD 5 mm PABBO BB/
PULPROG zgpg30
TD 65536
SOLVENT CDCl3
NS 1024
DS 4
SWH 24038.461 Hz
FIDRES 0.366798 Hz
AQ 1.3631988 sec
RG 196.87
DW 20.800 usec
DE 6.50 usec
TE 296.0 K
D1 2.00000000 sec
D11 0.03000000 sec
TD0 1

======== CHANNEL f1 =====
SFO1 100.6228293 MHz
NUC1 13C
P1 10.00 usec
SI 32768
SF 100.6127754 MHz
WDW EM
SSB 0
LB 1.00 Hz
GB 0
PC 1.40

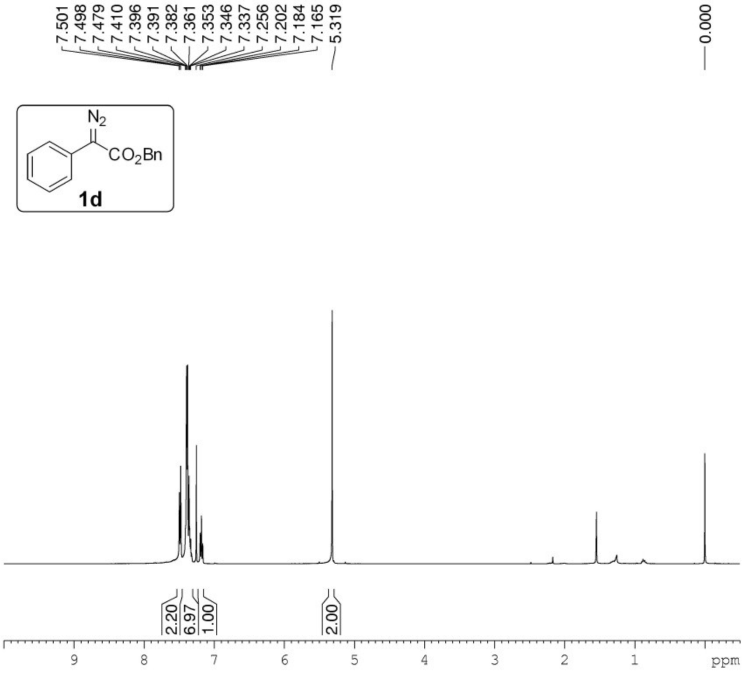

NAME 1d mzf155
EXPNO 1
PROCNO 1
Date_ 20141212
Time 3.04
INSTRUM spect
PROBHD 5 mm PABBO BB/
PULPROG zg30
TD 65536
SOLVENT CDCl3
NS 16
DS 2
SWH 8012.820 Hz
FIDRES 0.122266 Hz
AQ 4.0894966 sec
RG 110.75
DW 62.400 usec
DE 6.50 usec
TE 296.6 K
D1 1.00000000 sec
TD0 1

======== CHANNEL f1 ====
SFO1 400.1324710 MHz
NUC1 1H
P1 10.00 usec
SI 65536
SF 400.1300107 MHz
WDW EM
SSB 0
LB 0.30 Hz
GB 0
PC 1.00

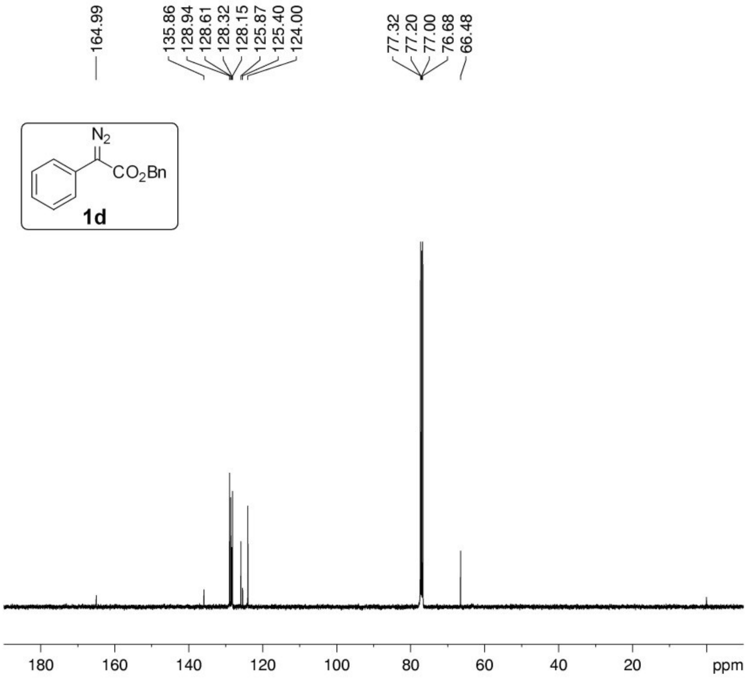

NAME 1d mzf155
EXPNO 2
PROCNO 1
Date_ 20141212
Time 4.03
INSTRUM spect
PROBHD 5 mm PABBO BB/
PULPROG zgpg30
TD 65536
SOLVENT CDCl3
NS 1024
DS 4
SWH 24038.461 Hz
FIDRES 0.366798 Hz
AQ 1.3631988 sec
RG 196.87
DW 20.800 usec
DE 6.50 usec
TE 297.4 K
D1 2.00000000 sec
D11 0.03000000 sec
TD0 1

======== CHANNEL f1 =====
SFO1 100.6228293 MHz
NUC1 13C
P1 10.00 usec
SI 32768
SF 100.6127713 MHz
WDW EM
SSB 0
LB 1.00 Hz
GB 0
PC 1.40

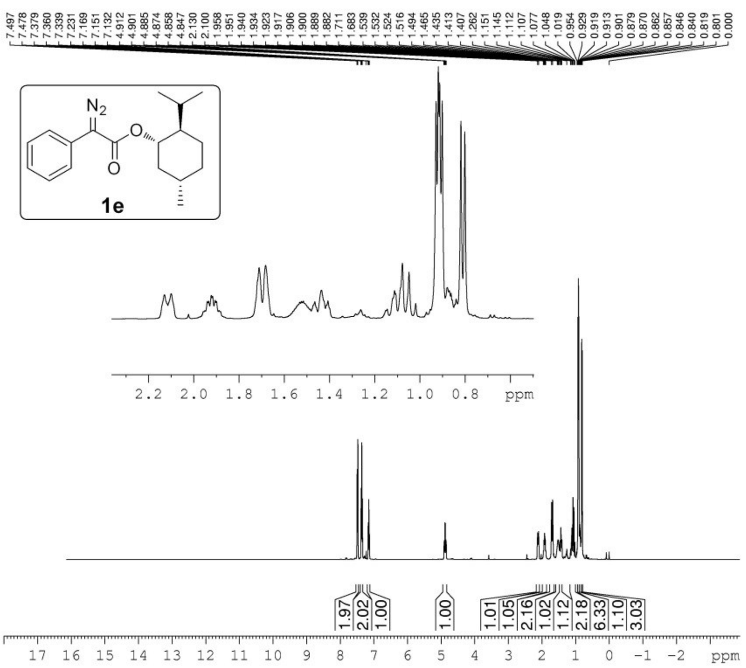

NAME 1e cg266
EXPNO 1
PROCNO 1
Date_ 20150417
Time 19.04
INSTRUM spect
PROBHD 5 mm PABBO BB/
PULPROG zg30
TD 65536
SOLVENT CDCl3
NS 8
DS 2
SWH 8012.820 Hz
FIDRES 0.122266 Hz
AQ 4.0894966 sec
RG 14.16
DW 62.400 usec
DE 6.50 usec
TE 298.1 K
D1 1.00000000 sec
TD0 1

======== CHANNEL f1 ====
SFO1 400.1324710 MHz
NUC1 1H
P1 10.00 usec
SI 65536
SF 400.1300202 MHz
WDW EM
SSB 0
LB 0.30 Hz
GB 0
PC 1.00

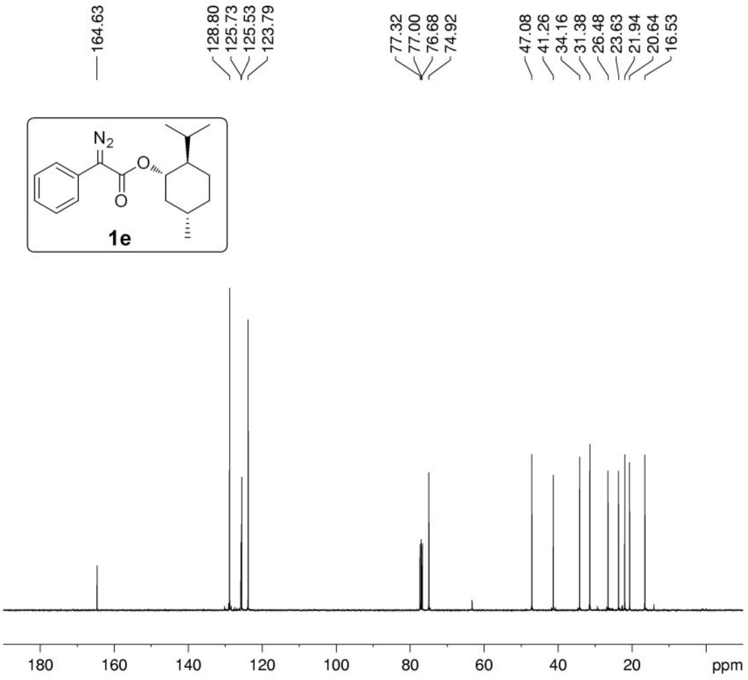

NAME 1e cg266
EXPNO 2
PROCNO 1
Date_ 20150417
Time 19.23
INSTRUM spect
PROBHD 5 mm PABBO BB/
PULPROG zgpg30
TD 65536
SOLVENT CDCl3
NS 300
DS 4
SWH 24038.461 Hz
FIDRES 0.366798 Hz
AQ 1.3631988 sec
RG 196.87
DW 20.800 usec
DE 6.50 usec
TE 298.8 K
D1 2.00000000 sec
D11 0.03000000 sec
TD0 1

======== CHANNEL f1 =====
SFO1 100.6228293 MHz
NUC1 13C
P1 10.00 usec
SI 32768
SF 100.6127798 MHz
WDW EM
SSB 0
LB 1.00 Hz
GB 0
PC 1.40

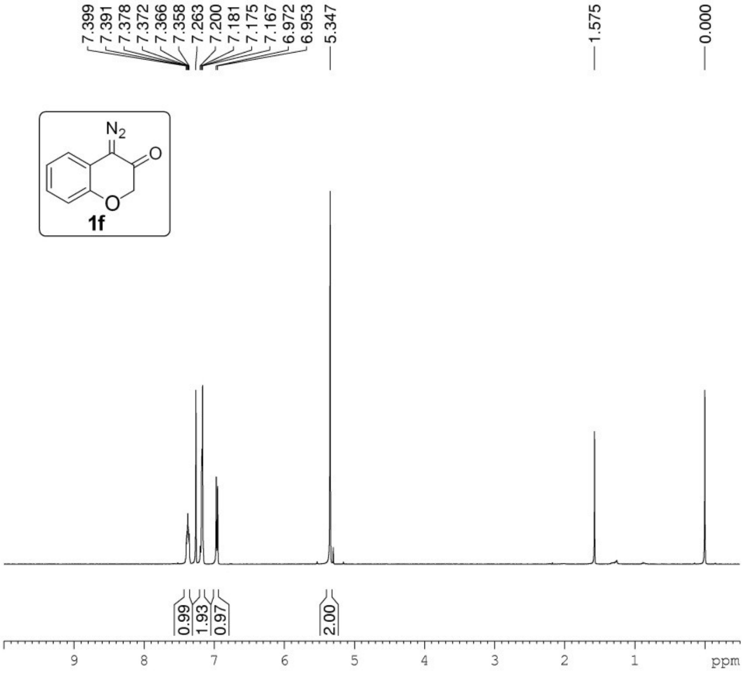

NAME 1f mzf160
EXPNO 1
PROCNO 1
Date_ 20141218
Time 7.37
INSTRUM spect
PROBHD 5 mm PABBO BB/
PULPROG zg30
TD 65536
SOLVENT CDCl3
NS 16
DS 2
SWH 8012.820 Hz
FIDRES 0.122266 Hz
AQ 4.0894966 sec
RG 124.06
DW 62.400 usec
DE 6.50 usec
TE 294.2 K
D1 1.00000000 sec
TD0 1

======== CHANNEL f1 ====
SFO1 400.1324710 MHz
NUC1 1H
P1 10.00 usec
SI 65536
SF 400.1300083 MHz
WDW EM
SSB 0
LB 0.30 Hz
GB 0
PC 1.00

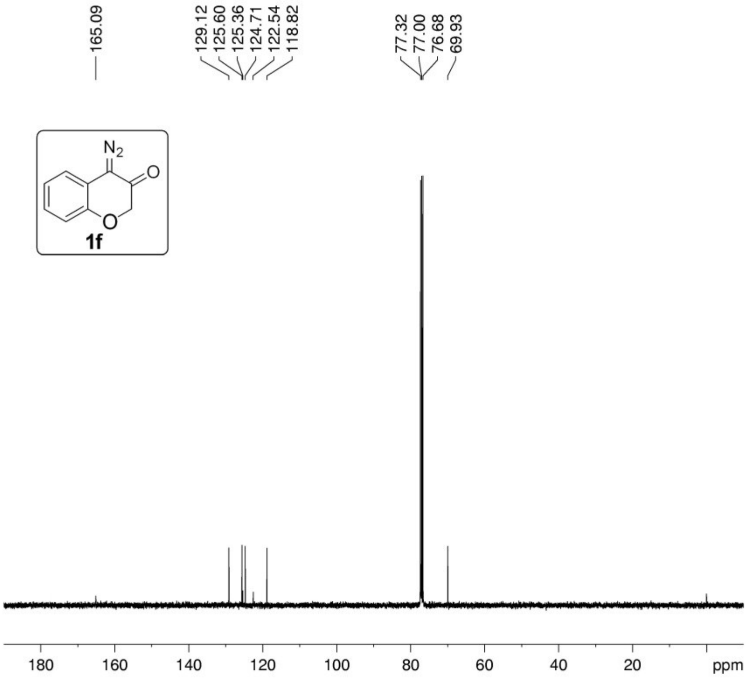

NAME 1f mzf160
EXPNO 2
PROCNO 1
Date_ 20141218
Time 8.06
INSTRUM spect
PROBHD 5 mm PABBO BB/
PULPROG zgpg30
TD 65536
SOLVENT CDCl3
NS 500
DS 4
SWH 24038.461 Hz
FIDRES 0.366798 Hz
AQ 1.3631988 sec
RG 196.87
DW 20.800 usec
DE 6.50 usec
TE 294.7 K
D1 2.00000000 sec
D11 0.03000000 sec
TD0 1

======== CHANNEL f1 =====
SFO1 100.6228293 MHz
NUC1 13C
P1 10.00 usec
SI 32768
SF 100.6127724 MHz
WDW EM
SSB 0
LB 1.00 Hz
GB 0
PC 1.40

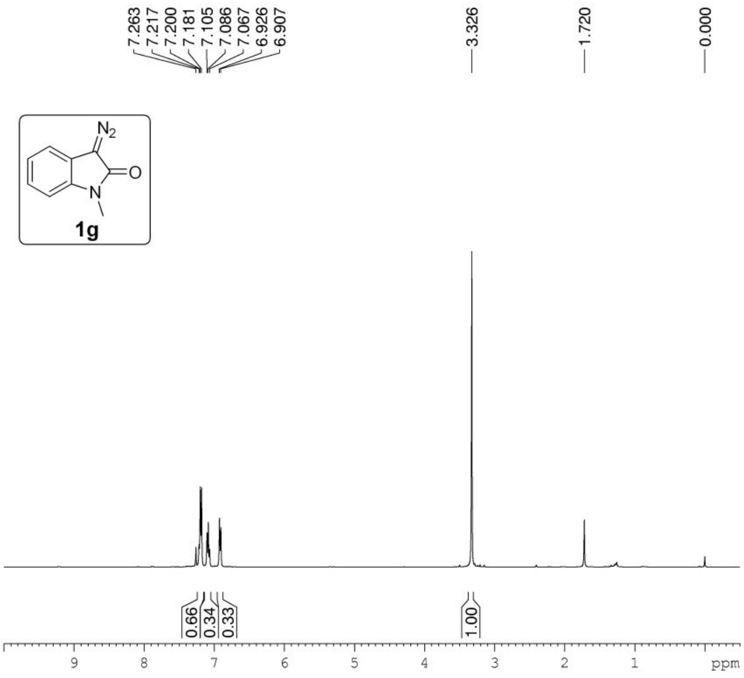
7.263 7.217 7.200 7.181 7.105 7.086 7.067 6.926 6.907
3.326
1.720
0.000
N2
O
N
1g
0.66 0.34 0.33
1.00
9 8 7 6 5 4 3 2 1 ppm
NAME 1g cg294
EXPNO 1
PROCNO 1
Date_ 20150509
Time 13.42
INSTRUM spect
PROBHD 5 mm PABBO BB/
PULPROG zg30
TD 65536
SOLVENT CDCl3
NS 16
DS 2
SWH 8012.820 Hz
FIDRES 0.122266 Hz
AQ 4.0894966 sec
RG 86.83
DW 62.400 usec
DE 6.50 usec
TE 301.6 K
D1 1.00000000 sec
TD0 1
======== CHANNEL f1 ====
SFO1 400.1324710 MHz
NUC1 1H
P1 10.00 usec
SI 65536
SF 400.1300080 MHz
WDW EM
SSB 0
LB 0.30 Hz
GB 0
PC 1.00

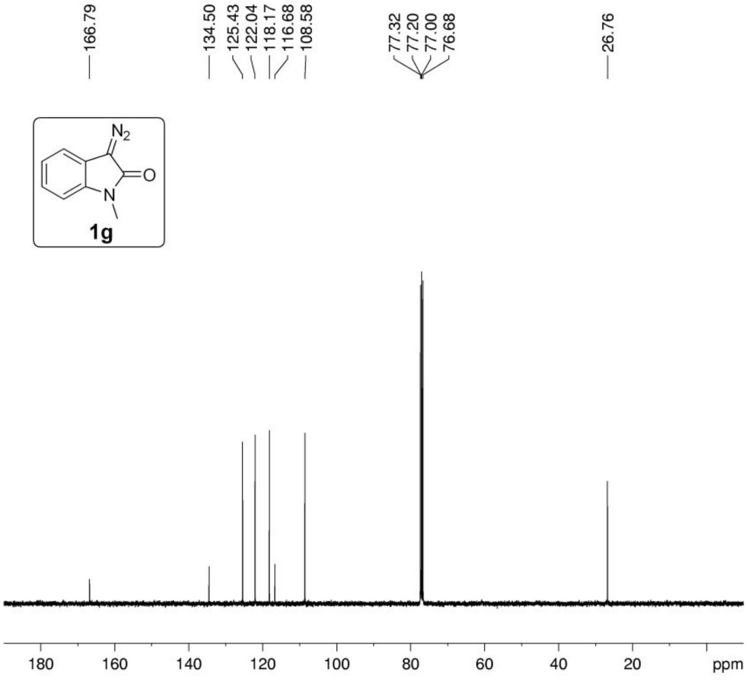
166.79
134.50 125.43 122.04 118.17 116.68 108.58
77.32 77.20 77.00 76.68
26.76
N2
O
N
1g
180 160 140 120 100 80 60 40 20 ppm
NAME 1g cg294
EXPNO 2
PROCNO 1
Date_ 20150509
Time 14.12
INSTRUM spect
PROBHD 5 mm PABBO BB/
PULPROG zgpg30
TD 65536
SOLVENT CDCl3
NS 500
DS 4
SWH 24038.461 Hz
FIDRES 0.366798 Hz
AQ 1.3631988 sec
RG 196.87
DW 20.800 usec
DE 6.50 usec
TE 302.3 K
D1 2.00000000 sec
D11 0.03000000 sec
TD0 1
======== CHANNEL f1 =====
SFO1 100.6228293 MHz
NUC1 13C
P1 10.00 usec
SI 32768
SF 100.6127724 MHz
WDW EM
SSB 0
LB 1.00 Hz
GB 0
PC 1.40

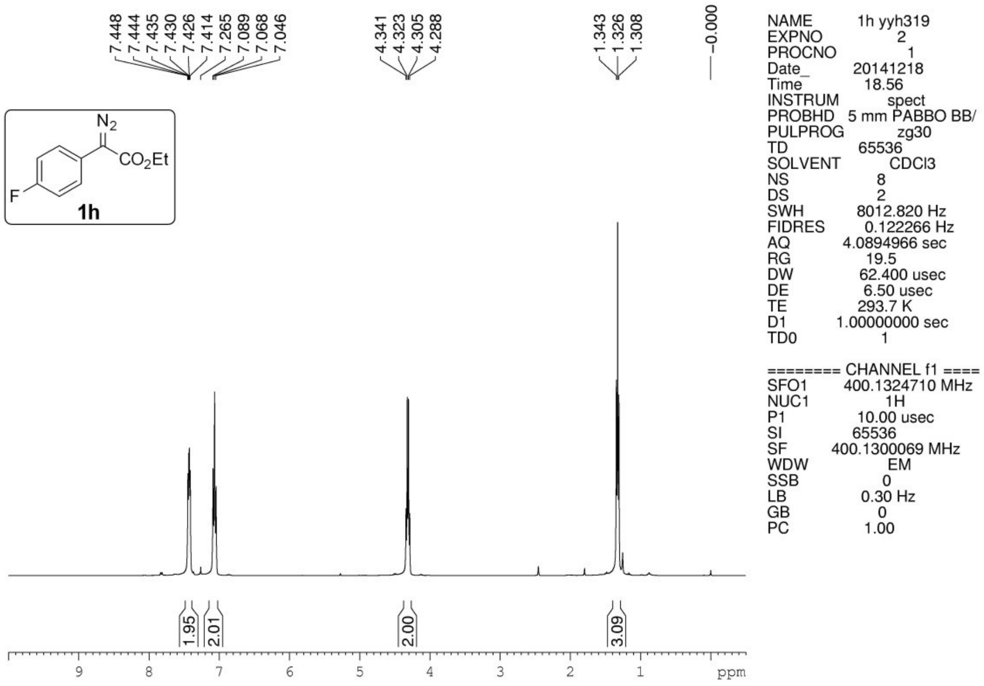
7.448 7.444 7.435 7.430 7.426 7.414 7.265 7.089 7.068 7.046
4.341 4.323 4.305 4.288
1.343 1.326 1.308
0.000
N2
CO2Et
F
1h
1.95 2.01 2.00 3.09
9 8 7 6 5 4 3 2 1 ppm
NAME 1h yyh319
EXPNO 2
PROCNO 1
Date_ 20141218
Time 18.56
INSTRUM spect
PROBHD 5 mm PABBO BB/
PULPROG zg30
TD 65536
SOLVENT CDCl3
NS 8
DS 2
SWH 8012.820 Hz
FIDRES 0.122266 Hz
AQ 4.0894966 sec
RG 19.5
DW 62.400 usec
DE 6.50 usec
TE 293.7 K
D1 1.00000000 sec
TD0 1
======== CHANNEL f1 ====
SFO1 400.1324710 MHz
NUC1 1H
P1 10.00 usec
SI 65536
SF 400.1300069 MHz
WDW EM
SSB 0
LB 0.30 Hz
GB 0
PC 1.00

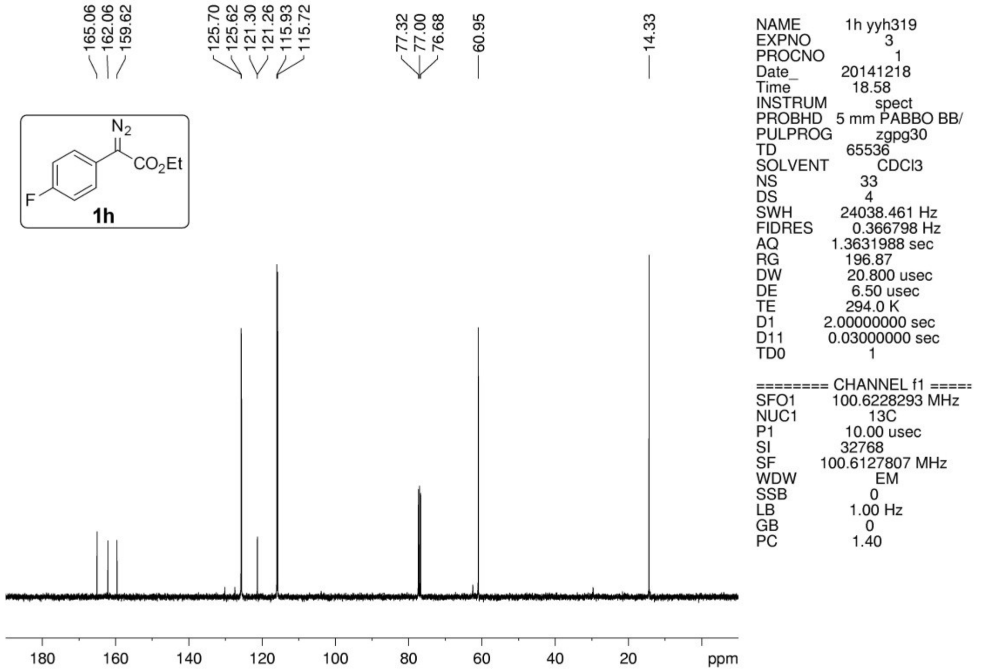
165.06 162.06 159.62
125.70 125.62 121.30 121.26 115.93 115.72
77.32 77.00 76.68
60.95
14.33
N2
CO2Et
F
1h
180 160 140 120 100 80 60 40 20 ppm
NAME 1h yyh319
EXPNO 3
PROCNO 1
Date_ 20141218
Time 18.58
INSTRUM spect
PROBHD 5 mm PABBO BB/
PULPROG zgpg30
TD 65536
SOLVENT CDCl3
NS 33
DS 4
SWH 24038.461 Hz
FIDRES 0.366798 Hz
AQ 1.3631988 sec
RG 196.87
DW 20.800 usec
DE 6.50 usec
TE 294.0 K
D1 2.00000000 sec
D11 0.03000000 sec
TD0 1
======== CHANNEL f1 =====
SFO1 100.6228293 MHz
NUC1 13C
P1 10.00 usec
SI 32768
SF 100.6127807 MHz
WDW EM
SSB 0
LB 1.00 Hz
GB 0
PC 1.40

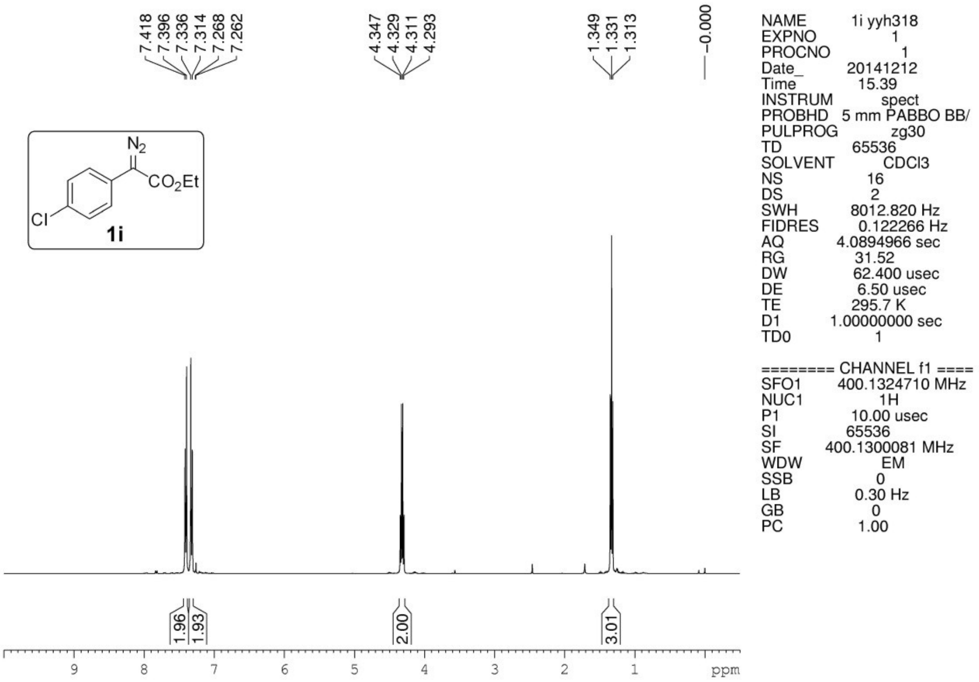
7.418
7.396
7.336
7.314
7.268
7.262
4.347
4.329
4.311
4.293
1.349
1.331
1.313
0.000
N2
CO2Et
Cl
1i
1.96
1.93
2.00
3.01
9 8 7 6 5 4 3 2 1 ppm
NAME 1i yyh318
EXPNO 1
PROCNO 1
Date_ 20141212
Time 15.39
INSTRUM spect
PROBHD 5 mm PABBO BB/
PULPROG zg30
TD 65536
SOLVENT CDCl3
NS 16
DS 2
SWH 8012.820 Hz
FIDRES 0.122266 Hz
AQ 4.0894966 sec
RG 31.52
DW 62.400 usec
DE 6.50 usec
TE 295.7 K
D1 1.00000000 sec
TD0 1
======== CHANNEL f1 ====
SFO1 400.1324710 MHz
NUC1 1H
P1 10.00 usec
SI 65536
SF 400.1300081 MHz
WDW EM
SSB 0
LB 0.30 Hz
GB 0
PC 1.00

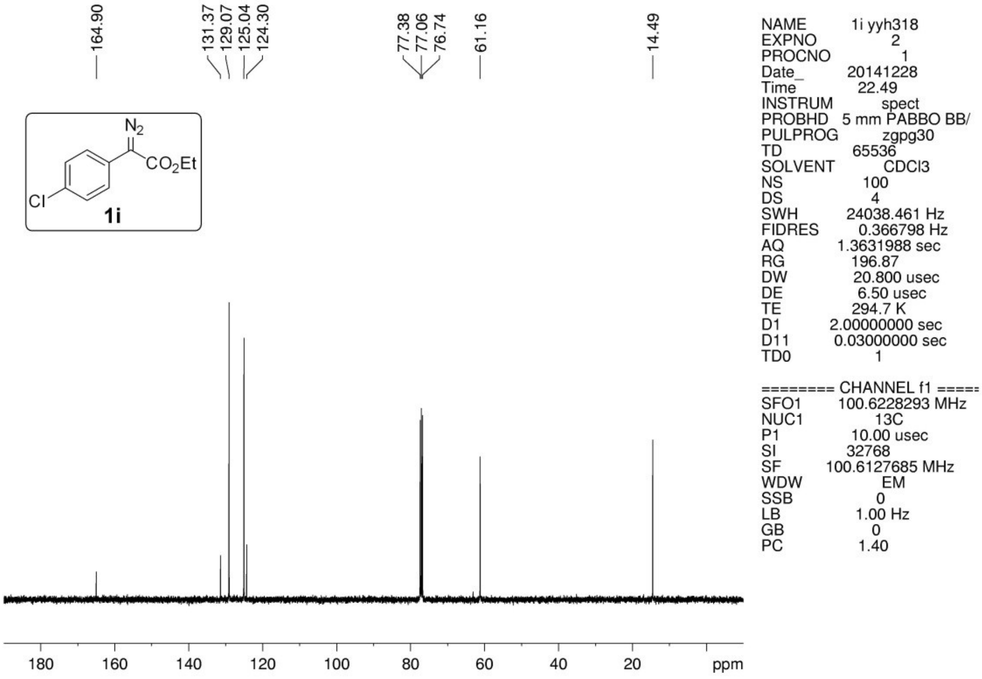
164.90
131.37
129.07
125.04
124.30
77.38
77.06
76.74
61.16
14.49
N2
CO2Et
Cl
1i
180 160 140 120 100 80 60 40 20 ppm
NAME 1i yyh318
EXPNO 2
PROCNO 1
Date_ 20141228
Time 22.49
INSTRUM spect
PROBHD 5 mm PABBO BB/
PULPROG zgpg30
TD 65536
SOLVENT CDCl3
NS 100
DS 4
SWH 24038.461 Hz
FIDRES 0.366798 Hz
AQ 1.3631988 sec
RG 196.87
DW 20.800 usec
DE 6.50 usec
TE 294.7 K
D1 2.00000000 sec
D11 0.03000000 sec
TD0 1
======== CHANNEL f1 =====
SFO1 100.6228293 MHz
NUC1 13C
P1 10.00 usec
SI 32768
SF 100.6127685 MHz
WDW EM
SSB 0
LB 1.00 Hz
GB 0
PC 1.40

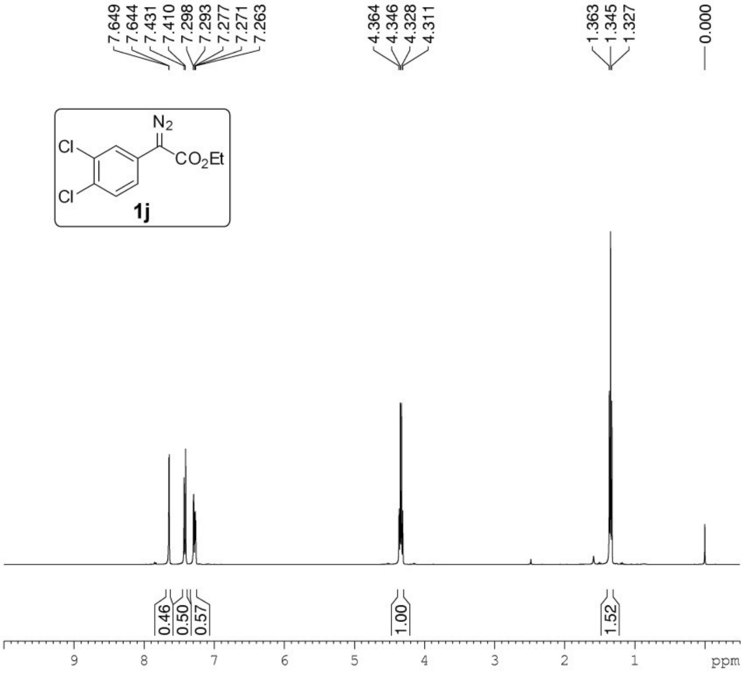

NAME 1j yyh321
EXPNO 1
PROCNO 1
Date_ 20141213
Time 17.28
INSTRUM spect
PROBHD 5 mm PABBO BB/
PULPROG zg30
TD 65536
SOLVENT CDCl3
NS 8
DS 2
SWH 8012.820 Hz
FIDRES 0.122266 Hz
AQ 4.0894966 sec
RG 70.24
DW 62.400 usec
DE 6.50 usec
TE 295.5 K
D1 1.00000000 sec
TD0 1

======== CHANNEL f1 ====
SFO1 400.1324710 MHz
NUC1 1H
P1 10.00 usec
SI 65536
SF 400.1300079 MHz
WDW EM
SSB 0
LB 0.30 Hz
GB 0
PC 1.00

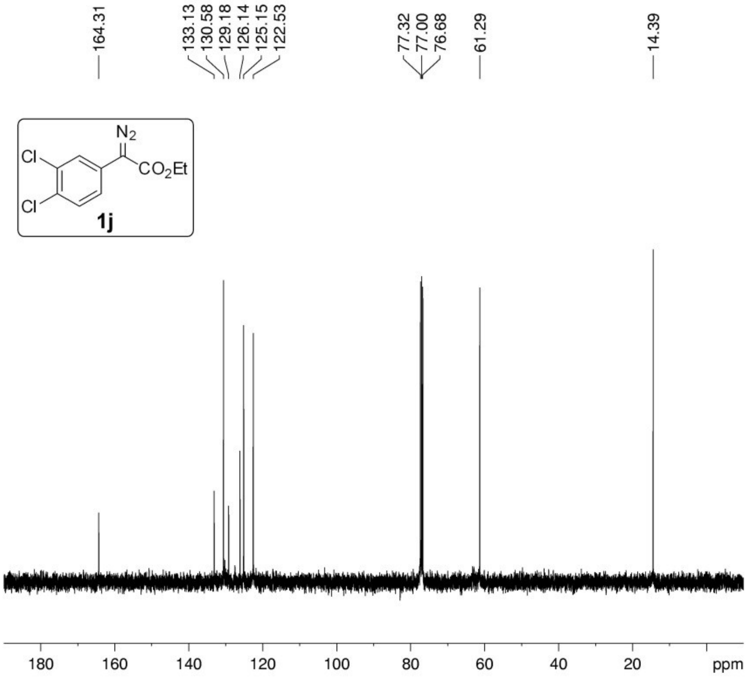

NAME 1j yyh321
EXPNO 3
PROCNO 1
Date_ 20141218
Time 21.47
INSTRUM spect
PROBHD 5 mm PABBO BB/
PULPROG zgpg30
TD 65536
SOLVENT CDCl3
NS 50
DS 4
SWH 24038.461 Hz
FIDRES 0.366798 Hz
AQ 1.3631988 sec
RG 196.87
DW 20.800 usec
DE 6.50 usec
TE 294.1 K
D1 2.00000000 sec
D11 0.03000000 sec
TD0 1

======== CHANNEL f1 =====
SFO1 100.6228293 MHz
NUC1 13C
P1 10.00 usec
SI 32768
SF 100.6127761 MHz
WDW EM
SSB 0
LB 1.00 Hz
GB 0
PC 1.40

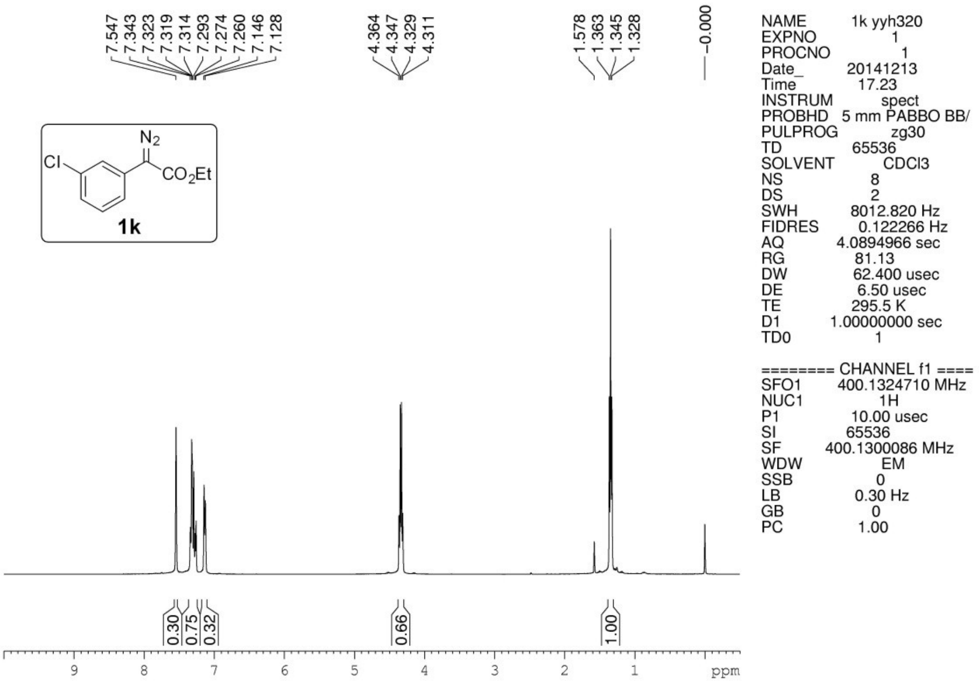
7.547 7.343 7.323 7.319 7.314 7.293 7.274 7.260 7.146 7.128
4.364 4.347 4.329 4.311
1.578 1.363 1.345 1.328
0.000
Cl
N2
CO2Et
1k
0.30 0.75 0.32 0.66 1.00
9 8 7 6 5 4 3 2 1 ppm
NAME 1k yyh320
EXPNO 1
PROCNO 1
Date_ 20141213
Time 17.23
INSTRUM spect
PROBHD 5 mm PABBO BB/
PULPROG zg30
TD 65536
SOLVENT CDCl3
NS 8
DS 2
SWH 8012.820 Hz
FIDRES 0.122266 Hz
AQ 4.0894966 sec
RG 81.13
DW 62.400 usec
DE 6.50 usec
TE 295.5 K
D1 1.00000000 sec
TD0 1
======== CHANNEL f1 ====
SFO1 400.1324710 MHz
NUC1 1H
P1 10.00 usec
SI 65536
SF 400.1300086 MHz
WDW EM
SSB 0
LB 0.30 Hz
GB 0
PC 1.00

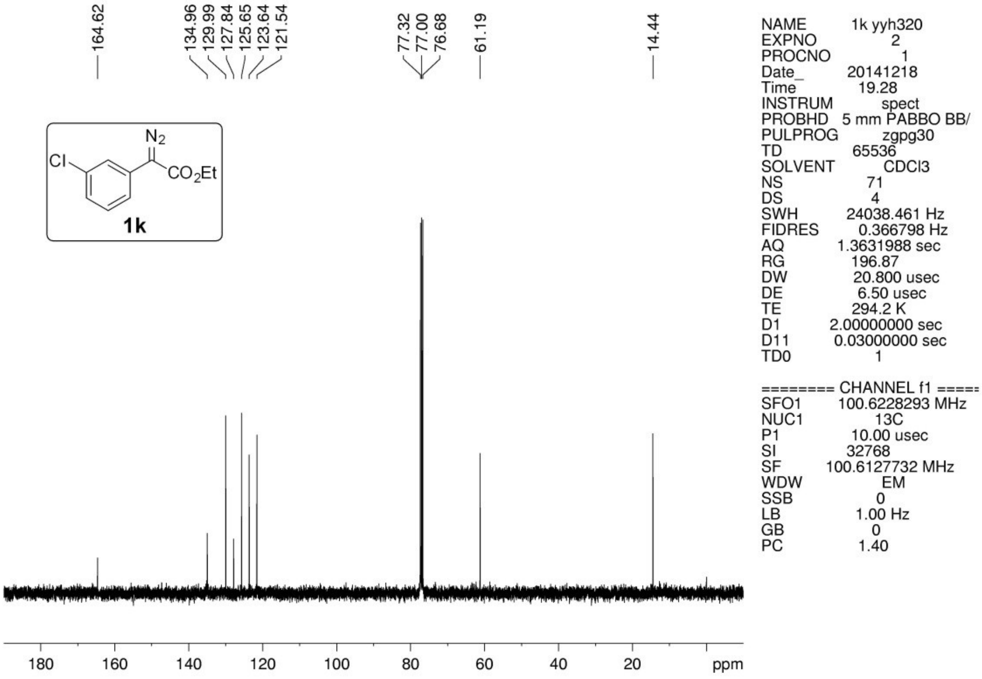
164.62
134.96 129.99 127.84 125.65 123.64 121.54
77.32 77.00 76.68
61.19
14.44
Cl
N2
CO2Et
1k
180 160 140 120 100 80 60 40 20 ppm
NAME 1k yyh320
EXPNO 2
PROCNO 1
Date_ 20141218
Time 19.28
INSTRUM spect
PROBHD 5 mm PABBO BB/
PULPROG zgpg30
TD 65536
SOLVENT CDCl3
NS 71
DS 4
SWH 24038.461 Hz
FIDRES 0.366798 Hz
AQ 1.3631988 sec
RG 196.87
DW 20.800 usec
DE 6.50 usec
TE 294.2 K
D1 2.00000000 sec
D11 0.03000000 sec
TD0 1
======== CHANNEL f1 =====
SFO1 100.6228293 MHz
NUC1 13C
P1 10.00 usec
SI 32768
SF 100.6127732 MHz
WDW EM
SSB 0
LB 1.00 Hz
GB 0
PC 1.40

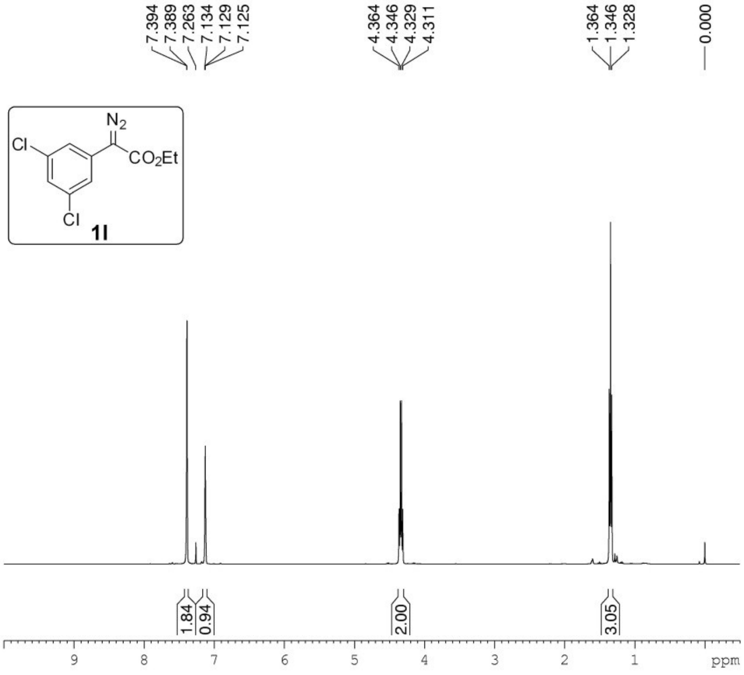

NAME 1l cg239b
EXPNO 1
PROCNO 1
Date_ 20150421
Time 12.04
INSTRUM spect
PROBHD 5 mm PABBO BB/
PULPROG zg30
TD 65536
SOLVENT CDCl3
NS 8
DS 2
SWH 8012.820 Hz
FIDRES 0.122266 Hz
AQ 4.0894966 sec
RG 54.34
DW 62.400 usec
DE 6.50 usec
TE 298.6 K
D1 1.00000000 sec
TD0 1

======== CHANNEL f1 ====
SFO1 400.1324710 MHz
NUC1 1H
P1 10.00 usec
SI 65536
SF 400.1300076 MHz
WDW EM
SSB 0
LB 0.30 Hz
GB 0
PC 1.00

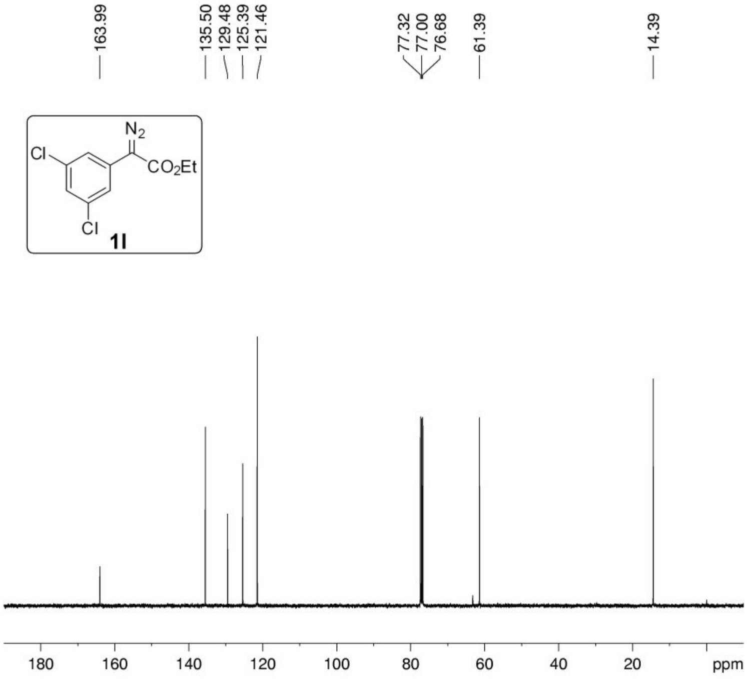

NAME 1l cg239b
EXPNO 2
PROCNO 1
Date_ 20150421
Time 12.26
INSTRUM spect
PROBHD 5 mm PABBO BB/
PULPROG zgpg30
TD 65536
SOLVENT CDCl3
NS 350
DS 4
SWH 24038.461 Hz
FIDRES 0.366798 Hz
AQ 1.3631988 sec
RG 196.87
DW 20.800 usec
DE 6.50 usec
TE 299.4 K
D1 2.00000000 sec
D11 0.03000000 sec
TD0 1

======== CHANNEL f1 =====
SFO1 100.6228293 MHz
NUC1 13C
P1 10.00 usec
SI 32768
SF 100.6127727 MHz
WDW EM
SSB 0
LB 1.00 Hz
GB 0
PC 1.40

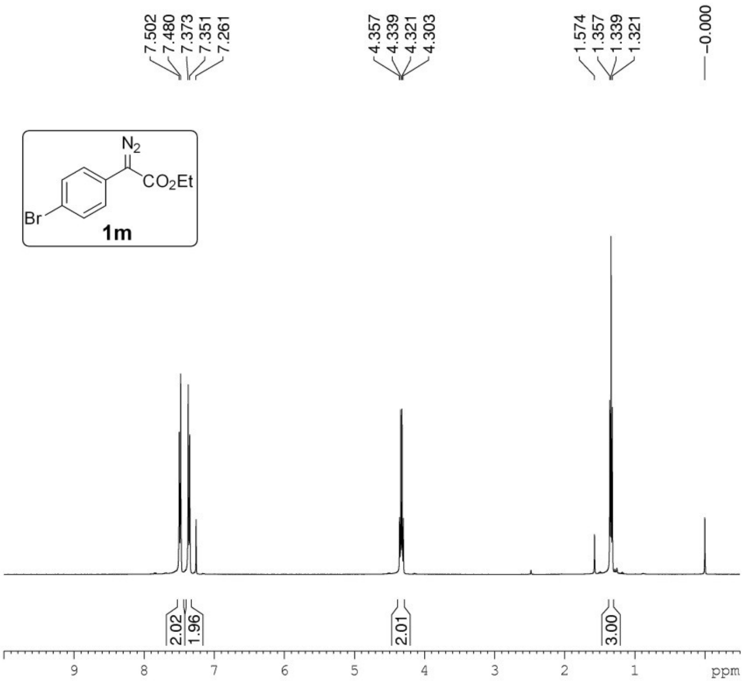

NAME 1m yyh311
EXPNO 1
PROCNO 1
Date_ 20141210
Time 19.51
INSTRUM spect
PROBHD 5 mm PABBO BB/
PULPROG zg30
TD 65536
SOLVENT CDCl3
NS 16
DS 2
SWH 8012.820 Hz
FIDRES 0.122266 Hz
AQ 4.0894966 sec
RG 86.83
DW 62.400 usec
DE 6.50 usec
TE 296.4 K
D1 1.00000000 sec
TD0 1

======== CHANNEL f1 ====
SFO1 400.1324710 MHz
NUC1 1H
P1 10.00 usec
SI 65536
SF 400.1300091 MHz
WDW EM
SSB 0
LB 0.30 Hz
GB 0
PC 1.00

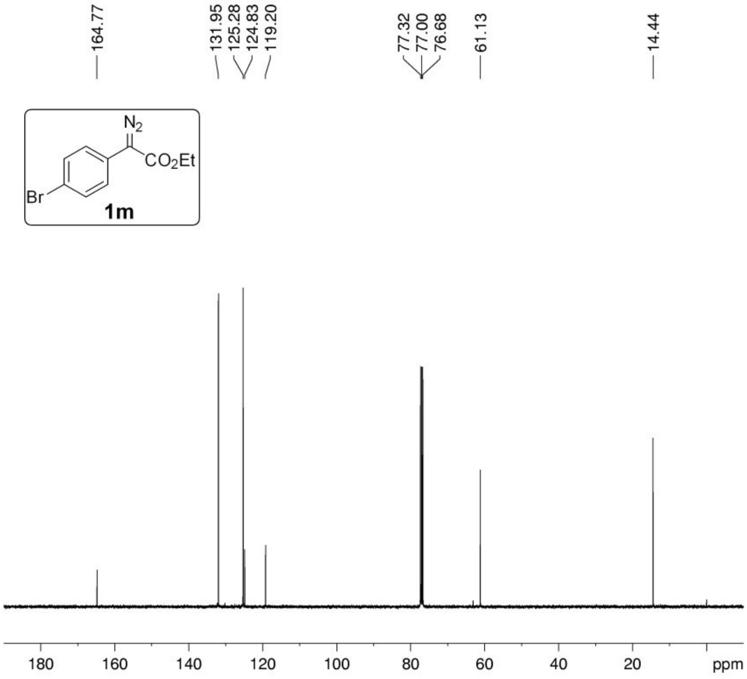

NAME 1m yyh311
EXPNO 2
PROCNO 1
Date_ 20141211
Time 1.09
INSTRUM spect
PROBHD 5 mm PABBO BB/
PULPROG zgpg30
TD 65536
SOLVENT CDCl3
NS 500
DS 4
SWH 24038.461 Hz
FIDRES 0.366798 Hz
AQ 1.3631988 sec
RG 196.87
DW 20.800 usec
DE 6.50 usec
TE 296.8 K
D1 2.00000000 sec
D11 0.03000000 sec
TD0 1

======== CHANNEL f1 =====
SFO1 100.6228293 MHz
NUC1 13C
P1 10.00 usec
SI 32768
SF 100.6127735 MHz
WDW EM
SSB 0
LB 1.00 Hz
GB 0
PC 1.40

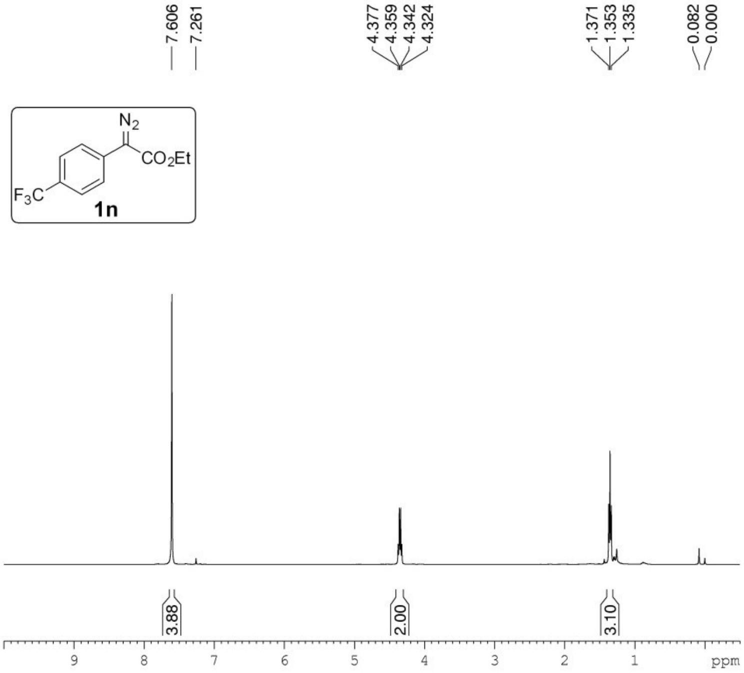

NAME 1n cg236
EXPNO 1
PROCNO 1
Date_ 20150327
Time 0.05
INSTRUM spect
PROBHD 5 mm PABBO BB/
PULPROG zg30
TD 65536
SOLVENT CDCl3
NS 4
DS 2
SWH 8012.820 Hz
FIDRES 0.122266 Hz
AQ 4.0894966 sec
RG 31.52
DW 62.400 usec
DE 6.50 usec
TE 294.9 K
D1 1.00000000 sec
TD0 1

======== CHANNEL f1 ====
SFO1 400.1324710 MHz
NUC1 1H
P1 10.00 usec
SI 65536
SF 400.1300080 MHz
WDW EM
SSB 0
LB 0.30 Hz
GB 0
PC 1.00

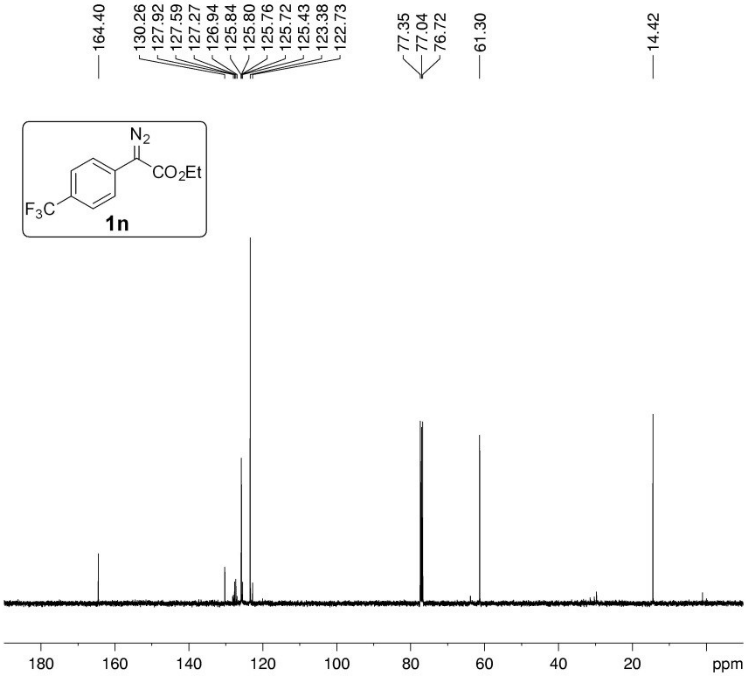

NAME 1n cg236
EXPNO 2
PROCNO 1
Date_ 20150327
Time 0.12
INSTRUM spect
PROBHD 5 mm PABBO BB/
PULPROG zgpg30
TD 65536
SOLVENT CDCl3
NS 100
DS 4
SWH 24038.461 Hz
FIDRES 0.366798 Hz
AQ 1.3631988 sec
RG 196.87
DW 20.800 usec
DE 6.50 usec
TE 295.5 K
D1 2.00000000 sec
D11 0.03000000 sec
TD0 1

======== CHANNEL f1 =====
SFO1 100.6228293 MHz
NUC1 13C
P1 10.00 usec
SI 32768
SF 100.6127685 MHz
WDW EM
SSB 0
LB 1.00 Hz
GB 0
PC 1.40

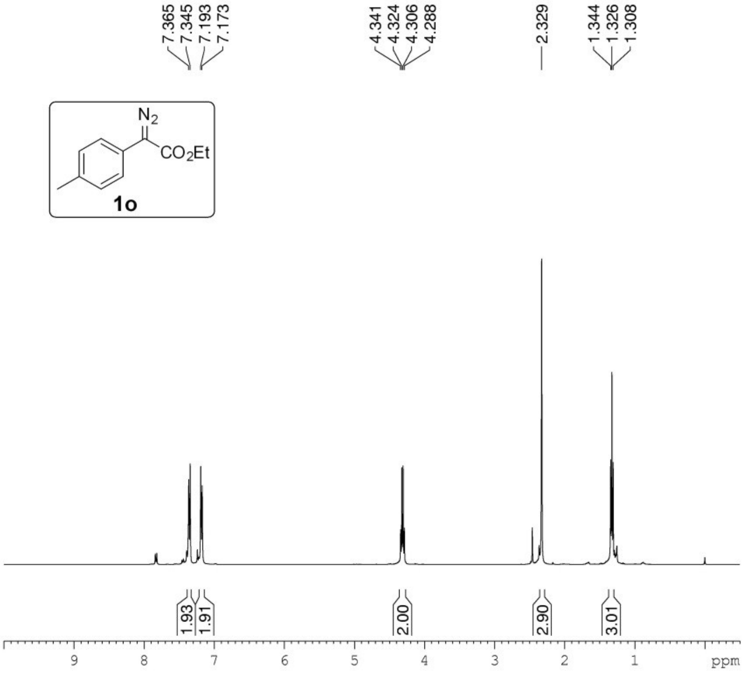

NAME 1o mzf164
EXPNO 1
PROCNO 1
Date_ 20141225
Time 4.36
INSTRUM spect
PROBHD 5 mm PABBO BB/
PULPROG zg30
TD 65536
SOLVENT CDCl3
NS 16
DS 2
SWH 8012.820 Hz
FIDRES 0.122266 Hz
AQ 4.0894966 sec
RG 31.52
DW 62.400 usec
DE 6.50 usec
TE 296.1 K
D1 1.00000000 sec
TD0 1

======== CHANNEL f1 ====
SFO1 400.1324710 MHz
NUC1 1H
P1 10.00 usec
SI 65536
SF 400.1300163 MHz
WDW EM
SSB 0
LB 0.30 Hz
GB 0
PC 1.00

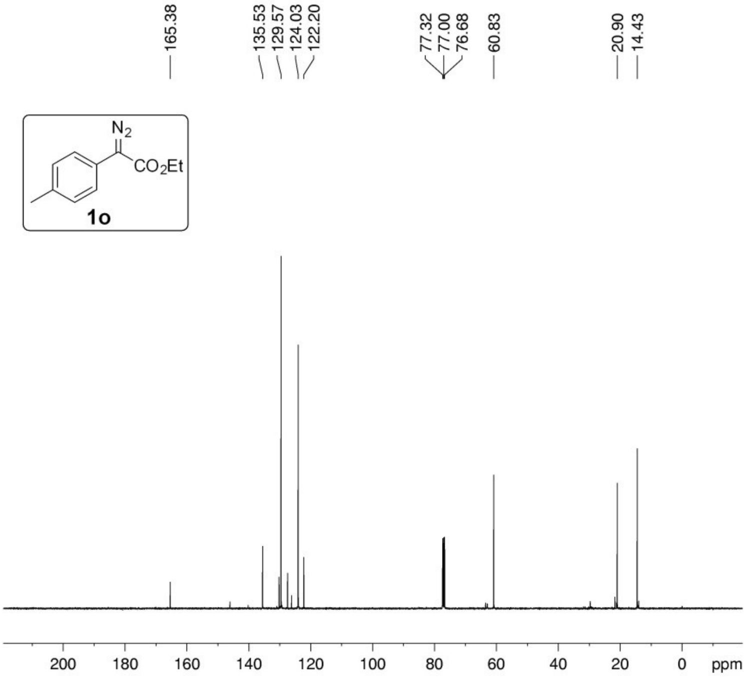

NAME 1o mzf164
EXPNO 2
PROCNO 1
Date_ 20141225
Time 5.06
INSTRUM spect
PROBHD 5 mm PABBO BB/
PULPROG zgpg30
TD 65536
SOLVENT CDCl3
NS 500
DS 4
SWH 24038.461 Hz
FIDRES 0.366798 Hz
AQ 1.3631988 sec
RG 196.87
DW 20.800 usec
DE 6.50 usec
TE 297.0 K
D1 2.00000000 sec
D11 0.03000000 sec
TD0 1

======== CHANNEL f1 =====
SFO1 100.6228293 MHz
NUC1 13C
P1 10.00 usec
SI 32768
SF 100.6127783 MHz
WDW EM
SSB 0
LB 1.00 Hz
GB 0
PC 1.40

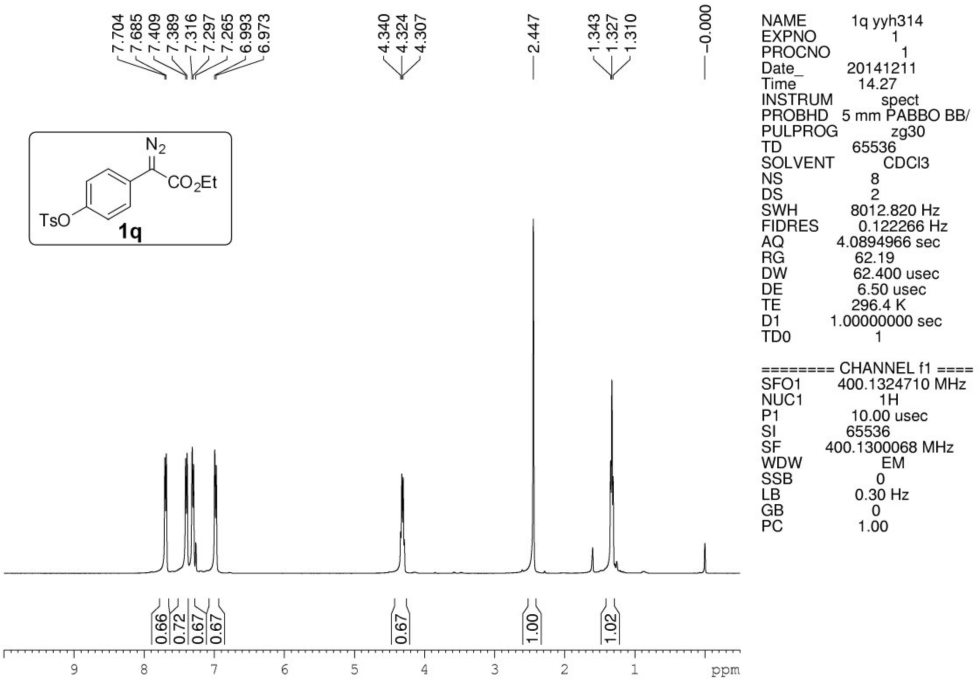
NAME 1q yyh314
EXPNO 1
PROCNO 1
Date_ 20141211
Time 14.27
INSTRUM spect
PROBHD 5 mm PABBO BB/
PULPROG zg30
TD 65536
SOLVENT CDCl3
NS 8
DS 2
SWH 8012.820 Hz
FIDRES 0.122266 Hz
AQ 4.0894966 sec
RG 62.19
DW 62.400 usec
DE 6.50 usec
TE 296.4 K
D1 1.00000000 sec
TD0 1
======== CHANNEL f1 ====
SFO1 400.1324710 MHz
NUC1 1H
P1 10.00 usec
SI 65536
SF 400.1300068 MHz
WDW EM
SSB 0
LB 0.30 Hz
GB 0
PC 1.00
TsO
1q
ppm

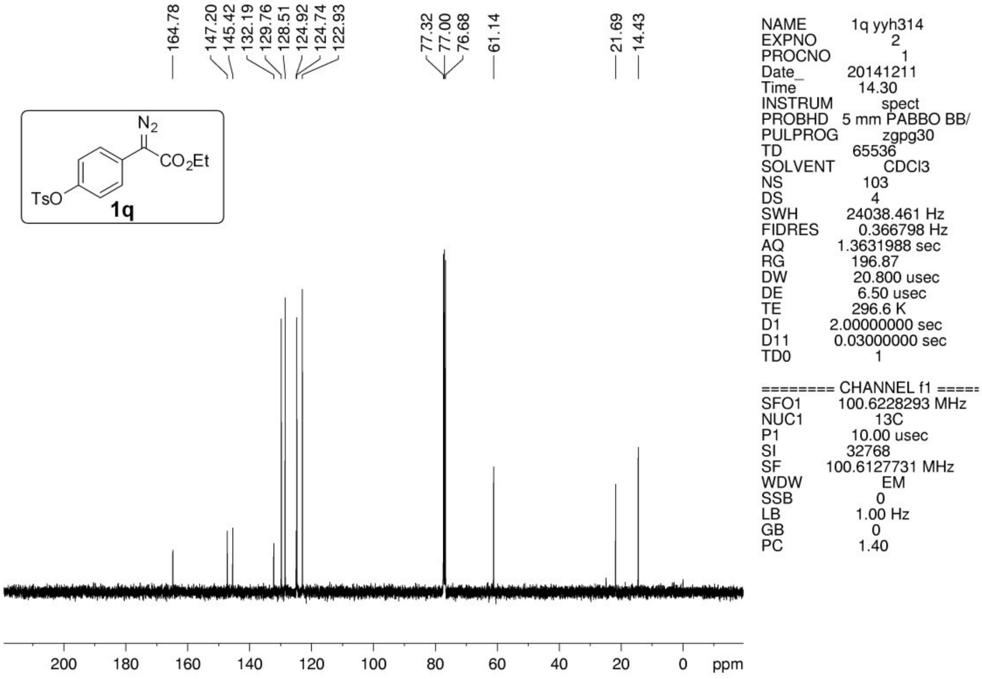
NAME 1q yyh314
EXPNO 2
PROCNO 1
Date_ 20141211
Time 14.30
INSTRUM spect
PROBHD 5 mm PABBO BB/
PULPROG zgpg30
TD 65536
SOLVENT CDCl3
NS 103
DS 4
SWH 24038.461 Hz
FIDRES 0.366798 Hz
AQ 1.3631988 sec
RG 196.87
DW 20.800 usec
DE 6.50 usec
TE 296.6 K
D1 2.00000000 sec
D11 0.03000000 sec
TD0 1
======== CHANNEL f1 =====
SFO1 100.6228293 MHz
NUC1 13C
P1 10.00 usec
SI 32768
SF 100.6127731 MHz
WDW EM
SSB 0
LB 1.00 Hz
GB 0
PC 1.40
TsO
1q
ppm

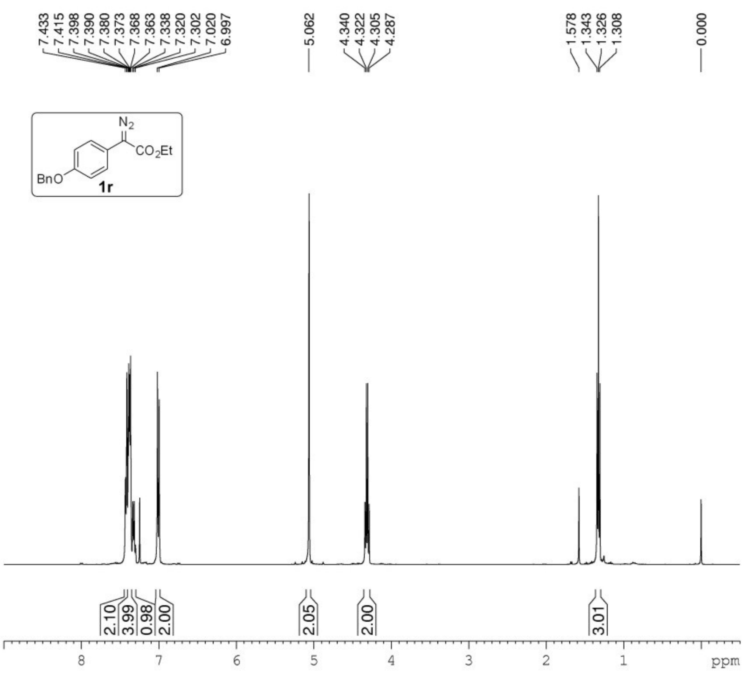

NAME 1r cg240
EXPNO 2
PROCNO 1
Date_ 20150329
Time 12.46
INSTRUM spect
PROBHD 5 mm PABBO BB/
PULPROG zg30
TD 65536
SOLVENT CDCl3
NS 4
DS 2
SWH 8012.820 Hz
FIDRES 0.122266 Hz
AQ 4.0894966 sec
RG 70.24
DW 62.400 usec
DE 6.50 usec
TE 296.1 K
D1 1.00000000 sec
TD0 1

======== CHANNEL f1 ====
SFO1 400.1324710 MHz
NUC1 1H
P1 10.00 usec
SI 65536
SF 400.1300133 MHz
WDW EM
SSB 0
LB 0.30 Hz
GB 0
PC 1.00

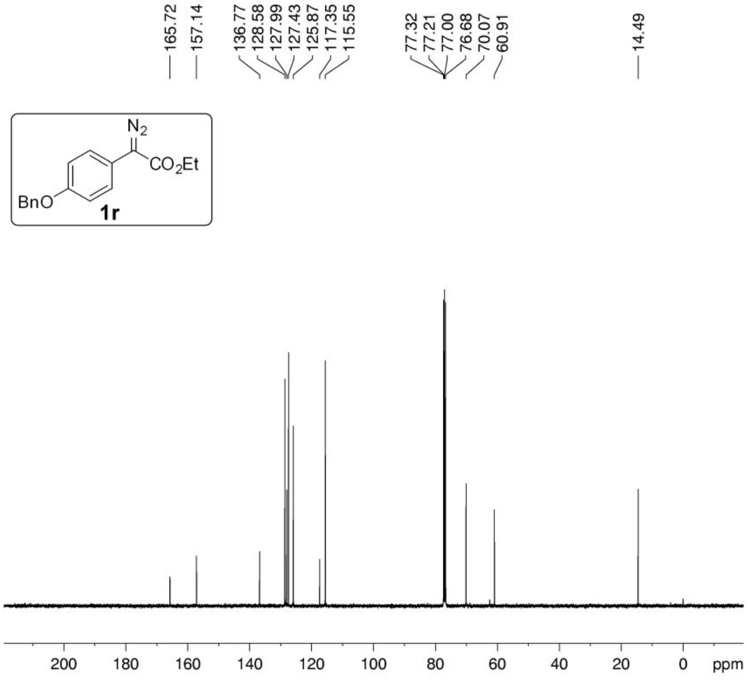

NAME 1r cg240
EXPNO 1
PROCNO 1
Date_ 20150329
Time 12.07
INSTRUM spect
PROBHD 5 mm PABBO BB/
PULPROG zgpg30
TD 65536
SOLVENT CDCl3
NS 845
DS 4
SWH 24038.461 Hz
FIDRES 0.366798 Hz
AQ 1.3631988 sec
RG 196.87
DW 20.800 usec
DE 6.50 usec
TE 296.9 K
D1 2.00000000 sec
D11 0.03000000 sec
TD0 1

======== CHANNEL f1 =====
SFO1 100.6228293 MHz
NUC1 13C
P1 10.00 usec
SI 32768
SF 100.6127731 MHz
WDW EM
SSB 0
LB 1.00 Hz
GB 0
PC 1.40

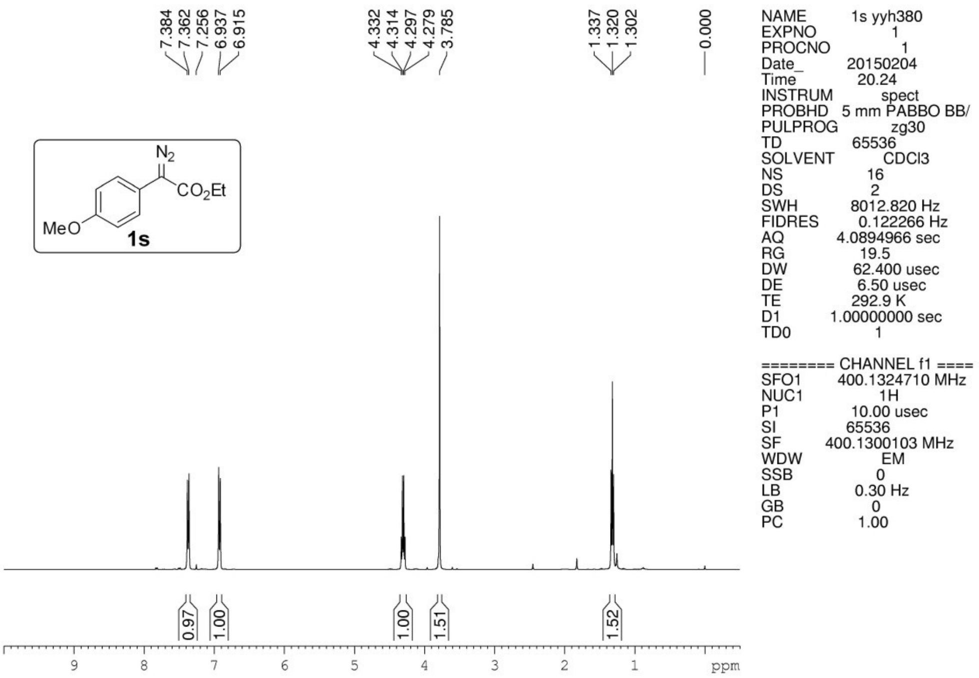
7.384
7.362
7.256
6.937
6.915
4.332
4.314
4.297
4.279
3.785
1.337
1.320
1.302
0.000
N2
CO2Et
MeO
1s
0.97
1.00
1.00
1.51
1.52
9 8 7 6 5 4 3 2 1 ppm
NAME 1s yyh380
EXPNO 1
PROCNO 1
Date_ 20150204
Time 20.24
INSTRUM spect
PROBHD 5 mm PABBO BB/
PULPROG zg30
TD 65536
SOLVENT CDCl3
NS 16
DS 2
SWH 8012.820 Hz
FIDRES 0.122266 Hz
AQ 4.0894966 sec
RG 19.5
DW 62.400 usec
DE 6.50 usec
TE 292.9 K
D1 1.00000000 sec
TD0 1
======== CHANNEL f1 ====
SFO1 400.1324710 MHz
NUC1 1H
P1 10.00 usec
SI 65536
SF 400.1300103 MHz
WDW EM
SSB 0
LB 0.30 Hz
GB 0
PC 1.00

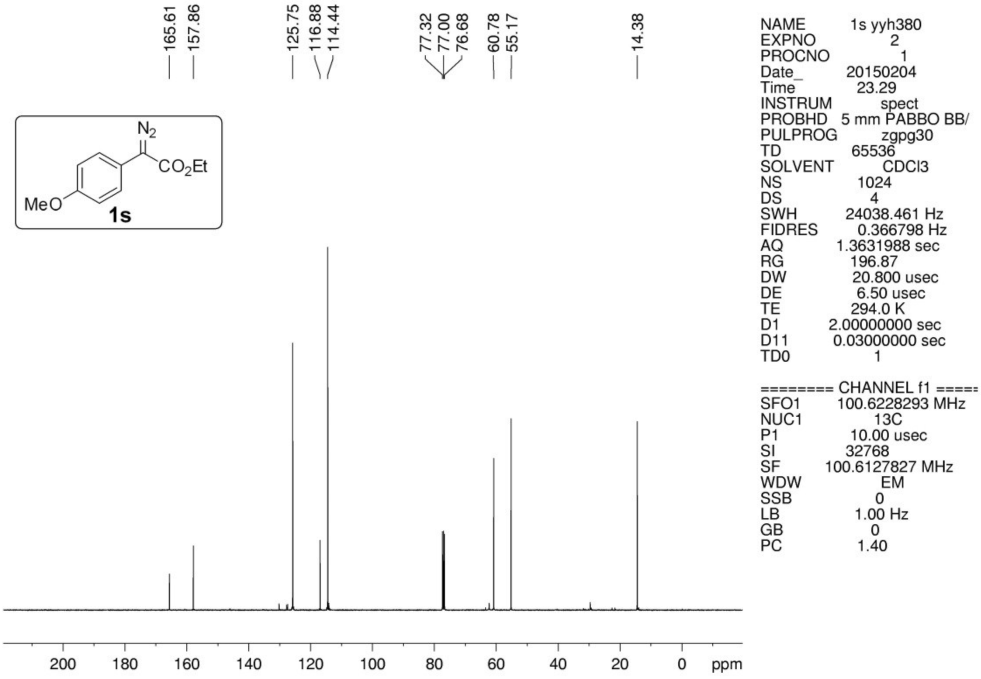
165.61
157.86
125.75
116.88
114.44
77.32
77.00
76.68
60.78
55.17
14.38
N2
CO2Et
MeO
1s
200 180 160 140 120 100 80 60 40 20 0 ppm
NAME 1s yyh380
EXPNO 2
PROCNO 1
Date_ 20150204
Time 23.29
INSTRUM spect
PROBHD 5 mm PABBO BB/
PULPROG zgpg30
TD 65536
SOLVENT CDCl3
NS 1024
DS 4
SWH 24038.461 Hz
FIDRES 0.366798 Hz
AQ 1.3631988 sec
RG 196.87
DW 20.800 usec
DE 6.50 usec
TE 294.0 K
D1 2.00000000 sec
D11 0.03000000 sec
TD0 1
======== CHANNEL f1 =====
SFO1 100.6228293 MHz
NUC1 13C
P1 10.00 usec
SI 32768
SF 100.6127827 MHz
WDW EM
SSB 0
LB 1.00 Hz
GB 0
PC 1.40

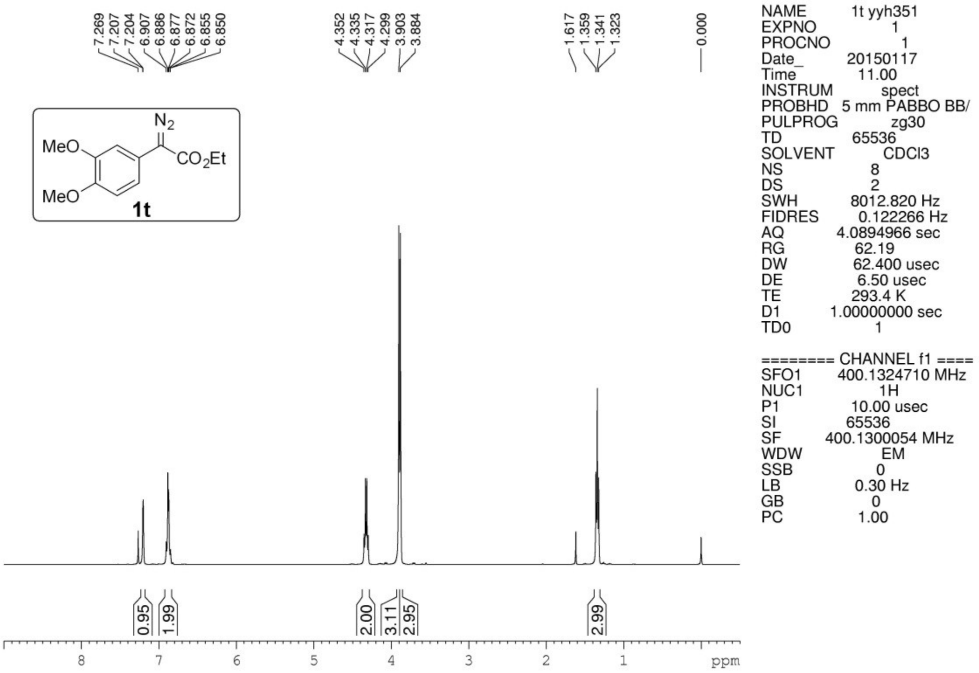
7.269
7.207
7.204
6.907
6.886
6.877
6.872
6.855
6.850
4.352
4.335
4.317
4.299
3.903
3.884
1.617
1.359
1.341
1.323
0.000
MeO
MeO
N2
CO2Et
1t
0.95
1.99
2.00
3.11
2.95
2.99
8
7
6
5
4
3
2
1
ppm
NAME 1t yyh351
EXPNO 1
PROCNO 1
Date_ 20150117
Time 11.00
INSTRUM spect
PROBHD 5 mm PABBO BB/
PULPROG zg30
TD 65536
SOLVENT CDCl3
NS 8
DS 2
SWH 8012.820 Hz
FIDRES 0.122266 Hz
AQ 4.0894966 sec
RG 62.19
DW 62.400 usec
DE 6.50 usec
TE 293.4 K
D1 1.00000000 sec
TD0 1
======== CHANNEL f1 ====
SFO1 400.1324710 MHz
NUC1 1H
P1 10.00 usec
SI 65536
SF 400.1300054 MHz
WDW EM
SSB 0
LB 0.30 Hz
GB 0
PC 1.00

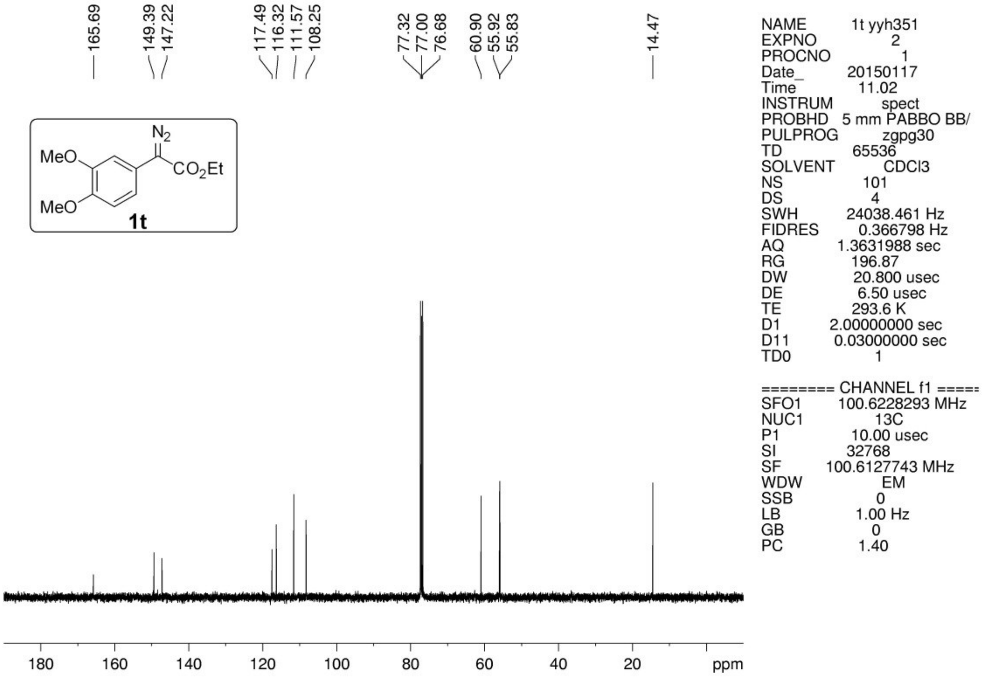
165.69
149.39
147.22
117.49
116.32
111.57
108.25
77.32
77.00
76.68
60.90
55.92
55.83
14.47
MeO
MeO
N2
CO2Et
1t
180
160
140
120
100
80
60
40
20
ppm
NAME 1t yyh351
EXPNO 2
PROCNO 1
Date_ 20150117
Time 11.02
INSTRUM spect
PROBHD 5 mm PABBO BB/
PULPROG zgpg30
TD 65536
SOLVENT CDCl3
NS 101
DS 4
SWH 24038.461 Hz
FIDRES 0.366798 Hz
AQ 1.3631988 sec
RG 196.87
DW 20.800 usec
DE 6.50 usec
TE 293.6 K
D1 2.00000000 sec
D11 0.03000000 sec
TD0 1
======== CHANNEL f1 =====
SFO1 100.6228293 MHz
NUC1 13C
P1 10.00 usec
SI 32768
SF 100.6127743 MHz
WDW EM
SSB 0
LB 1.00 Hz
GB 0
PC 1.40

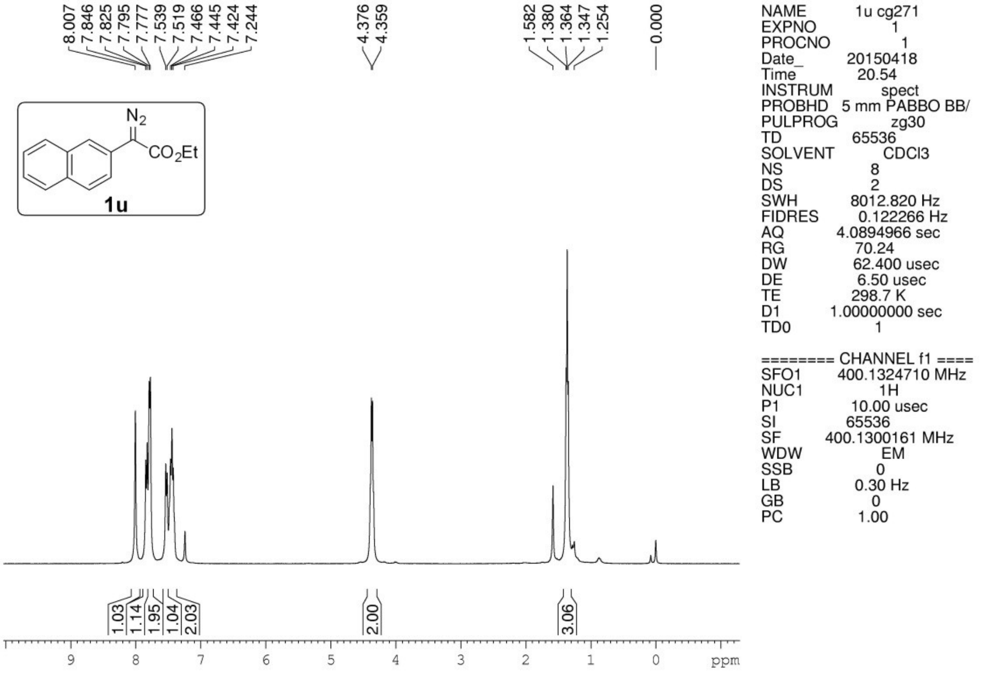
8.007
7.846
7.825
7.795
7.777
7.539
7.519
7.466
7.445
7.424
7.244
4.376
4.359
1.582
1.380
1.364
1.347
1.254
0.000
N_2
CO_2Et
1u
1.03
1.14
1.95
1.04
2.03
2.00
3.06
9 8 7 6 5 4 3 2 1 0 ppm
NAME 1u cg271
EXPNO 1
PROCNO 1
Date_ 20150418
Time 20.54
INSTRUM spect
PROBHD 5 mm PABBO BB/
PULPROG zg30
TD 65536
SOLVENT CDCl3
NS 8
DS 2
SWH 8012.820 Hz
FIDRES 0.122266 Hz
AQ 4.0894966 sec
RG 70.24
DW 62.400 usec
DE 6.50 usec
TE 298.7 K
D1 1.00000000 sec
TD0 1
======== CHANNEL f1 ====
SFO1 400.1324710 MHz
NUC1 1H
P1 10.00 usec
SI 65536
SF 400.1300161 MHz
WDW EM
SSB 0
LB 0.30 Hz
GB 0
PC 1.00

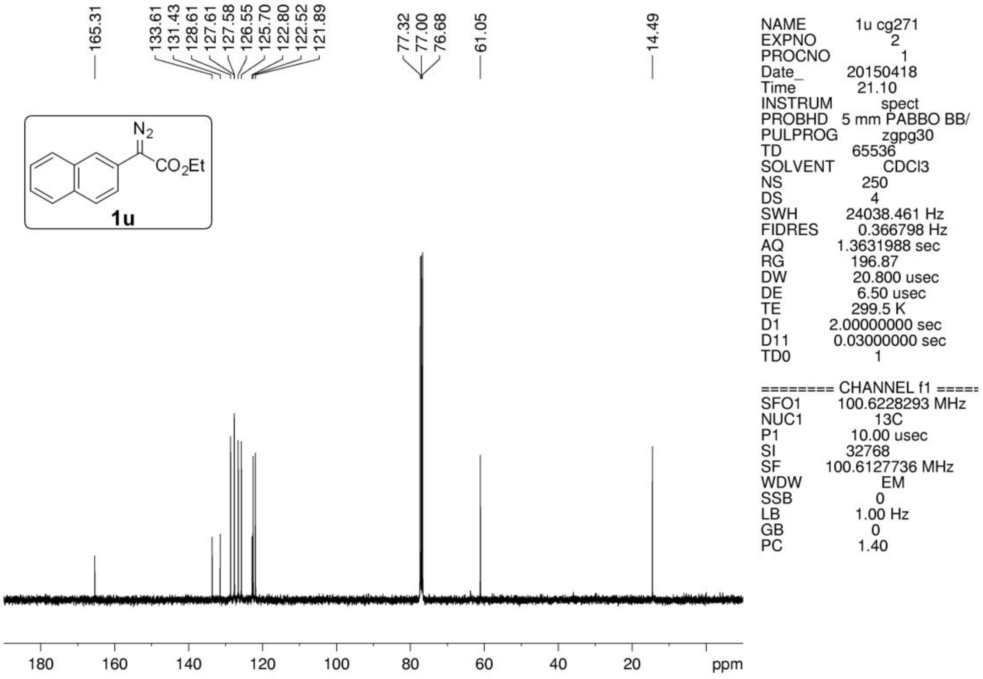
165.31
133.61
131.43
128.61
127.61
127.58
126.55
125.70
122.80
122.52
121.89
77.32
77.00
76.68
61.05
14.49
N_2
CO_2Et
1u
180 160 140 120 100 80 60 40 20 ppm
NAME 1u cg271
EXPNO 2
PROCNO 1
Date_ 20150418
Time 21.10
INSTRUM spect
PROBHD 5 mm PABBO BB/
PULPROG zgpg30
TD 65536
SOLVENT CDCl3
NS 250
DS 4
SWH 24038.461 Hz
FIDRES 0.366798 Hz
AQ 1.3631988 sec
RG 196.87
DW 20.800 usec
DE 6.50 usec
TE 299.5 K
D1 2.00000000 sec
D11 0.03000000 sec
TD0 1
======== CHANNEL f1 =====
SFO1 100.6228293 MHz
NUC1 13C
P1 10.00 usec
SI 32768
SF 100.6127736 MHz
WDW EM
SSB 0
LB 1.00 Hz
GB 0
PC 1.40

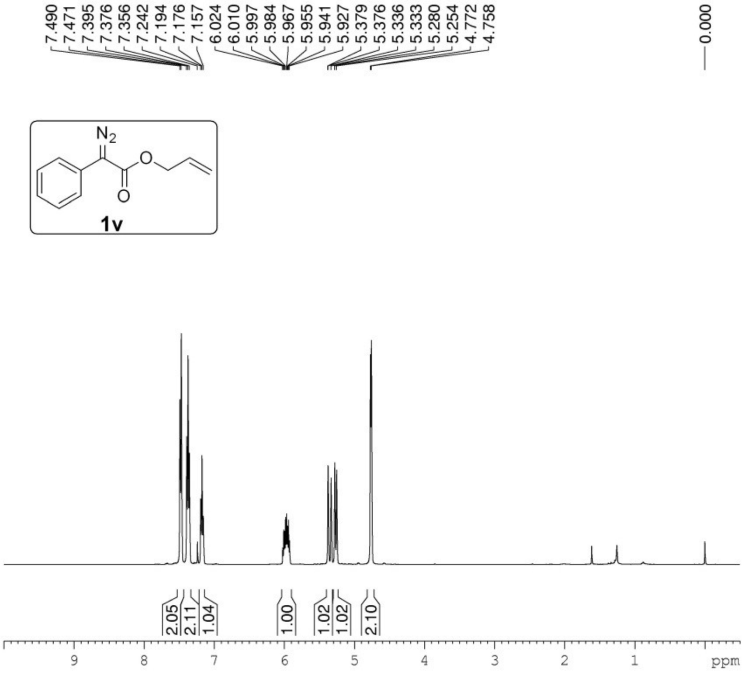

NAME 1v mzf153
EXPNO 1
PROCNO 1
Date_ 20141210
Time 19.44
INSTRUM spect
PROBHD 5 mm PABBO BB/
PULPROG zg30
TD 65536
SOLVENT CDCl3
NS 16
DS 2
SWH 8012.820 Hz
FIDRES 0.122266 Hz
AQ 4.0894966 sec
RG 31.52
DW 62.400 usec
DE 6.50 usec
TE 296.4 K
D1 1.00000000 sec
TD0 1

======== CHANNEL f1 ====
SFO1 400.1324710 MHz
NUC1 1H
P1 10.00 usec
SI 65536
SF 400.1300169 MHz
WDW EM
SSB 0
LB 0.30 Hz
GB 0
PC 1.00

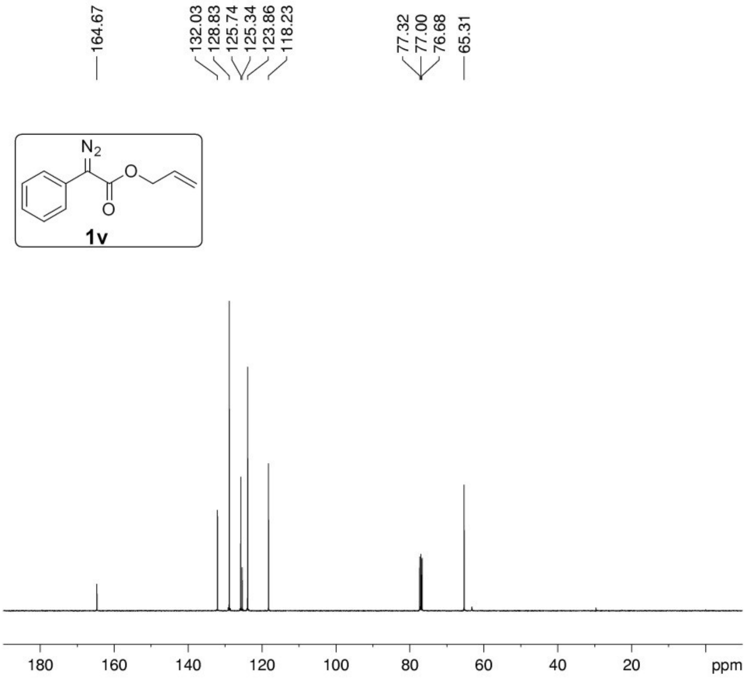

NAME 1v mzf153
EXPNO 2
PROCNO 1
Date_ 20141211
Time 1.04
INSTRUM spect
PROBHD 5 mm PABBO BB/
PULPROG zgpg30
TD 65536
SOLVENT CDCl3
NS 500
DS 4
SWH 24038.461 Hz
FIDRES 0.366798 Hz
AQ 1.3631988 sec
RG 196.87
DW 20.800 usec
DE 6.50 usec
TE 297.3 K
D1 2.00000000 sec
D11 0.03000000 sec
TD0 1

======== CHANNEL f1 =====
SFO1 100.6228293 MHz
NUC1 13C
P1 10.00 usec
SI 32768
SF 100.6127820 MHz
WDW EM
SSB 0
LB 1.00 Hz
GB 0
PC 1.40

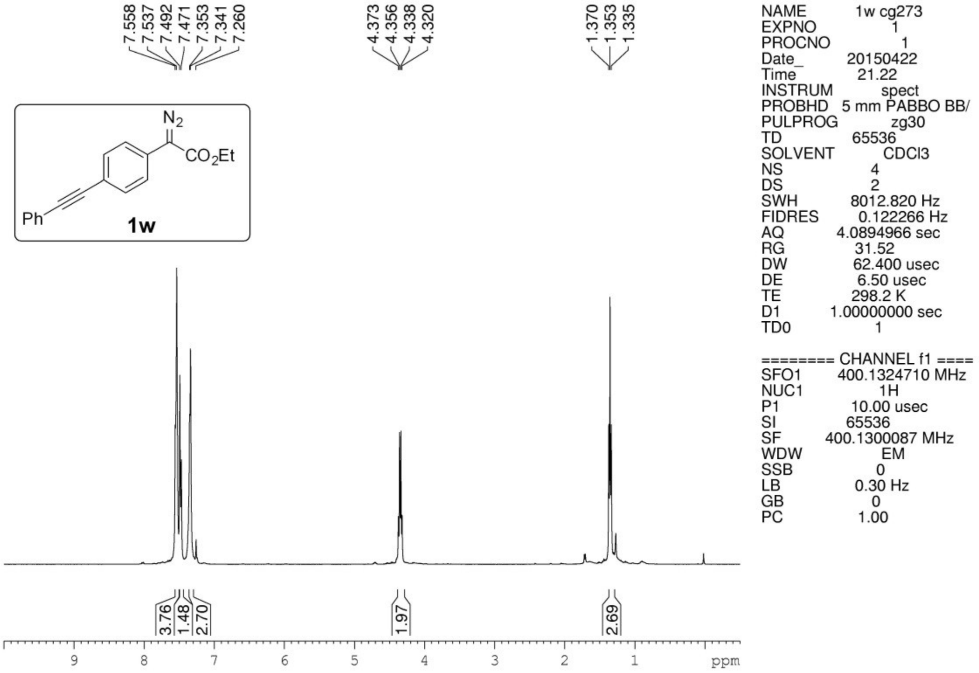
7.558
7.537
7.492
7.471
7.353
7.341
7.260
4.373
4.356
4.338
4.320
1.370
1.353
1.335
N2
CO2Et
Ph
1w
3.76
1.48
2.70
1.97
2.69
9 8 7 6 5 4 3 2 1 ppm
NAME 1w cg273
EXPNO 1
PROCNO 1
Date_ 20150422
Time 21.22
INSTRUM spect
PROBHD 5 mm PABBO BB/
PULPROG zg30
TD 65536
SOLVENT CDCl3
NS 4
DS 2
SWH 8012.820 Hz
FIDRES 0.122266 Hz
AQ 4.0894966 sec
RG 31.52
DW 62.400 usec
DE 6.50 usec
TE 298.2 K
D1 1.00000000 sec
TD0 1
======== CHANNEL f1 ====
SFO1 400.1324710 MHz
NUC1 1H
P1 10.00 usec
SI 65536
SF 400.1300087 MHz
WDW EM
SSB 0
LB 0.30 Hz
GB 0
PC 1.00

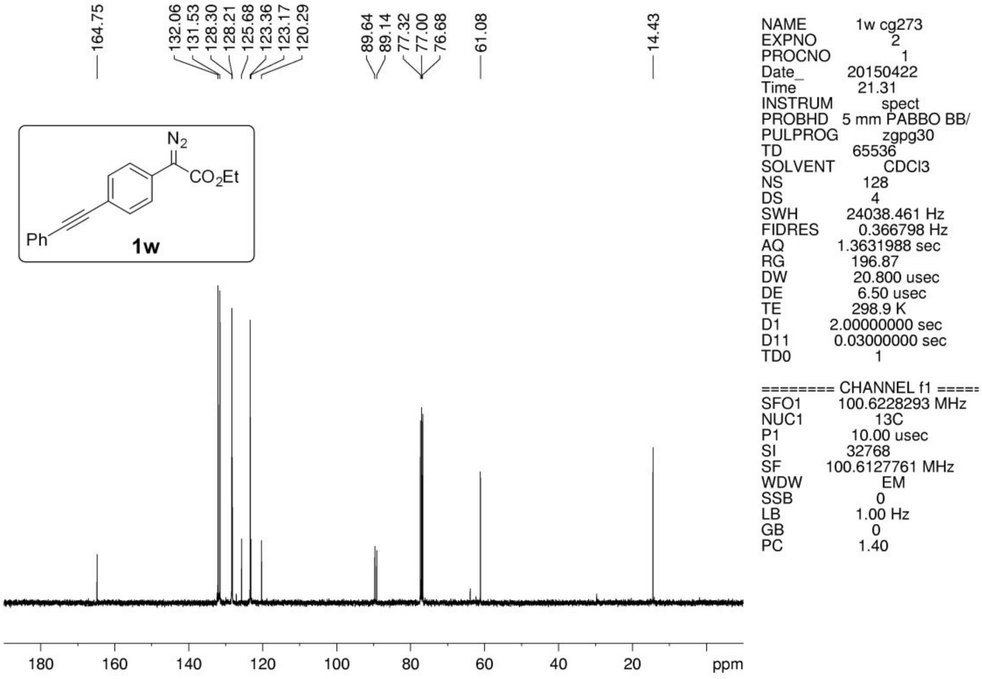
164.75
132.06
131.53
128.30
128.21
125.68
123.36
123.17
120.29
89.64
89.14
77.32
77.00
76.68
61.08
14.43
N2
CO2Et
Ph
1w
180 160 140 120 100 80 60 40 20 ppm
NAME 1w cg273
EXPNO 2
PROCNO 1
Date_ 20150422
Time 21.31
INSTRUM spect
PROBHD 5 mm PABBO BB/
PULPROG zgpg30
TD 65536
SOLVENT CDCl3
NS 128
DS 4
SWH 24038.461 Hz
FIDRES 0.366798 Hz
AQ 1.3631988 sec
RG 196.87
DW 20.800 usec
DE 6.50 usec
TE 298.9 K
D1 2.00000000 sec
D11 0.03000000 sec
TD0 1
======== CHANNEL f1 =====
SFO1 100.6228293 MHz
NUC1 13C
P1 10.00 usec
SI 32768
SF 100.6127761 MHz
WDW EM
SSB 0
LB 1.00 Hz
GB 0
PC 1.40

^{1}H, ^{13}C and ^{19}F NMR spectra for the prepared products

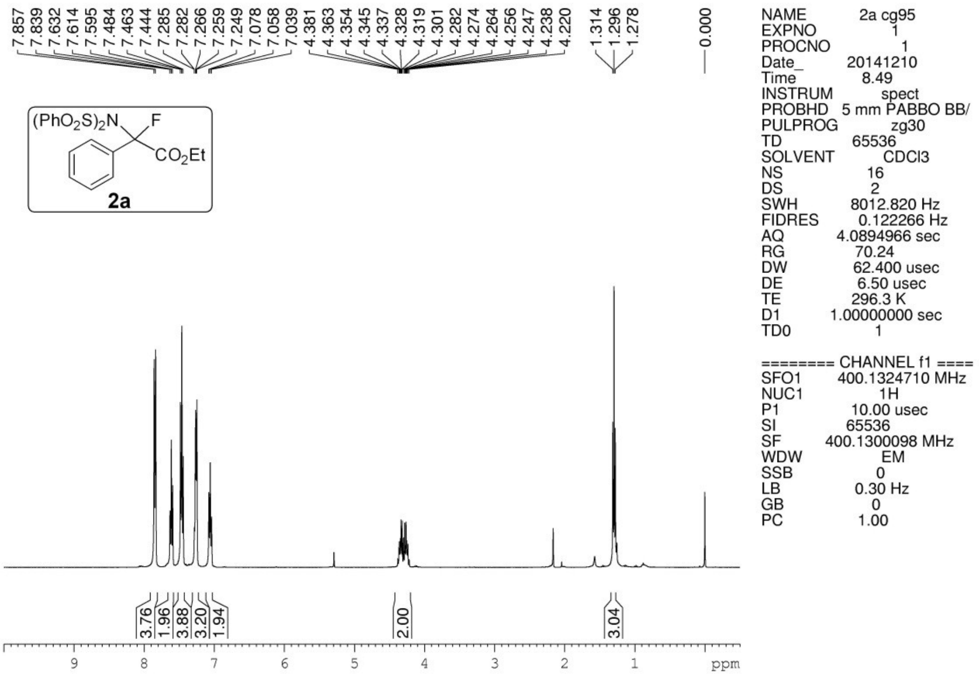

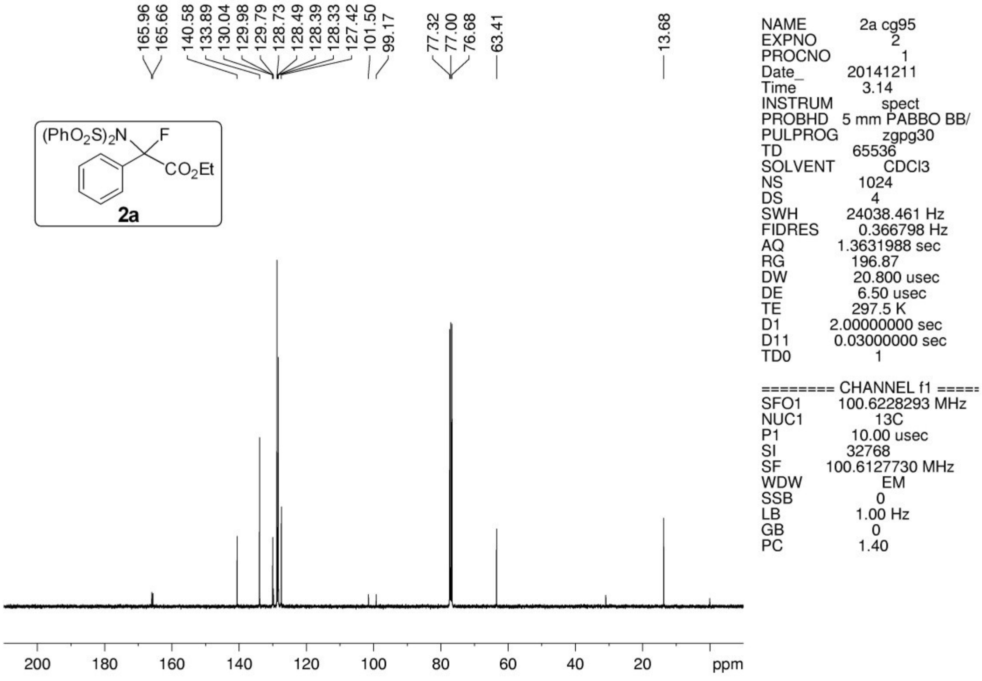

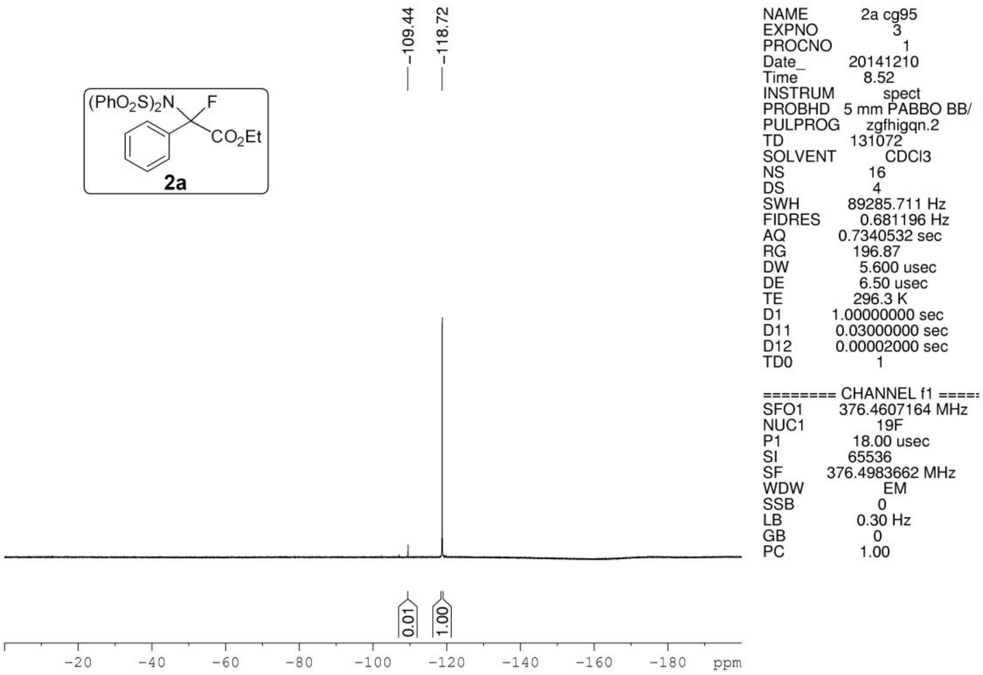

−109.44
−118.72
(PhO2S)2N F CO2Et
2a
0.01
1.00
−20 −40 −60 −80 −100 −120 −140 −160 −180 ppm
NAME 2a cg95
EXPNO 3
PROCNO 1
Date_ 20141210
Time 8.52
INSTRUM spect
PROBHD 5 mm PABBO BB/
PULPROG zgfhigqn.2
TD 131072
SOLVENT CDCl3
NS 16
DS 4
SWH 89285.711 Hz
FIDRES 0.681196 Hz
AQ 0.7340532 sec
RG 196.87
DW 5.600 usec
DE 6.50 usec
TE 296.3 K
D1 1.00000000 sec
D11 0.03000000 sec
D12 0.00002000 sec
TD0 1
======== CHANNEL f1 ====
SFO1 376.4607164 MHz
NUC1 19F
P1 18.00 usec
SI 65536
SF 376.4983662 MHz
WDW EM
SSB 0
LB 0.30 Hz
GB 0
PC 1.00

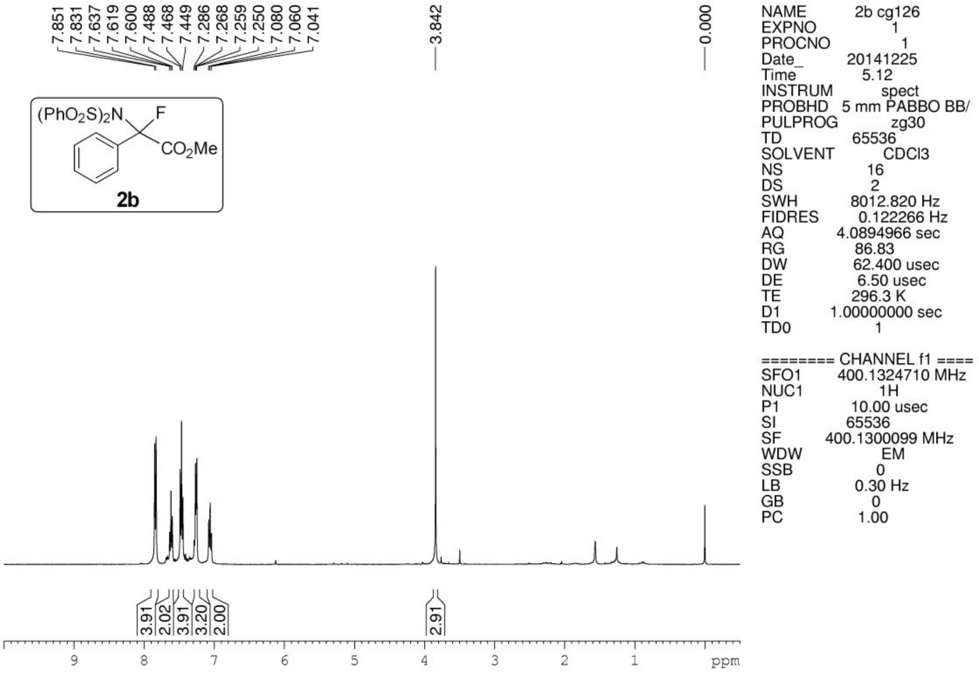

7.851 7.831 7.637 7.619 7.600 7.488 7.468 7.449 7.286 7.268 7.259 7.250 7.080 7.060 7.041
3.842
0.000
(PhO2S)2N F CO2Me
2b
3.91 2.02 3.91 3.20 2.00
2.91
9 8 7 6 5 4 3 2 1 ppm
NAME 2b cg126
EXPNO 1
PROCNO 1
Date_ 20141225
Time 5.12
INSTRUM spect
PROBHD 5 mm PABBO BB/
PULPROG zg30
TD 65536
SOLVENT CDCl3
NS 16
DS 2
SWH 8012.820 Hz
FIDRES 0.122266 Hz
AQ 4.0894966 sec
RG 86.83
DW 62.400 usec
DE 6.50 usec
TE 296.3 K
D1 1.00000000 sec
TD0 1
======== CHANNEL f1 ====
SFO1 400.1324710 MHz
NUC1 1H
P1 10.00 usec
SI 65536
SF 400.1300099 MHz
WDW EM
SSB 0
LB 0.30 Hz
GB 0
PC 1.00

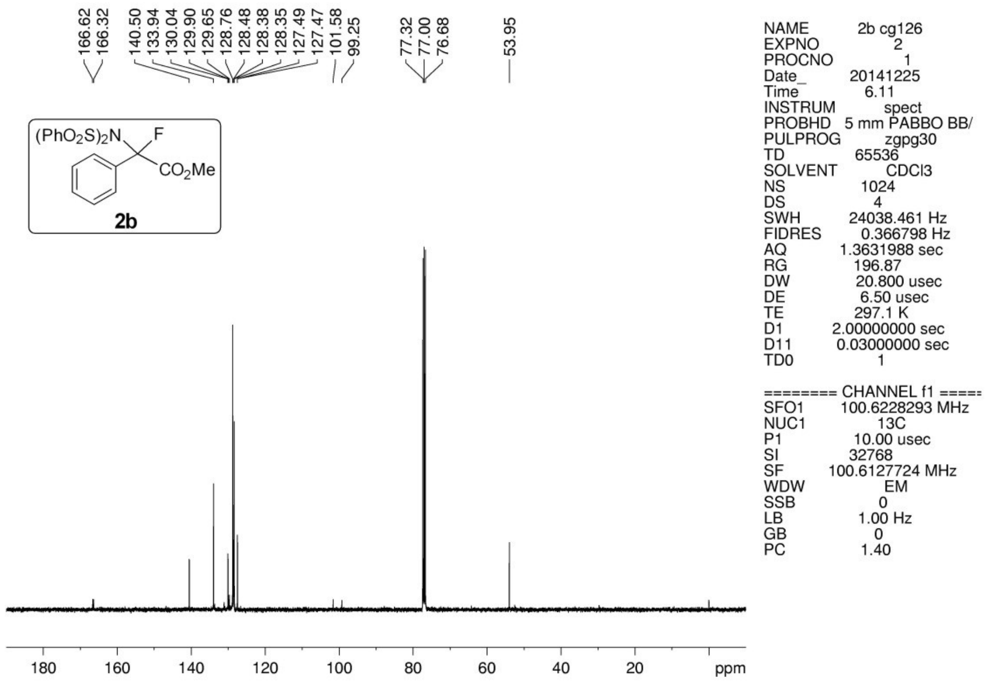
166.62
166.32
140.50
133.94
130.04
129.90
129.65
128.76
128.48
128.38
128.35
127.49
127.47
101.58
99.25
77.32
77.00
76.68
53.95
(PhO2S)2N F CO2Me
2b
180 160 140 120 100 80 60 40 20 ppm
NAME 2b cg126
EXPNO 2
PROCNO 1
Date_ 20141225
Time 6.11
INSTRUM spect
PROBHD 5 mm PABBO BB/
PULPROG zgpg30
TD 65536
SOLVENT CDCl3
NS 1024
DS 4
SWH 24038.461 Hz
FIDRES 0.366798 Hz
AQ 1.3631988 sec
RG 196.87
DW 20.800 usec
DE 6.50 usec
TE 297.1 K
D1 2.00000000 sec
D11 0.03000000 sec
TD0 1
======== CHANNEL f1 ========
SFO1 100.6228293 MHz
NUC1 13C
P1 10.00 usec
SI 32768
SF 100.6127724 MHz
WDW EM
SSB 0
LB 1.00 Hz
GB 0
PC 1.40

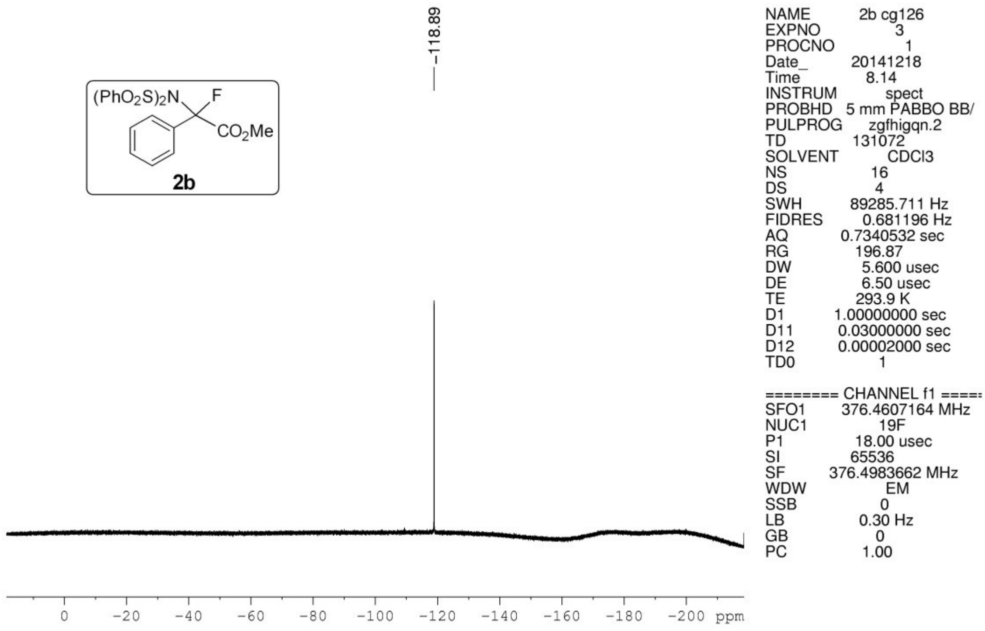
-118.89
(PhO2S)2N F CO2Me
2b
0 -20 -40 -60 -80 -100 -120 -140 -160 -180 -200 ppm
NAME 2b cg126
EXPNO 3
PROCNO 1
Date_ 20141218
Time 8.14
INSTRUM spect
PROBHD 5 mm PABBO BB/
PULPROG zgfhigqn.2
TD 131072
SOLVENT CDCl3
NS 16
DS 4
SWH 89285.711 Hz
FIDRES 0.681196 Hz
AQ 0.7340532 sec
RG 196.87
DW 5.600 usec
DE 6.50 usec
TE 293.9 K
D1 1.00000000 sec
D11 0.03000000 sec
D12 0.00002000 sec
TD0 1
======== CHANNEL f1 ========
SFO1 376.4607164 MHz
NUC1 19F
P1 18.00 usec
SI 65536
SF 376.4983662 MHz
WDW EM
SSB 0
LB 0.30 Hz
GB 0
PC 1.00

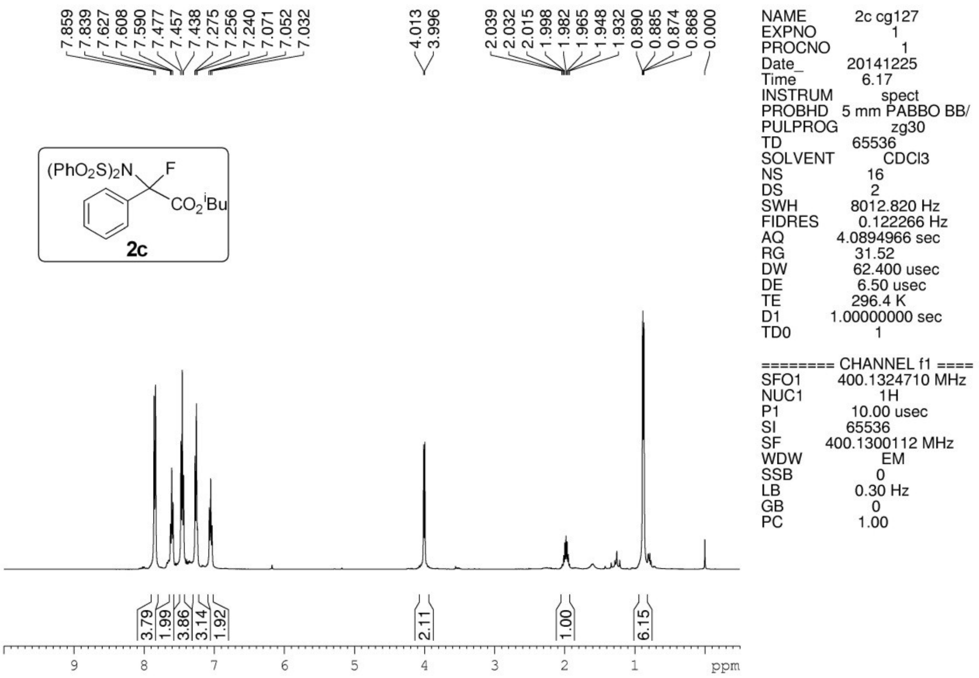
(PhO2S)2N F
CO2iBu
2c
NAME 2c cg127
EXPNO 1
PROCNO 1
Date_ 20141225
Time 6.17
INSTRUM spect
PROBHD 5 mm PABBO BB/
PULPROG zg30
TD 65536
SOLVENT CDCl3
NS 16
DS 2
SWH 8012.820 Hz
FIDRES 0.122266 Hz
AQ 4.0894966 sec
RG 31.52
DW 62.400 usec
DE 6.50 usec
TE 296.4 K
D1 1.00000000 sec
TD0 1
======== CHANNEL f1 ====
SFO1 400.1324710 MHz
NUC1 1H
P1 10.00 usec
SI 65536
SF 400.1300112 MHz
WDW EM
SSB 0
LB 0.30 Hz
GB 0
PC 1.00

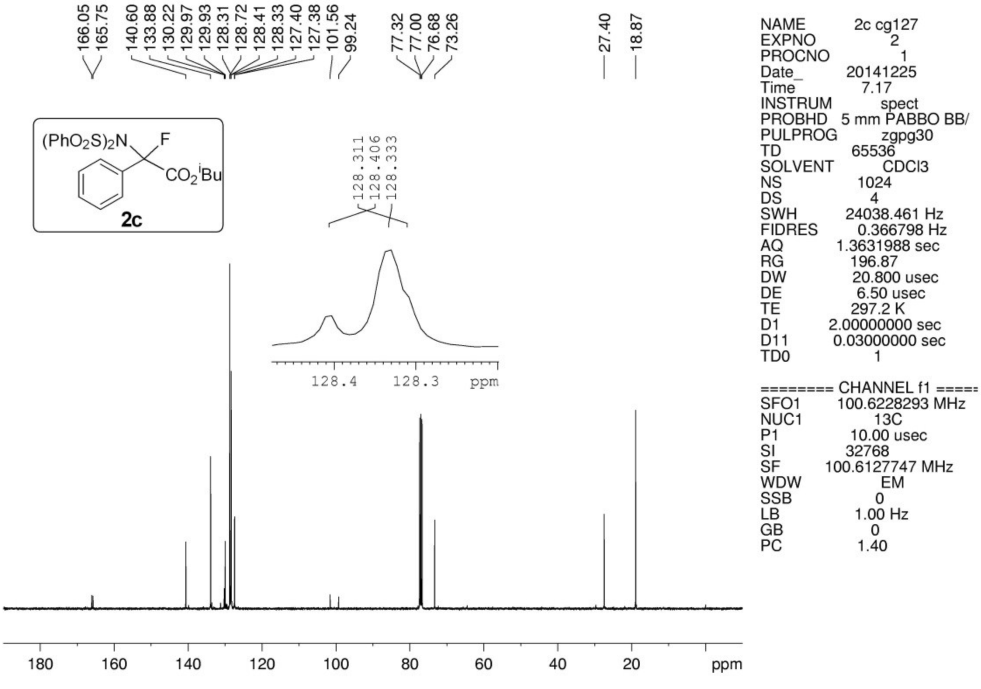
(PhO2S)2N F
CO2iBu
2c
NAME 2c cg127
EXPNO 2
PROCNO 1
Date_ 20141225
Time 7.17
INSTRUM spect
PROBHD 5 mm PABBO BB/
PULPROG zgpg30
TD 65536
SOLVENT CDCl3
NS 1024
DS 4
SWH 24038.461 Hz
FIDRES 0.366798 Hz
AQ 1.3631988 sec
RG 196.87
DW 20.800 usec
DE 6.50 usec
TE 297.2 K
D1 2.00000000 sec
D11 0.03000000 sec
TD0 1
======== CHANNEL f1 =====
SFO1 100.6228293 MHz
NUC1 13C
P1 10.00 usec
SI 32768
SF 100.6127747 MHz
WDW EM
SSB 0
LB 1.00 Hz
GB 0
PC 1.40

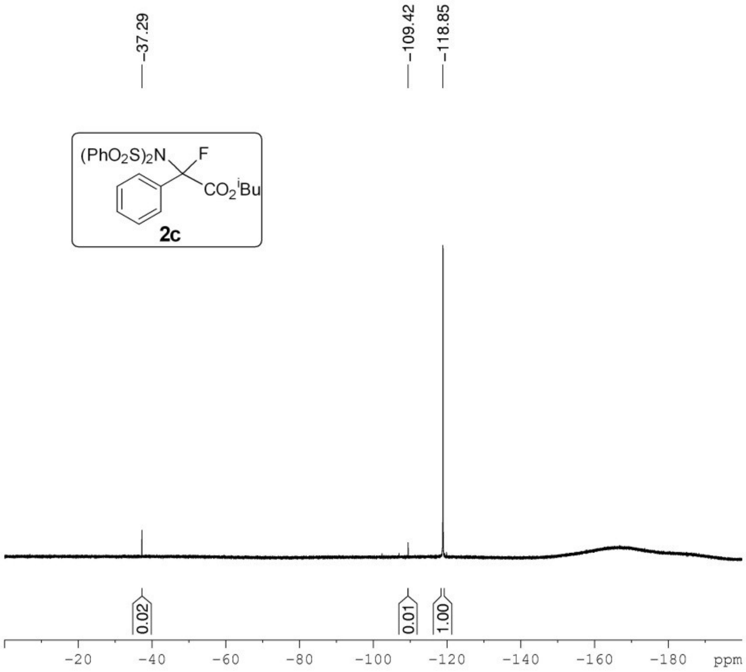

NAME 2c cg127
EXPNO 3
PROCNO 1
Date_ 20141218
Time 8.21
INSTRUM spect
PROBHD 5 mm PABBO BB/
PULPROG zgfhigqn.2
TD 131072
SOLVENT CDCl3
NS 16
DS 4
SWH 89285.711 Hz
FIDRES 0.681196 Hz
AQ 0.7340532 sec
RG 196.87
DW 5.600 usec
DE 6.50 usec
TE 293.6 K
D1 1.00000000 sec
D11 0.03000000 sec
D12 0.00002000 sec
TD0 1

======== CHANNEL f1 ====
SFO1 376.4607164 MHz
NUC1 19F
P1 18.00 usec
SI 65536
SF 376.4983662 MHz
WDW EM
SSB 0
LB 0.30 Hz
GB 0
PC 1.00

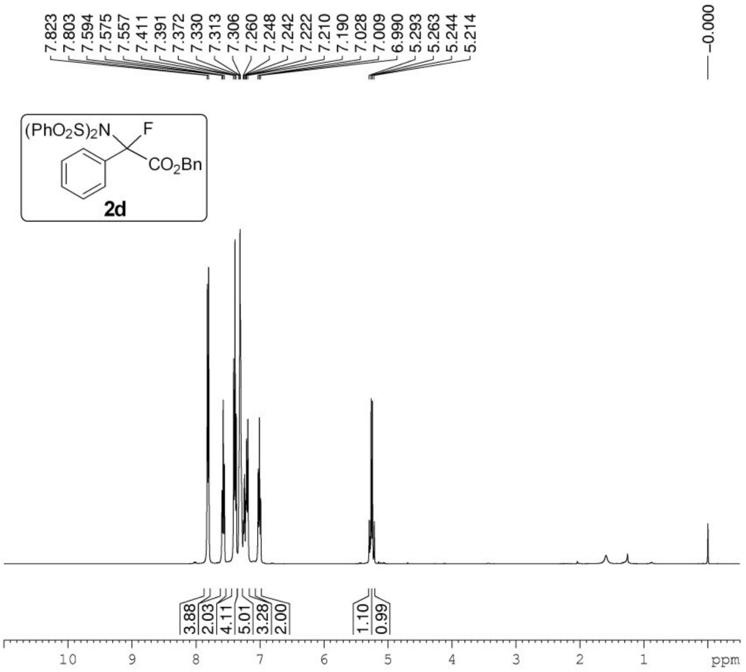

NAME 2d cg129
EXPNO 1
PROCNO 1
Date_ 20150104
Time 22.07
INSTRUM spect
PROBHD 5 mm PABBO BB/
PULPROG zg30
TD 65536
SOLVENT CDCl3
NS 16
DS 2
SWH 8012.820 Hz
FIDRES 0.122266 Hz
AQ 4.0894966 sec
RG 31.52
DW 62.400 usec
DE 6.50 usec
TE 293.6 K
D1 1.00000000 sec
TD0 1

======== CHANNEL f1 ====
SFO1 400.1324710 MHz
NUC1 1H
P1 10.00 usec
SI 65536
SF 400.1300136 MHz
WDW EM
SSB 0
LB 0.30 Hz
GB 0
PC 1.00

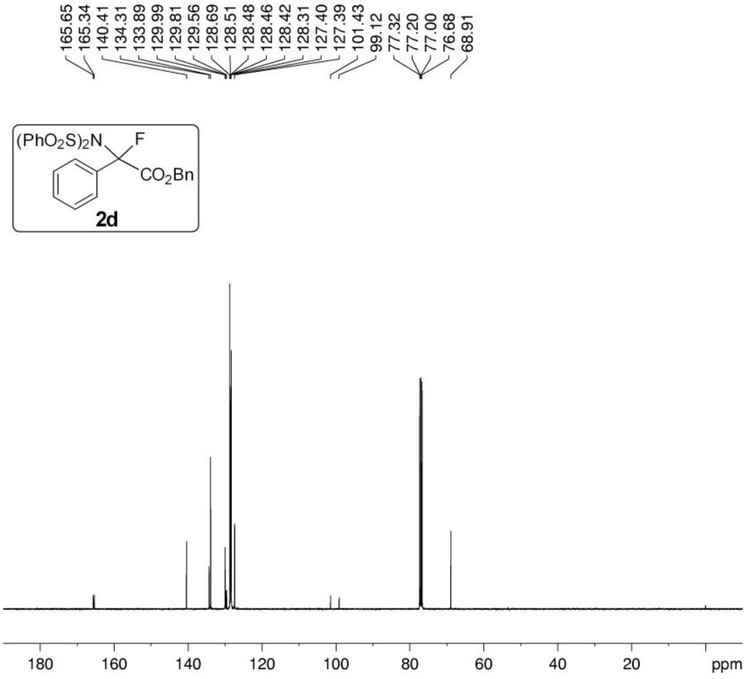

NAME 2d cg129
EXPNO 2
PROCNO 1
Date_ 20150105
Time 0.03
INSTRUM spect
PROBHD 5 mm PABBO BB/
PULPROG zgpg30
TD 65536
SOLVENT CDCl3
NS 2000
DS 4
SWH 24038.461 Hz
FIDRES 0.366798 Hz
AQ 1.3631988 sec
RG 196.87
DW 20.800 usec
DE 6.50 usec
TE 294.7 K
D1 2.00000000 sec
D11 0.03000000 sec
TD0 1

======== CHANNEL f1 =====
SFO1 100.6228293 MHz
NUC1 13C
P1 10.00 usec
SI 32768
SF 100.6127761 MHz
WDW EM
SSB 0
LB 1.00 Hz
GB 0
PC 1.40

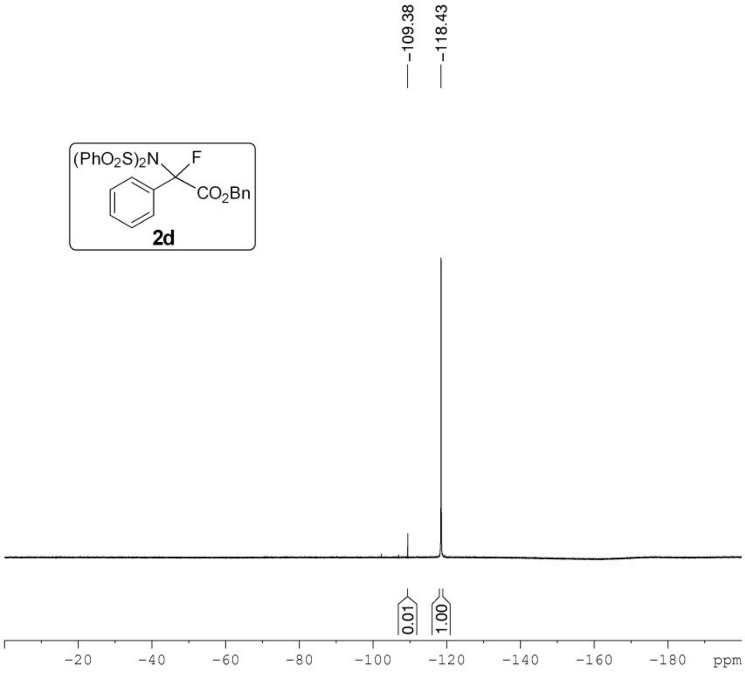

NAME 2d cg129
EXPNO 3
PROCNO 1
Date_ 20150609
Time 4.43
INSTRUM spect
PROBHD 5 mm PABBO BB/
PULPROG zgfhigqn.2
TD 131072
SOLVENT CDCl3
NS 16
DS 4
SWH 89285.711 Hz
FIDRES 0.681196 Hz
AQ 0.7340532 sec
RG 196.87
DW 5.600 usec
DE 6.50 usec
TE 297.8 K
D1 1.00000000 sec
D11 0.03000000 sec
D12 0.00002000 sec
TD0 1

======== CHANNEL f1 =====
SFO1 376.4607164 MHz
NUC1 19F
P1 18.00 usec
SI 65536
SF 376.4983662 MHz
WDW EM
SSB 0
LB 0.30 Hz
GB 0
PC 1.00

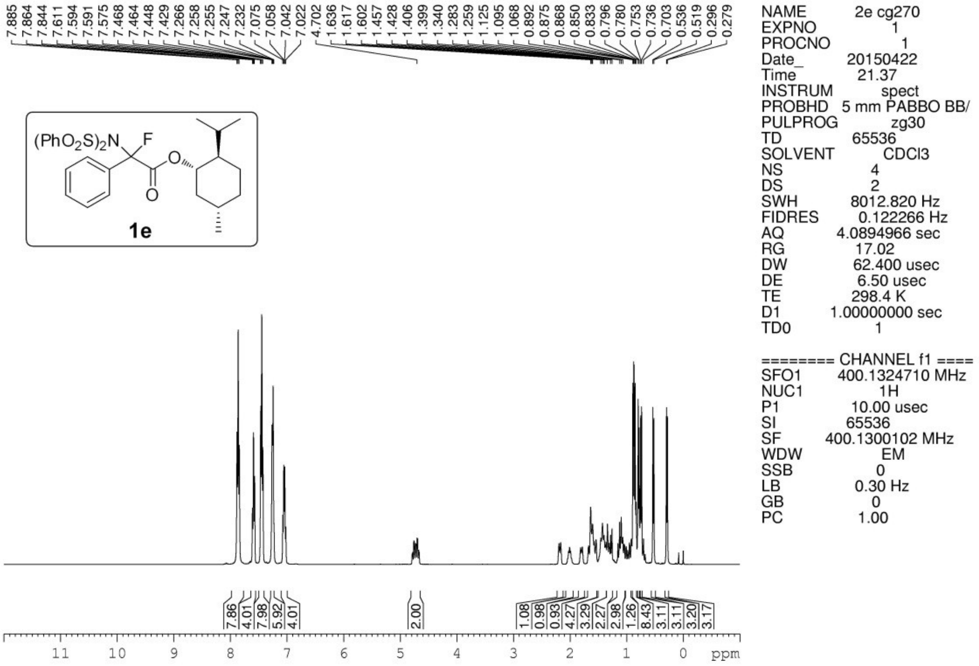
7.885 7.864 7.844 7.611 7.594 7.591 7.575 7.468 7.464 7.448 7.429 7.266 7.258 7.255 7.247 7.232 7.075 7.058 7.042 7.022 4.702 1.636 1.617 1.602 1.457 1.428 1.406 1.399 1.340 1.283 1.259 1.125 1.095 1.068 0.892 0.875 0.868 0.850 0.833 0.796 0.780 0.753 0.736 0.703 0.536 0.519 0.296 0.279
(PhO2S)2N F
O
1e
NAME 2e cg270
EXPNO 1
PROCNO 1
Date_ 20150422
Time 21.37
INSTRUM spect
PROBHD 5 mm PABBO BB/
PULPROG zg30
TD 65536
SOLVENT CDCl3
NS 4
DS 2
SWH 8012.820 Hz
FIDRES 0.122266 Hz
AQ 4.0894966 sec
RG 17.02
DW 62.400 usec
DE 6.50 usec
TE 298.4 K
D1 1.00000000 sec
TD0 1
======== CHANNEL f1 ====
SFO1 400.1324710 MHz
NUC1 1H
P1 10.00 usec
SI 65536
SF 400.1300102 MHz
WDW EM
SSB 0
LB 0.30 Hz
GB 0
PC 1.00
7.86 4.01 7.98 5.92 4.01
2.00
1.08 0.98 0.93 4.27 3.29 2.27 2.98 1.26 8.43 3.11 3.20 3.17
11 10 9 8 7 6 5 4 3 2 1 0 ppm

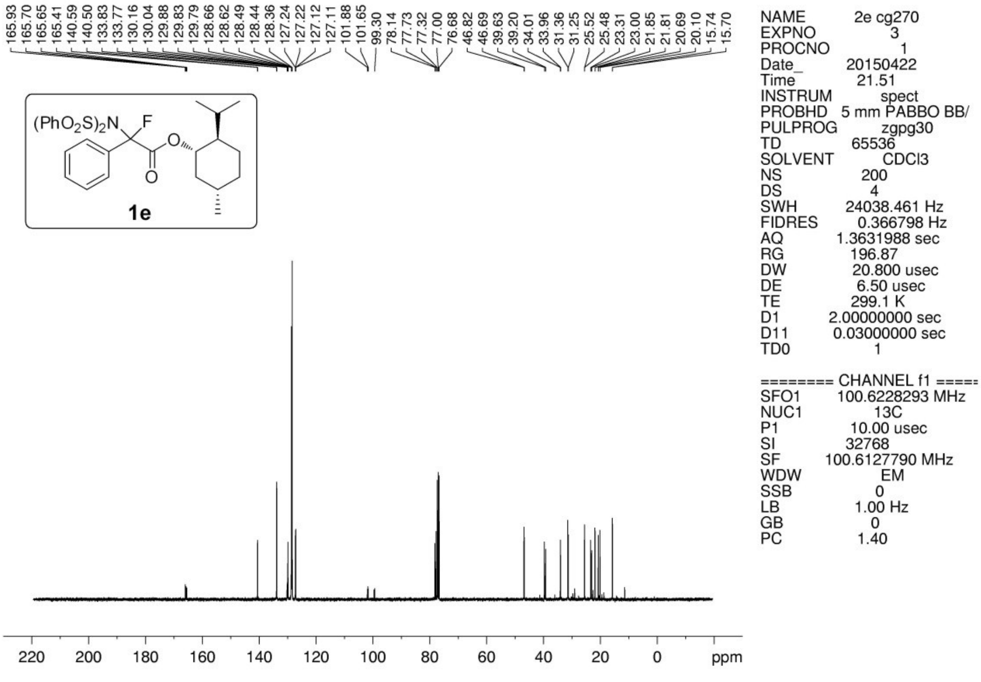
165.93 165.70 165.65 165.41 140.59 140.50 133.83 133.77 130.16 130.04 129.88 129.83 129.79 128.66 128.62 128.49 128.44 128.36 127.24 127.22 127.12 127.11 101.88 101.65 99.30 78.14 77.73 77.32 77.00 76.68 46.82 46.69 39.63 39.20 34.01 33.96 31.36 31.25 25.52 25.48 23.31 23.00 21.85 21.81 20.69 20.10 15.74 15.70
(PhO2S)2N F
O
1e
NAME 2e cg270
EXPNO 3
PROCNO 1
Date_ 20150422
Time 21.51
INSTRUM spect
PROBHD 5 mm PABBO BB/
PULPROG zgpg30
TD 65536
SOLVENT CDCl3
NS 200
DS 4
SWH 24038.461 Hz
FIDRES 0.366798 Hz
AQ 1.3631988 sec
RG 196.87
DW 20.800 usec
DE 6.50 usec
TE 299.1 K
D1 2.00000000 sec
D11 0.03000000 sec
TD0 1
======== CHANNEL f1 =====
SFO1 100.6228293 MHz
NUC1 13C
P1 10.00 usec
SI 32768
SF 100.6127790 MHz
WDW EM
SSB 0
LB 1.00 Hz
GB 0
PC 1.40
220 200 180 160 140 120 100 80 60 40 20 0 ppm

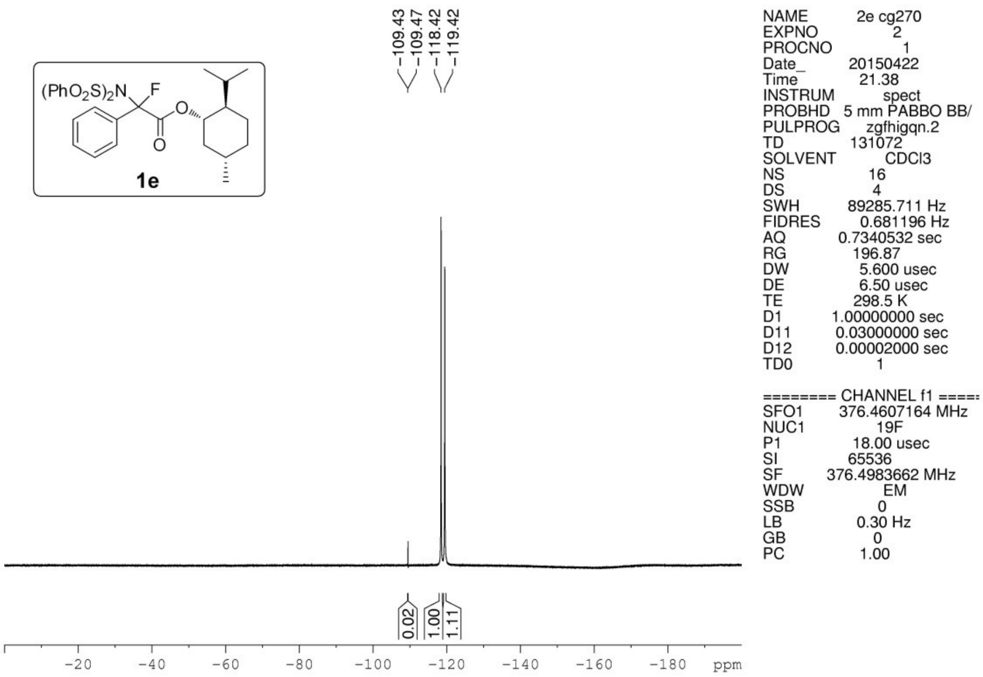

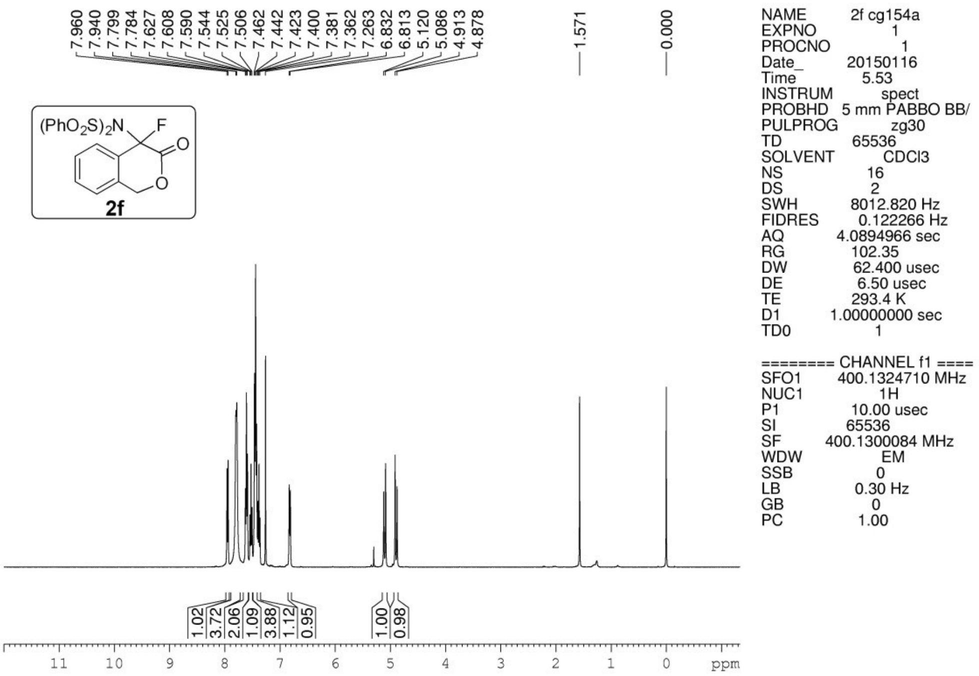

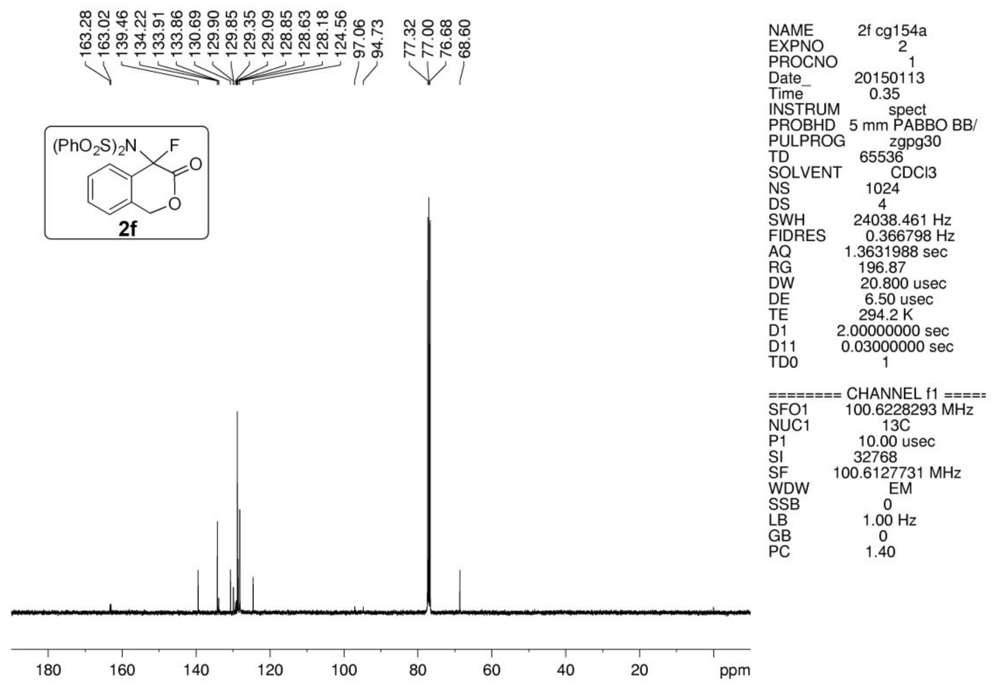
163.28
163.02
139.46
134.22
133.91
133.86
130.69
129.90
129.85
129.35
129.09
128.85
128.63
128.18
124.56
97.06
94.73
77.32
77.00
76.68
68.60
(PhO2S)2N
F
O
O
2f
180
160
140
120
100
80
60
40
20
ppm
NAME 2f cg154a
EXPNO 2
PROCNO 1
Date_ 20150113
Time 0.35
INSTRUM spect
PROBHD 5 mm PABBO BB/
PULPROG zgpg30
TD 65536
SOLVENT CDCl3
NS 1024
DS 4
SWH 24038.461 Hz
FIDRES 0.366798 Hz
AQ 1.3631988 sec
RG 196.87
DW 20.800 usec
DE 6.50 usec
TE 294.2 K
D1 2.00000000 sec
D11 0.03000000 sec
TD0 1
======== CHANNEL f1 ========
SFO1 100.6228293 MHz
NUC1 13C
P1 10.00 usec
SI 32768
SF 100.6127731 MHz
WDW EM
SSB 0
LB 1.00 Hz
GB 0
PC 1.40

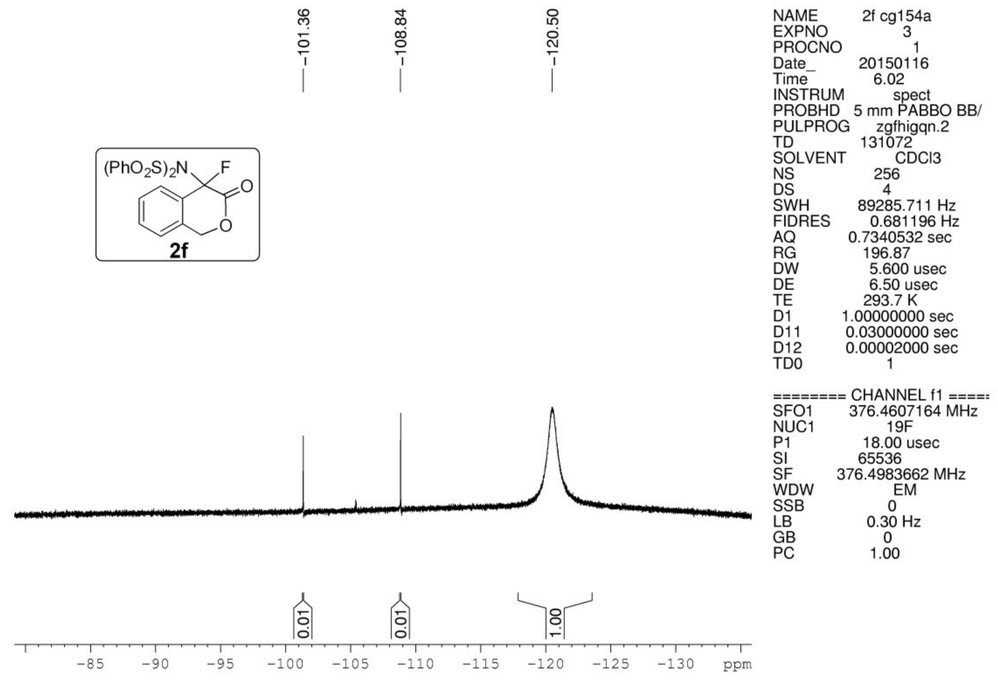
-101.36
-108.84
-120.50
(PhO2S)2N
F
O
O
2f
0.01
0.01
1.00
-85
-90
-95
-100
-105
-110
-115
-120
-125
-130
ppm
NAME 2f cg154a
EXPNO 3
PROCNO 1
Date_ 20150116
Time 6.02
INSTRUM spect
PROBHD 5 mm PABBO BB/
PULPROG zgfhigqn.2
TD 131072
SOLVENT CDCl3
NS 256
DS 4
SWH 89285.711 Hz
FIDRES 0.681196 Hz
AQ 0.7340532 sec
RG 196.87
DW 5.600 usec
DE 6.50 usec
TE 293.7 K
D1 1.00000000 sec
D11 0.03000000 sec
D12 0.00002000 sec
TD0 1
======== CHANNEL f1 ========
SFO1 376.4607164 MHz
NUC1 19F
P1 18.00 usec
SI 65536
SF 376.4983662 MHz
WDW EM
SSB 0
LB 0.30 Hz
GB 0
PC 1.00

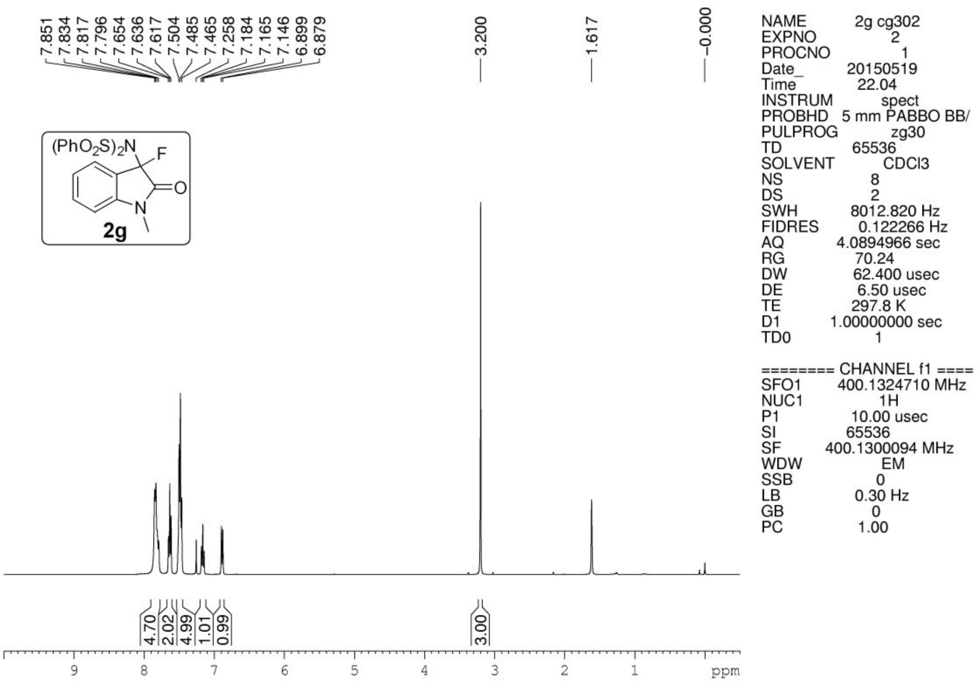
7.851
7.834
7.817
7.796
7.654
7.636
7.617
7.504
7.485
7.465
7.258
7.184
7.165
7.146
6.899
6.879
3.200
1.617
-0.000
(PhO2S)2N
F
O
N
2g
4.70
2.02
4.99
1.01
0.99
3.00
9
8
7
6
5
4
3
2
1
ppm
NAME 2g cg302
EXPNO 2
PROCNO 1
Date_ 20150519
Time 22.04
INSTRUM spect
PROBHD 5 mm PABBO BB/
PULPROG zg30
TD 65536
SOLVENT CDCl3
NS 8
DS 2
SWH 8012.820 Hz
FIDRES 0.122266 Hz
AQ 4.0894966 sec
RG 70.24
DW 62.400 usec
DE 6.50 usec
TE 297.8 K
D1 1.00000000 sec
TD0 1
======== CHANNEL f1 ====
SFO1 400.1324710 MHz
NUC1 1H
P1 10.00 usec
SI 65536
SF 400.1300094 MHz
WDW EM
SSB 0
LB 0.30 Hz
GB 0
PC 1.00

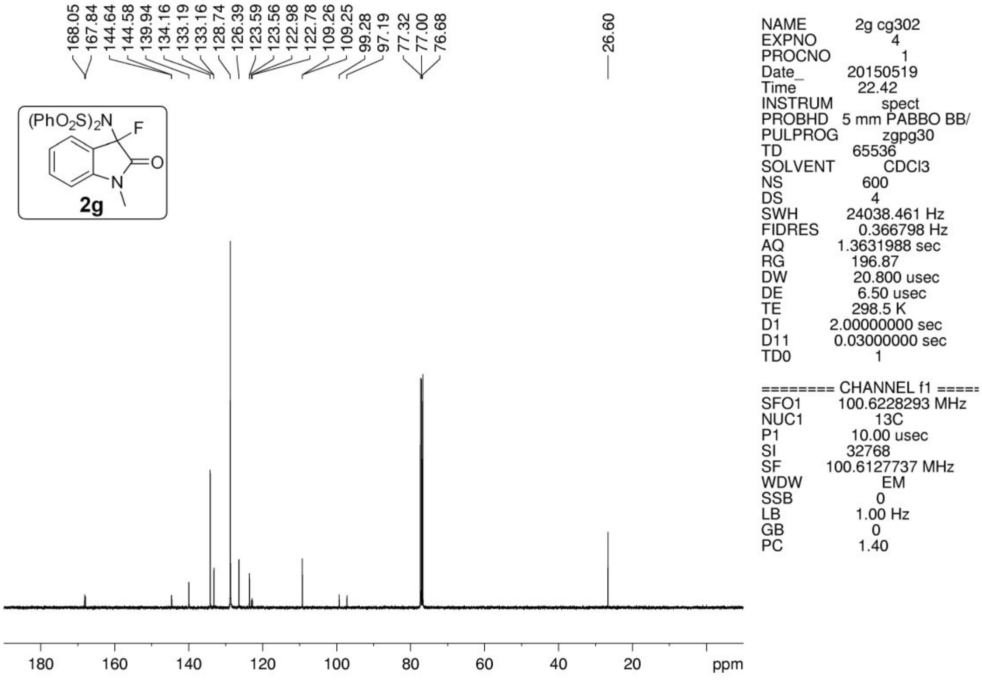
168.05
167.84
144.64
144.58
139.94
134.16
133.19
133.16
128.74
126.39
123.59
123.56
122.98
122.78
109.26
109.25
99.28
97.19
77.32
77.00
76.68
26.60
(PhO2S)2N
F
O
N
2g
180
160
140
120
100
80
60
40
20
ppm
NAME 2g cg302
EXPNO 4
PROCNO 1
Date_ 20150519
Time 22.42
INSTRUM spect
PROBHD 5 mm PABBO BB/
PULPROG zgpg30
TD 65536
SOLVENT CDCl3
NS 600
DS 4
SWH 24038.461 Hz
FIDRES 0.366798 Hz
AQ 1.3631988 sec
RG 196.87
DW 20.800 usec
DE 6.50 usec
TE 298.5 K
D1 2.00000000 sec
D11 0.03000000 sec
TD0 1
======== CHANNEL f1 =====
SFO1 100.6228293 MHz
NUC1 13C
P1 10.00 usec
SI 32768
SF 100.6127737 MHz
WDW EM
SSB 0
LB 1.00 Hz
GB 0
PC 1.40

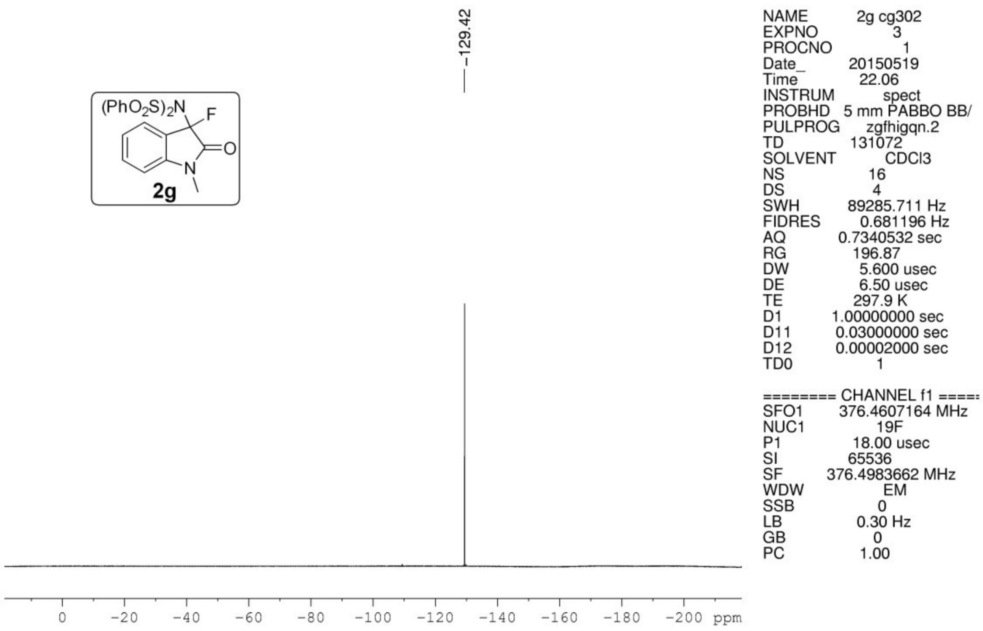

−129.42
(PhO2S)2N
F
O
N
2g
0
−20
−40
−60
−80
−100
−120
−140
−160
−180
−200 ppm
NAME 2g cg302
EXPNO 3
PROCNO 1
Date_ 20150519
Time 22.06
INSTRUM spect
PROBHD 5 mm PABBO BB/
PULPROG zgfhigqn.2
TD 131072
SOLVENT CDCl3
NS 16
DS 4
SWH 89285.711 Hz
FIDRES 0.681196 Hz
AQ 0.7340532 sec
RG 196.87
DW 5.600 usec
DE 6.50 usec
TE 297.9 K
D1 1.00000000 sec
D11 0.03000000 sec
D12 0.00002000 sec
TD0 1
======== CHANNEL f1 ====
SFO1 376.4607164 MHz
NUC1 19F
P1 18.00 usec
SI 65536
SF 376.4983662 MHz
WDW EM
SSB 0
LB 0.30 Hz
GB 0
PC 1.00

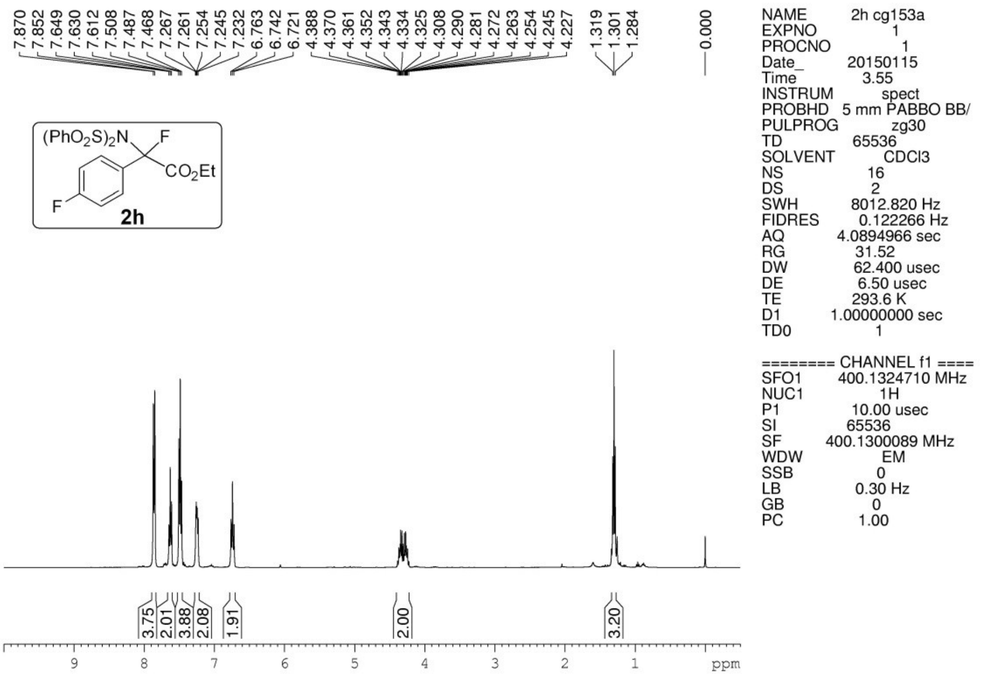

7.870 7.852 7.649 7.630 7.612 7.508 7.487 7.468 7.267 7.261 7.254 7.245 7.232 6.763 6.742 6.721 4.388 4.370 4.361 4.352 4.343 4.334 4.325 4.308 4.290 4.281 4.272 4.263 4.254 4.245 4.227 1.319 1.301 1.284 0.000
(PhO2S)2N
F
CO2Et
F
2h
3.75 2.01 3.88 2.08 1.91 2.00 3.20
9 8 7 6 5 4 3 2 1 ppm
NAME 2h cg153a
EXPNO 1
PROCNO 1
Date_ 20150115
Time 3.55
INSTRUM spect
PROBHD 5 mm PABBO BB/
PULPROG zg30
TD 65536
SOLVENT CDCl3
NS 16
DS 2
SWH 8012.820 Hz
FIDRES 0.122266 Hz
AQ 4.0894966 sec
RG 31.52
DW 62.400 usec
DE 6.50 usec
TE 293.6 K
D1 1.00000000 sec
TD0 1
======== CHANNEL f1 ====
SFO1 400.1324710 MHz
NUC1 1H
P1 10.00 usec
SI 65536
SF 400.1300089 MHz
WDW EM
SSB 0
LB 0.30 Hz
GB 0
PC 1.00

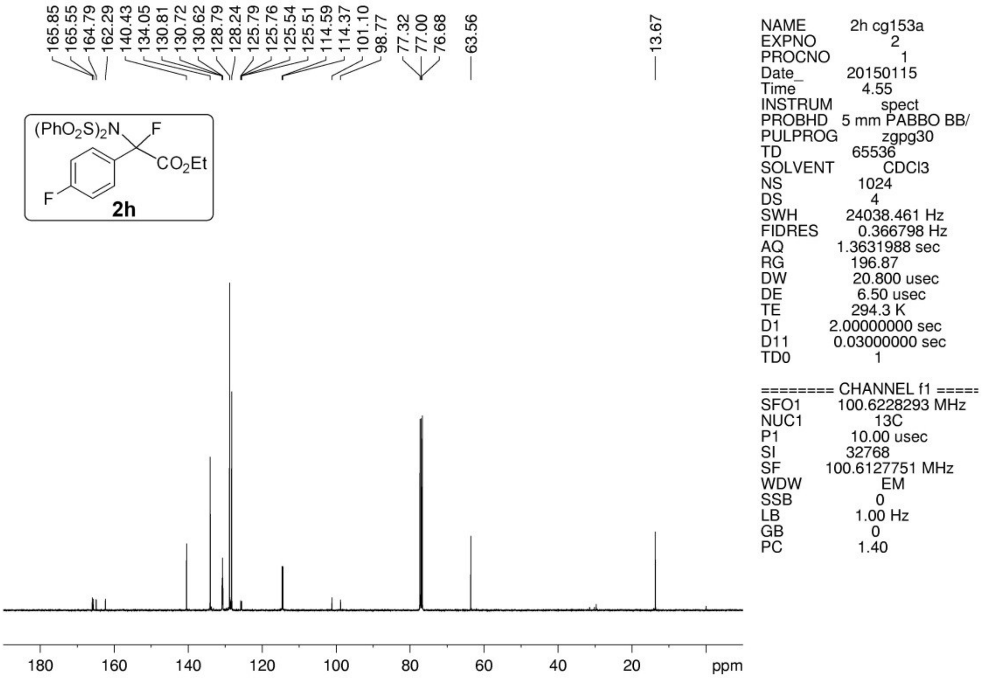
165.85
165.55
164.79
162.29
140.43
134.05
130.81
130.72
130.62
128.79
128.24
125.79
125.76
125.54
125.51
114.59
114.37
101.10
98.77
77.32
77.00
76.68
63.56
13.67
(PhO2S)2N F
CO2Et
F
2h
180 160 140 120 100 80 60 40 20 ppm
NAME 2h cg153a
EXPNO 2
PROCNO 1
Date_ 20150115
Time 4.55
INSTRUM spect
PROBHD 5 mm PABBO BB/
PULPROG zgpg30
TD 65536
SOLVENT CDCl3
NS 1024
DS 4
SWH 24038.461 Hz
FIDRES 0.366798 Hz
AQ 1.3631988 sec
RG 196.87
DW 20.800 usec
DE 6.50 usec
TE 294.3 K
D1 2.00000000 sec
D11 0.03000000 sec
TD0 1
======== CHANNEL f1 ======
SFO1 100.6228293 MHz
NUC1 13C
P1 10.00 usec
SI 32768
SF 100.6127751 MHz
WDW EM
SSB 0
LB 1.00 Hz
GB 0
PC 1.40

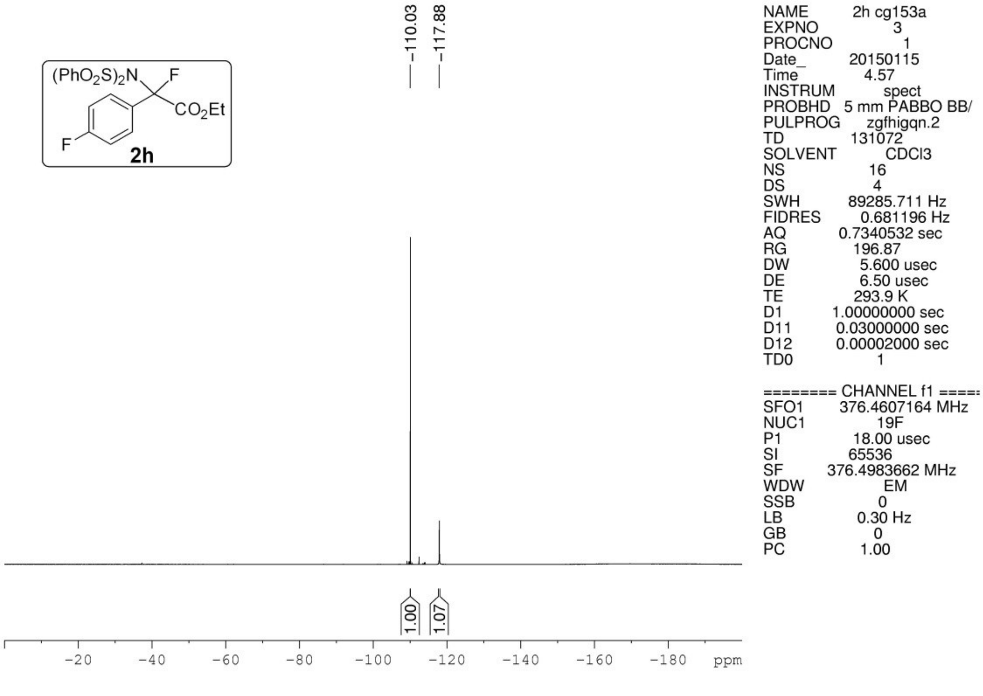
-110.03
-117.88
(PhO2S)2N F
CO2Et
F
2h
1.00
1.07
-20 -40 -60 -80 -100 -120 -140 -160 -180 ppm
NAME 2h cg153a
EXPNO 3
PROCNO 1
Date_ 20150115
Time 4.57
INSTRUM spect
PROBHD 5 mm PABBO BB/
PULPROG zgfhigqn.2
TD 131072
SOLVENT CDCl3
NS 16
DS 4
SWH 89285.711 Hz
FIDRES 0.681196 Hz
AQ 0.7340532 sec
RG 196.87
DW 5.600 usec
DE 6.50 usec
TE 293.9 K
D1 1.00000000 sec
D11 0.03000000 sec
D12 0.00002000 sec
TD0 1
======== CHANNEL f1 ======
SFO1 376.4607164 MHz
NUC1 19F
P1 18.00 usec
SI 65536
SF 376.4983662 MHz
WDW EM
SSB 0
LB 0.30 Hz
GB 0
PC 1.00

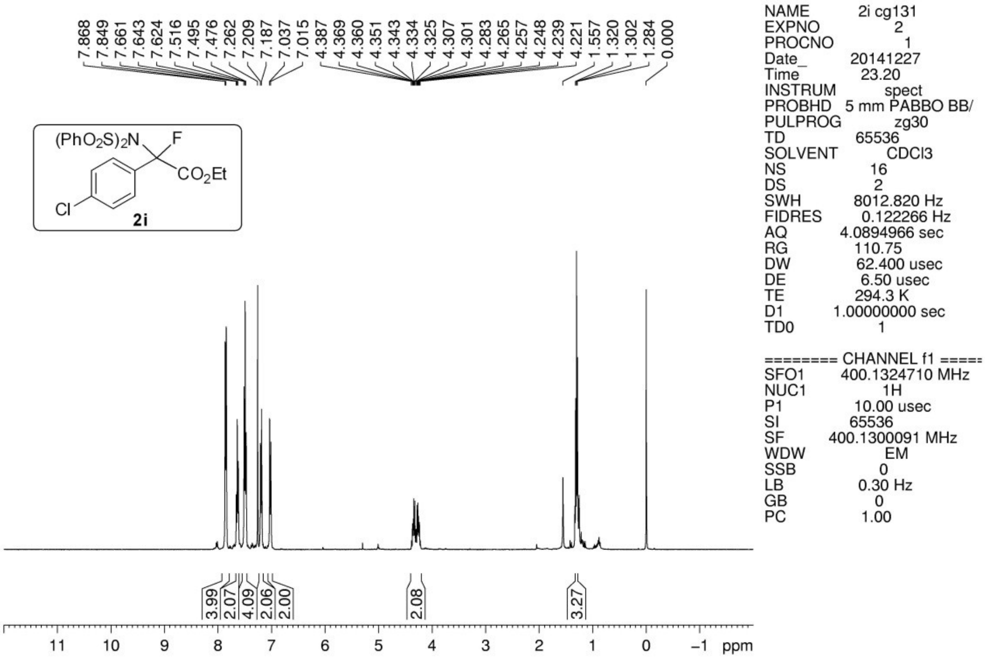

7.868 7.849 7.661 7.643 7.624 7.516 7.495 7.476 7.262 7.209 7.187 7.037 7.015 4.387 4.369 4.360 4.351 4.343 4.334 4.325 4.307 4.301 4.283 4.265 4.257 4.248 4.239 4.221 1.557 1.320 1.302 1.284 0.000
(PhO2S)2N
F
CO2Et
Cl
2i
3.99 2.07 4.09 2.06 2.00 2.08 3.27
11 10 9 8 7 6 5 4 3 2 1 0 –1 ppm
NAME 2i cg131
EXPNO 2
PROCNO 1
Date_ 20141227
Time 23.20
INSTRUM spect
PROBHD 5 mm PABBO BB/
PULPROG zg30
TD 65536
SOLVENT CDCl3
NS 16
DS 2
SWH 8012.820 Hz
FIDRES 0.122266 Hz
AQ 4.0894966 sec
RG 110.75
DW 62.400 usec
DE 6.50 usec
TE 294.3 K
D1 1.00000000 sec
TD0 1
======== CHANNEL f1 ========
SFO1 400.1324710 MHz
NUC1 1H
P1 10.00 usec
SI 65536
SF 400.1300091 MHz
WDW EM
SSB 0
LB 0.30 Hz
GB 0
PC 1.00

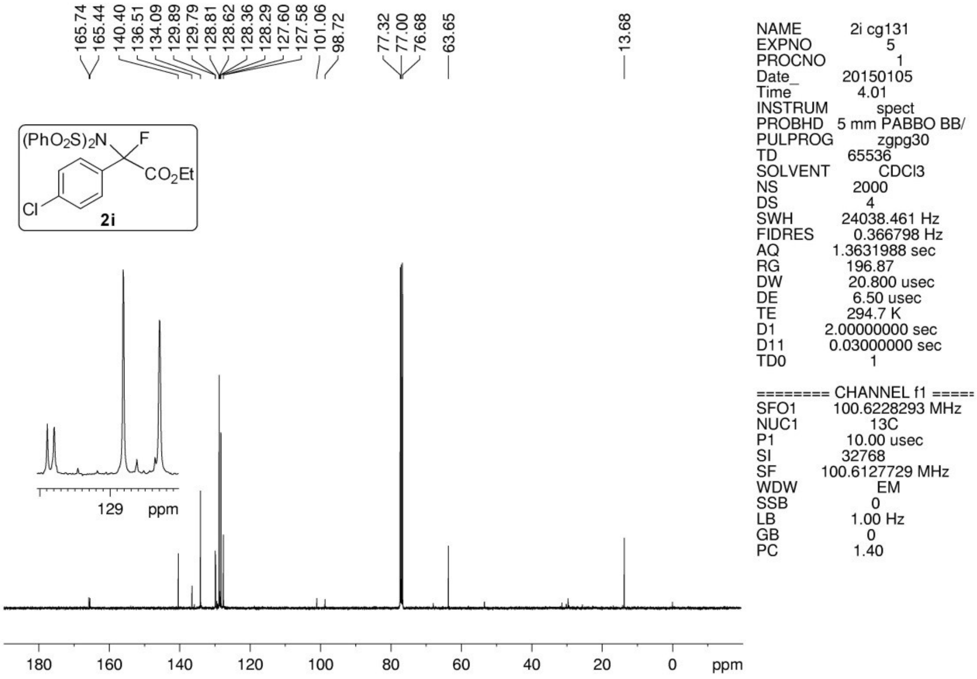

165.74 165.44 140.40 136.51 134.09 129.89 129.79 128.81 128.62 128.36 128.29 127.60 127.58 101.06 98.72 77.32 77.00 76.68 63.65 13.68
(PhO2S)2N
F
CO2Et
Cl
2i
129 ppm
180 160 140 120 100 80 60 40 20 0 ppm
NAME 2i cg131
EXPNO 5
PROCNO 1
Date_ 20150105
Time 4.01
INSTRUM spect
PROBHD 5 mm PABBO BB/
PULPROG zgpg30
TD 65536
SOLVENT CDCl3
NS 2000
DS 4
SWH 24038.461 Hz
FIDRES 0.366798 Hz
AQ 1.3631988 sec
RG 196.87
DW 20.800 usec
DE 6.50 usec
TE 294.7 K
D1 2.00000000 sec
D11 0.03000000 sec
TD0 1
======== CHANNEL f1 ========
SFO1 100.6228293 MHz
NUC1 13C
P1 10.00 usec
SI 32768
SF 100.6127729 MHz
WDW EM
SSB 0
LB 1.00 Hz
GB 0
PC 1.40

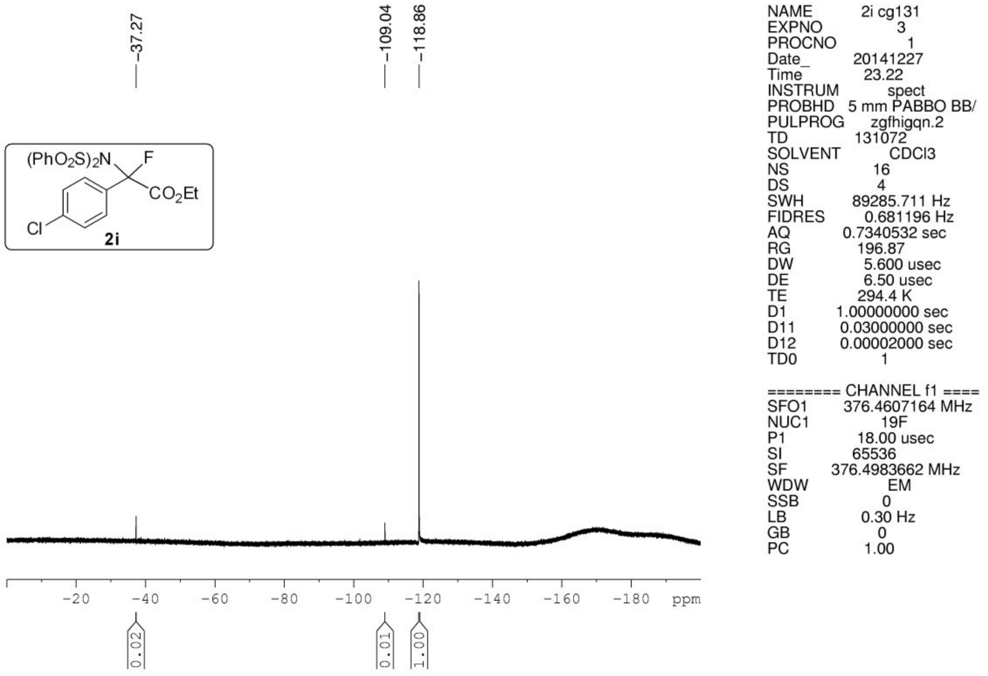
-37.27
-109.04
-118.86
(PhO2S)2N F CO2Et Cl
2i
NAME 2i cg131
EXPNO 3
PROCNO 1
Date_ 20141227
Time 23.22
INSTRUM spect
PROBHD 5 mm PABBO BB/
PULPROG zgfhigqn.2
TD 131072
SOLVENT CDCl3
NS 16
DS 4
SWH 89285.711 Hz
FIDRES 0.681196 Hz
AQ 0.7340532 sec
RG 196.87
DW 5.600 usec
DE 6.50 usec
TE 294.4 K
D1 1.00000000 sec
D11 0.03000000 sec
D12 0.00002000 sec
TD0 1
======== CHANNEL f1 ====
SFO1 376.4607164 MHz
NUC1 19F
P1 18.00 usec
SI 65536
SF 376.4983662 MHz
WDW EM
SSB 0
LB 0.30 Hz
GB 0
PC 1.00
-20 -40 -60 -80 -100 -120 -140 -160 -180 ppm
0.02 0.01 1.00

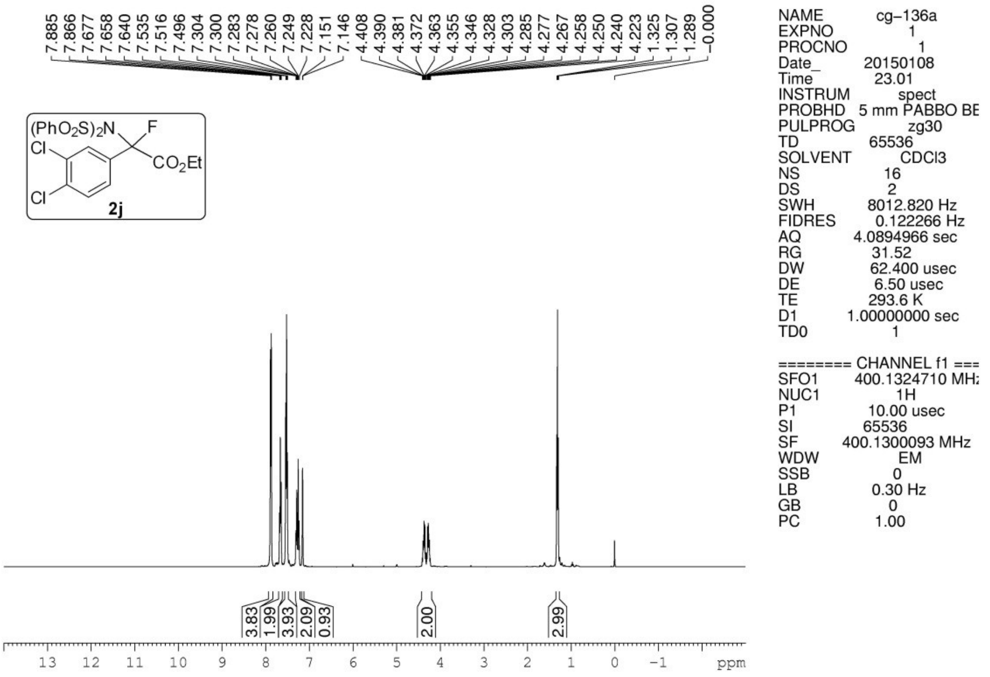
7.885 7.866 7.677 7.658 7.640 7.535 7.516 7.496 7.304 7.300 7.283 7.278 7.260 7.249 7.228 7.151 7.146 4.408 4.390 4.381 4.372 4.363 4.355 4.346 4.328 4.303 4.285 4.277 4.267 4.258 4.250 4.240 4.223 1.325 1.307 1.289 -0.000
(PhO2S)2N F Cl CO2Et Cl
2j
NAME cg-136a
EXPNO 1
PROCNO 1
Date_ 20150108
Time 23.01
INSTRUM spect
PROBHD 5 mm PABBO BE
PULPROG zg30
TD 65536
SOLVENT CDCl3
NS 16
DS 2
SWH 8012.820 Hz
FIDRES 0.122266 Hz
AQ 4.0894966 sec
RG 31.52
DW 62.400 usec
DE 6.50 usec
TE 293.6 K
D1 1.00000000 sec
TD0 1
======== CHANNEL f1 ===
SFO1 400.1324710 MH
NUC1 1H
P1 10.00 usec
SI 65536
SF 400.1300093 MHz
WDW EM
SSB 0
LB 0.30 Hz
GB 0
PC 1.00
3.83 1.99 3.93 2.09 0.93 2.00 2.99
13 12 11 10 9 8 7 6 5 4 3 2 1 0 -1 ppm

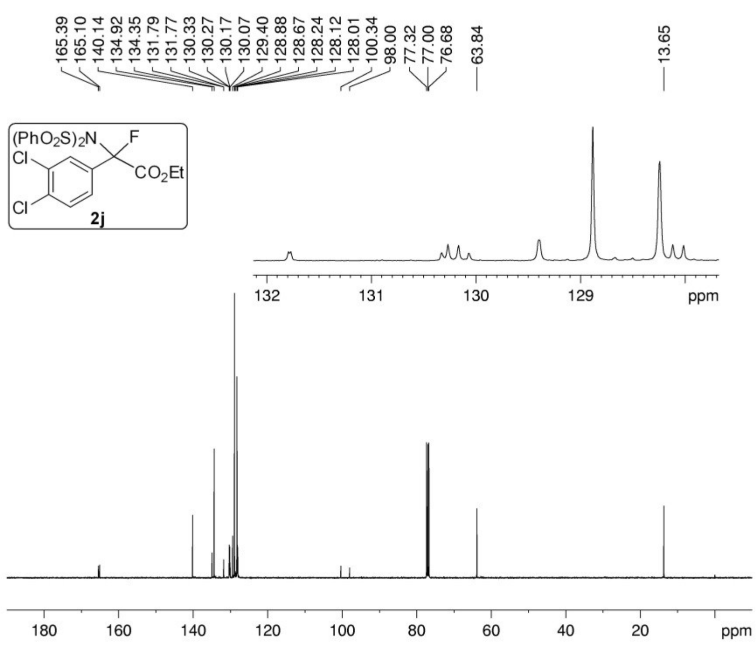

NAME 2j cg136a
EXPNO 2
PROCNO 1
Date_ 20150109
Time 0.00
INSTRUM spect
PROBHD 5 mm PABBO BB
PULPROG zgpg30
TD 65536
SOLVENT CDCl3
NS 1024
DS 4
SWH 24038.461 Hz
FIDRES 0.366798 Hz
AQ 1.3631988 sec
RG 196.87
DW 20.800 usec
DE 6.50 usec
TE 294.6 K
D1 2.00000000 sec
D11 0.03000000 sec
TD0 1

======== CHANNEL f1 ===
SFO1 100.6228293 MHz
NUC1 13C
P1 10.00 usec
SI 32768
SF 100.6127764 MHz
WDW EM
SSB 0
LB 1.00 Hz
GB 0
PC 1.40

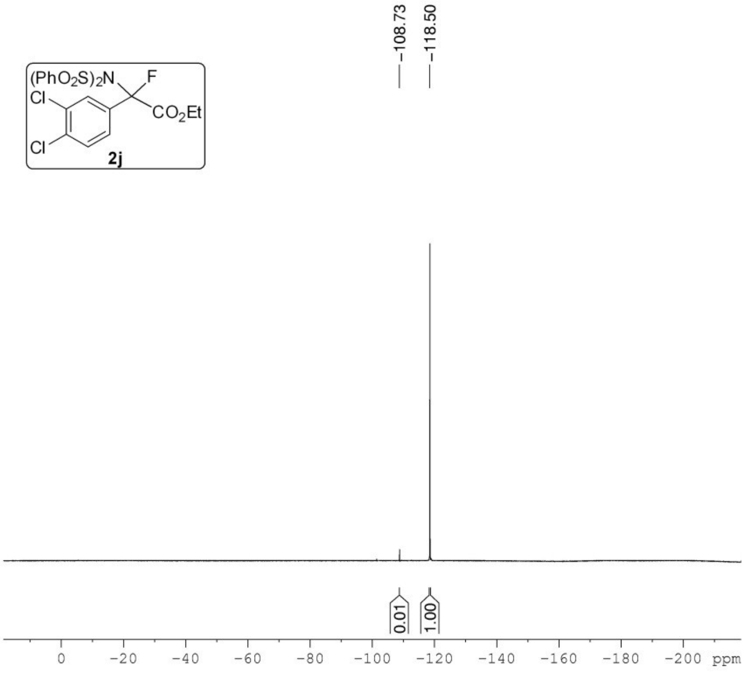

NAME 2j cg136a
EXPNO 3
PROCNO 1
Date_ 20150109
Time 0.02
INSTRUM spect
PROBHD 5 mm PABBO BB/
PULPROG zgfhigqn.2
TD 131072
SOLVENT CDCl3
NS 16
DS 4
SWH 89285.711 Hz
FIDRES 0.681196 Hz
AQ 0.7340532 sec
RG 196.87
DW 5.600 usec
DE 6.50 usec
TE 294.2 K
D1 1.00000000 sec
D11 0.03000000 sec
D12 0.00002000 sec
TD0 1

======== CHANNEL f1 =====
SFO1 376.4607164 MHz
NUC1 19F
P1 18.00 usec
SI 65536
SF 376.4983662 MHz
WDW EM
SSB 0
LB 0.30 Hz
GB 0
PC 1.00

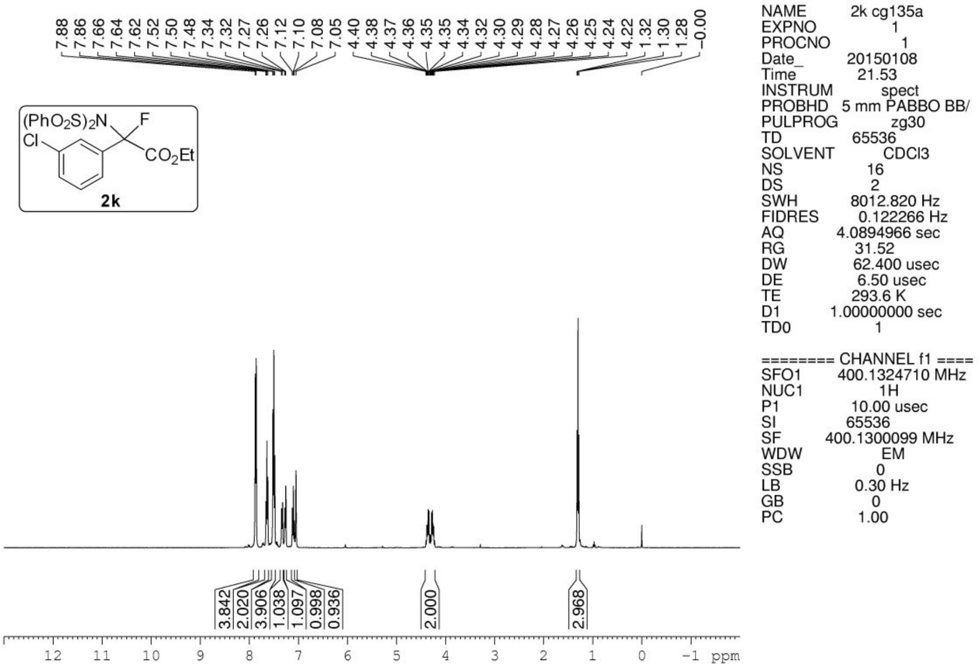
7.88 7.86 7.66 7.64 7.62 7.52 7.50 7.48 7.34 7.32 7.27 7.26 7.12 7.10 7.08 7.05 4.40 4.38 4.37 4.36 4.35 4.35 4.34 4.32 4.30 4.29 4.28 4.27 4.26 4.25 4.24 4.22 1.32 1.30 1.28 -0.00
(PhO2S)2N F
Cl
CO2Et
2k
3.842 2.020 3.906 1.038 1.097 0.998 0.936 2.000 2.968
12 11 10 9 8 7 6 5 4 3 2 1 0 -1 ppm
NAME 2k cg135a
EXPNO 1
PROCNO 1
Date_ 20150108
Time 21.53
INSTRUM spect
PROBHD 5 mm PABBO BB/
PULPROG zg30
TD 65536
SOLVENT CDCl3
NS 16
DS 2
SWH 8012.820 Hz
FIDRES 0.122266 Hz
AQ 4.0894966 sec
RG 31.52
DW 62.400 usec
DE 6.50 usec
TE 293.6 K
D1 1.00000000 sec
TD0 1
======== CHANNEL f1 ====
SFO1 400.1324710 MHz
NUC1 1H
P1 10.00 usec
SI 65536
SF 400.1300099 MHz
WDW EM
SSB 0
LB 0.30 Hz
GB 0
PC 1.00

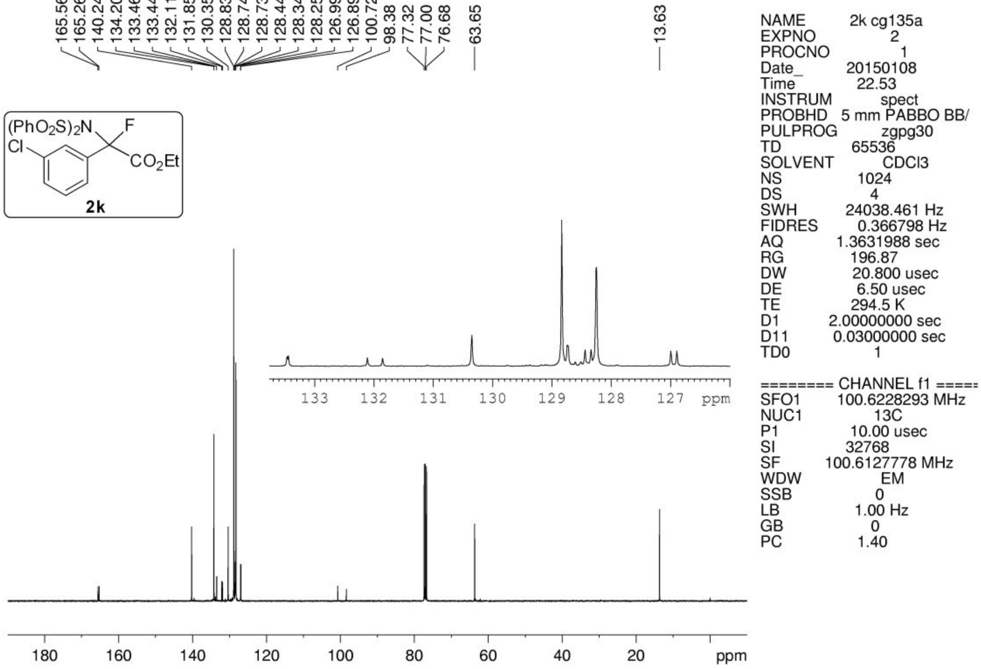
165.56 165.26 140.24 134.20 133.46 133.44 132.11 131.85 130.35 128.83 128.74 128.73 128.44 128.34 128.25 126.99 126.89 100.72 98.38 77.32 77.00 76.68 63.65 13.63
(PhO2S)2N F
Cl
CO2Et
2k
133 132 131 130 129 128 127 ppm
180 160 140 120 100 80 60 40 20 ppm
NAME 2k cg135a
EXPNO 2
PROCNO 1
Date_ 20150108
Time 22.53
INSTRUM spect
PROBHD 5 mm PABBO BB/
PULPROG zgpg30
TD 65536
SOLVENT CDCl3
NS 1024
DS 4
SWH 24038.461 Hz
FIDRES 0.366798 Hz
AQ 1.3631988 sec
RG 196.87
DW 20.800 usec
DE 6.50 usec
TE 294.5 K
D1 2.00000000 sec
D11 0.03000000 sec
TD0 1
======== CHANNEL f1 =====
SFO1 100.6228293 MHz
NUC1 13C
P1 10.00 usec
SI 32768
SF 100.6127778 MHz
WDW EM
SSB 0
LB 1.00 Hz
GB 0
PC 1.40

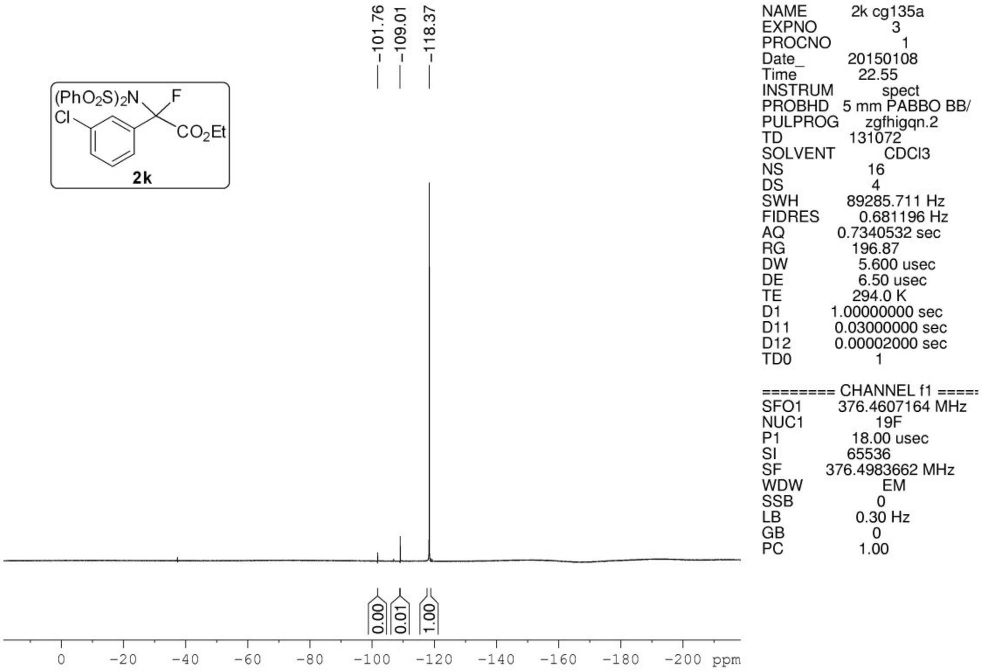
-101.76
-109.01
-118.37
(PhO2S)2N F
Cl
CO2Et
2k
0.00
0.01
1.00
0 -20 -40 -60 -80 -100 -120 -140 -160 -180 -200 ppm
NAME 2k cg135a
EXPNO 3
PROCNO 1
Date_ 20150108
Time 22.55
INSTRUM spect
PROBHD 5 mm PABBO BB/
PULPROG zgfhigqn.2
TD 131072
SOLVENT CDCl3
NS 16
DS 4
SWH 89285.711 Hz
FIDRES 0.681196 Hz
AQ 0.7340532 sec
RG 196.87
DW 5.600 usec
DE 6.50 usec
TE 294.0 K
D1 1.00000000 sec
D11 0.03000000 sec
D12 0.00002000 sec
TD0 1
======== CHANNEL f1 ====
SFO1 376.4607164 MHz
NUC1 19F
P1 18.00 usec
SI 65536
SF 376.4983662 MHz
WDW EM
SSB 0
LB 0.30 Hz
GB 0
PC 1.00

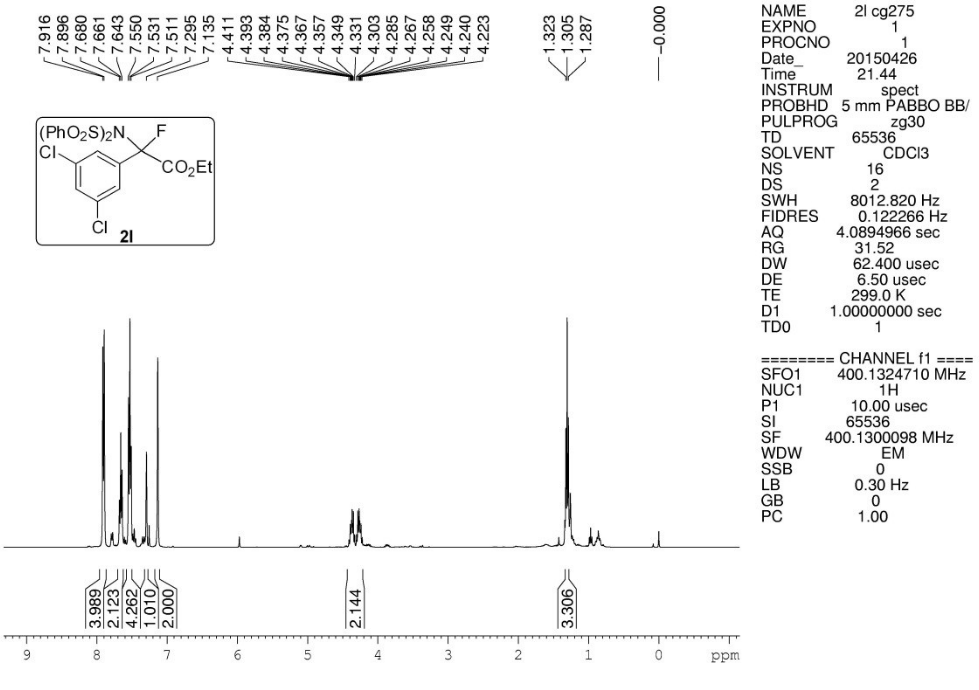
7.916
7.896
7.680
7.661
7.643
7.550
7.531
7.511
7.295
7.135
4.411
4.393
4.384
4.375
4.367
4.357
4.349
4.331
4.303
4.285
4.267
4.258
4.249
4.240
4.223
1.323
1.305
1.287
-0.000
(PhO2S)2N F
Cl
CO2Et
Cl 2l
3.989
2.123
4.262
1.010
2.000
2.144
3.306
9 8 7 6 5 4 3 2 1 0 ppm
NAME 2l cg275
EXPNO 1
PROCNO 1
Date_ 20150426
Time 21.44
INSTRUM spect
PROBHD 5 mm PABBO BB/
PULPROG zg30
TD 65536
SOLVENT CDCl3
NS 16
DS 2
SWH 8012.820 Hz
FIDRES 0.122266 Hz
AQ 4.0894966 sec
RG 31.52
DW 62.400 usec
DE 6.50 usec
TE 299.0 K
D1 1.00000000 sec
TD0 1
======== CHANNEL f1 ====
SFO1 400.1324710 MHz
NUC1 1H
P1 10.00 usec
SI 65536
SF 400.1300098 MHz
WDW EM
SSB 0
LB 0.30 Hz
GB 0
PC 1.00

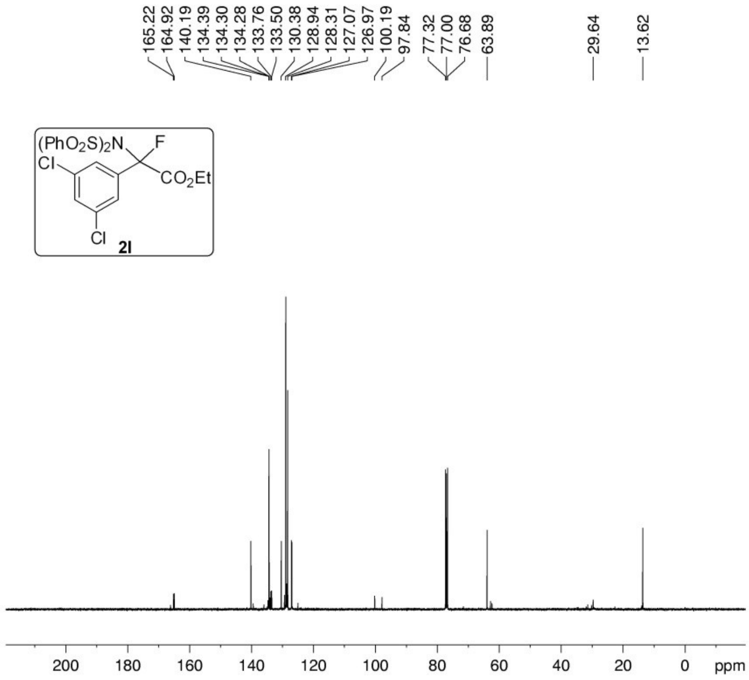

NAME 2l cg275
EXPNO 3
PROCNO 1
Date_ 20150426
Time 22.13
INSTRUM spect
PROBHD 5 mm PABBO BB/
PULPROG zgpg30
TD 65536
SOLVENT CDCl3
NS 450
DS 4
SWH 24038.461 Hz
FIDRES 0.366798 Hz
AQ 1.3631988 sec
RG 196.87
DW 20.800 usec
DE 6.50 usec
TE 299.7 K
D1 2.00000000 sec
D11 0.03000000 sec
TD0 1

======== CHANNEL f1 =====
SFO1 100.6228293 MHz
NUC1 13C
P1 10.00 usec
SI 32768
SF 100.6127750 MHz
WDW EM
SSB 0
LB 1.00 Hz
GB 0
PC 1.40

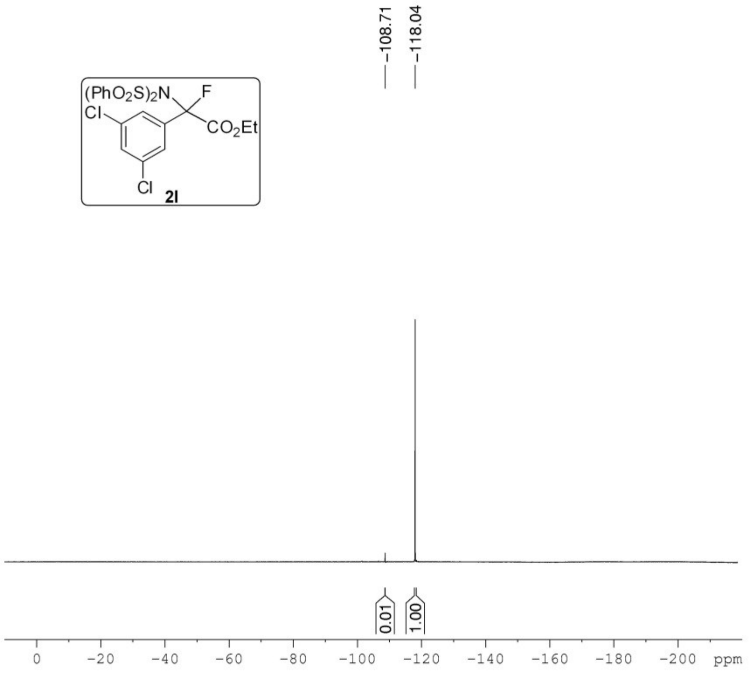

NAME 2l cg275
EXPNO 2
PROCNO 1
Date_ 20150426
Time 21.46
INSTRUM spect
PROBHD 5 mm PABBO BB/
PULPROG zgfhigqn.2
TD 131072
SOLVENT CDCl3
NS 16
DS 4
SWH 89285.711 Hz
FIDRES 0.681196 Hz
AQ 0.7340532 sec
RG 196.87
DW 5.600 usec
DE 6.50 usec
TE 299.0 K
D1 1.00000000 sec
D11 0.03000000 sec
D12 0.00002000 sec
TD0 1

======== CHANNEL f1 =====
SFO1 376.4607164 MHz
NUC1 19F
P1 18.00 usec
SI 65536
SF 376.4983662 MHz
WDW EM
SSB 0
LB 0.30 Hz
GB 0
PC 1.00

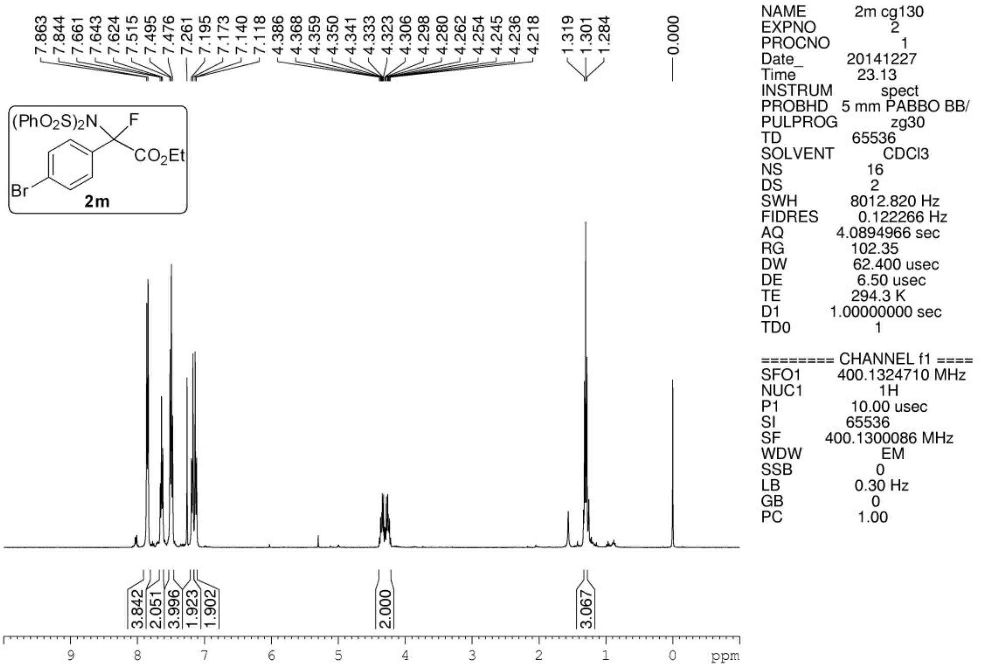
7.863 7.844 7.661 7.643 7.624 7.515 7.495 7.476 7.261 7.195 7.173 7.140 7.118 4.386 4.368 4.359 4.350 4.341 4.333 4.323 4.306 4.298 4.280 4.262 4.254 4.245 4.236 4.218 1.319 1.301 1.284 0.000
(PhO2S)2N F
CO2Et
Br
2m
3.842 2.051 3.996 1.923 1.902 2.000 3.067
9 8 7 6 5 4 3 2 1 0 ppm
NAME 2m cg130
EXPNO 2
PROCNO 1
Date_ 20141227
Time 23.13
INSTRUM spect
PROBHD 5 mm PABBO BB/
PULPROG zg30
TD 65536
SOLVENT CDCl3
NS 16
DS 2
SWH 8012.820 Hz
FIDRES 0.122266 Hz
AQ 4.0894966 sec
RG 102.35
DW 62.400 usec
DE 6.50 usec
TE 294.3 K
D1 1.00000000 sec
TD0 1
======== CHANNEL f1 ====
SFO1 400.1324710 MHz
NUC1 1H
P1 10.00 usec
SI 65536
SF 400.1300086 MHz
WDW EM
SSB 0
LB 0.30 Hz
GB 0
PC 1.00

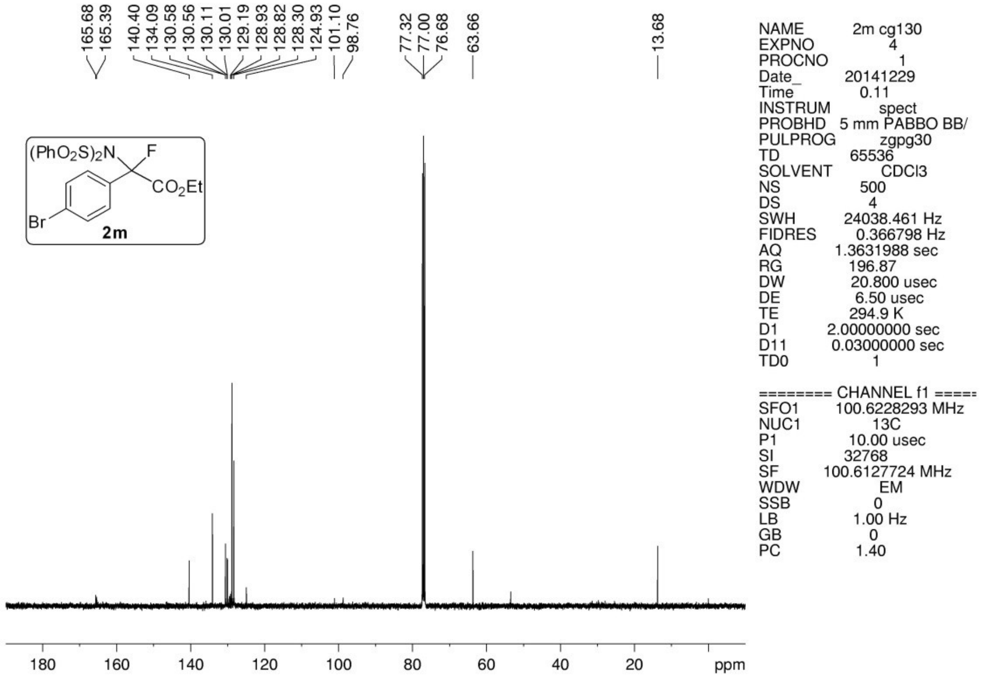
165.68 165.39 140.40 134.09 130.58 130.56 130.11 130.01 129.19 128.93 128.82 128.30 124.93 101.10 98.76 77.32 77.00 76.68 63.66 13.68
(PhO2S)2N F
CO2Et
Br
2m
180 160 140 120 100 80 60 40 20 ppm
NAME 2m cg130
EXPNO 4
PROCNO 1
Date_ 20141229
Time 0.11
INSTRUM spect
PROBHD 5 mm PABBO BB/
PULPROG zgpg30
TD 65536
SOLVENT CDCl3
NS 500
DS 4
SWH 24038.461 Hz
FIDRES 0.366798 Hz
AQ 1.3631988 sec
RG 196.87
DW 20.800 usec
DE 6.50 usec
TE 294.9 K
D1 2.00000000 sec
D11 0.03000000 sec
TD0 1
======== CHANNEL f1 =====
SFO1 100.6228293 MHz
NUC1 13C
P1 10.00 usec
SI 32768
SF 100.6127724 MHz
WDW EM
SSB 0
LB 1.00 Hz
GB 0
PC 1.40

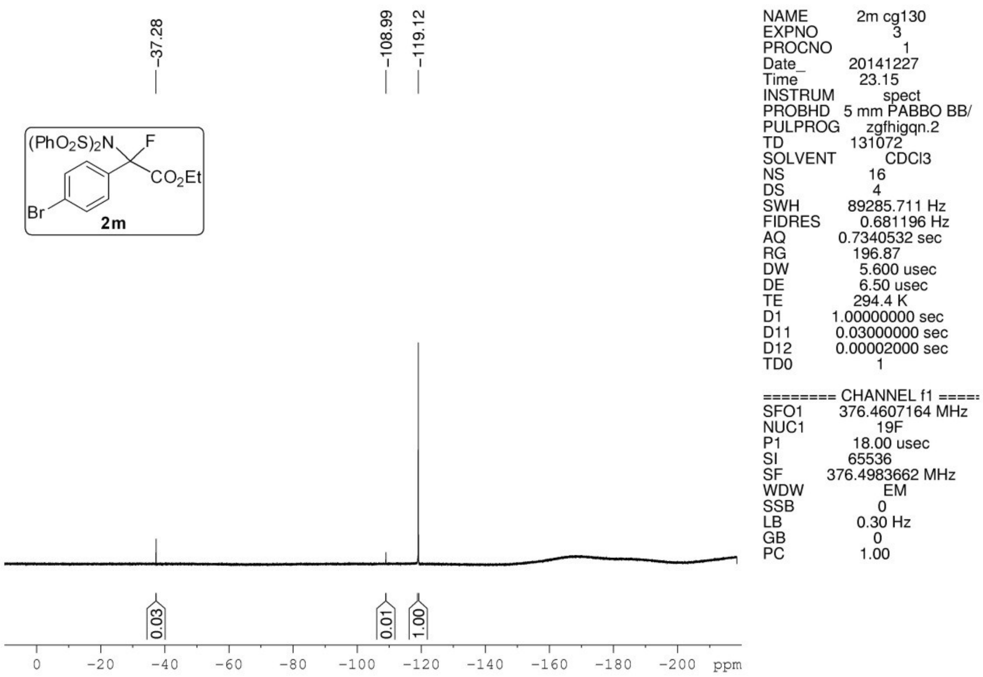

-37.28
-108.99
-119.12
(PhO2S)2N F
CO2Et
Br
2m
NAME 2m cg130
EXPNO 3
PROCNO 1
Date_ 20141227
Time 23.15
INSTRUM spect
PROBHD 5 mm PABBO BB/
PULPROG zgfhigqn.2
TD 131072
SOLVENT CDCl3
NS 16
DS 4
SWH 89285.711 Hz
FIDRES 0.681196 Hz
AQ 0.7340532 sec
RG 196.87
DW 5.600 usec
DE 6.50 usec
TE 294.4 K
D1 1.00000000 sec
D11 0.03000000 sec
D12 0.00002000 sec
TD0 1
======== CHANNEL f1 ========
SFO1 376.4607164 MHz
NUC1 19F
P1 18.00 usec
SI 65536
SF 376.4983662 MHz
WDW EM
SSB 0
LB 0.30 Hz
GB 0
PC 1.00
0.03
0.01
1.00
0 -20 -40 -60 -80 -100 -120 -140 -160 -180 -200 ppm

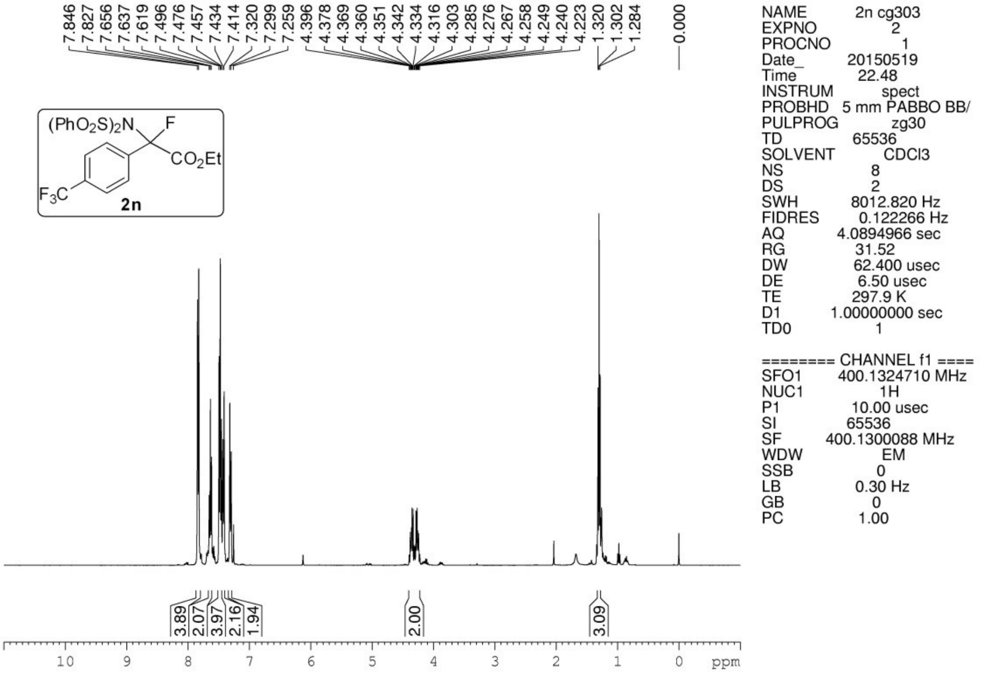

7.846 7.827 7.656 7.637 7.619 7.496 7.476 7.457 7.434 7.414 7.320 7.299 7.259 4.396 4.378 4.369 4.360 4.351 4.342 4.334 4.316 4.303 4.285 4.276 4.267 4.258 4.249 4.240 4.223 1.320 1.302 1.284 0.000
(PhO2S)2N F
CO2Et
F3C
2n
NAME 2n cg303
EXPNO 2
PROCNO 1
Date_ 20150519
Time 22.48
INSTRUM spect
PROBHD 5 mm PABBO BB/
PULPROG zg30
TD 65536
SOLVENT CDCl3
NS 8
DS 2
SWH 8012.820 Hz
FIDRES 0.122266 Hz
AQ 4.0894966 sec
RG 31.52
DW 62.400 usec
DE 6.50 usec
TE 297.9 K
D1 1.00000000 sec
TD0 1
======== CHANNEL f1 ========
SFO1 400.1324710 MHz
NUC1 1H
P1 10.00 usec
SI 65536
SF 400.1300088 MHz
WDW EM
SSB 0
LB 0.30 Hz
GB 0
PC 1.00
3.89 2.07 3.97 2.16 1.94
2.00
3.09
10 9 8 7 6 5 4 3 2 1 0 ppm

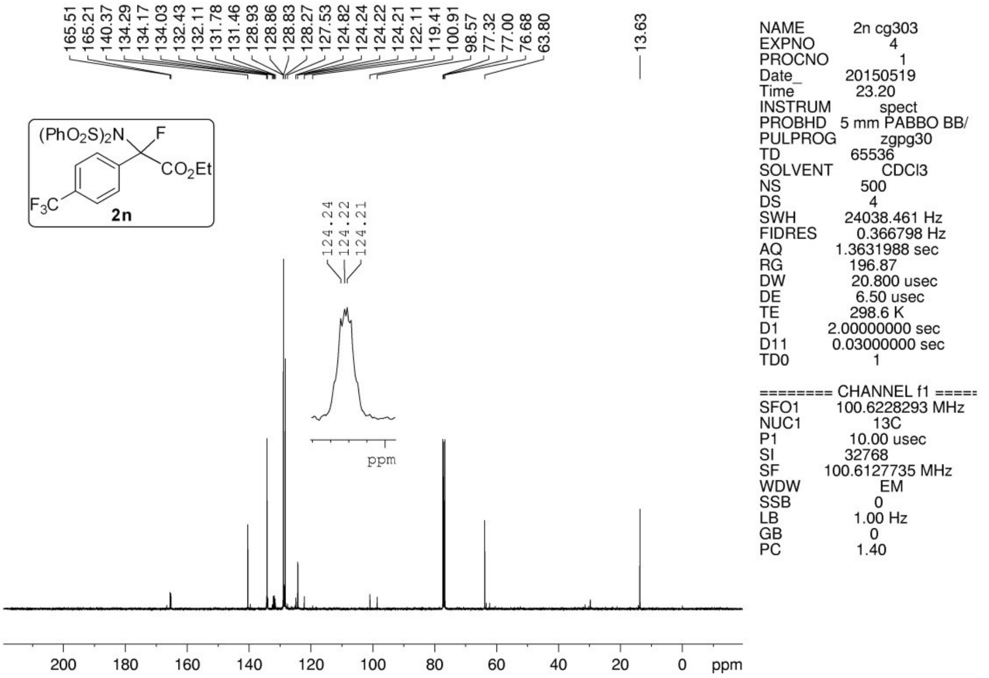
165.51
165.21
140.37
134.29
134.17
134.03
132.43
132.11
131.78
131.46
128.93
128.86
128.83
128.27
127.53
124.82
124.24
124.22
124.21
122.11
119.41
100.91
98.57
77.32
77.00
76.68
63.80
13.63
(PhO2S)2N F
CO2Et
F3C
2n
124.24
124.22
124.21
ppm
200 180 160 140 120 100 80 60 40 20 0 ppm
NAME 2n cg303
EXPNO 4
PROCNO 1
Date_ 20150519
Time 23.20
INSTRUM spect
PROBHD 5 mm PABBO BB/
PULPROG zgpg30
TD 65536
SOLVENT CDCl3
NS 500
DS 4
SWH 24038.461 Hz
FIDRES 0.366798 Hz
AQ 1.3631988 sec
RG 196.87
DW 20.800 usec
DE 6.50 usec
TE 298.6 K
D1 2.00000000 sec
D11 0.03000000 sec
TD0 1
======== CHANNEL f1 =====
SFO1 100.6228293 MHz
NUC1 13C
P1 10.00 usec
SI 32768
SF 100.6127735 MHz
WDW EM
SSB 0
LB 1.00 Hz
GB 0
PC 1.40

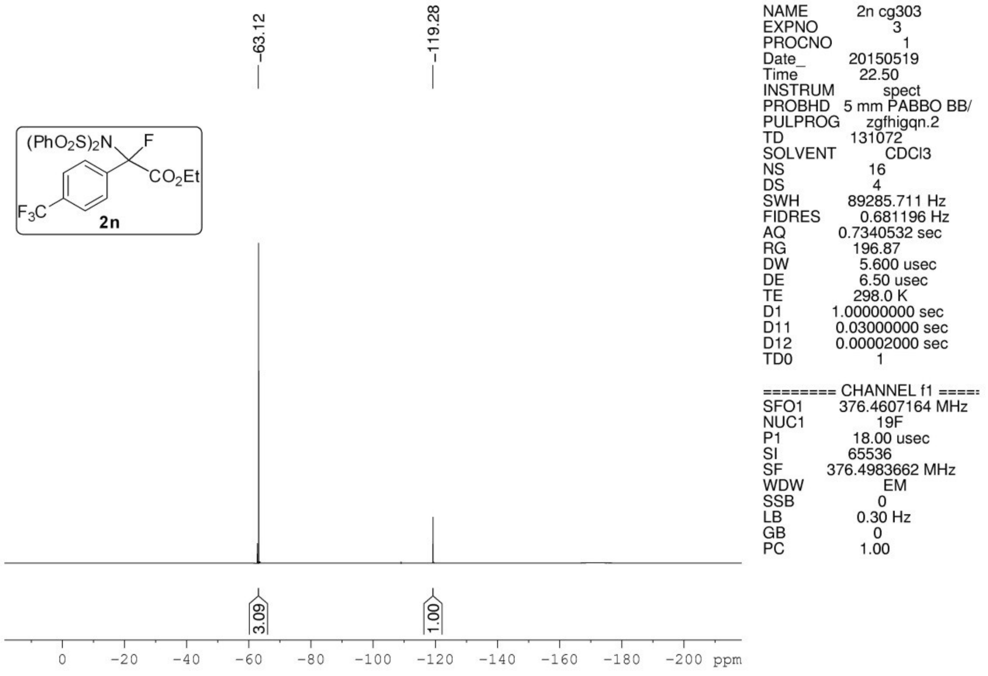
-63.12
-119.28
(PhO2S)2N F
CO2Et
F3C
2n
3.09
1.00
0 -20 -40 -60 -80 -100 -120 -140 -160 -180 -200 ppm
NAME 2n cg303
EXPNO 3
PROCNO 1
Date_ 20150519
Time 22.50
INSTRUM spect
PROBHD 5 mm PABBO BB/
PULPROG zgfhigqn.2
TD 131072
SOLVENT CDCl3
NS 16
DS 4
SWH 89285.711 Hz
FIDRES 0.681196 Hz
AQ 0.7340532 sec
RG 196.87
DW 5.600 usec
DE 6.50 usec
TE 298.0 K
D1 1.00000000 sec
D11 0.03000000 sec
D12 0.00002000 sec
TD0 1
======== CHANNEL f1 =====
SFO1 376.4607164 MHz
NUC1 19F
P1 18.00 usec
SI 65536
SF 376.4983662 MHz
WDW EM
SSB 0
LB 0.30 Hz
GB 0
PC 1.00

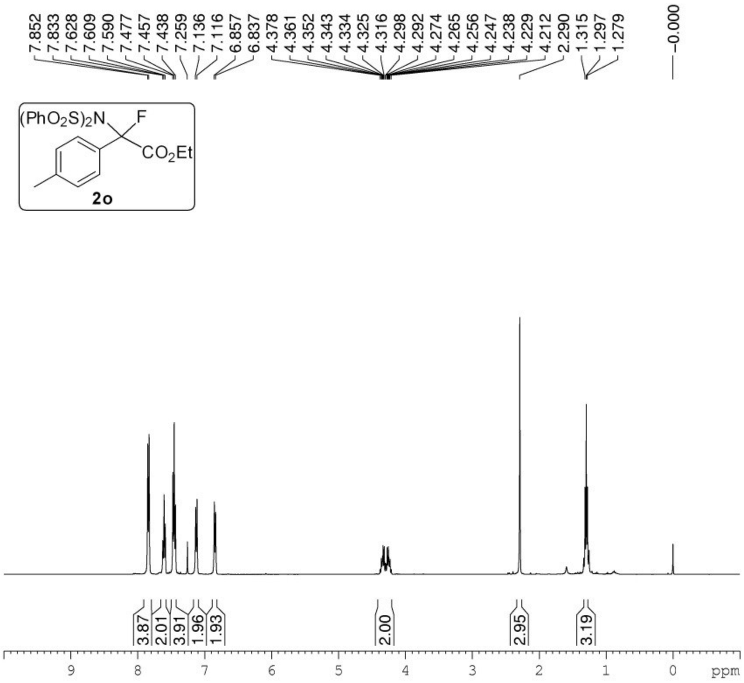

NAME 2o cg152
EXPNO 2
PROCNO 1
Date_ 20150123
Time 1.27
INSTRUM spect
PROBHD 5 mm PABBO BB/
PULPROG zg30
TD 65536
SOLVENT CDCl3
NS 16
DS 2
SWH 8012.820 Hz
FIDRES 0.122266 Hz
AQ 4.0894966 sec
RG 48.95
DW 62.400 usec
DE 6.50 usec
TE 293.5 K
D1 1.00000000 sec
TD0 1

======== CHANNEL f1 ====
SFO1 400.1324710 MHz
NUC1 1H
P1 10.00 usec
SI 65536
SF 400.1300095 MHz
WDW EM
SSB 0
LB 0.30 Hz
GB 0
PC 1.00

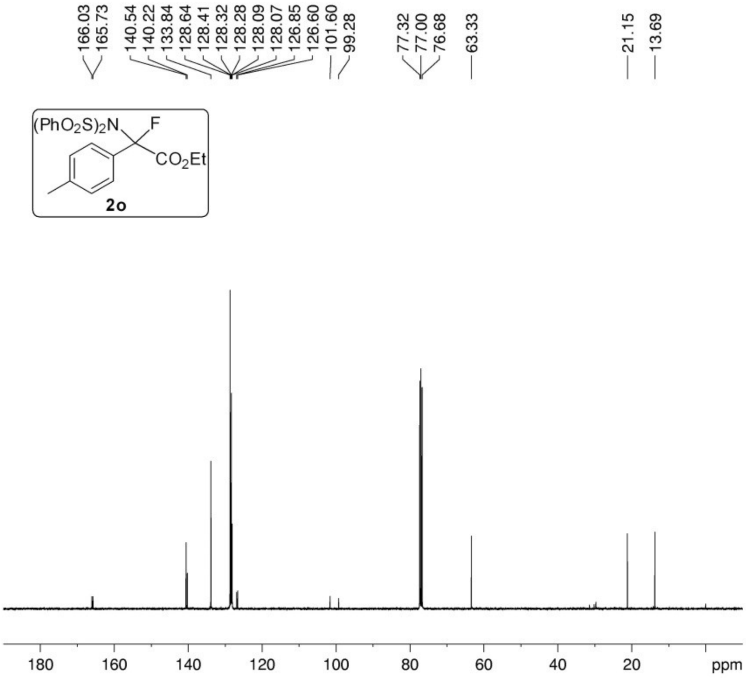

NAME 2o cg152
EXPNO 3
PROCNO 1
Date_ 20150123
Time 2.27
INSTRUM spect
PROBHD 5 mm PABBO BB/
PULPROG zgpg30
TD 65536
SOLVENT CDCl3
NS 1024
DS 4
SWH 24038.461 Hz
FIDRES 0.366798 Hz
AQ 1.3631988 sec
RG 196.87
DW 20.800 usec
DE 6.50 usec
TE 294.5 K
D1 2.00000000 sec
D11 0.03000000 sec
TD0 1

======== CHANNEL f1 =====
SFO1 100.6228293 MHz
NUC1 13C
P1 10.00 usec
SI 32768
SF 100.6127746 MHz
WDW EM
SSB 0
LB 1.00 Hz
GB 0
PC 1.40

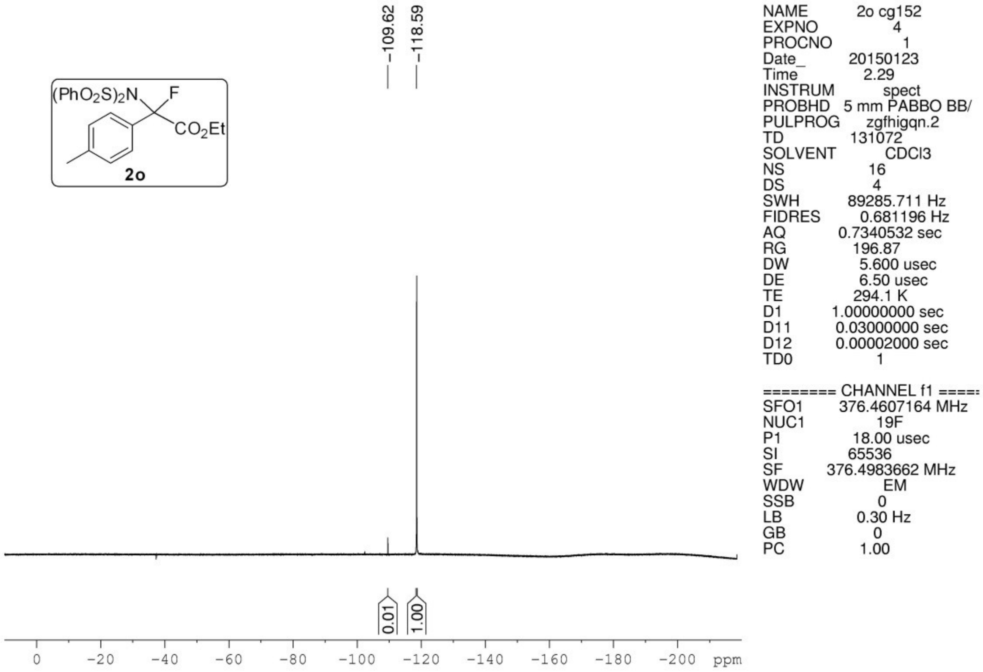
(PhO2S)2N F
CO2Et
2o
-109.62
-118.59
0.01
1.00
0 -20 -40 -60 -80 -100 -120 -140 -160 -180 -200 ppm
NAME 2o cg152
EXPNO 4
PROCNO 1
Date_ 20150123
Time 2.29
INSTRUM spect
PROBHD 5 mm PABBO BB/
PULPROG zgfhigqn.2
TD 131072
SOLVENT CDCl3
NS 16
DS 4
SWH 89285.711 Hz
FIDRES 0.681196 Hz
AQ 0.7340532 sec
RG 196.87
DW 5.600 usec
DE 6.50 usec
TE 294.1 K
D1 1.00000000 sec
D11 0.03000000 sec
D12 0.00002000 sec
TD0 1
======== CHANNEL f1 ====
SFO1 376.4607164 MHz
NUC1 19F
P1 18.00 usec
SI 65536
SF 376.4983662 MHz
WDW EM
SSB 0
LB 0.30 Hz
GB 0
PC 1.00

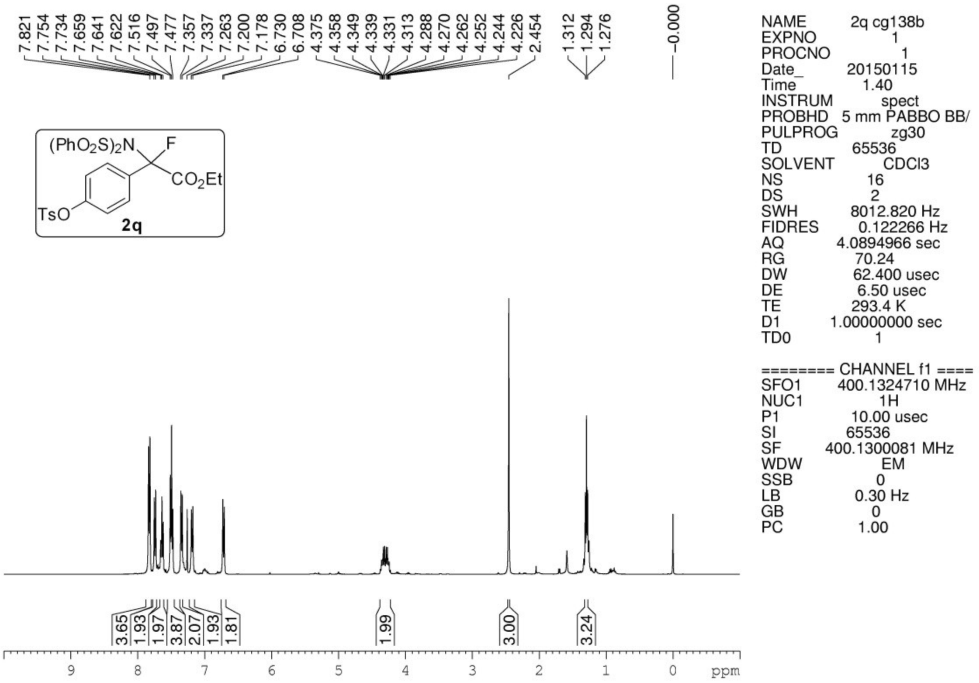
7.821 7.754 7.734 7.659 7.641 7.622 7.516 7.497 7.477 7.357 7.337 7.263 7.200 7.178 6.730 6.708 4.375 4.358 4.349 4.339 4.331 4.313 4.288 4.270 4.262 4.252 4.244 4.226 2.454 1.312 1.294 1.276 -0.000
(PhO2S)2N F
CO2Et
TsO
2q
3.65 1.93 1.97 3.87 2.07 1.93 1.81 1.99 3.00 3.24
9 8 7 6 5 4 3 2 1 0 ppm
NAME 2q cg138b
EXPNO 1
PROCNO 1
Date_ 20150115
Time 1.40
INSTRUM spect
PROBHD 5 mm PABBO BB/
PULPROG zg30
TD 65536
SOLVENT CDCl3
NS 16
DS 2
SWH 8012.820 Hz
FIDRES 0.122266 Hz
AQ 4.0894966 sec
RG 70.24
DW 62.400 usec
DE 6.50 usec
TE 293.4 K
D1 1.00000000 sec
TD0 1
======== CHANNEL f1 ====
SFO1 400.1324710 MHz
NUC1 1H
P1 10.00 usec
SI 65536
SF 400.1300081 MHz
WDW EM
SSB 0
LB 0.30 Hz
GB 0
PC 1.00

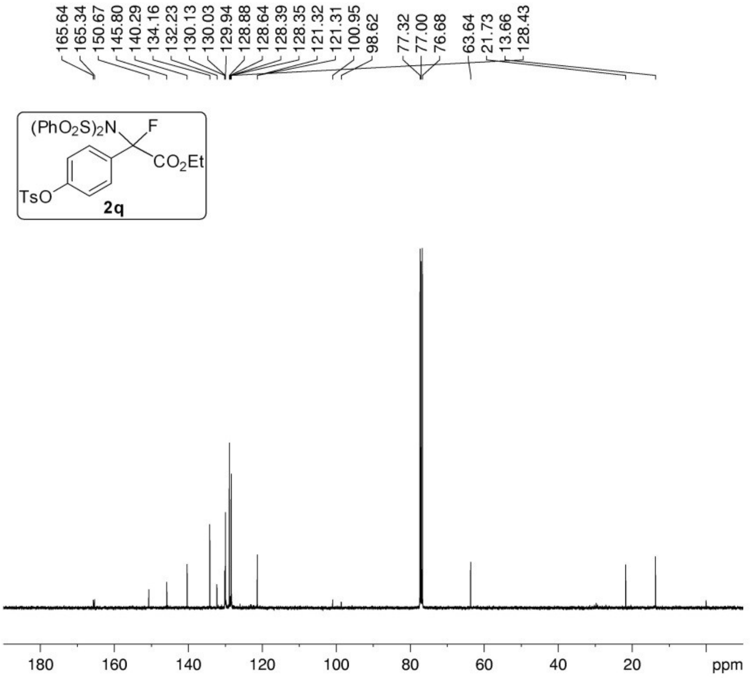

NAME 2q cg138b
EXPNO 2
PROCNO 1
Date_ 20150115
Time 2.39
INSTRUM spect
PROBHD 5 mm PABBO BB/
PULPROG zgpg30
TD 65536
SOLVENT CDCl3
NS 1024
DS 4
SWH 24038.461 Hz
FIDRES 0.366798 Hz
AQ 1.3631988 sec
RG 196.87
DW 20.800 usec
DE 6.50 usec
TE 294.3 K
D1 2.00000000 sec
D11 0.03000000 sec
TD0 1

======== CHANNEL f1 ========
SFO1 100.6228293 MHz
NUC1 13C
P1 10.00 usec
SI 32768
SF 100.6127735 MHz
WDW EM
SSB 0
LB 1.00 Hz
GB 0
PC 1.40

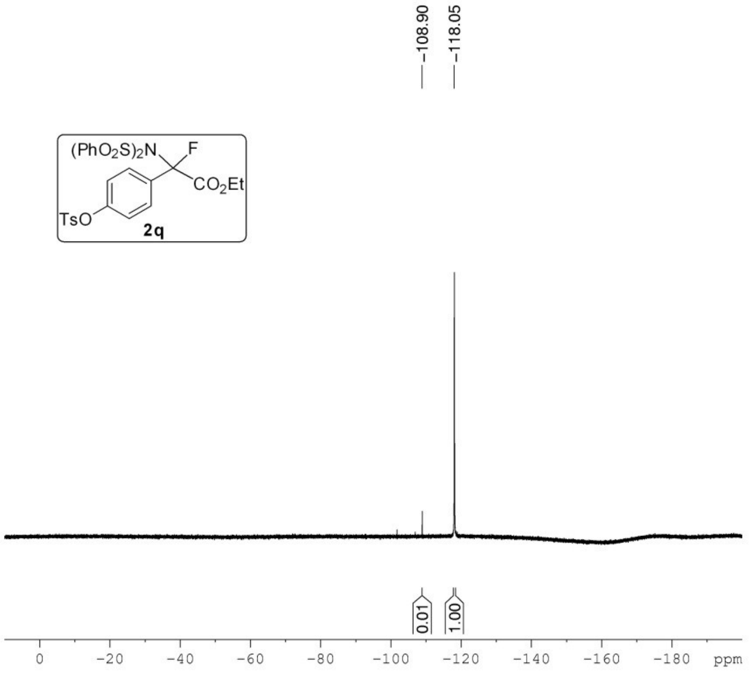

NAME 2q cg138b
EXPNO 3
PROCNO 1
Date_ 20150115
Time 2.41
INSTRUM spect
PROBHD 5 mm PABBO BB/
PULPROG zgfhigqn.2
TD 131072
SOLVENT CDCl3
NS 16
DS 4
SWH 89285.711 Hz
FIDRES 0.681196 Hz
AQ 0.7340532 sec
RG 196.87
DW 5.600 usec
DE 6.50 usec
TE 293.9 K
D1 1.00000000 sec
D11 0.03000000 sec
D12 0.00002000 sec
TD0 1

======== CHANNEL f1 ========
SFO1 376.4607164 MHz
NUC1 19F
P1 18.00 usec
SI 65536
SF 376.4983662 MHz
WDW EM
SSB 0
LB 0.30 Hz
GB 0
PC 1.00

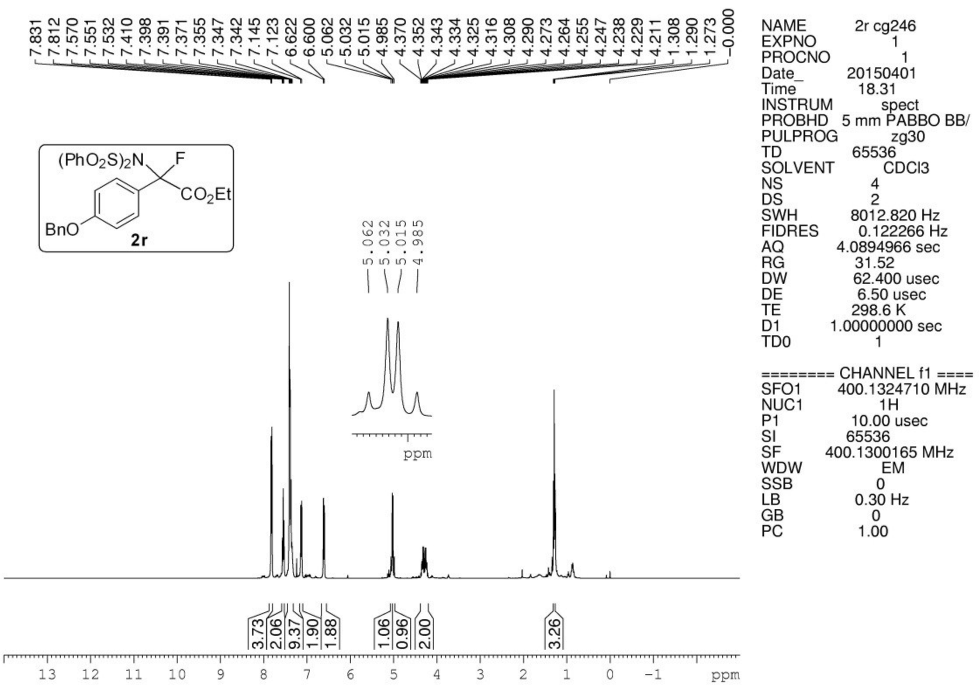

7.831
7.812
7.570
7.551
7.532
7.410
7.398
7.391
7.371
7.355
7.347
7.342
7.145
7.123
6.622
6.600
5.062
5.032
5.015
4.985
4.370
4.352
4.343
4.334
4.325
4.316
4.308
4.290
4.273
4.264
4.255
4.247
4.238
4.229
4.211
1.308
1.290
1.273
-0.000
(PhO2S)2N
F
CO2Et
BnO
2r
5.062
5.032
5.015
4.985
ppm
3.73
2.06
9.37
1.90
1.88
1.06
0.96
2.00
3.26
13 12 11 10 9 8 7 6 5 4 3 2 1 0 -1 ppm
NAME 2r cg246
EXPNO 1
PROCNO 1
Date_ 20150401
Time 18.31
INSTRUM spect
PROBHD 5 mm PABBO BB/
PULPROG zg30
TD 65536
SOLVENT CDCl3
NS 4
DS 2
SWH 8012.820 Hz
FIDRES 0.122266 Hz
AQ 4.0894966 sec
RG 31.52
DW 62.400 usec
DE 6.50 usec
TE 298.6 K
D1 1.00000000 sec
TD0 1
======== CHANNEL f1 ====
SFO1 400.1324710 MHz
NUC1 1H
P1 10.00 usec
SI 65536
SF 400.1300165 MHz
WDW EM
SSB 0
LB 0.30 Hz
GB 0
PC 1.00

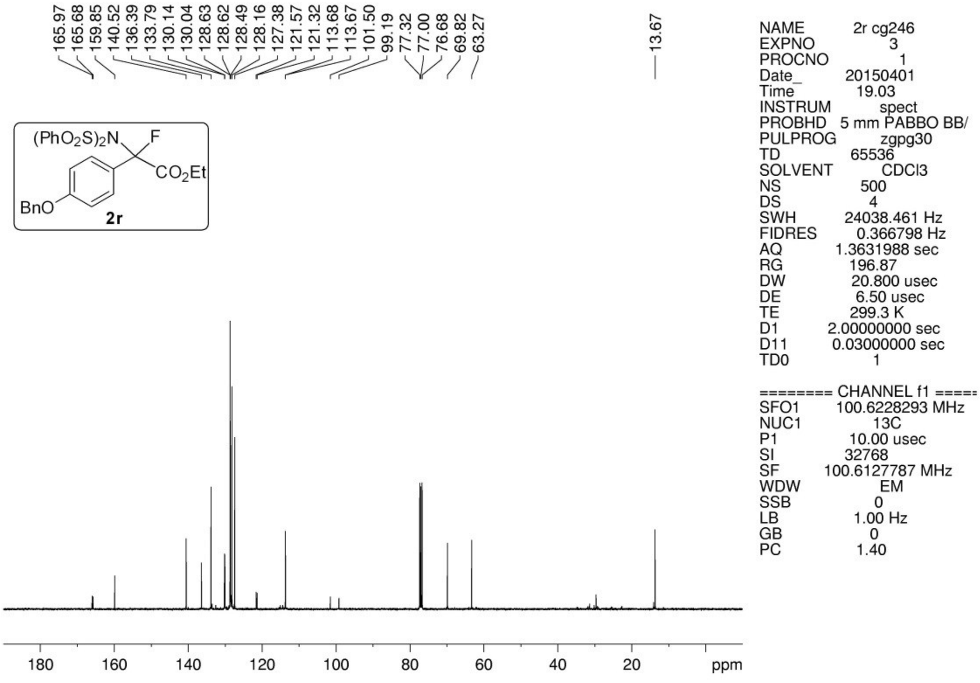

165.97
165.68
159.85
140.52
136.39
133.79
130.14
130.04
128.63
128.62
128.49
128.16
127.38
121.57
121.32
113.68
113.67
101.50
99.19
77.32
77.00
76.68
69.82
63.27
13.67
(PhO2S)2N
F
CO2Et
BnO
2r
180 160 140 120 100 80 60 40 20 ppm
NAME 2r cg246
EXPNO 3
PROCNO 1
Date_ 20150401
Time 19.03
INSTRUM spect
PROBHD 5 mm PABBO BB/
PULPROG zgpg30
TD 65536
SOLVENT CDCl3
NS 500
DS 4
SWH 24038.461 Hz
FIDRES 0.366798 Hz
AQ 1.3631988 sec
RG 196.87
DW 20.800 usec
DE 6.50 usec
TE 299.3 K
D1 2.00000000 sec
D11 0.03000000 sec
TD0 1
======== CHANNEL f1 =====
SFO1 100.6228293 MHz
NUC1 13C
P1 10.00 usec
SI 32768
SF 100.6127787 MHz
WDW EM
SSB 0
LB 1.00 Hz
GB 0
PC 1.40

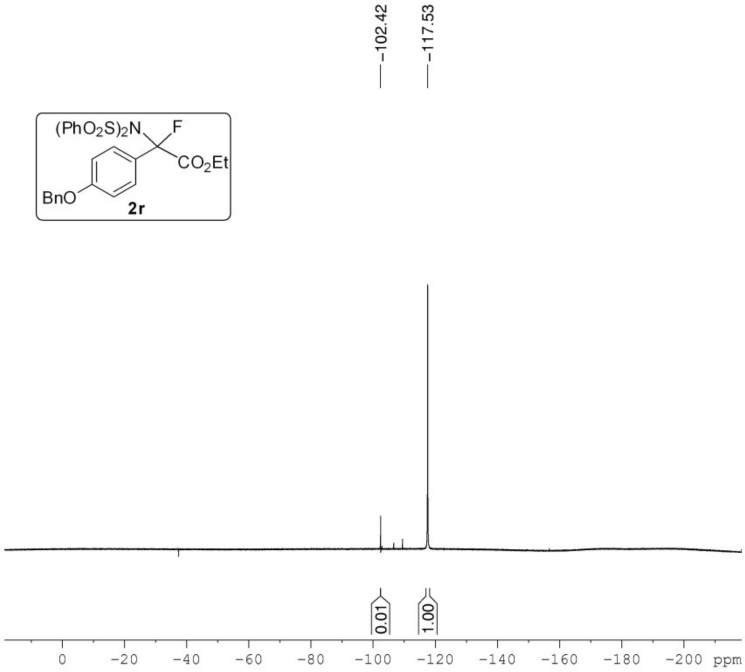

NAME 2r cg246
EXPNO 2
PROCNO 1
Date_ 20150401
Time 18.33
INSTRUM spect
PROBHD 5 mm PABBO BB/
PULPROG zgfhigqn.2
TD 131072
SOLVENT CDCl3
NS 16
DS 4
SWH 89285.711 Hz
FIDRES 0.681196 Hz
AQ 0.7340532 sec
RG 196.87
DW 5.600 usec
DE 6.50 usec
TE 298.6 K
D1 1.00000000 sec
D11 0.03000000 sec
D12 0.00002000 sec
TD0 1

======== CHANNEL f1 ========
SFO1 376.4607164 MHz
NUC1 19F
P1 18.00 usec
SI 65536
SF 376.4983662 MHz
WDW EM
SSB 0
LB 0.30 Hz
GB 0
PC 1.00

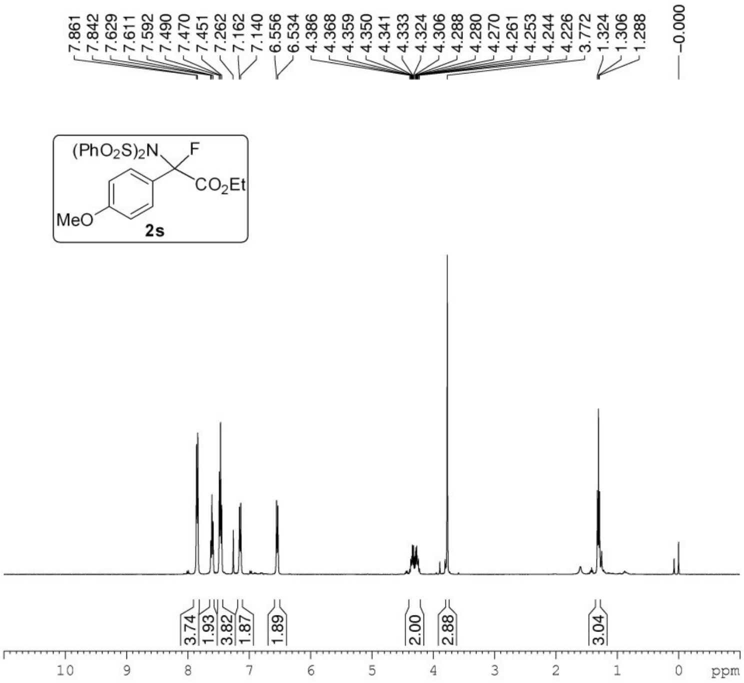

NAME 2s cg203
EXPNO 1
PROCNO 1
Date_ 20150211
Time 22.22
INSTRUM spect
PROBHD 5 mm PABBO BB/
PULPROG zg30
TD 65536
SOLVENT CDCl3
NS 16
DS 2
SWH 8012.820 Hz
FIDRES 0.122266 Hz
AQ 4.0894966 sec
RG 62.19
DW 62.400 usec
DE 6.50 usec
TE 292.3 K
D1 1.00000000 sec
TD0 1

======== CHANNEL f1 ========
SFO1 400.1324710 MHz
NUC1 1H
P1 10.00 usec
SI 65536
SF 400.1300079 MHz
WDW EM
SSB 0
LB 0.30 Hz
GB 0
PC 1.00

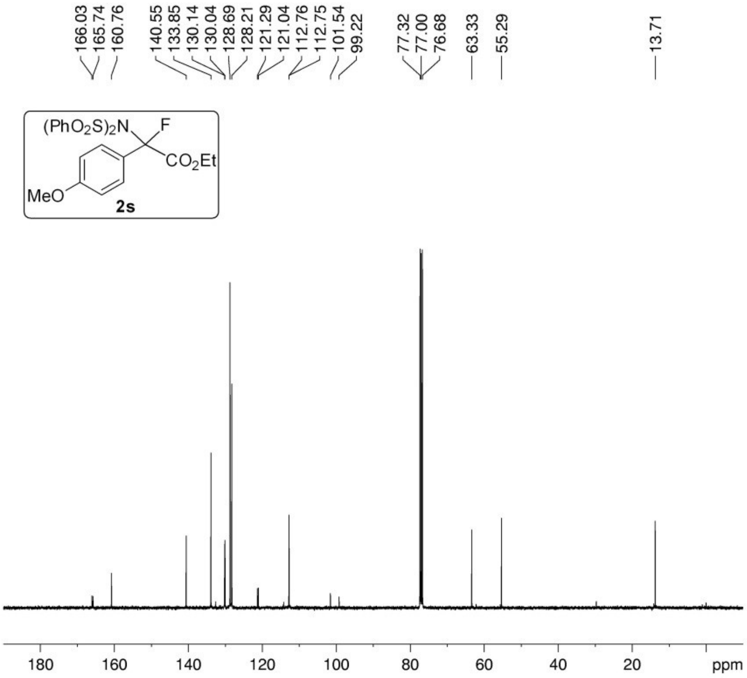

NAME 2s cg203
EXPNO 3
PROCNO 1
Date_ 20150211
Time 23.16
INSTRUM spect
PROBHD 5 mm PABBO BB/
PULPROG zgpg30
TD 65536
SOLVENT CDCl3
NS 1024
DS 4
SWH 24038.461 Hz
FIDRES 0.366798 Hz
AQ 1.3631988 sec
RG 196.87
DW 20.800 usec
DE 6.50 usec
TE 293.3 K
D1 2.00000000 sec
D11 0.03000000 sec
TD0 1

======== CHANNEL f1 =====
SFO1 100.6228293 MHz
NUC1 13C
P1 10.00 usec
SI 32768
SF 100.6127743 MHz
WDW EM
SSB 0
LB 1.00 Hz
GB 0
PC 1.40

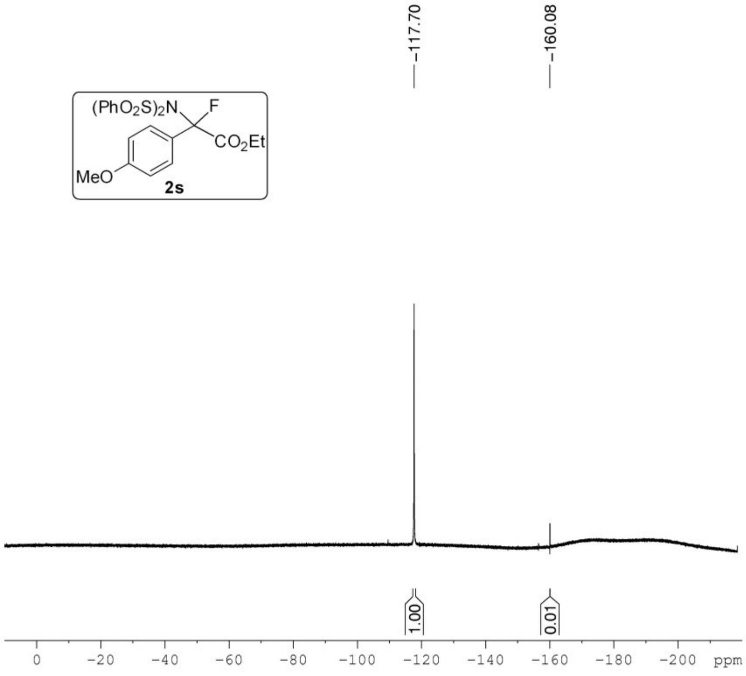

NAME 2s cg203
EXPNO 2
PROCNO 1
Date_ 20150211
Time 23.24
INSTRUM spect
PROBHD 5 mm PABBO BB/
PULPROG zgfhigqn.2
TD 131072
SOLVENT CDCl3
NS 16
DS 4
SWH 89285.711 Hz
FIDRES 0.681196 Hz
AQ 0.7340532 sec
RG 196.87
DW 5.600 usec
DE 6.50 usec
TE 292.8 K
D1 1.00000000 sec
D11 0.03000000 sec
D12 0.00002000 sec
TD0 1

======== CHANNEL f1 =====
SFO1 376.4607164 MHz
NUC1 19F
P1 18.00 usec
SI 65536
SF 376.4983662 MHz
WDW EM
SSB 0
LB 0.30 Hz
GB 0
PC 1.00

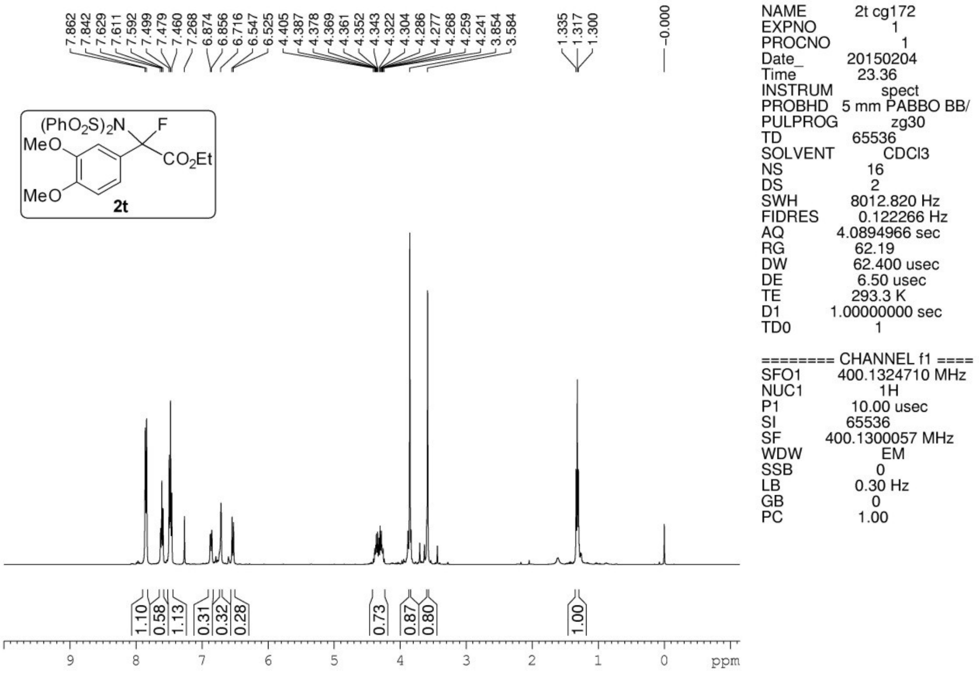
7.862 7.842 7.629 7.611 7.592 7.499 7.479 7.460 7.268 6.874 6.856 6.716 6.547 6.525 4.405 4.387 4.378 4.369 4.361 4.352 4.343 4.322 4.304 4.286 4.277 4.268 4.259 4.241 3.854 3.584
1.335 1.317 1.300
-0.000
(PhO2S)2N F
MeO
CO2Et
MeO
2t
1.10 0.58 1.13 0.31 0.32 0.28 0.73 0.87 0.80 1.00
9 8 7 6 5 4 3 2 1 0 ppm
NAME 2t cg172
EXPNO 1
PROCNO 1
Date_ 20150204
Time 23.36
INSTRUM spect
PROBHD 5 mm PABBO BB/
PULPROG zg30
TD 65536
SOLVENT CDCl3
NS 16
DS 2
SWH 8012.820 Hz
FIDRES 0.122266 Hz
AQ 4.0894966 sec
RG 62.19
DW 62.400 usec
DE 6.50 usec
TE 293.3 K
D1 1.00000000 sec
TD0 1
======== CHANNEL f1 ====
SFO1 400.1324710 MHz
NUC1 1H
P1 10.00 usec
SI 65536
SF 400.1300057 MHz
WDW EM
SSB 0
LB 0.30 Hz
GB 0
PC 1.00

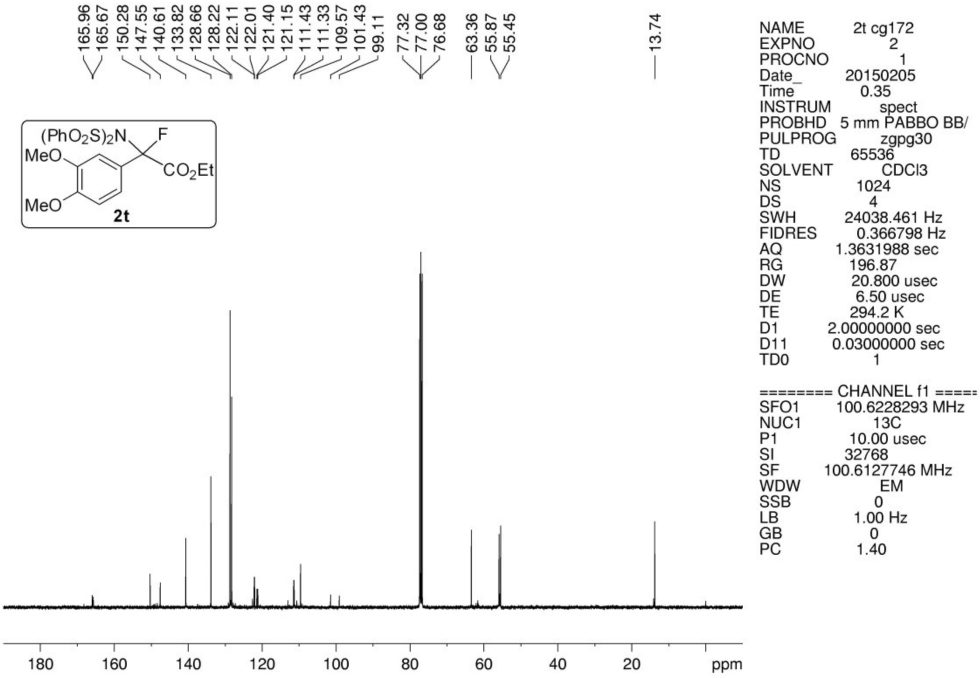
165.96 165.67 150.28 147.55 140.61 133.82 128.66 128.22 122.11 122.01 121.40 121.15 111.43 111.33 109.57 101.43 99.11 77.32 77.00 76.68 63.36 55.87 55.45
13.74
(PhO2S)2N F
MeO
CO2Et
MeO
2t
180 160 140 120 100 80 60 40 20 ppm
NAME 2t cg172
EXPNO 2
PROCNO 1
Date_ 20150205
Time 0.35
INSTRUM spect
PROBHD 5 mm PABBO BB/
PULPROG zgpg30
TD 65536
SOLVENT CDCl3
NS 1024
DS 4
SWH 24038.461 Hz
FIDRES 0.366798 Hz
AQ 1.3631988 sec
RG 196.87
DW 20.800 usec
DE 6.50 usec
TE 294.2 K
D1 2.00000000 sec
D11 0.03000000 sec
TD0 1
======== CHANNEL f1 =====
SFO1 100.6228293 MHz
NUC1 13C
P1 10.00 usec
SI 32768
SF 100.6127746 MHz
WDW EM
SSB 0
LB 1.00 Hz
GB 0
PC 1.40

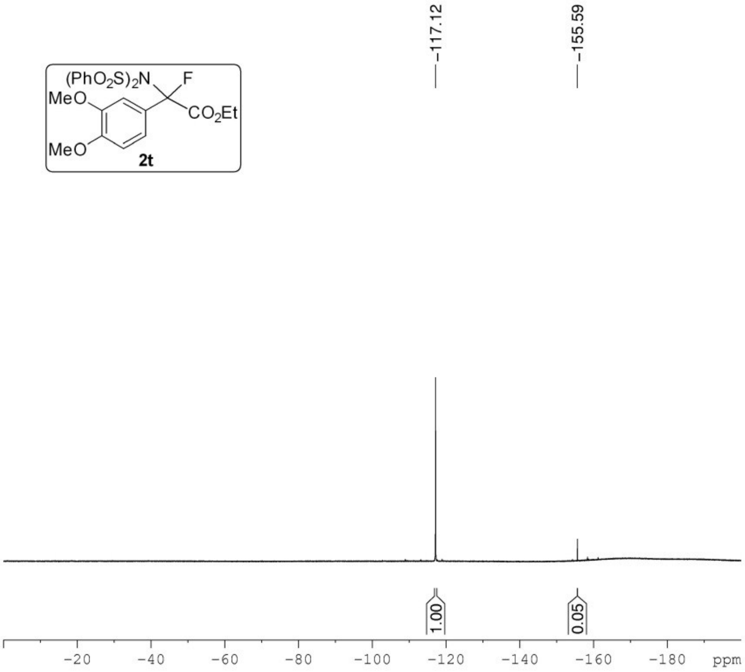

NAME 2t cg172
EXPNO 3
PROCNO 1
Date_ 20150205
Time 0.37
INSTRUM spect
PROBHD 5 mm PABBO BB/
PULPROG zgfhigqn.2
TD 131072
SOLVENT CDCl3
NS 16
DS 4
SWH 89285.711 Hz
FIDRES 0.681196 Hz
AQ 0.7340532 sec
RG 196.87
DW 5.600 usec
DE 6.50 usec
TE 293.7 K
D1 1.00000000 sec
D11 0.03000000 sec
D12 0.00002000 sec
TD0 1

======== CHANNEL f1 ====
SFO1 376.4607164 MHz
NUC1 19F
P1 18.00 usec
SI 65536
SF 376.4983662 MHz
WDW EM
SSB 0
LB 0.30 Hz
GB 0
PC 1.00

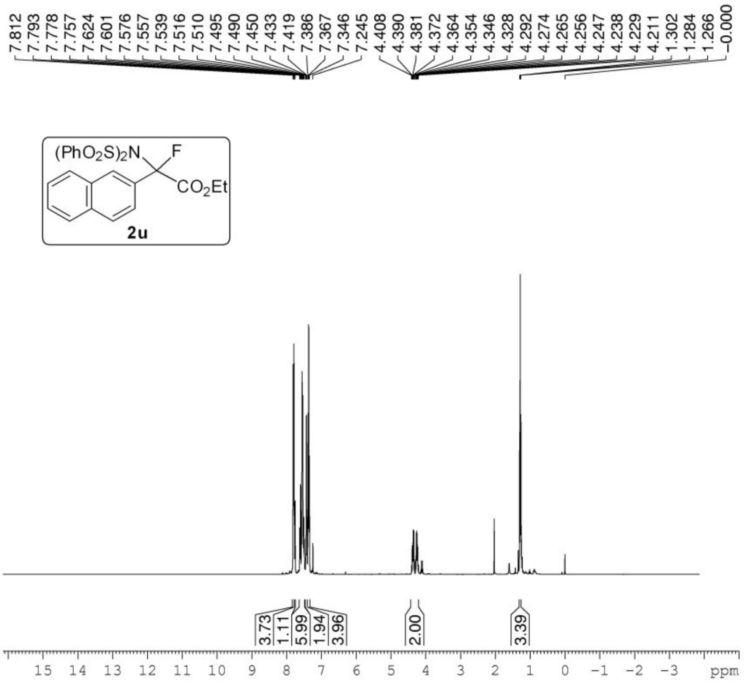

NAME 2u cg272
EXPNO 1
PROCNO 1
Date_ 20150422
Time 21.57
INSTRUM spect
PROBHD 5 mm PABBO BB/
PULPROG zg30
TD 65536
SOLVENT CDCl3
NS 4
DS 2
SWH 8012.820 Hz
FIDRES 0.122266 Hz
AQ 4.0894966 sec
RG 31.52
DW 62.400 usec
DE 6.50 usec
TE 298.5 K
D1 1.00000000 sec
TD0 1

======== CHANNEL f1 ====
SFO1 400.1324710 MHz
NUC1 1H
P1 10.00 usec
SI 65536
SF 400.1300143 MHz
WDW EM
SSB 0
LB 0.30 Hz
GB 0
PC 1.00

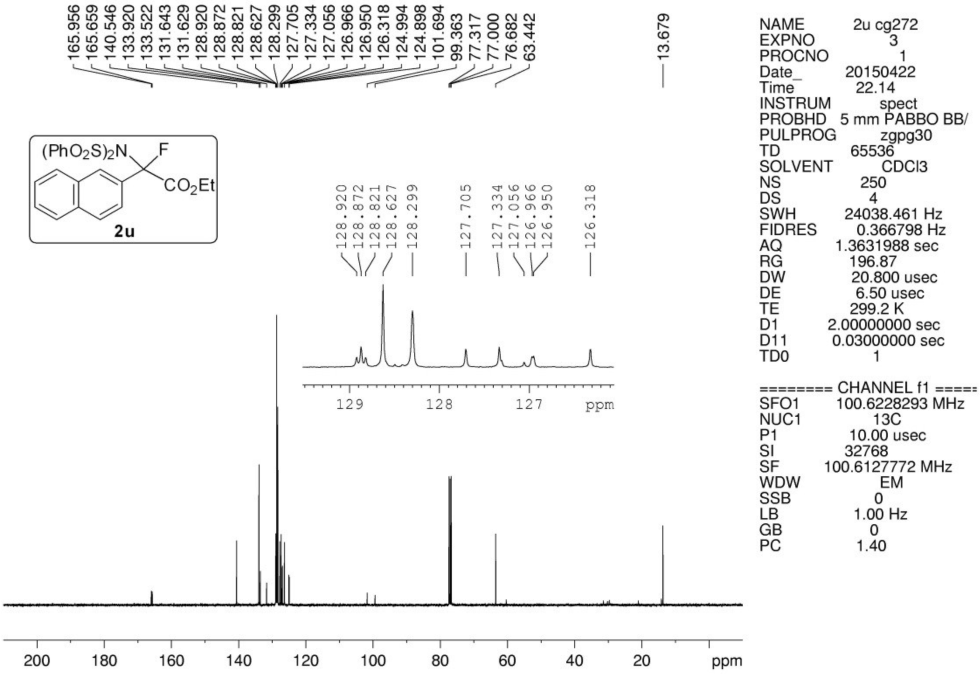

165.956
165.659
140.546
133.920
133.522
131.643
131.629
128.920
128.872
128.821
128.627
128.299
127.705
127.334
127.056
126.966
126.950
126.318
124.994
124.898
101.694
99.363
77.317
77.000
76.682
63.442
13.679
$(PhO_2S)_2N$
F
CO_2Et
2u
129
128
127
ppm
200
180
160
140
120
100
80
60
40
20
ppm
NAME 2u cg272
EXPNO 3
PROCNO 1
Date_ 20150422
Time 22.14
INSTRUM spect
PROBHD 5 mm PABBO BB/
PULPROG zgpg30
TD 65536
SOLVENT CDCl3
NS 250
DS 4
SWH 24038.461 Hz
FIDRES 0.366798 Hz
AQ 1.3631988 sec
RG 196.87
DW 20.800 usec
DE 6.50 usec
TE 299.2 K
D1 2.00000000 sec
D11 0.03000000 sec
TD0 1
======== CHANNEL f1 =====
SFO1 100.6228293 MHz
NUC1 13C
P1 10.00 usec
SI 32768
SF 100.6127772 MHz
WDW EM
SSB 0
LB 1.00 Hz
GB 0
PC 1.40

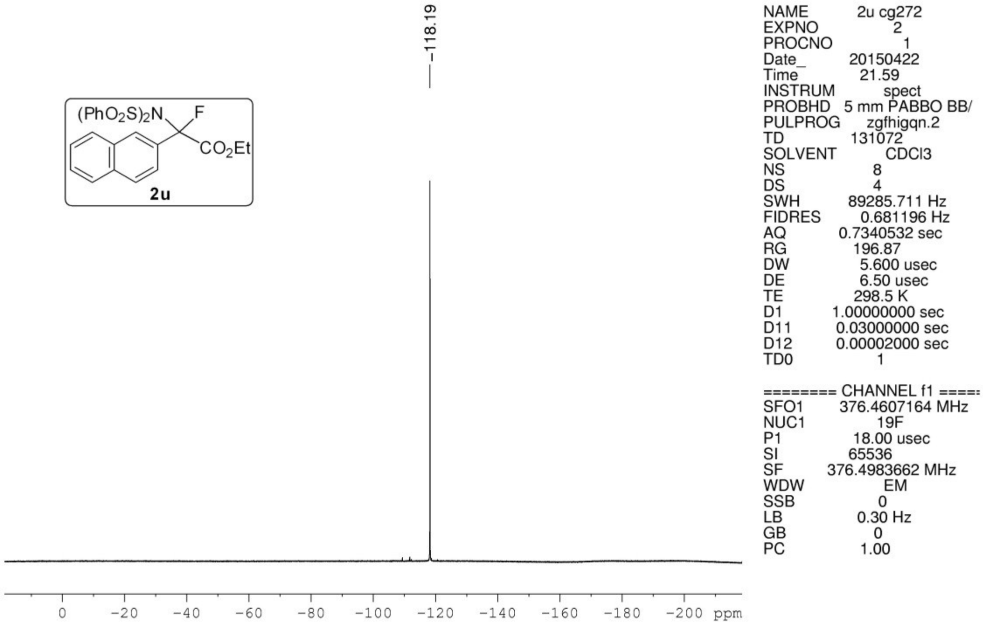

-118.19
$(PhO_2S)_2N$
F
CO_2Et
2u
0
-20
-40
-60
-80
-100
-120
-140
-160
-180
-200 ppm
NAME 2u cg272
EXPNO 2
PROCNO 1
Date_ 20150422
Time 21.59
INSTRUM spect
PROBHD 5 mm PABBO BB/
PULPROG zgfhigqn.2
TD 131072
SOLVENT CDCl3
NS 8
DS 4
SWH 89285.711 Hz
FIDRES 0.681196 Hz
AQ 0.7340532 sec
RG 196.87
DW 5.600 usec
DE 6.50 usec
TE 298.5 K
D1 1.00000000 sec
D11 0.03000000 sec
D12 0.00002000 sec
TD0 1
======== CHANNEL f1 =====
SFO1 376.4607164 MHz
NUC1 19F
P1 18.00 usec
SI 65536
SF 376.4983662 MHz
WDW EM
SSB 0
LB 0.30 Hz
GB 0
PC 1.00

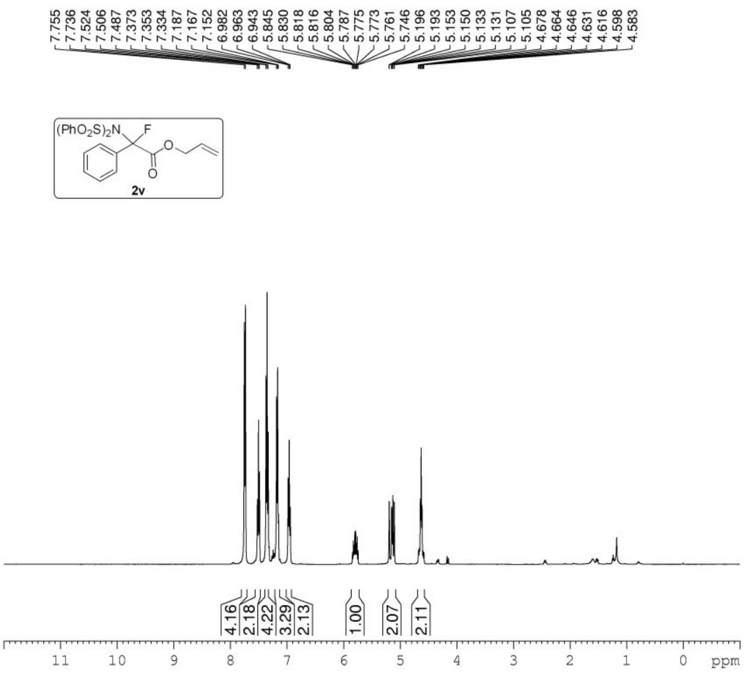

NAME 2v cg128
EXPNO 1
PROCNO 1
Date_ 20150618
Time 0.04
INSTRUM spect
PROBHD 5 mm PABBO BB/
PULPROG zg30
TD 65536
SOLVENT CDCl3
NS 16
DS 2
SWH 8012.820 Hz
FIDRES 0.122266 Hz
AQ 4.0894966 sec
RG 31.52
DW 62.400 usec
DE 6.50 usec
TE 297.9 K
D1 1.00000000 sec
TD0 1

======== CHANNEL f1 ====
SFO1 400.1324710 MHz
NUC1 1H
P1 10.00 usec
SI 65536
SF 400.1300473 MHz
WDW EM
SSB 0
LB 0.30 Hz
GB 0
PC 1.00

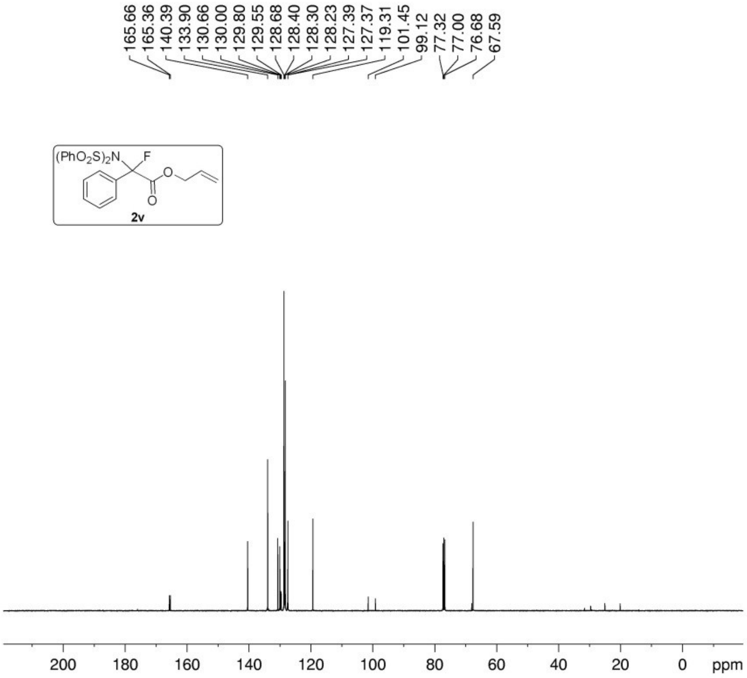

NAME 2v cg128
EXPNO 3
PROCNO 1
Date_ 20150618
Time 1.04
INSTRUM spect
PROBHD 5 mm PABBO BB/
PULPROG zgpg30
TD 65536
SOLVENT CDCl3
NS 1024
DS 4
SWH 24038.461 Hz
FIDRES 0.366798 Hz
AQ 1.3631988 sec
RG 196.87
DW 20.800 usec
DE 6.50 usec
TE 298.7 K
D1 2.00000000 sec
D11 0.03000000 sec
TD0 1

======== CHANNEL f1 =====
SFO1 100.6228293 MHz
NUC1 13C
P1 10.00 usec
SI 32768
SF 100.6127827 MHz
WDW EM
SSB 0
LB 1.00 Hz
GB 0
PC 1.40

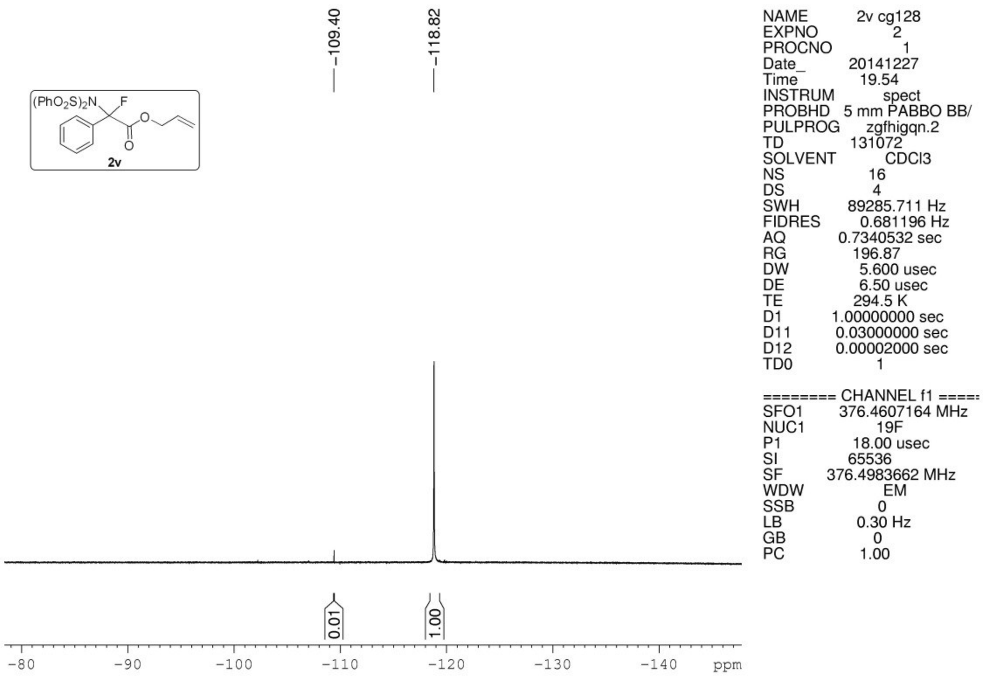

-109.40
-118.82
$(PhO_2S)_2N$ F
O
2v
0.01
1.00
-80 -90 -100 -110 -120 -130 -140 ppm
NAME 2v cg128
EXPNO 2
PROCNO 1
Date_ 20141227
Time 19.54
INSTRUM spect
PROBHD 5 mm PABBO BB/
PULPROG zgfhigqn.2
TD 131072
SOLVENT CDCl3
NS 16
DS 4
SWH 89285.711 Hz
FIDRES 0.681196 Hz
AQ 0.7340532 sec
RG 196.87
DW 5.600 usec
DE 6.50 usec
TE 294.5 K
D1 1.00000000 sec
D11 0.03000000 sec
D12 0.00002000 sec
TD0 1
======== CHANNEL f1 ========
SFO1 376.4607164 MHz
NUC1 19F
P1 18.00 usec
SI 65536
SF 376.4983662 MHz
WDW EM
SSB 0
LB 0.30 Hz
GB 0
PC 1.00

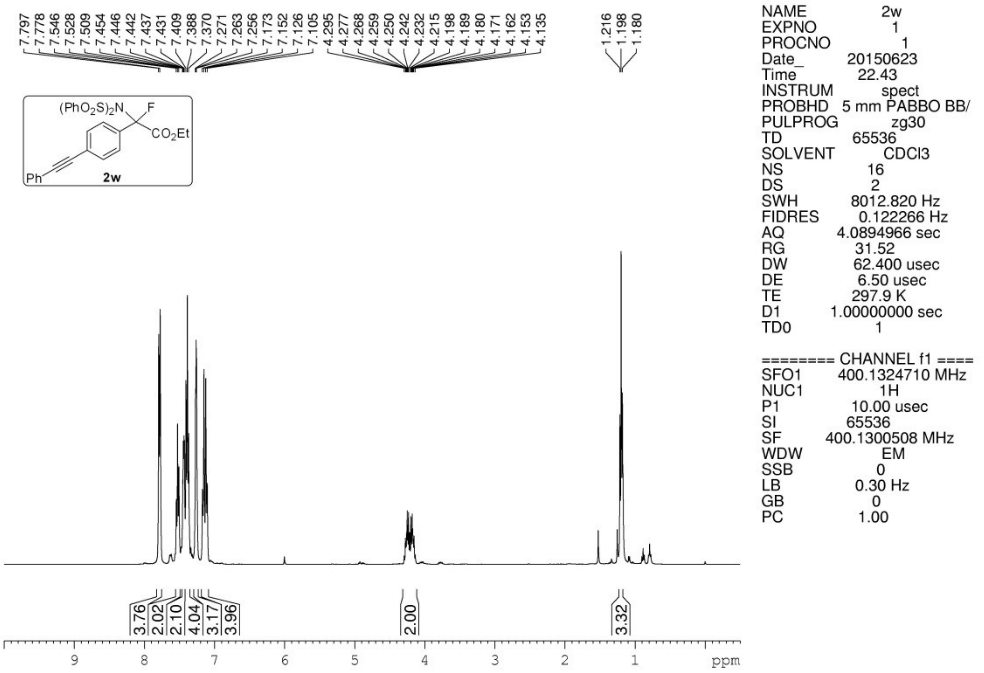

7.797 7.778 7.546 7.528 7.509 7.454 7.446 7.442 7.437 7.431 7.409 7.388 7.370 7.271 7.263 7.256 7.173 7.152 7.126 7.105 4.295 4.277 4.268 4.259 4.250 4.242 4.232 4.215 4.198 4.189 4.180 4.171 4.162 4.153 4.135
1.216 1.198 1.180
$(PhO_2S)_2N$ F
CO_2Et
Ph
2w
3.76 2.02 2.10 4.04 3.17 3.96
2.00
3.32
9 8 7 6 5 4 3 2 1 ppm
NAME 2w
EXPNO 1
PROCNO 1
Date_ 20150623
Time 22.43
INSTRUM spect
PROBHD 5 mm PABBO BB/
PULPROG zg30
TD 65536
SOLVENT CDCl3
NS 16
DS 2
SWH 8012.820 Hz
FIDRES 0.122266 Hz
AQ 4.0894966 sec
RG 31.52
DW 62.400 usec
DE 6.50 usec
TE 297.9 K
D1 1.00000000 sec
TD0 1
======== CHANNEL f1 ========
SFO1 400.1324710 MHz
NUC1 1H
P1 10.00 usec
SI 65536
SF 400.1300508 MHz
WDW EM
SSB 0
LB 0.30 Hz
GB 0
PC 1.00

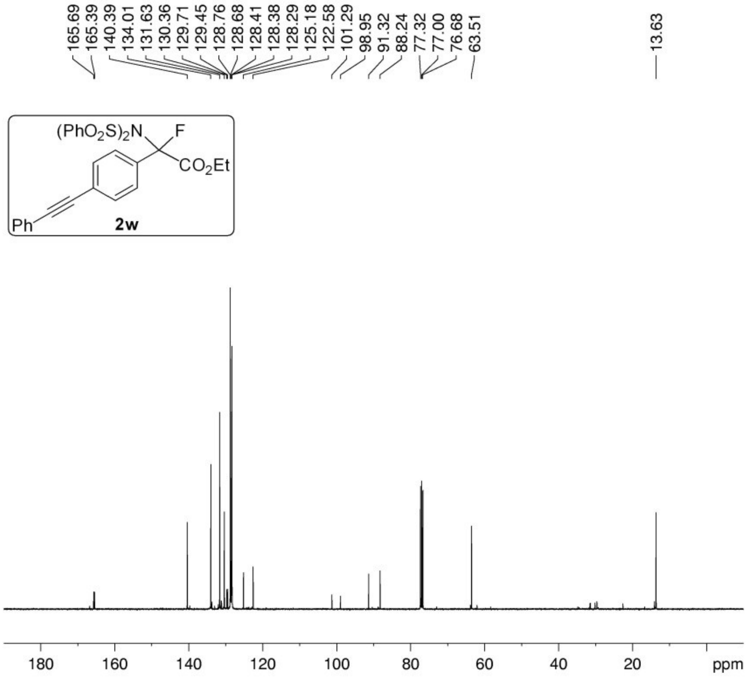

NAME cg–2w
EXPNO 3
PROCNO 1
Date_ 20150623
Time 23.38
INSTRUM spect
PROBHD 5 mm PABBO BB/
PULPROG zgpg30
TD 65536
SOLVENT CDCl3
NS 900
DS 4
SWH 24038.461 Hz
FIDRES 0.366798 Hz
AQ 1.3631988 sec
RG 196.87
DW 20.800 usec
DE 6.50 usec
TE 298.7 K
D1 2.00000000 sec
D11 0.03000000 sec
TD0 1

======== CHANNEL f1 =====
SFO1 100.6228293 MHz
NUC1 13C
P1 10.00 usec
SI 32768
SF 100.6127798 MHz
WDW EM
SSB 0
LB 1.00 Hz
GB 0
PC 1.40

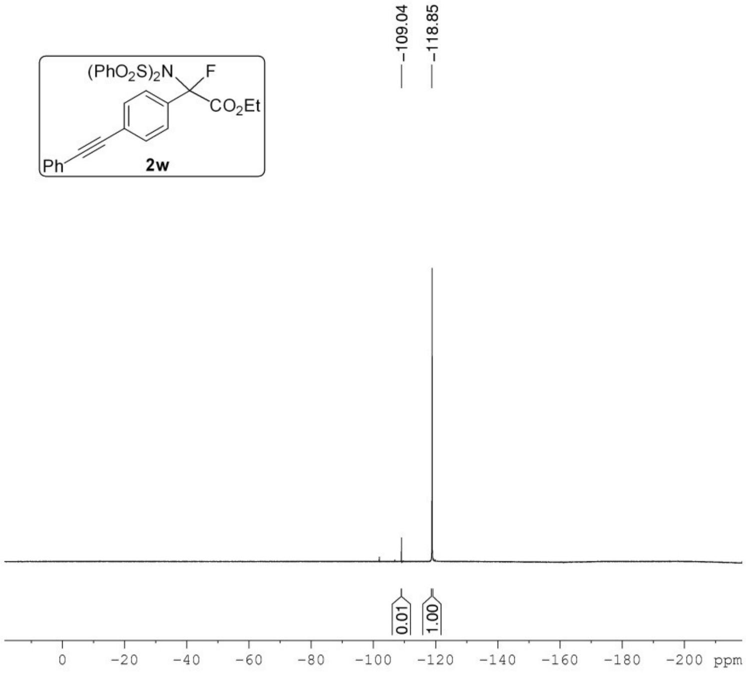

NAME cg–2w
EXPNO 2
PROCNO 1
Date_ 20150623
Time 22.45
INSTRUM spect
PROBHD 5 mm PABBO BB/
PULPROG zgfhigqn.2
TD 131072
SOLVENT CDCl3
NS 16
DS 4
SWH 89285.711 Hz
FIDRES 0.681196 Hz
AQ 0.7340532 sec
RG 196.87
DW 5.600 usec
DE 6.50 usec
TE 298.0 K
D1 1.00000000 sec
D11 0.03000000 sec
D12 0.00002000 sec
TD0 1

======== CHANNEL f1 =====
SFO1 376.4607164 MHz
NUC1 19F
P1 18.00 usec
SI 65536
SF 376.4983662 MHz
WDW EM
SSB 0
LB 0.30 Hz
GB 0
PC 1.00
